# Investigations of Intramolecular and Intermolecular Noncovalent Interactions in Alkaline Earth Metal Complexes of ^–^OC(Ph)(CF_3_)_2_

**DOI:** 10.1021/acs.inorgchem.6c01110

**Published:** 2026-06-12

**Authors:** Anna Y. O’Brien, Yuriko Takahashi, Miriam Gillett-Kunnath, Damian G. Allis, Ana Torvisco, Karin Ruhlandt-Senge

**Affiliations:** † Department of Chemistry, 7809 Le Moyne College, 1419 Salt Springs Rd, Syracuse, New York 13214, United States; ‡ Department of Chemistry, 1-014 Center for Science and Technology, 2029 Syracuse University, Syracuse, New York 13244, United States; § Institute of Inorganic Chemistry, Technical University of Graz, 8010 Graz, Austria; ∥ Department for Physical and Environmental Sciences, University of Toronto, Scarborough, ON M1C 1A4, Canada

## Abstract

Alkaline earth metals are relatively abundant, inexpensive and desirable as metal–organic complexes for applications in electronics and catalysis. Synthetic challenges arise from their high reactivity and the tendency of their compounds to form aggregates. Previous work demonstrated that certain fluorinated alkoxide ligands improved the volatility of alkaline earth metal and mixed-metal complexes, in part due to the presence of noncovalent secondary M···F interactions. This work further explores the impact of secondary interactions on their structure. The fluorinated alkoxide 2,2,2,2′,2′,2′-hexafluorocumyl alkoxide, ^–^OC­(Ph)­(CF_3_)_2_ (“L”), introduces the possibility of both M···F and M···π stabilizing interactions. Synthetic methods including alkane elimination (Mg), direct metalation (Ca, Sr, Ba), and protonolysis (Ba) with complexation to L in the controlled presence and/or absence of neutral donors afforded [MgL_2_(thf)_2_] (**1**), [*trans*-CaL_2_(thf)_4_] (**2**), [*cis*-SrL_2_(thf)_4_] (**3**), [Sr_2_(μ^2^-L)_3_L­(thf)_3_] (**4**), [Sr_3_(μ^2^-L)_4_L_2_(OEt_2_)_2_] (**5**), [Ba­(μ^2^-L)_2_]_*n*_· $\frac{1}{4}$ ­(OEt_2_) (**6**), and [Ba­(μ^2^-L)_2_]_*n*_ (**7**). Structural characterization shows the number of secondary interactions increase for the heavier metals and decreasing presence of the neutral donor, coinciding with increased nuclearity. Computational studies predict notable differences between preferred configurations that might exist in the gas-phase and those observed in the solid state.

## Graphical abstract

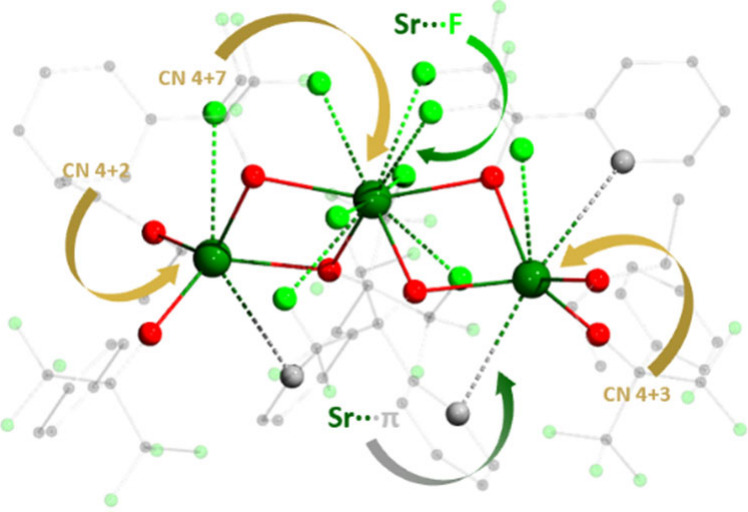

## 1 Introduction

A resurgence of interest in alkaline earth metal–organic complexes over the past 25 years, primarily motivated by applications in catalysis^1–8^ and precursors for electronic materials,^9–14^ was made possible by improved synthetic methodologies.^15–21^ While the alkaline earth metals, Mg, Ca, Sr, and Ba, are relatively abundant, and in the case of Mg and Ca, biocompatible, their metal–organic complexes require careful synthesis and handling under inert conditions due to their reactivity.^22–25^ In the presence of air or moisture, the metals preferentially form oxides and hydroxides over the desired metal–organic complexes due to their strong electropositive character and negative reduction potentials. The increasing ionic radii^26^ descending from Mg^2+^ (1.12 Å) to Ba^2+^ (1.91 Å) require higher coordination numbers for the heavier analogs to achieve steric saturation, with a significant tendency toward aggregation and formation of coordination polymers, thus limiting their solubility and volatility. Further, the ionic nature of the metal–ligand bonds increases from Mg^2+^ to Ba^2+^, making their complexes in solution increasingly labile and susceptible to ligand redistribution. Their increasing ionic nature also limits solubility of the complexes in nonpolar solvents.^2,6,23^

Applications of alkaline earth metal–organic complexes as chemical vapor deposition (CVD) precursors for ferroelectric materials^10^ and high-*T*_c_ superconductors^27^ require them to be volatile and thermally stable to reach the substrate, yet decompose cleanly without contaminating the deposited film. To suppress the tendency of the heavier alkaline earth compounds to form high molecular weight aggregates, neutral donors, such as triglyme (CH_3_(OCH_2_CH_2_)_3_OCH_3_) and tetraglyme (CH_3_(OCH_2_CH_2_)_4_OCH_3_), are utilized in addition to anionic ligands. However, upon heating, the loss of neutral donor typically leads to aggregation, accompanied by loss of volatility.^10^ Another approache to address this problem is to modify the anionic ligand,^28^ classic examples being β-diketonates,^29^ β-ketoiminates,^30^ β-ketiminates,^31^ alkylamides,^32^ and alkoxides,^33–38^ with the most common modification the introduction of fluorinated substituents.^9^ The hydrophobic fluorine may aid in minimizing moisture-sensitivity, while the high electronegativity of the fluorine improves thermal stability and reduces the basicity of the ligand, leading to reduced aggregation tendencies of the resulting compounds.^9,10^ The presence of fluorinated substituents also creates the possibility of stabilizing M···F secondary interactions.^9^ The downside of these is the tendency for the unwanted incorporation of fluorine in the metal oxide film, which might be minimized by incorporating water in the reaction gas to remove the fluorine as HF with the detriment of oxide or hydroxide formation.^9^

To explore the potentially stabilizing effect of M···F secondary interactions on alkaline earth metal complexes, we considered fluorinated alkoxides (Figure 1) as they are readily available and tunable, allowing us to build on our previous work. Fluorinated isopropoxide was among the first ligands to be complexed to an alkaline earth metal for CVD applications, including hexafluoroisopropoxide (^−^OCH­(CF_3_)_2_) (L^1^, Figure 1) in the barium cluster [Ba_5_(OH)­(L^1^)_9_(thf)_4_(H_2_O)·THF].^39^ Hexafluoroisopropoxide (L^1^) has since been reported in a small number of alkaline earth metal and heterobimetallic complexes.^35,40–43^ A number of examples include the trifluorinated phenyl-substituted alkoxide ligand ^–^OC­(H)­(Ph)­(CF_3_) (L^2^, Figure 1)^44^ along with further variations of partially fluorinated alkoxide alkaline earth metal compounds.^45–47^ However, the partially fluorinated L^1^ is especially prone to undesired decomposition in CVD applications. For example, NaL^1^ decomposes to NaF in the gas phase through intramolecular Na···F interaction, 1,2-migration of the proton, and loss of the fluorinated ketone.^48^

**Figure 1:**
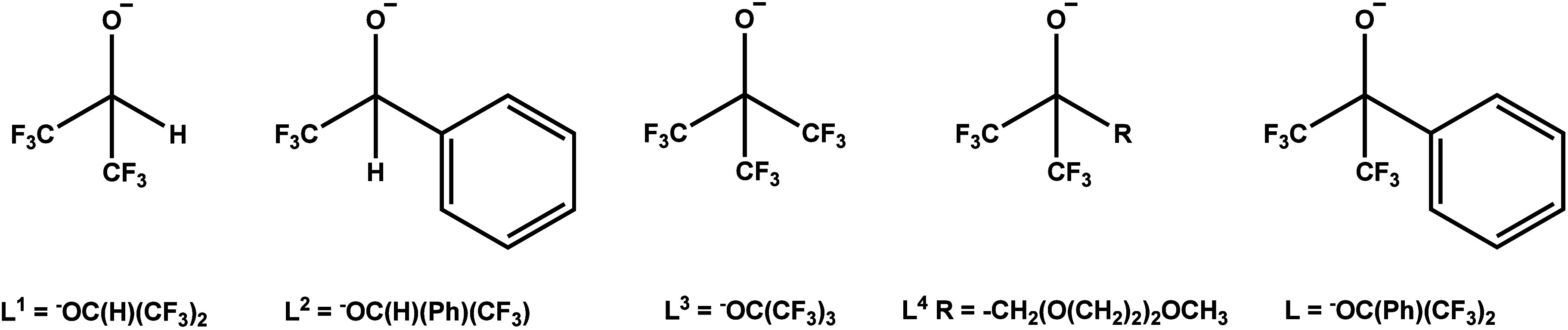
Previously reported fluoroalkoxides in alkaline earth metal complexes include partially fluorinated alkoxides (L^1^, L^2^), the perfluorinated alkoxide ligand, ^–^OC­(CF_3_)_3_ (L^3^), and a dozen fluorinated alkoxides with neutral donor appendages (L^4^ shown here as an example). Of interest in this paper is the phenyl-substituted fluoroalkoxide ligand ^–^OC­(Ph)­(CF_3_)_2_ (L).

It was not until the introduction of the fully fluorinated ^–^OC­(CF_3_)_3_ ligand that the potential of fluorination, in order to build volatile compounds that sublime mostly intact, could be realized.^13^ The perfluoroalkoxide ligand, ^–^OC­(CF_3_)_3_ (L^3^, Figure 1), allowed the synthesis of three series of highly volatile monomeric homometallic complexes [Ae­(L^3^)_2_(donor)_*x*_] (Ae = Mg, Ca, Sr, and Ba; donors = tetrahydrofuran (thf), 1,2-dimethoxyethane (dme), and diglyme).^13^ The hydrophobicity of the perfluoroalkoxide ligand made these complexes air-stable, and the electronegative −CF_3_ groups helped reduce aggregation, leading to excellent volatility.^13^ These successes led us to investigate mixed-metal alkali and alkaline earth metal complexes of L^3^ as single-source precursors for CVD, which are desirable for their precise stoichiometric control over the deposited film.^49^ The heterobimetallic complexes [AM­(L^3^)_3_(coligand)_*x*_]_4_ (where A = Na, K; M = Mg, Sr, Ba, Eu; coligand = thf, toluene) were prepared, with [KSr­(L^3^)_3_(thf)]_4_ demonstrating high volatility.^49^ The presence of numerous M···F interactions impacted the solid state structures by saturating the coordination sphere of the metals. The perfluorinated alkoxide (L^3^) has appeared in the literature in a number of other alkaline earth metal complexes including [*cis*-Mg­(L^3^)_2_(dme)_2_],^50^ [Ba­(μ-L^3^)_2_L^3^(thf)_3_Ag­(thf)],^40^ [MgNa_2_(μ-L^3^)_4_(dme)_2_],^51^ and the charge separated [Sr­(L^3^)­(thf)_5_] [Al­(L^3^)_4_].^52^ The perfluorinated ligand is also found in the aluminate ion [Al­(L^3^)_4_]^−^ paired with magnesium complex cations for battery applications,^53,54^ and with Mg, Ca, Sr, and Ba-arene complexes for catalysis.^7,55^

Fluoroalkoxide ligands have also been modified to incorporate a neutral donor appendage arm including ethers and amines (L^4^, Figure 1, and L^5^-L^15^, Figure S1, SI). One reason to attach the neutral donor to the ligand is to prevent its loss upon heating. Examples include the homoleptic [ML^4^_2_], [ML^5^_2_]_2_,^14^ and [ML^6^_2_],^56^ (M = Sr and Ba, L^4–6^ = see Figure S1, SI), for depositing the corresponding metal fluoride thin films. Sarazin et al. have reported nearly two dozen alkaline earth metal complexes of this type, mostly heteroleptic with silylamido ligands for enhanced solubility, for applications as catalysts,^57–61^ as well as homoleptic macrocyclic-functionalized bisfluoroalkoxides [SrL^7^·thf], [SrL^7^·H_2_O], [BaL^7^]_2_, and [ML^8^] (M = Ca, Sr, and Ba, L^7,8^ = see Figure S1, SI).^8^ All report on the contribution of M···F secondary interactions to the stability of the complexes, especially for metals with enhanced electrophilicity (Ca < Sr < Ba), fewer neutral donor atoms coordinating, and lower coordination numbers in general (Table S3, SI).

In addition to M···F noncovalent interactions, we also wanted to explore the secondary interaction between a metal cation and the π-system of a phenyl ring (M···π). Our previous work emphasized the significance of M···π interactions in the absence of neutral donors, as shown with a family of heavy alkaline earth metal pyrazolates [{M­(3,5-di-*tert*-butylpyrazolate)_2_}_n_] (M = Ca, n = 3; M = Sr, n = 4; M = Ba, n = 6),^62^ the potassium 2,6-diphenylphenolate (Odpp) coordination polymer [K­(Odpp)]_∞_,^63^ and separated ions of tetraphenylborate [M­(donor)_n_]­[BPh_4_]_2_ (M = Ca, Sr, Ba; donor = [18]-crown-6, thf, hexamethylphosphoramide, dme, and acetonitrile).^64^

To explore the potential role of both M···F and M···π interactions in alkaline earth metal complexes, we have chosen the ligand 2,2,2,2′,2′,2′-hexafluorocumyl alkoxide, ^–^OC­(Ph)­(CF_3_)_2_ (L, Figure 1). This ligand has been used previously with aluminum,^65^ in lithium aluminates for catalysis,^66–69^ and with lithium and titanium for battery applications.^70^ More recently, Doerrer *et. al* have complexed this ligand to copper and zinc for catalysis.^71,72^ Of note is that the heterobimetallic complexes deprotonate the ligand phenyl ring, forming the Cu­(III) species, [K([18]­crown-6)]­[Cu^III^(OC­(C_6_H_4_)­(CF_3_)_2_)_2_].^73^ Rare earth complexes of this ligand, [Er­(L)_3_(Ph_3_PO)_2_][Bibr ref74] and {[Ph­(CF_3_)_2_CO]_2_Er­(μ^2^-L)}_2_,^75^ have also been isolated in the quest for single molecular magnets. A CSD search revealed a single Mg­(L)_2_(dme)_2_ structure.^76^

These advances merit further investigations into the use of the phenyl-substituted fluoroalkoxide ligand, L, for complexation with alkaline earth metals. Much remains to be understood about controlling the structures of their coordination complexes and thus the physical properties of potential precursor molecules. In this work, Mg, Ca, Sr, and Ba complexes [MgL_2_(thf)_2_] **(1)**, [*trans*-CaL_2_(thf)_4_] **(2)**, [*cis*-SrL_2_(thf)_4_] **(3)**, [Sr_2_(μ^2^-L)_3_L­(thf)_3_] **(4)**, [Sr_3_(μ^2^-L)_4_L_2_(OEt_2_)_2_] **(5)**, [Ba­(μ^2^-L)_2_]_n_· $\frac{1}{4}$ ­(OEt_2_) **(6)**, and [Ba­(μ^2^-L)_2_]_n_ **(7)** were afforded using synthetic routes established by our group and others for alkaline earth amides, pyrazolates, aryl, benzyl, alkoxides, thiolates, and selenolates.^15,17,18,23,32^ These synthetic routes also allowed for control over the presence of neutral donor, so as to explore their impact on secondary interactions. X-ray crystallographic data (of **1**-**7**) provide a means to assess the presence of M···F^77^ and M···π ^62–64^ interactions, as well as additional secondary noncovalent interactions between the ligands and neutral donors (π···π, π···H, and F···H, see Figure 2) in the solid state. Density functional theory (DFT) calculations (of **1**-**4**) provide the likely preferred configurations in gas phase and solution for comparison.

**Figure 2:**
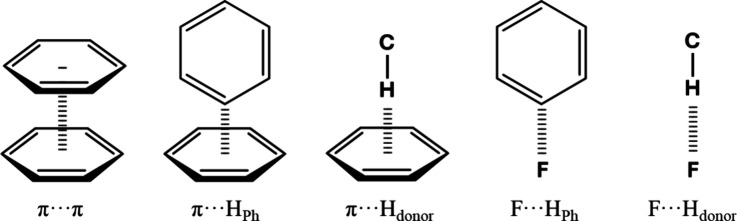
In addition to M···F and M···π secondary interactions, these secondary noncovalent interactions between the ligands and neutral donors are explored. Abbreviations used in this paper are shown, and justification for interaction cutoffs is given in Table S1, SI.

## 2 Experimental Section

### 2.1 Synthesis and Characterization

WARNING: Di-*n*-butyl magnesium, calcium metal, strontium metal, and barium metal in contact with water are highly reactive and emit flammable gases. These reagents and their products were handled in an inert-atmosphere glovebox or under nitrogen gas on a Schlenk line. Extreme care should be taken both in the handling of the cryogen liquid nitrogen and its use in the Schlenk line trap to avoid the condensation of oxygen from air.

WARNING: Liquified ammonia is highly corrosive, toxic, and poses severe danger to skin and eyes, requiring personal protective equipment (PPE) and strict handling protocols to avoid inhalation or contact. Reaction vessels containing liquified ammonia were at all times submerged in a −78 °C acetone/dry ice bath, behind a blast shield in a hood. Liquified ammonia was allowed to evaporate to room temperature with slow warming of the cooling bath. Uncontrolled warming of the liquified ammonia to room temperature will result in the rapid expansion of the corrosive, toxic ammonia gas, potentially bursting the container and posing extreme risk.

WARNING: The thick-walled Pyrex tube contents are under high pressure and measures must be taken to prevent tube bursting and potential injury. Tubes are sealed under vacuum in a hood behind a blast shield, and must be transported in metal sleeves. Reactions are conducted behind a blast shield in a hood. It was ensured that the working pressure inside the sealed tube never exceeded 10 bar, limiting volatile solvents to 1.0 mL of Et_2_O in a tube volume of 10 mL at 35 °C. Although it is not possible to determine the exact pressure as the reaction occurs, it can be assumed the pressure did not exceed safe operating levels with this estimation.

All manipulations were carried out in a N_2_ inert atmosphere using a glovebox and Schlenk-line techniques. 1,1,1,3,3,3-hexafluoro-2-phenyl-2-propanol (TCI, > 99.0% pure) was dried over calcium hydride (Acros, ca. 93% extra pure, 0–2 mm size, rinsed three times with dried and degassed hexanes) by reflux overnight and distilled prior to use. Anhydrous ammonia was prepared by condensing NH_3_ gas over sodium metal using an acetone/dry ice bath at −78 °C. The dried condensed ammonia was allowed to evaporate and was recondensed into the reaction mixtures. Ammonia gas is acutely toxic and corrosive. Exercise extreme caution when handling condensed gases at low temperatures, as even small increases in temperature can cause rapid expansion of the gas beyond the size of the glassware. All solvents including tetrahydrofuran, toluene, hexanes, diethyl ether, were dried using a solvent purification system and degassed with three freeze–pump–thaw cycles prior to use. Di-*n*-butyl magnesium [CH_3_(CH_2_)_3_]_2_Mg (Sigma-Aldrich, 1.0 M solution in heptanes), calcium turnings (Sigma-Aldrich, 99%), strontium pieces (STREM Chemicals, 99%), and barium metal (CERAC, 99.7%) were used as received. The barium hexamethyldisilazide reagent, [Ba­(N­(Si­(CH_3_)_3_)_2_)_2_(thf)_2_], was prepared according to literature methods.^19^

Transmission IR spectra were collected on KBr plates as Nujol mulls using a Nicolet L200 FTIR spectrometer (Nicolet, Prague, Czech Republic). Transmission data between 4000 to 400 cm^–1^ were collected. Any IR peaks overlapping with Nujol are omitted in the summarized data. ^1^H, ^13^C, and ^19^F NMR data were collected on 300 and 400 MHz Bruker Avance spectrometers (Bruker, Billerica, Massachusetts, USA). Chemical shifts for ^1^H and ^13^C are referenced to the solvent (benzene, 7.16 ppm; TMS in THF, 0.00 ppm) and ^19^F are corrected against an internal standard (trifluoroacetic acid, CF_3_COOH, −76.55 ppm) added via a capillary tube to C_6_D_6_ solutions of compounds **1**-**4**, and an external standard (trifluorotoluene, PhCF_3_, in benzene −62.74 ppm) for compounds **5**-**7**.^78^ Compounds were grease-sealed in melting point tubes under N_2_, and uncorrected melting points were collected on a Mel-temp II benchtop device. TGA were performed on representative compounds **2** and **3** on a TA Q 500 Instrument. Sample sizes of 13 mg and 18 mg, respectively, were quickly loaded in the air onto platinum pans and heated from room temperature to 800 °C at a rate of 10 °C per minute. Purified nitrogen gas was used for all samples, with a balance purge rate of 40 mL/min and a sample purge rate of 60 mL/min.

Characterization of the bulk compounds by elemental analysis was not successful due to well-established challenges with desolvation, air- and moisture-sensitivity, along with the formation of nonvolatile oxides reported for similar compounds.^8,13,44^ As such, the formulas for the described compounds are based on the crystallographic data and are further supported by spectroscopic data (IR, multinuclear NMR). ^1^H NMR integration values for neutral donors are inconsistent with crystallographic formulas due to the need to remove crystals from the mother liquor and subsequent evacuation to place in the glovebox for sample preparation. To prevent loss of donor, attempts were also made to preserve some of the mother liquor, resulting in excess donor in some NMR spectra, as has also been observed previously.^13,62^

The general procedure for direct metalation via ammonia activation for **2**, **3**, **5**, and **6** is as follows. A Schlenk tube was charged with metal filings, HOC­(C_6_H_5_)­(CF_3_)_2_, and either THF or toluene, and cooled to −78 °C utilizing an acetone/dry ice bath. Anhydrous ammonia (15 mL), as prepared above, was recondensed into the reaction mixture, during which the solution turned blue. After 12 h, the acetone/dry ice bath was removed and the solution was gradually allowed to warm to room temperature while stirring, upon which the solution became yellow. All remaining volatiles were removed by evacuation and the residue was redissolved in 20 mL of THF (**2** and **3**) or rinsed with toluene and redissolved in diethyl ether (**5** and **6**). The resulting solution was filtered through a filter frit and then concentrated to saturation by vacuum. Details of scale and crystallization conditions are described for each.

#### [Mg­(OC­(Ph)­(CF_3_)_2_)_2_(thf)_2_] (**1**)

Di-*n*-butyl magnesium (3 mmol, 1 M in heptanes, 3 mL) was slowly added via syringe to HOC­(C_6_H_5_)­(CF_3_)_2_ (6 mmol, 1.01 mL) in 10 mL of toluene giving a clear yellow solution. After stirring for 3 h at room temperature, all volatiles were removed by vacuum resulting in a yellow solid, which was redissolved in a minimal amount of toluene and THF and stored at −23 °C. Colorless crystals (block) formed in 1 day, yielding 0.71 g (36%). Decomposed 184–185 °C. ^1^H NMR (C_6_D_6_, 300 MHz): δ 8.17­(d, 4H, *J* = 7.4 Hz), 7.23 (t, 4H, *J* = 7.3 Hz), 7.11 (m, 2H), 3.57 (m, 15H), 1.19 (m, 15H). ^13^C NMR (C_6_D_6_, 75 MHz): δ 128.9, 128.2, 127.9, 69.4, 25.2. ^19^F NMR (C_6_D_6_, 282 MHz) δ −76.96. IR (Nujol mull, cm^–1^): 3348­(w), 3168­(w), 2029­(w), 1969­(w), 1895­(w), 1825­(w), 1759­(w), 1692­(w), 1681­(w), 1666­(w), 1603­(w), 1567­(w), 1555­(w), 1262(s), 1213­(m), 1167­(m), 1076­(m), 1019­(m), 968­(m), 935­(w), 917­(w), 799­(m), 770­(w).

#### [*trans*-Ca­(OC­(Ph)­(CF_3_)_2_)_2_(thf)_4_] (**2**)

The general procedure for DM was carried out for calcium metal (2.5 mmol, 0.10 g) and HOC­(Ph)­(CF_3_)_2_ (5 mmol, 0.84 mL) in THF (10 mL). The concentrated filtrate was layered with hexanes and stored at −23 °C to crystallize. Colorless crystals (plates) formed in 2 days, yielding 1.36 g (67%). Decomposed 274–278 °C. ^1^H NMR (C_6_D_6_, 300 MHz): δ 7.64 (bs, 4H), 7.01 (m, 6H), 3.49 (t, 7H, *J* = 6.6 Hz), 1.32 (m, 7H). ^13^C NMR (C_6_D_6_, 75 MHz): δ 128.7, 128.1, 127.8, 67.9, 25.6. ^19^F NMR (C_6_D_6_, 282 MHz) δ −76.87. IR (Nujol mull, cm^–1^): 3363­(w), 3172­(w), 1958­(w), 1599­(w), 1549­(w), 1269­(m), 1234­(m), 1213­(m), 1167­(m), 1078­(w), 1016­(w), 968­(m), 937­(w), 921­(w), 760­(w).

#### [*cis*-Sr­(OC­(Ph)­(CF_3_)_2_)_2_(thf)_4_] (**3**)

The general procedure for DM was carried out for strontium metal (6 mmol, 0.53 g) and HOC­(Ph)­(CF_3_)_2_ (12 mmol 2.02 mL) in THF (20 mL). The concentrated filtrate was layered with hexanes and stored at −23 °C to crystallize. Colorless crystals (plate) formed in 2 days, yielding 1.64 g (32%). Decomposed 276–277 °C. ^1^H NMR (C_6_D_6_, 300 MHz): δ 7.95 (d, 4H, *J* = 6.4 Hz), 7.18 (t, 4H, *J* = 7.7 Hz), 7.07 (t, 2H, *J* = 7.2 Hz), 3.45 (m, 10H), 1.28 (m, 10H). ^13^C NMR (C_6_D_6_, 75 MHz): δ 129.5, 128.8, 128.1, 127.8, 127.2, 68.0, 25.4. ^19^F NMR (C_6_D_6_, 282 MHz) δ −76.86. IR (Nujol mull, cm^–1^): 3177­(m), 3063­(w), 2022­(w), 1958­(w), 1888­(w), 1820­(w), 1759­(w), 1678­(w), 1603­(w), 1585­(w), 1269(s), 1230(s), 1213(s), 1994(s), 1175(s), 1104­(m), 1075­(m), 1058­(m), 1031­(m), 1005­(w), 967(s), 934(s), 916(s), 893­(m), 761­(m), 713(s).

#### [Sr_2_(μ^2^-OC­(Ph)­(CF_3_)_2_)_3_(OC­(Ph)­(CF_3_)_2_)­(thf)_3_] (**4**)

In a 100 mL Schlenk tube, **3** as prepared above was redissolved in 20 mL of toluene. The solution was stirred for 1 h, filtered through a filter frit, concentrated, and layered with hexanes. This solution was stored at −23 °C to crystallize. Colorless crystals (plates) formed in 2 days, yielding 1.43 g (35%). Decomposed 191–192 °C. ^1^H NMR (C_6_D_6_, 300 MHz): δ 7.93 (bs, 8H), 7.72–7.85 (m, 4H), 6.65–7.07 (m, 8H), 3.32 (t, 15H, *J* = 6.1 Hz), 1.23 (t, 17H, *J* = 6.4 Hz). ^13^C NMR (C_6_D_6_, 75 MHz): δ 129.6, 128.9, 128.1, 127.8, 127.1, 68.0, 25.3. ^19^F NMR (C_6_D_6_, 282 MHz) δ −76.78. IR (Nujol mull, cm^–1^): 3354­(w), 3163­(w), 2029­(w), 1958­(w), 1887­(w), 1820­(w), 1666­(w), 1591­(w), 1272­(m), 1167­(m), 1076­(w), 1030­(w), 962­(m), 938­(m), 892­(w), 761­(w).

#### [Sr_3_(μ^2^-(OC­(Ph)­(CF_3_)_2_)_4_(OC­(Ph)­(CF_3_)_2_)_2_(OEt_2_)_2_] (**5**)

The general procedure for DM was carried out for strontium metal (10 mmol, 0.87 g) and HOC­(Ph)­(CF_3_)_2_ (20 mmol, 3.3 mL) in 20 mL toluene. The concentrated yellow filtrate was stored at −23 °C to crystallize. Colorless crystals (plate) formed in 5 days, yielding 0.50 g (8%). Decomposed 172 °C. ^1^H NMR (C_6_D_6_, 400 MHz): δ 7.96 (d, 2H, *J* = 5.7 Hz), 7.86 (t, 4H, *J* = 7.7 Hz), 7.72 (d, 4H, *J* = 7.3 Hz), 7.56 (bs, 6H), 6.9–7.1 (m), 6.81 (m, 4H), 6.70 (m, 2H), 3.26 (q, 6H, *J* = 7.0 Hz), 1.11 (t, 9H, *J* = 7.0 Hz). ^13^C NMR (C_6_D_6_, 100 MHz): δ 136.1, 135.7, 130.2, 129.2, 128.8, 128.4, 128.1, 128.0, 127.5, 127.4, 127.2, 126.8, 65.8, 15.4. ^19^F NMR (C_6_D_6_, 376 MHz) δ −73.85 (bs, 6H), −74.44 (6H), −74.95 (6H), −75.91 (6H), −76.86 (12H). IR (Nujol mull, cm^–1^): 3171­(w), 3111­(w), 3065­(w), 2025­(w), 1962­(w), 1893­(w), 1820­(w), 1712­(m), 1681­(w), 1604­(w), 1585­(w), 1564­(w), 1554­(w), 1537­(w), 1497­(m), 1272(s), 1214­(m), 1195­(m), 1169(s), 1131­(m), 1076­(m), 1035­(w), 1004­(w), 958­(m), 940­(m), 915­(m), 846­(w), 796­(w), 754­(w), 713(s).

#### [Ba­(μ^2^-OC­(Ph)­(CF_3_)_2_)_2_]_*n*_· $\frac{1}{4}$ ­(OEt_2_) (**6**)

The general procedure for DM was carried out for barium metal (10 mmol, 1.37 g) and HOC­(Ph)­(CF_3_)_2_ (20 mmol, 3.3 mL) in 20 mL toluene. Slow evaporation of the diethyl ether solvent at room temperature over 12 h yielded colorless crystals (block), 3.12 g (49%). Decomposed 260 °C. ^1^H NMR (*d*_8_-THF with TMS, 400 MHz): δ 7.70 (s, 4H), 7.24 (s, 6H), 3.29 (m, < 1H), 1.18 (s, < 1H). ^13^C NMR (*d*_8_-THF, 100 MHz): δ 129.5, 128.5, 128.3. ^19^F NMR (C_6_D_6_, 376 MHz) δ −73.19 to −73.46 (m, 1H), −73.75 to −74.72 (m, 4H), −75.15 to −75.46 (m, 2H), −76.61 to −76.69 (bm, 5H). IR (Nujol mull, cm^–1^): 3342­(w), 3175­(w), 3096­(w), 3077­(w), 3060­(w), 3027­(w), 2021­(w), 1962­(w), 1890­(w), 1817­(w), 1776­(w), 1765­(w), 1666­(w), 1603­(w), 1583­(w), 1548­(m), 1494­(m), 1322­(w), 1291­(m), 1263(s), 1225(s), 1164(s), 1128(s), 1108(s), 1076(s), 1033­(m), 1020­(m), 1003­(m), 992­(m), 964(s), 936(s), 915­(m), 859­(w), 844­(w), 804­(m), 759­(m), 750­(w), 711(s).

#### [Ba­(μ^2^-OC­(Ph)­(CF_3_)_2_)_2_]_n_ (**7**)

A heavy-walled Pyrex tube was charged with [Ba­(N­(Si­(CH_3_)_3_)_2_)_2_(thf)_2_] (0.05 mmol, 0.030 g) and HOC­(Ph)­(CF_3_)_2_ (0.10 mmol, 0.017 mL) in 1 mL of diethyl ether. The tube was removed from the glovebox, frozen in liquid nitrogen, and flame-sealed under vacuum. Storage at 35 °C (reflux for Et_2_O) for 3 weeks yielded colorless crystals (blocks) on walls of the tube at room temperature, 0.01 g (30%). Decomposed 260 °C. ^1^H NMR (*d*_8_-THF with TMS, 400 MHz): δ 7.74 (bs, 2H), 7.62 (m, 1H), 7.42–7.43 (m, 1.5 H), 7.33 (bs, 3H). ^13^C NMR (*d*_8_-THF, 100 MHz): δ 132.5, 131.0, 129.3, 128.7, 128.1, 128.0. ^19^F NMR (C_6_D_6_, 376 MHz) δ −73.46 (1H), −75.27 (bs, 11H). IR (Nujol mull, cm^–1^): 3394­(w), 3170­(w), 3095­(w), 3068­(m), 3030­(w), 2019­(w), 1958­(w), 1885­(w), 1812­(w), 1778­(w), 1758­(w), 1657­(w), 1628­(w), 1602­(w), 1585­(w), 1566­(w), 1496­(m), 1320(s), 1297(s), 1256(s), 1202(s), 1134(s), 1107(s), 1074(s), 1034­(m), 1002(s), 968(s), 942(s), 916(s), 879­(m), 848­(m), 813­(w), 759(s), 748(s), 713(s).

### 2.2 Crystallographic Characterization

Single crystal X-ray diffractometry was used to characterize compounds **1**-**7**. Due to the air-sensitive nature of the compounds, suitable single crystals were removed under inert gas from a Schlenk and immediately covered with a layer of highly viscous Paratone oil. XRD data collection was performed on a Bruker Kappa Duo diffractometer^79^ with Mo Kα radiation (λ = 0.71073 Å) and an Apex II CCD area detector. All software and refinement details, including disorder parameters used for **5** and **6** as well as twin refinement of **3** are found in the SI. CIF files for compound **1**-**7** are deposited with the CCDC (2487076–2487082) and can be obtained free of charge from The Cambridge Crystallographic Data Centre via www.ccdc.cam.ac.uk/data_request/cif.

### 2.3 Computation Details

Conformational sampling of **1–3** was performed from the crystallographic geometries of each with CREST-XTB^80,81^ using the GFN2-xTB method.^82^ Conformers obtained from these calculations were optimized with the B3LYP hybrid density functional,^83^ 6–31G­(d,p) basis set,^84^ and the D3 version of the Grimme dispersion correction with Becke-Johnson damping^85^ as a preparative step before optimizations with the range-corrected lc-ωPBE density functional,^86^ Def2-TZVP basis set,^87^ and the D3 version of the Grimme dispersion correction with Becke-Johnson damping. All DFT optimizations were performed with an implicit benzene Polarizable Continuum Model (PCM) self-consistent reaction field (SCRF) applied.^88^ Final geometries from the complete DFT survey were characterized as minima based on normal-mode analyses. Calculations were performed with Gaussian09 ver. D.01 ^89^ with program-option “ultrafine” grid sizes (integration grid of 99 radial shells and 590 angular points per shell) and “tight” convergence criteria (force criterion RMS < 1.0 × 10^–5^, density matrix RMS < 1.0 × 10^–8^). Images were generated with VMD^90^ and POV-Ray.^91^

## 3 Results

### 3.1 Synthesis and Structural Characterization

The synthetic routes available for the target compounds are determined by the reactivity of the metal. (Scheme 1) The alkane elimination synthetic route is limited to magnesium, compounds **2**-**6** were successfully synthesized by activation of the metal with liquid ammonia and subsequent treatment with alcohol, and barium compound **7** was prepared by treatment of barium hexamethyldisilazide with the alcohol.^18^

**Scheme 1:**
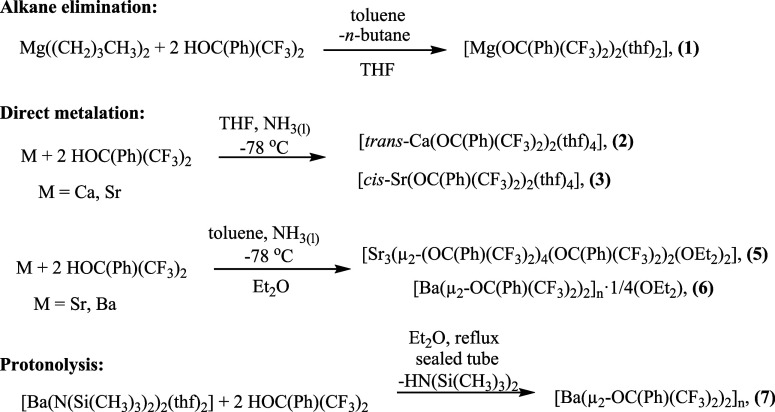
Synthetic Methods for Alkaline Earth Metal Complexes **1**–**3** and **5**–**7** ^a^

a Not shown, the dimeric [Sr_2_(μ^2^-OC­(Ph)­(CF_3_)_2_)_3_(OC­(Ph)­(CF_3_)_2_)­(thf)_3_] (**4**) was recrystallized from a solution of **3** in toluene. The crystallization solvent, if different than reaction solvent, is given below the arrow.

Alkane elimination of di-*n*-butyl magnesium in the presence of the fluorinated alcohol, followed by recrystallization from a mixture of toluene and THF, lead to the isolation of the magnesium monomer, [MgL_2_(thf)_2_] **(1)** (Figure 3). This is the first structurally characterized monomeric thf-adduct reported for magnesium with a fluoroalkoxide ligand; all others reported are either dme adducts or multinuclear: [Mg­(L^3^)_2_(dme)_2_],^50^ [MgNa_2_(μ-L^3^)_4_(dme)_2_],^51^ [Mg­(dme)_2_(μ-L^1^)_2_Mg­(L^1^)_2_],^42^ and [MgCl­(thf)­(μ-L^2^)_2_Mg­(thf)­(μ-Cl)_3_Mg­(thf)_3_],^44^ or fluoroaryloxides.^92–94^ Compound **1** is monomeric with distorted tetrahedral geometry about the Mg center (O3–Mg–O4 98.82(7)° to O1–Mg–O2 124.28(7)°), with the largest angle between the two ligands and the smallest angle between the two thf donors. As expected, the Mg–O bond lengths for the anionic ligands (1.863(2)-1.866(2) Å) are shorter than for the neutral thf donors (1.996(2)-2.005(2) Å).

**Figure 3:**
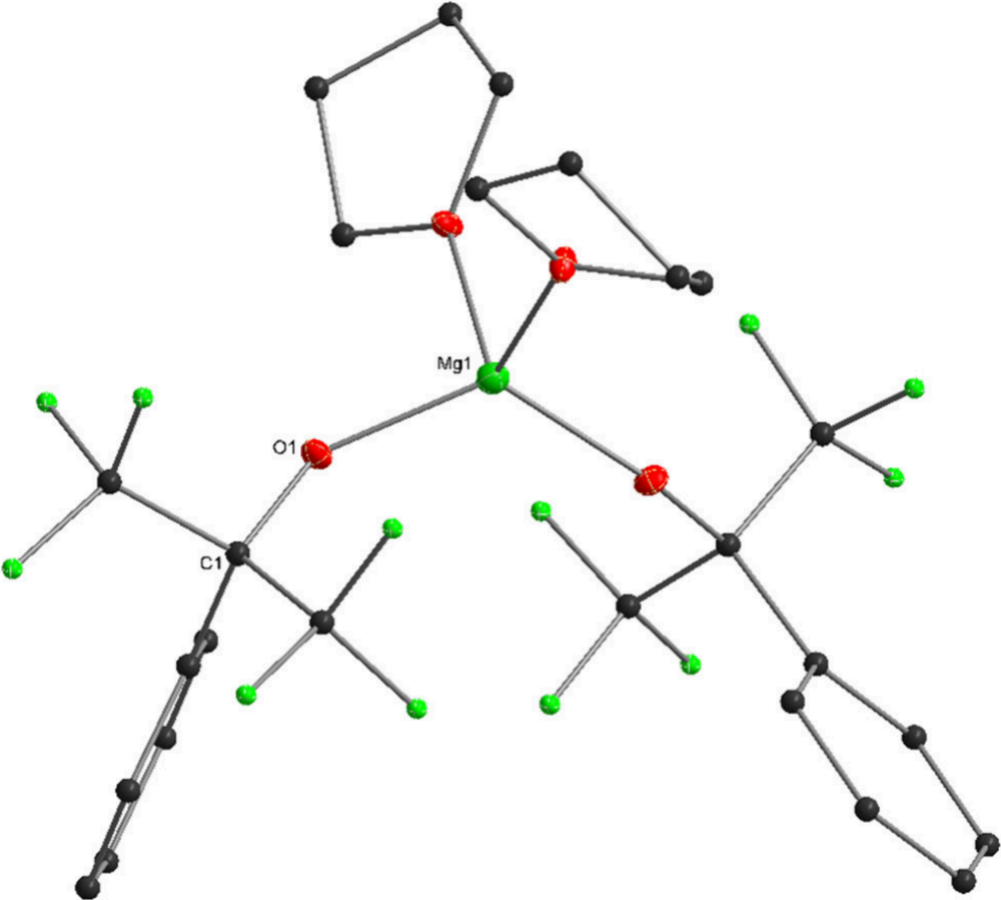
Crystal structure diagram for [Mg­(OC­(Ph)­(CF_3_)­2)_2_(thf)_2_] (**1**). All metal and oxygen atoms shown as 30% shaded ellipsoids.

A closer look for intramolecular secondary interactions in **1** revealed only F···H_THF_ between the CF_3_ group of L and a hydrogen of a thf. A comparison to structurally similar MgL_2_(dme)_2_ ^76^ showed similar intramolecular secondary interactions (Table S4, Figure S2). Expanding our search to intermolecular interactions between neighboring monomers in the extended solid state, we observed a significant number of π···H and F···H interactions for both **1** and MgL_2_(dme)_2_ (Table S5, Figures S3–S4).

Access to the heavier congeners proved more challenging, with no single synthetic route yielding all of them. Rather, direct metalation and protonolysis routes were utilized under a variety of conditions (Scheme 1). In direct metalation, metal filings are activated by condensing dry liquid ammonia at a low temperature. This is done in the presence of the alcohol HOC­(Ph)­(CF_3_)_2_ and solvent such as THF, forming a bright blue solution. In this way, we set out to synthesize the full series of Ca, Sr, and Ba compounds. While a barium product could not be isolated in crystalline form after multiple attempts, monomeric complexes [*trans*-CaL_2_(thf)_4_] **(2)** and [*cis*-SrL_2_(thf)_4_] **(3)** were readily recrystallized from the filtered reaction mixture layered with hexanes.

Compound **2** (Figure 4) is monomeric, adopting an octahedral *trans*-geometry about the Ca center, typical of other calcium coordination compounds, although *cis*-geometries are not uncommon, with differences attributed to crystal packing effects and polarization.^13,95^ The symmetry-generated O–Ca–O axis is 180.0°, with the ligands in the axial positions (Ca–O 2.2054(9) Å). The four neutral thf donors occupy the equatorial positions with equatorial angles at 89.12(4)° and 90.88(4)° and longer Ca–O distances (2.3939(9) and 2.398(1) Å) compared to the alkoxide ligand. No Ca···F interactions were found below the cutoff (2.99 Å), with the *trans*-geometry keeping the closest M–F contact (4.38 Å) far outside of any interaction. The presence of four neutral thf donors sufficiently completes the coordinative saturation requirements of the calcium ion. Similarities are found when comparing **2** with the previously reported [*cis*-Ca­(L^3^)_2_(thf)_4_], (L^3^, Figure 1), as both are monomeric with their coordination sphere saturated by four thf molecules and no Ca···F interactions.^13^ Similarly, in [*cis*-Ca­(L^3^)_2_(dme)_2_] and [*trans*-Ca­(L^3^)_2_(diglyme)_2_], the presence of neutral donors dme and diglyme also supersede the need for Ca···F interactions.^13^ In contrast, when the neutral donor is instead present as an ancillary arm on the anionic fluoroalkoxide ligand (see Figure S1), Ca···F interactions (ranging from 2.605(1) to 3.119(2) Å) were reported in every compound of the type [Ca­(L*)­N­(SiMe_2_H)_2_]_2_ and [Ca­(L**)­N­(SiMe_3_)_2_]_2_ (where L* = L^6^, L^9–12^ L^14^, and L** = L^6^, L^9^, all L’s defined in Figure S1 with M···F interactions listed in Table S3)^57–60^ as well as three trimeric complexes with similar ligands.^60,61^ The authors use somewhat longer cutoffs for Ca···F interactions than the 2.99 Å implemented here in Table S1, but this does not diminish their significance in these dimeric and trimeric compounds of fluoroalkoxides with an ancillary arm. Only one such calcium compound, [CaL^8^], reports no Ca···F interactions.^8^

**Figure 4:**
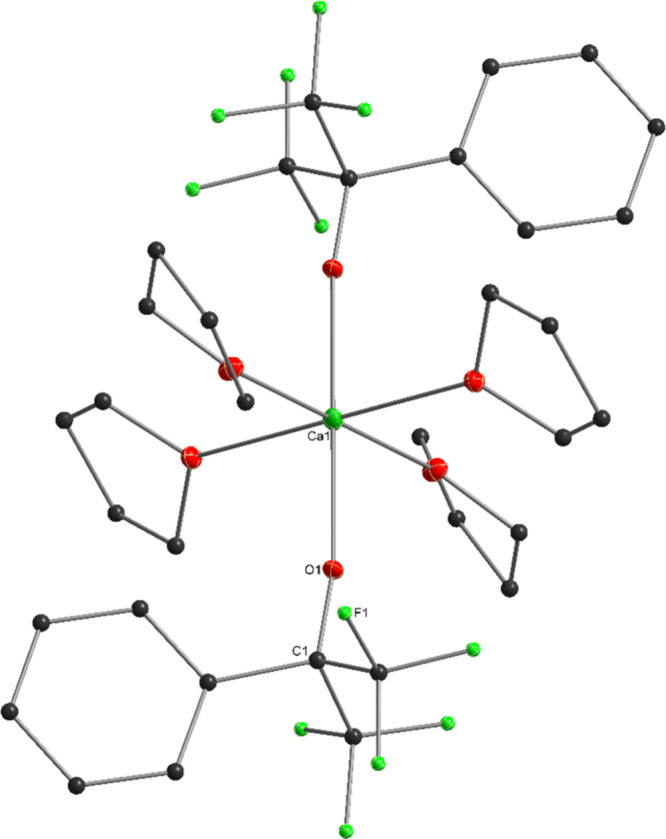
Crystal structure diagram for [*trans*-Ca­(OC­(Ph)­(CF_3_)_2_)_2_(thf)_4_] (**2**). All metal and oxygen atoms shown as 30% shaded ellipsoids.

The strontium compound [*cis*-SrL_2_(thf)_4_] (**3**) (Figure 5), crystallizes with two unique but structurally similar molecules per unit cell. Similar to the magnesium and calcium thf adducts, (**3**) is also monomeric, but adopts a distorted octahedral *cis*-geometry about the Sr center. The geometry is similar to that found in [*cis*-Sr­(L^3^)­(thf)_4_] and [*cis*-Sr­(L^3^)­(dme)_2_].^13^ The angle between the two alkoxide ligands is larger than the expected 90° in each of the two unique molecules (O1–Sr1–O2 104.7(1)°, O7–Sr2–O8 103.6(1)°). As a result, other angles are compressed. The O–Sr–O angle between one of the ligands and the thf donor opposite it ranges from 159.2(1)°-164.3(1)°. The angles between two opposite thf donors are 161.2(1)° and 168.4(1)°. Sr–O alkoxide bond lengths range from 2.305(3) to 2.314(3) Å, shorter than Sr–O bonds to the neutral donors (2.540(3)-2.603(3) Å). The shortest Sr···F interaction is 3.96 Å, well outside of the cutoff of 3.18 Å in Table S1. It appears that the presence of the four thf molecules satisfies the coordination sphere of the Sr ion. Structural details for the perfluorinated ligand strontium complexes [*cis*-Sr­(L^3^)­(thf)_4_], [*cis*-Sr­(L^3^)­(dme)_2_], and [*trans*-Sr­(L^3^)­(diglyme)_2_] also do not show Sr···F interactions.^13^ As was mentioned before, the use of ligands with separate neutral donors results in a limited degree of Sr···F interactions (Table S2). On the other hand, as was also mentioned before, the use of fluoroalkoxide ligands with ancillary neutral donor arms are often reported with Sr···F interactions, for example [Sr­(L^5^)_2_]_2_,^14^ [SrL^7^(H_2_O)], [SrL^8^],^8^ [Sr­(L*)­N­(SiMe_2_H)_2_]_2_ (where * = 10, 11-H_2_, 12)^58–60^ report Sr···F interactions ranging from 2.806(3) - 3.172(1) Å. However, three compounds of this type, Sr­(L^4^)_2_,^14^ Sr­(L^6^)_2_,^56^ [SrL^7^(thf)],^8^ report no Sr···F interactions (Table S3).

**Figure 5:**
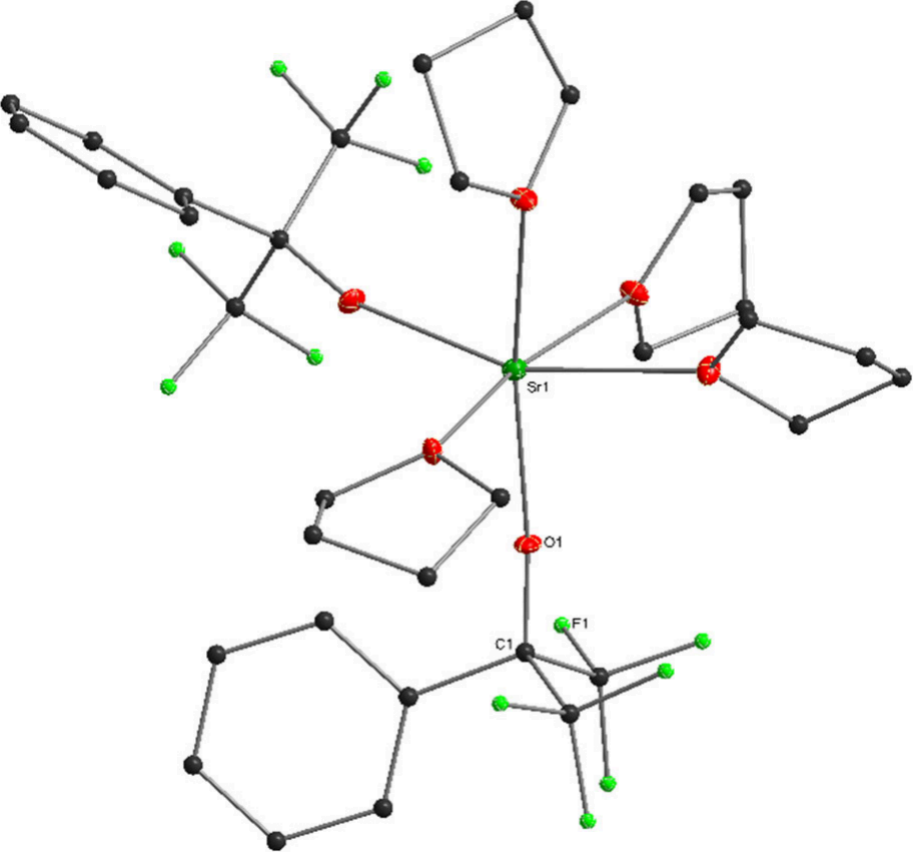
Crystal structure diagram for [*cis*-Sr­(OC­(Ph)­(CF_3_)_2_)_2_(thf)_4_] (**3**). All metal and oxygen atoms shown as 30% shaded ellipsoids.

While no M···F interactions were observed in either **2** or **3**, intramolecular secondary interactions π····H and F···H were observed between the ligand and the thf donors (Table S6, Figure S5). In addition, extensive intermolecular π····H and F···H interactions were observed for both **2** and **3** (Table S7, Figures S6–S7).

To assess the role of the neutral donor, compound **3** was dissolved in toluene to obtain a compound with fewer thf donors, yielding [Sr_2_(μ^2^-L)_3_(L)­(thf)_3_] (**4**) (Figure 6). The asymmetric dimeric compound **4** has one 4 + 6-coordinate strontium (Sr1) with three μ^2^-bridging alkoxide ligands (Sr1–O 2.542(2)-2.558(2) Å) and one terminal alkoxide ligand (Sr1–O 2.270(2) Å), resulting in a formal coordination number of four. The terminal alkoxide has the widest O1–Sr1–O angles (134.10(7)-137.54(7)°) and the bridging alkoxide oxygens have the most compressed O–Sr1–O angles (73.23(6)-77.98(6)°). The three bridging ligands are arranged so that all CF_3_ groups are oriented toward Sr1, enabling six Sr···F interactions (2.654(2)-2.909(2) Å). The six Sr···F interactions occur in a nearly planar arrangement around the Sr1 atom, similar to the effect of a crown-ether. The other strontium (Sr2) has a six-coordinate distorted octahedral geometry similar to the strontium in **3**, with O–Sr2–O angles 169.16(7)-171.64(7)° (for those expected to be close to 180°) and 76.82(7)-110.94(7)° (for those expected to be close to 90°). In addition to the three bridging alkoxide ligands (Sr2–O 2.437(2)-2.456(2) Å), Sr2 is coordinated by three thf donors (Sr2–O 2.555(2)-2.597(2) Å) and, in analogy with compound **3**, does not engage in Sr···F interactions. The asymmetry of the bridging ^–^OC­(Ph)­(CF_3_)_2_ ligands prevents Sr2 from having close contacts with any CF_3_ groups. Compound **4** is structurally very similar to the heterobimetallic perfluorinated alkoxide complexes [AM­(L^3^)_3_(thf)_4_] reported previously,^49^ where A= Na or K and M = Ba, Sr, or Eu. The difference in valency accounts for a fourth alkoxide ligand terminally bound to Sr1 in **4**, whereas in the heterobimetallic compounds, that terminal position is instead occupied by a fourth, neutral thf. The triply bridging motif of **4** is identical to the facially fused octahedral metal centers in the [AM­(L^3^)_3_(thf)_4_] compounds.^49^

**Figure 6:**
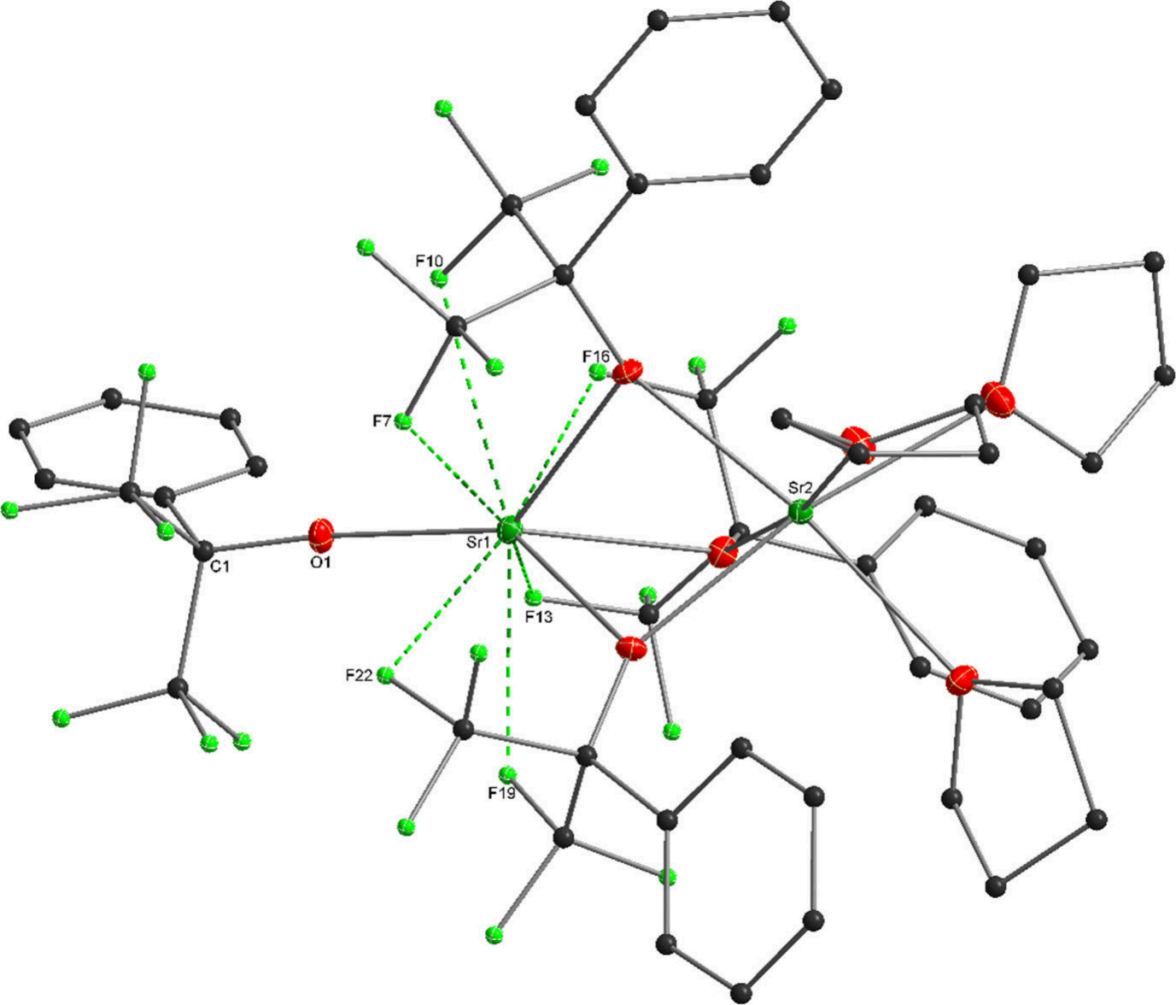
Crystal structure diagram for [Sr_2_(μ^2^-OC­(Ph)­(CF_3_)_2_)_3_(OC­(Ph)­(CF_3_)_2_)­(thf)_3_] (**4**). All metal and oxygen atoms shown as 30% shaded ellipsoids. Sr···F interactions highlighted by dashed bonds.

Compound **4** demonstrates the potential impact of the donor in stabilizing the metal centers. In the presence of reduced donor, there are a larger number of noncovalent interactions within the cutoff. Accordingly, attempts to examine the capacity of noncovalent interactions to stabilize the metal centers led to direct metalation reactions conducted in the absence of Lewis donors (Scheme 1). After unsuccessful attempts to obtain a series of donor-free Ca, Sr, and Ba target compounds from direct metalation reactions conducted in toluene, the residues from the Sr and Ba reactions were recrystallized from diethyl ether to afford the trimeric [Sr_3_(μ^2^-L)_4_(L)_2_(OEt_2_)_2_] (**5**) and oligomeric [Ba­(μ^2^-L)_2_]_n_· $\frac{1}{4}$ ­(OEt_2_) (**6**). It is important to note that direct metalation for calcium is not known to be successful in toluene,^96^ and ether solvents can be important in the stability of reaction intermediates,^97^ as shown previously.

Trimeric compound **5** (Figure 7) in essence contains two different types of Sr centers: the central Sr2 and the terminal Sr1 and Sr3. The central Sr2 (CN = 4 + 7) is connected to the two terminal strontium atoms by four bridging alkoxide ligands (Sr2–O 2.464(3)-2.549(3) Å) for a formal coordination number of 4, in addition to seven Sr···F interactions (2.75–3.11 Å) with CF_3_ groups of the bridging alkoxides. The O–Sr2–O angles on the central strontium illustrate the distortion (angles range from 73.63(9) to 158.41(9)°). The planes formed on either side of Sr2 (Sr2–O–Sr1–O) and (Sr2–O–Sr3–O) have an angle between them of 71.08°. The terminal strontium atoms Sr1 (CN = 4 + 2) and Sr3 (CN = 4 + 3) differ from each other by a Sr···π interaction, but all other parameters are similar. Each have a formal coordination number of four from the two bridging alkoxide ligands (Sr1–O 2.439(3)-2.478(3) Å, Sr3–O 2.432(3)-2.461(3)­Å), one terminal alkoxide ligand (Sr1–O1 2.264(3), Sr3–O7 2.258(3) Å), and one diethyl ether donor (Sr1–O8 2.561(3), Sr3–O9 2.521(4) Å). Each also has short contacts with fluorine atoms and phenyl groups. Sr1 has a Sr···F interaction (3.07 Å) and a Sr···π interaction (3.39 Å), with two different bridging alkoxides. Sr3 has a Sr···F interaction (3.12 Å) and a Sr···π interaction (3.42 Å) with one bridging alkoxide, and a Sr···π interaction (3.27 Å) with the other. Based on these observations in the solid-state, the secondary interactions of the ^–^OC­(Ph)­(CF_3_)_2_ ligand appear to enhance its bridging capabilities. For example, one of the bridging alkoxides between Sr2 and Sr3 engages in μ^2^-η^3^:η^3^-coordination, believed to be the first of its kind for a fluorinated alkoxide. This is the first reported trimeric strontium complex with fluoroalkoxide ligands (as mentioned earlier, only monomeric and dimeric strontium examples are known).

**Figure 7:**
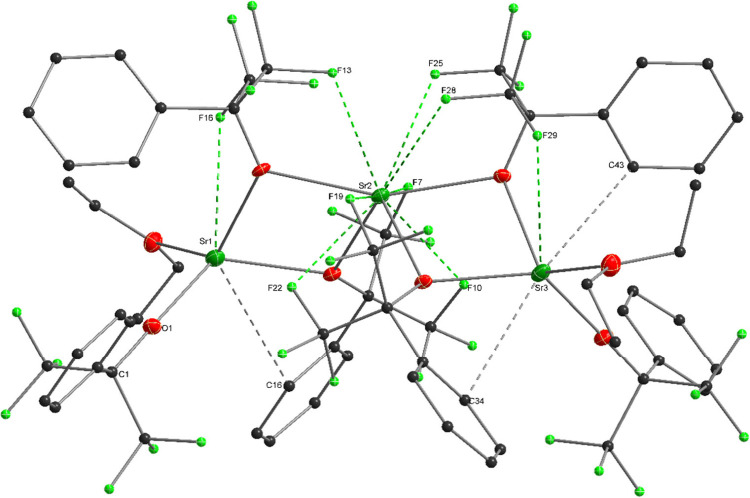
Crystal structure diagram for [Sr_3_(μ^2^-(OC­(Ph)­(CF_3_)_2_)_4_(OC­(Ph)­(CF_3_)_2_)_2_(OEt_2_)_2_] (**5**). All metal and oxygen atoms shown as 30% shaded ellipsoids. Sr···F and Sr···π interactions highlighted by dashed bonds.

Also synthesized by direct metalation in toluene and recrystallized from diethyl ether is the coordination polymer, Compound **6** (Figure 8a). The formula is [Ba­(μ^2^-L)_2_]_n_· $\frac{1}{4}$ ­(OEt_2_) due to diethyl ether being present as a lattice solvent in a ratio of one diethyl ether for every four formula units of BaL_2_. Figure 8b shows the arrangement of diethyl ether solvent in channels formed by the one-dimensional coordination polymer of BaL_2_ repeating units. There are no close contacts between the diethyl ether and any of the atoms of the coordination polymer. The solvent-free coordination polymer, [Ba­(L)_2_]_n_ **(7)** (Figure 9a-b), was obtained by a different synthetic route. Protonolysis has been established by our group^13^ and others^8^ as a viable route to alkaline earth alkoxides. However, here we chose to utilize diethyl ether as the solvent to avoid coordination of THF in the final product. Also, the reactions were carried out in sealed tubes to avoid incorporation of adventitious moisture and promote crystal growth in a controlled environment. While such reactions were carried out using [M­(N­(SiMe_3_)_2_)_2_(thf)_2_], where M = Ca, Sr, and Ba, only the reaction of the barium amide with the alcohol yielded crystalline product **7**. The coordination polymer of **6** and **7** are structurally similar, despite being synthesized *via* different routes, and therefore are described together.

**Figure 8:**
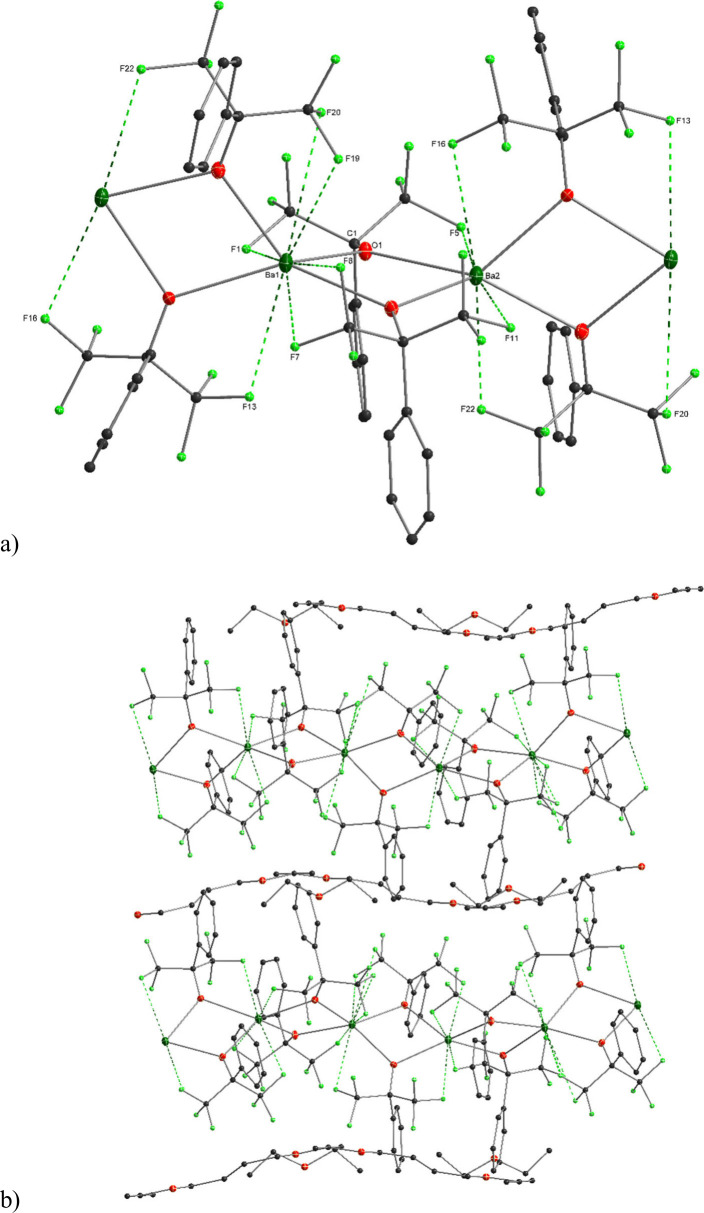
Crystal structure diagram for [Ba­(μ^2^-OC­(Ph)­(CF_3_)_2_)_2_]_*n*_ **·** $\frac{1}{4}$ ­(OEt_2_) (**6**). (a) Truncated view of one polymeric chain along *a*-axis. (b) View along the *a*-axis of two neighboring polymeric chains illustrating the location of the lattice diethyl ether molecules. All metal and oxygen atoms shown as 30% shaded ellipsoids. Ba···F interactions highlighted by dashed bonds.

**Figure 9:**
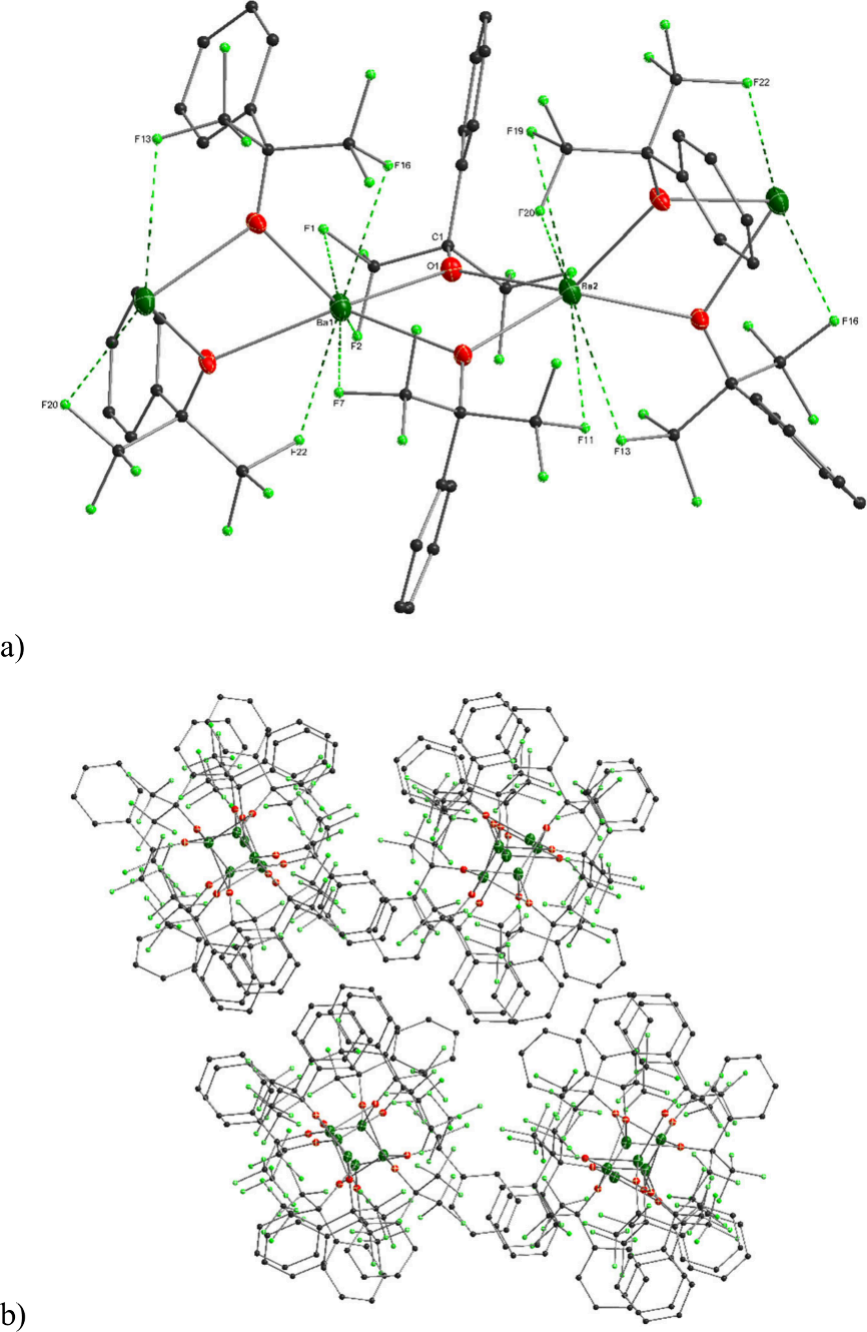
Crystal structure diagram for [Ba­(μ^2^-OC­(Ph)­(CF_3_)_2_)_2_]_*n*_ (**7**). (a) Truncated view of one polymeric chain along the *a*-axis. (b) View along the *b*-axis of four neighboring polymeric chains illustrating the absence of diethyl ether in the channels between them, in contrast to **6**. All metal and oxygen atoms shown as 30% shaded ellipsoids. Ba···F interactions highlighted by dashed bonds.

In both **6** and **7**, neighboring barium atoms are bridged by two alkoxides (Ba–O 2.579(3)-2.652(3) Å **6** and 2.658–2.716 Å **7**) to form a one-dimensional coordination polymer. The bridging alkoxides are oriented symmetrically between two metal centers, unlike in **4** and **5**, allowing Ba···F interactions (Ba···F 2.96–3.32 Å **6**, 2.95–3.31 Å **7**) with each barium. The barium atoms collectively range from 7-coordinate (CN = 4 + 3) to 10-coordinate (CN = 4 + 6), depending on the number of Ba···F interactions (Table S2). The phenyl rings are therefore roughly perpendicular to the propagation of the metal–ligand chain, and as such, no Ba···π interactions are observed. The planes formed on either side of the barium atoms, (Ba1–O1–Ba2–O2) and (Ba1–O3–Ba2–O4), have an angle between them of 86.52° (**6**) and 84.26° (**7**). The O–Ba–O angles range from 72.9(1) to 137.5(1)° (**6**) and (69.8(2) to 151.5(2)°) (**7**). One notable feature of **6** and **7** is the absence of coordinated neutral donor molecules, setting them apart from previously reported barium fluoroalkoxide complexes, including monomeric ([*trans*-Ba­(L^3^)_2_(dme)_3_], [*trans*-Ba­(L^3^)_2_(diglyme)_2_],^13^ Ba­(L^4^)_2_,^14^ Ba­(L^6^)_2_,^56^ Ba­(L^8^),^8^), dimeric ([Ba­(L^5^)_2_]_2_,^14^ [Ba­(L^7^)]_2_,^8^) and a hydroxide cluster, [Ba_5_(OH)­(L^1^)_9_(thf)_4_(H_2_O)·THF].^39^ Also structurally similar is the [Ba­(hfa)_2_(OH_2_)] (hfa= 1,1,1,5,5,5-hexafluoropentane-2,4-dione) complex.^98^ The absence of coordinated neutral donors in **6** and **7** leads to increased nuclearity and an increased propensity for Ba···F interactions. Secondary interactions, such as Ba···F, are more often observed in barium complexes due to the large coordination sphere of the Ba^2+^ ion. Even in the presence of neutral donors, Ba···F interactions were reported in these related complexes: [Ba_5_(OH)­(L^1^)_9_(thf)_4_(H_2_O)·THF] (2.99–3.31 Å), [Ba­(L^5^)_2_]_2_ (3.21–3.36 Å), Ba­(L^6^)_2_ (3.13–3.21 Å), [Ba­(L^7^)]_2_ (3.00 Å), and Ba­(L^8^) (3.14 Å).

### 3.2 Theoretical Studies, NMR, and Thermal Stability

#### 3.2.1 Theoretical Studies

Compounds **1**, **2**, **3** and **4** are model systems for exploring the crystallographic influences on observed configurations in comparison to the likely preferred configurations in the gas phase and solution with computational studies. Results are discussed in general terms here, details are in the Supporting Information. Theoretical investigations of **1**-**4** were prioritized because these compounds had more potential for applications as CVD precursors, but we are actively investigating the gas phase conformations of **5**-**7** to better understand the stabilizing forces at play in these large structures.

For the magnesium monomer **1**, the family of predicted gas-phase configurations (Table S8, Figure S8) all lie at least 14 kJ/mol lower in energy than the configuration based on experimental X-ray data. Interestingly, none of these geometries contain M···F interactions (all M···F distances are >3.10 Å). The most stable predicted gas-phase configuration (Figure 10, 1a, two views) is nearly C_2_-symmetric with each phenyl ring facing a hydrogen on a thf donor, resulting in two significant π···H_THF_ intramolecular interactions within established values (Table S1), as well as ten F···H interactions.^99,100^ The second and third most stable motifs (Figure 10, 1c) show the phenyl rings facing each other, resulting in a *π*_*centroid*_•••*π*_*centroid*_ distance of 3.84 Å, within cutoff values (Table S1), as well as several F···H interactions. The configurations most similar to the crystal structure were identified as higher energy local minima (Figure 10, **crystal**), but they do not include either of the significant features of **1a** or **1c**: no π···H_THF_ or anything close to π-π stacking (the *π*_*centroid*_•••*π*_*centroid*_ distance of 7.26 Å is well outside the accepted range). They do include several F···H_THF_ interactions. Factors contributing to stability in the solid state appear to be the π···H and F···H intermolecular interactions between ligand L and neighboring molecules discussed earlier (Table S5 and Figures S3–S4). The predicted gas-phase configurations reveal the extent to which factors *other than* M···F contacts influence the preferred geometries of **1**.

**Figure 10:**
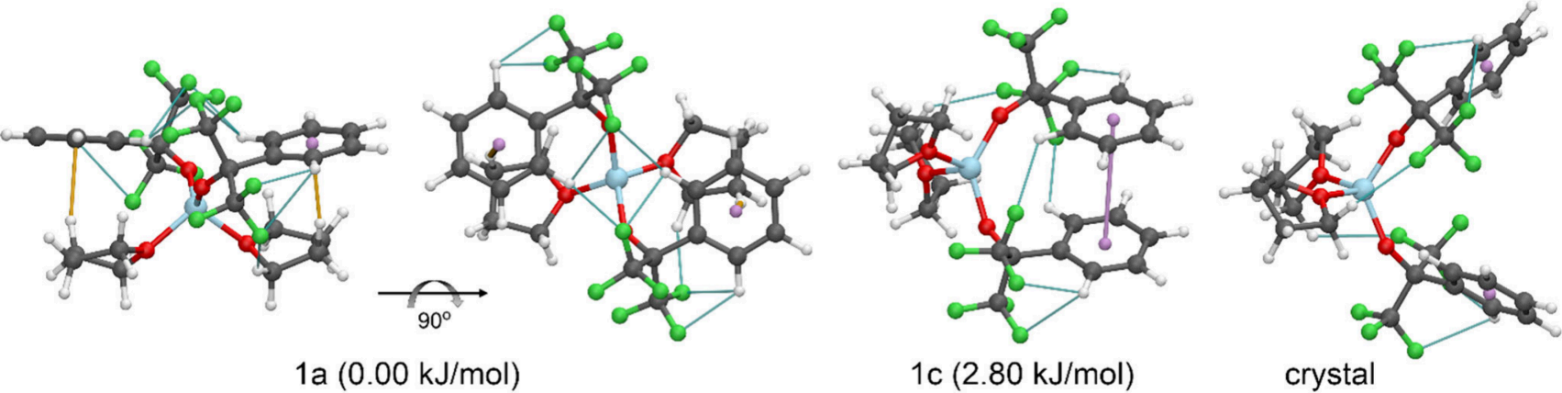
The most-stable gas-phase configuration (**1a**, two views shown) exhibits π···H_THF_ interactions (shown in gold) and F···H interactions (shown in green). The next most stable configurations (**1c**, shown as representative) exhibit a significant π···π interaction (shown in purple) and F···H interactions. In the higher-energy configurations most similar to the crystal structure (**crystal**), there is no π···π interaction, only several F···H interactions.

Octahedral **2** and **3** were modeled to investigate the preference of having the L ligands in either *cis*- or *trans*-configurations with the choice of Ca^2+^ or Sr^2+^ metal center, resulting in four sets of configuration geometries: *trans*-Ca (**2**), *trans*-Sr, *cis*-Ca, and *cis*-Sr (**3**). This assessment began with CREST-XTB-GFN2 conformational sampling of **2** (“*trans*-Ca”, Figure 11, left) and **3** (“*cis*-Sr”, Figure 11, right), followed by swapping of the metal centers to produce **2**-Sr (“*trans*-Sr”) and **3**-Ca (“*cis*-Ca”).

**Figure 11:**
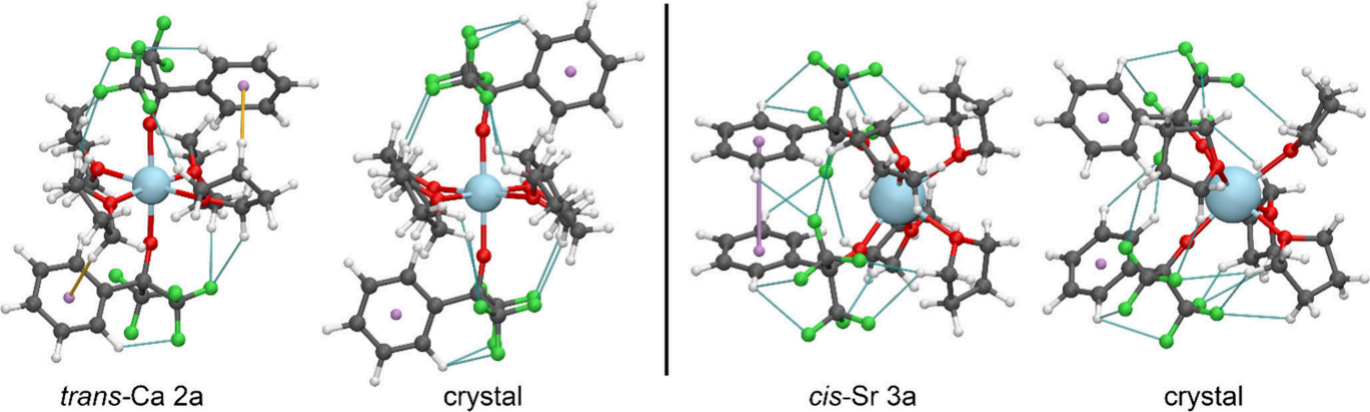
Representative configurations of computational outcomes investigating **2** and **3**. *Trans* **-Ca 2a** is the lowest energy configuration for a *trans*-ligand arrangement around Ca^2+^; it exhibits π···H_THF_ interactions (shown in gold) and F···H interactions (shown in green). The configuration most similar to the **crystal** of **2** (“crystal”) has fully extended ligands, L, with only F···H interactions (green), and is 4.82 kJ/mol higher in energy than the predicted global minimum to its left. **Cis-Sr 3a** is the lowest energy configuration for the *cis*-ligand arrangement around Sr^2+^; it exhibits significant π···π interaction (shown in purple), as well as 14 F···H interactions. The **crystal** configuration of **3** (“crystal”) is not predicted to be a minimum energy geometry from the calculations, favoring formation of the π···π interaction.

The same DFT optimization workflow and uniqueness criteria used for **1** produced 16 *trans*-Ca (Table S9), and 21 *trans*-Sr configuration geometries (Table S10), while only producing four *cis*-Ca and two *cis*-Sr configurations (Table S11). The larger number of *trans*-Ca and *trans*-Sr gas-phase configurations is due to their multiple and varied networks of F···H_Ph_ and F···H_THF_ contacts. The smaller number of *cis*-configurations is attributed entirely to the formation of a stabilizing π-stacking interaction between the two alkoxide ligands, L, and the overall reduced number of thf arrangements that maximize favorable F···H_THF_ interactions due to the π-stacking constraint of the larger ligands. There are no close Ca···F or Sr···F contacts in any of the optimized geometries. The shortest M···F distance among the entire series is 3.90 Å, well above the cutoff (Table S1).

The lowest energy *trans*-configuration (*trans* **-Ca 2a**) is mostly similar to the **crystal** configuration, except that its phenyl rings rotate to engage in a π···H_THF_ interaction that does not appear in the **crystal** configuration, which instead has its alkoxide ligands, L, splayed out with a long π···π distance (10.49 Å) and C_i_ geometry. Looking among the family of *trans*-configurations (Table S9) for those that might resemble the C_i_ geometry of **2**, we find five with long π···π distances (10.4–10.61 Å), and three of these lie within 10 kJ/mol of the predicted global minimum. The same trends are observed in the *trans*-Sr series (Table S10), which share similar relative energies across the series, having the longest π···π distances among the higher-energy configurations of the series. The observed C_i_ geometry of **2** may be due in part to the ordering of thf molecules, as the calculated geometries include varied arrangements of thf molecules across minima, resulting in several geometries that reproduce the C_i_-like character in the crystal cell.

The lowest energy *cis*-configuration (**cis-Sr 3a**) with the π-stacking is not the one observed in the crystal. In the **crystal**, the two phenyl ring centroids have been offset by 1.4 Å and do not engage in π···π interactions, likely due to stabilizations from π···H_THF_ and F···H_THF_ intra- and intermolecular interactions discussed earlier (Tables S6–7, Figures S5–7). It is interesting to note that the relative energies of the predicted gas-phase *cis*-Ca configurations trend, albeit with a small sample size, almost linearly with π···π centroid distance (Table S11), suggesting that π-stacking in *cis*-Ca is not competitive against the number of predicted F···H_THF_ interactions formed from having the alkoxide ligands, L, in a *trans*-geometry.

Relative energy comparisons do, to a limited extent, reproduce the observation of the Ca^2+^ preferring a *trans*-L arrangement and the Sr^2+^ preferring a *cis*-configuration for this combination of two L ligands and four coordinated thf molecules. For *trans*-Ca and *cis*-Ca, the relative energies of the *cis*-configurations lie in the midrange of the sub-10 kJ/mol *trans*-configurations, with five of the *trans*-L configurations (those consistent with the crystal geometry of **2**) still lower in energy. In the cases of *trans*-Sr and *cis*-Sr, the most stable geometry contains π-stacking and the closest-lying *trans*-configuration is 3.76 kJ/mol higher in energy, yielding some predicted preference for the intramolecular stabilization by this stacking at the lc-ωPBE­(GD3-BJ)/Def2-TZVP level of theory.

Conformations of the dimer **4** were generated by simple rotations of all three bridging-alkoxide ligands, L, to explore how the orientations of these three, and the pair of M···F interactions provided by each in the crystal cell, might vary across other accessible minima. Five calculated geometries (Figure 12, **4a**-**e**), including the crystallographic configuration of the three L ligands (**4a**), were obtained as characterized minima. Three of these five (**4b**, **d**, and **e**) are predicted to be more stable than the crystal-like geometry. Configuration **4d**, which flips one of the equatorial L, yields one longer-range π···H_Ph_ interaction to the axial L and reduces the distances of several π···H_THF_ interactions on the other side, resulting in a geometry 6.13 kJ/mol lower in energy than the crystal and containing two fewer Sr···F contacts. The most stable geometry obtained (**4e**, 7.11 kJ/mol more stable than the crystal configuration) flips two L to produce a pair of π···H_Ph_ interactions involving the original axial L, while significantly increasing the number of F···H_THF_ contacts at the overall expense of three of the original six Sr···F contacts.

**Figure 12:**
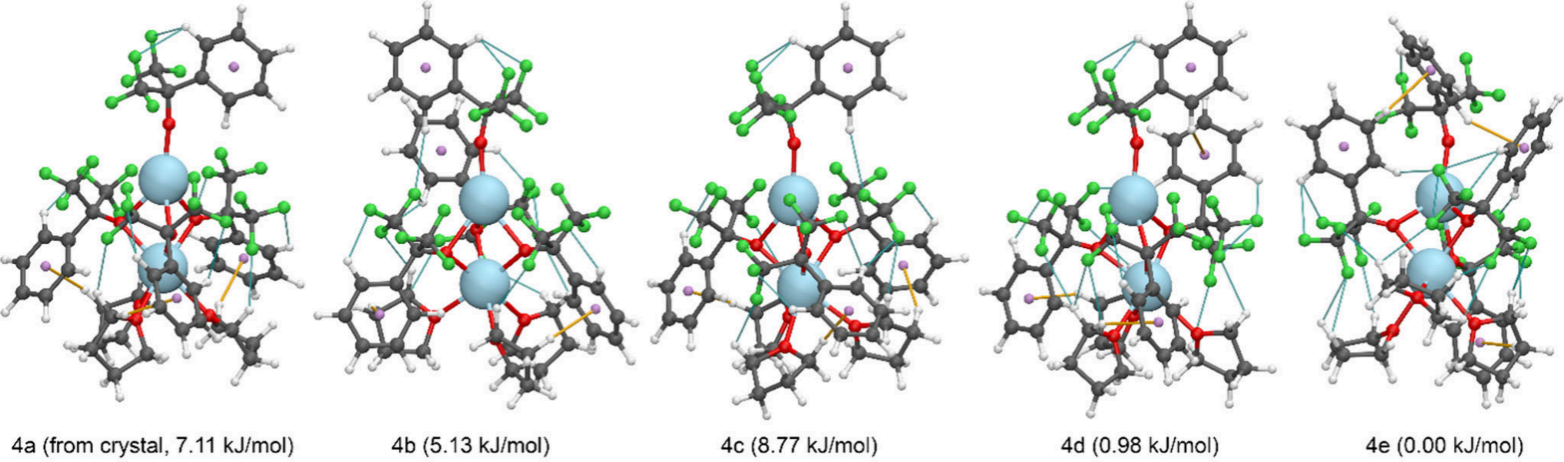
Five calculated representations of the dimer **4**, in which the three bridging-alkoxide ligands, L, were rotated to explore the effect on M···F interactions. In the crystal-like configuration (**4a**) all three bridging-alkoxide ligands are oriented in the same direction, resulting in six Sr···F interactions. In **4b**–**e**, different combinations of the bridging L’s are flipped, diminishing the number of Sr···F contacts and, in three cases, *lowering* the energy relative to **4a**.

#### 3.2.2 NMR Studies

The ^19^F NMR spectra were investigated for evidence of peak splitting to see if fluorine atoms in the CF_3_ groups were in unique chemical environments in solution, potentially caused by limited rotation of the C–CF_3_ bond due to noncovalent interactions such as M···F, the hindered rotation of the phenyl ring due to π···H, or the locking of fluorine atoms in F···H_THF_ interactions.^101,102^ A single peak was found for the CF_3_ groups in compounds **1–4** which correlates not only with the solid state and theoretical findings that **1**-**3** do not have any M···F interactions, but that no strong interactions exist to inhibit rotation in a manner that would differentiate the fluorine atoms in any of the existing CF_3_ groups. The M···F interactions observed in the solid-state of **4** are not observed in solution. This behavior was previously reported in the alkaline earth metal complexes of the perfluorinated alkoxide ligand, L^3^, [Ae­(L^3^)_2_(donor)_*x*_] (Ae = Mg, Ca, Sr, and Ba; donors = thf, dme, and diglyme)^13^ and [AM­(L^3^)_3_(coligand)_*x*_]_4_ (where A = Na, K; M = Mg, Sr, Ba, Eu; coligand = thf, toluene),^49^ which exhibit M···F interactions in the solid state but give a single peak in the ^19^F NMR spectra. The ^1^H NMR spectra of **1**-**4** exhibit chemical shifts and coupling constants for the phenyl groups in expected ranges (7–8 ppm, 6–7 Hz), further suggesting the lack of noncovalent π···H or F···H_Ph_ interactions in solution.

The asymmetry of compound **5** creates unique environments for enantiotopic CF_3_ groups, resulting in splitting of the ^19^F signals. Compound **6** displays multiplets and indistinguishable peaks, but compound **7** has two signals (one broad) with a difference of ∼ 1.8 ppm. Their solid-state structures show nine (**6**) and 17 (**7**) close Ba···F contacts. It is possible that Ba···F interactions are present in solution, as they are in the solid state, however, it requires further in-depth analysis. Mg···F interactions have been found to shift ^19^F NMR peaks ∼ 1.2–1.9 ppm, depending on concentration.^103^ Due to solubility issues, concentration studies were not possible. While M···F interactions are often credited with stabilizing roles, our theoretical studies suggest that F···H_THF_ interactions play a more significant role in limiting rotation in the gas phase than M···F interactions. The implications of these complicating factors for solution behavior have motivated us to explore in future work the role of noncovalent interactions in these complexes in solution with variable solvent and temperature effects.

#### 3.2.3 Thermal Gravimetric Analysis

Representative thermogravimetric analysis was performed on **2** and **3** (Figures S37, S38). Due to the similar formulas of **2** and **3** (ML_2_(thf)_4_), one would expect to see similar behavior in the TGA for both compounds, but they decompose quite differently. The results for compound **2** show two sharp decreases in weight loss, the first one beginning at 38 °C, indicating the probable loss of coordinated thf, although the percentages do not correspond to a well-defined residue. The second significant weight loss occurs at 266 °C with a remaining residue of 16.5 wt %, which is more than the expected 5% if only calcium metal were remaining. Compound **3** exhibits a gradual weight loss over an extended temperature range (25–313 °C), leaving residue of 30.8 wt %. The percentages for compound **3** also do not correspond to a well-defined residue. A strontium atom and one ligand would give 38.4% mass remaining and the strontium only would be 10.2%, and this weight loss falls in between these. Further heating of **3** to 800 °C only slightly reduces the remaining residue to 27.8 wt %. While the TGA analysis is performed under inert gas, loading the samples requires brief contact with air. Thus, using TGA for confirming bulk purity of these samples poses limitations. While the superior TGA performance of the homo- and heterometallic ^–^OC­(CF_3_)_3_ complexes might be attributed to their air-stable nature,^13,49^ there is value in knowing the impact of ligand modification on stability and volatility.

## 4 Discussion

It has been well established that the presence of fluorinated substituents creates the possibility for stabilizing M···F secondary interactions in alkaline earth metal fluoroalkoxides (see Table 1 and references therein). To identify the presence of intramolecular M···F interactions for compounds **1**-**7**, the distance between the metal cation and a separate covalently bound fluorine atom were measured using the solid-state crystallographic data. Since the metal is cationic, not a neutral atom, we added the van der Waals radii of the metal ions (Table 1, first column) as calculated by Merz, Li et al.,^26^ to the consistent van der Waals radius of fluorine as confirmed by Truhlar et al. (1.47 Å)^104^ to obtain the cutoff values used in this work (Table 1, second column). Below the cutoff value, the M···F interaction is considered significant in that it may provide stabilization of the metal cation.^77^ For example, Mg···F distances under 2.59 Å would be considered a noncovalent M···F interaction in this work. A summary of M···F interactions below the cutoff values observed in the crystallographic data for compounds **4**-**7** are presented in Table 1, third column. No M···F interactions below the cutoff values were observed for **1**-**3**.

**Table 1:** Cutoff Values Used for Establishing Secondary Noncovalent Interactions in Compounds **1**–**7**, M···F Intramolecular Interactions Observed in **4**–**7**, and Ranges of M···F Interactions Reported by Others for Alkaline Earth Metal Fluoroalkoxides^a^

	M···F interactions			
	van der Waals radii of metal ions^26^	Cutoff for M^2+^···F intramolecular interactions in this work^b^	Crystallographically observed M···F intramolecular interactions for compounds **4**–**7**	Crystallographically observed M···F interactions in alkaline earth metal fluoroalkoxides reported elsewhere
Mg^2+^	1.12	2.59		2.17—2.74 ^42,92–94^
Ca^2+^	1.52	2.99		2.61—3.12 ^8,13,57–61,77^
Sr^2+^	1.71	3.18	2.65—3.12	2.81—3.17 ^8,13,14,58–60,77^
Ba^2+^	1.91	3.38	2.90—3.31	2.99—3.36 ^8,14,39,56,77,105^

a Interatomic distances below the cutoff values listed in the second column are considered significant in this paper. All values given in Å. An expanded version of this table is found in Table S1.

b Sum of van der Waals radii of metal ion and fluorine (1.47 Å).^104^

A list of M···F interactions reported for alkaline earth metal fluoroalkoxides in other publications is summarized in Table 1, fourth column. Only those M···F interactions which were identified by the authors of those publications are included. The reported M···F interactions span a range of distances, and the smallest and largest distances reported in those papers are given in the table for reference. While M···F interactions are certainly known for a variety of alkaline earth metal complexes containing fluorinated ligands, the scope of this list includes only alkaline earth metal fluoroalkoxides similar to the ligand, L, used in this work. A detailed list of the reported M···F interactions in alkaline earth fluoroalkoxides from other publications is presented in Tables S2 and S3, SI.

In a similar manner, we also explored the stabilizing role of M···π secondary interactions in compounds **1**-**7**. The maximum distance considered to be a M···π interaction in this work is the sum of van der Waals radii of the metal ions given in Table 1, first column^26^ and the van der Waals radius of carbon (1.70 Å),^104^ resulting in M···π cutoff values of 2.82, 3.22, 3.41, and 3.61 Å for M = Mg, Ca, Sr, and Ba, respectively. The cutoff values for heavy alkaline earth metal M···π interactions are explored experimentally and through DFT calculations in our recent work on tetraarylborates.^106^ For π···π interactions, the cutoff of 3.9 Å is measured as a centroid-centroid distance regardless of displacement from the parallel stack of the phenyl rings for both X-ray data^107–110^ and computational analysis.^99,100^ For X-ray crystallographic data, the π···H_Ph_ interaction is the distance from the centroid of one phenyl ring to the carbon atom of the other, with a cutoff of 3.4 Å.^107–110^ For computational studies, the π···H_Ph_ interaction is a range of distances from each of the six carbon atoms of one phenyl to one hydrogen atom on the other phenyl, which also gives an indication of how centered it is over the ring, with a cutoff of 2.81–2.97 Å.^99,100^ Finally, the F···H cutoff is determined by the sum of the van der Waals radii of fluorine (see above) and hydrogen (1.10 ^104^ – 1.20 ^111^ Å) with a cutoff of 2.7 Å.^104,111–113^

The synthetic routes employed allowed for some control over the amount and type of neutral donor in the alkaline earth metal complexes of the partially fluorinated alkoxide ligand, ^–^OC­(CF_3_)_2_Ph, L, presented here. The ligand, L, was chosen for its capabilities to potentially engage in both M···F and M···π interactions, to expand upon our previous work with the perfluorinated alkoxide ligand, L^3^. Despite the incorporation of the phenyl group in L, the compounds presented here had almost no instances of M···π interactions in the solid state or obtained geometries from computational modeling. In fact, the monomers **1**, **2**, and **3** also had no M···F interactions in any form, in theory or experiment. Instead, we found that the presence of the phenyl ring in L provided a number of π···H and F···H_Ph_ intramolecular and intermolecular interactions, which were especially significant to the stability and configurations of the monomeric complexes. For example, in **1**, the lowest energy predicted gas-phase configuration is not observed in the solid state, which is instead stabilized with π···H and F···H interactions (Table S4–5, Figures S2–4). Also, the lowest energy configuration in the computational model of **3** displays π ···π stacking between the two alkoxide ligands in the *cis*-configuration, but in corresponding models of **2**, it is π···H_THF_ and F···H_THF_ interactions that win out over the π ···π stacking, resulting in the *trans*-configuration of the ligands.

The significance of these weak interactions is again illustrated comparing the crystallographic results of monomeric **3** and dimeric **4**. The reduced number of thf molecules per Sr atom in **4** (1.5 thf molecules) compared to **3** (four thf molecules) is compensated for by the appearance of six Sr···F interactions in **4**. However, the computational models of **4** show lower energy configurations have *fewer* Sr···F interactions and more F···H_THF_ and π···H_Ph_ interactions. It is more difficult to say with confidence that the Sr···F interactions are of value at all in the overall stability of the crystal geometry for **4**, or if crystal packing and intermolecular interactions are doing more to stabilize the observed arrangement and the close Sr···F contacts are simply a consequence of the net total of all other interactions in the solid imposing orientations on the L ligands that force placement of the CF_3_ groups.

In the cases of **1** and **4**, the differences between theory and experiment can be reasonably well reduced to the presence of the phenyl rings in these ligands and the stabilizing intramolecular calculated interactions versus the number of stabilizing intermolecular interactions observed in the solid. That said, the TGA results for **2** and **3** are in significant contrast to those reported for [Ae­(L^3^)_2_(donor)_*x*_] (Ae = Mg, Ca, Sr, and Ba; donors = thf, dme, and diglyme).^13^ Purely in terms of application, the combination of fluorination and ligand choice appear to play a significant role in the usability of the complex, with the inclusion of pendant groups capable of noncovalent interactions disfavoring their use in CVD based on the TGA data. The phenyl group is an excellent motif for future studies along the lines of improved CVD properties because of its ability to be highly modified by way of steric and electronic changes to its ring. Alternatively, improved properties for CVD applications may only come from removing the possibility of noncovalent interactions, more in line with the results reported earlier.^13^

As is expected and as we have extensively reported before,^20,23,32,62^ the general trend in **1**-**7** was observed that the larger metal size has a propensity for increased nuclearity. The role of neutral donors to limit the number of metal centers in a complex is most clearly observed in the Sr-complexes **3**, **4**, and **5** with decreasing donor:metal ratios of 4:1 (monomer), 1.5:1 (dimer), and 0.7:1 (trimer). The presence of close M···F distances in the multinuclear **4**-**7** solid-state X-ray crystallographic data might suggest the M···F interactions are stabilizing the metal centers in the increased absence of neutral donors (with no neutral donors coordinated to the metal in **6** and **7**). However, in light of our theoretical findings for **1**-**4** that show the predicted M···F interactions in the gas phase are weaker than F···H and π···H, the reasons for the observed solid-state structures may in fact be more complex than M···F stabilization alone. Indeed, reliance on solid-state interatomic distances to determine the importance of relatively weak interactions such as M···F, F···H and π···H, assuming that shorter-is-stronger, may lead to overemphasis of their importance. Not all interactions are favorable, and other more favorable interactions elsewhere in the solid state may force two atoms to be nearer than expected.

## Conclusions

The incorporation of phenyl into a fluorinated alkoxide ligand for the formation of alkaline earth metal complexes allowed us to explore the impact of secondary noncovalent interactions on the solid state and predicted gas-phase geometries of the resulting complexes. Comparison of the crystallographic geometries with those obtained from DFT calculations show a number of different noncovalent interactions contribute to the structure and stability of the compounds in the solid state and predicted gas-phase. We found that M···F contacts were not predicted to impart stability in the gas phase conformations of the presented monomeric and dimeric compounds. This work is continuing with detailed studies assessing the role of M···F and M···π interactions and their combinations in the presence or absence of other potentially stabilizing noncovalent interactions.

## Supplementary Material



## Notes

Tables listing M···F interactions in compounds reported elsewhere, images of intra- and intermolecular interactions in **1**–**3**, details of X-ray crystallography and computational studies, ^1^H, ^13^C, ^19^F NMR spectra, IR spectra, TGAs of **2** and **3**, and Cartesian coordinates (PDF)
