# Supplementary material for: Investigations of Intramolecular and Intermolecular Noncovalent Interactions in Alkaline Earth Metal Complexes of –OC(Ph)(CF3)2
Source: Inorg Chem. 2026 Jun 12;65(25):13932–48. doi: 10.1021/acs.inorgchem.6c01110 (PMC13321312; doi:10.1021/acs.inorgchem.6c01110)
Supplement: Supplementary file 1 [file ic6c01110_si_001.pdf]

Supporting Information

Investigations of intramolecular and intermolecular noncovalent interactions in alkaline earth metal complexes of $^{-}OC(Ph)(CF_3)_2$

Anna Y. O'Brien,[a,b*] *Yuriko Takahashi,*[b] *Miriam Gillett-Kunnath,*[b] *Damian G. Allis,*[b] *Ana Torvisco,*[c] *Karin Ruhlandt-Senge*[b,d*]

(a) Department of Chemistry, Le Moyne College, 1419 Salt Springs Rd, Syracuse, NY 13214

(b) Department of Chemistry, 1-014 Center for Science and Technology, Syracuse University, Syracuse, NY 13244, USA

(c) Institute of Inorganic Chemistry, Technical University of Graz, 8010 Graz, Austria

(d) Department for Physical and Environmental Sciences, University of Toronto, Scarborough, ON M1C 1A4, Canada

Correspondence: obrienay@lemoyne.edu

Contents

Supplemental Information for Introduction 4

Table S1. Detailed information to supplement **Table 1** regarding cutoff values used for establishing secondary noncovalent interactions in this paper and reported elsewhere. 4

Table S2. List of M···F interactions for L^1-L^3, L 5

$[Mg(OC(C_6F_5)_3)_2]_2$ 6

Figure S1. Structures of fluoroalkoxides used as ligands (L^4-L^{15}) in compounds 7

Table S3. List of M···F interactions for alkaline earth metal complexes of L^4-L^{15} 9

Supplemental Information for X-ray crystallography results and discussion 11

Table S4. Intramolecular distances for **1** and a related compound, $Mg(HFPP)_2(dme)_2$ 11

Figure S2. Images of intramolecular interactions in **1** and $Mg(HFPP)_2(dme)_2$ 11

Table S5. Intermolecular interactions in **1** and a related compound, $Mg(HFPP)_2(dme)_2$ 12

Figure S3. Images of π···H intermolecular interactions in **1** and $Mg(HFPP)_2(dme)_2$ 12

Figure S4. Images of F···H intermolecular interactions in **1** and $Mg(HFPP)_2(dme)_2$ 13

Table S6. Intramolecular interactions in **2** and **3** 14

Figure S5. Images of intramolecular interactions in **2** and **3** 14

Table S7. Intermolecular interactions in **2** and **3** 15

Figure S6. Images of π···H intermolecular interactions in **2** and **3** 15

Figure S7. Images of F···H intermolecular interactions in **2** and **3** 16

Supplemental Information for computational studies results and discussion 17

Table S8. Family of predicted gas-phase configurations of **1**. 17

Figure S8. Key structural motifs among the final configurations of **1** 18

Table S9. Family of predicted gas-phase *trans*-configurations of **2** with Ca 19
Table S10. Family of predicted gas-phase *trans*-configurations of **2** with Sr 20
Table S11. Families of predicted gas-phase *cis*-configurations of **3** with Ca and Sr 21
X-ray crystallographic data collection and refinement details 21
Computation software and other details 22
X-ray crystallographic data tables 23
Table S12. Crystallographic data and details of measurements for compounds **1-7** 23
Characterization data 24
Figure S9. ^{1}H NMR $[Mg(OC(CF_3)_2Ph)_2(thf)_2]$ (**1**). 24
Figure S10. ^{13}C NMR $[Mg(OC(CF_3)_2Ph)_2(thf)_2]$ (**1**). 25
Figure S11. ^{19}F NMR $[Mg(OC(CF_3)_2Ph)_2(thf)_2]$ (**1**). 26
Figure S12. IR $[Mg(OC(CF_3)_2Ph)_2(thf)_2]$ (**1**). 27
Figure S13. ^{1}H NMR [*trans*-$Ca(OC(CF_3)_2Ph)_2(thf)_4$] (**2**). 28
Figure S14. ^{13}C NMR [*trans*-$Ca(OC(CF_3)_2Ph)_2(thf)_4$] (**2**). 29
Figure S15. ^{19}F NMR [*trans*-$Ca(OC(CF_3)_2Ph)_2(thf)_4$] (**2**) 30
Figure S16. IR [*trans*-$Ca(OC(CF_3)_2Ph)_2(thf)_4$] (**2**). 31
Figure S17. ^{1}H NMR [*cis*-$Sr(OC(CF_3)_2Ph)_2(thf)_4$] (**3**). 32
Figure S18. ^{13}C NMR [*cis*-$Sr(OC(CF_3)_2Ph)_2(thf)_4$] (**3**). 33
Figure S19. ^{19}F NMR [*cis*-$Sr(OC(CF_3)_2Ph)_2(thf)_4$] (**3**). 34
Figure S20. IR [*cis*-$Sr(OC(CF_3)_2Ph)_2(thf)_4$] (**3**) 35
Figure S21. ^{1}H NMR $[Sr_2(\mu^2\text{-}OC(CF_3)_2Ph)_3(OC(CF_3)_2Ph)(thf)_3]$ (**4**). 36
Figure S22. ^{13}C NMR $[Sr_2(\mu^2\text{-}OC(CF_3)_2Ph)_3(OC(CF_3)_2Ph)(thf)_3]$ (**4**). 37
Figure S23. ^{19}F NMR $[Sr_2(\mu^2\text{-}OC(CF_3)_2Ph)_3(OC(CF_3)_2Ph)(thf)_3]$ (**4**). 38
Figure S24. IR $[Sr_2(\mu^2\text{-}OC(CF_3)_2Ph)_3(OC(CF_3)_2Ph)(thf)_3]$ (**4**). 39
Figure S25. ^{1}H NMR $[Sr_3(\mu^2\text{-}(OC(CF_3)_2Ph)_4(OC(CF_3)_2Ph)_2(OEt_2)_2]$ (**5**). 40
Figure S26. ^{13}C NMR $[Sr_3(\mu^2\text{-}(OC(CF_3)_2Ph)_4(OC(CF_3)_2Ph)_2(OEt_2)_2]$ (**5**). 41
Figure S27. ^{19}F NMR $[Sr_3(\mu^2\text{-}(OC(CF_3)_2Ph)_4(OC(CF_3)_2Ph)_2(OEt_2)_2]$ (**5**). 42
Figure S28. IR $[Sr_3(\mu^2\text{-}(OC(CF_3)_2Ph)_4(OC(CF_3)_2Ph)_2(OEt_2)_2]$ (**5**). 43
Figure S29. ^{1}H NMR $[Ba(\mu^2\text{-}OC(CF_3)_2Ph)_2]_n\cdot$**14**$(OEt_2)$ (**6**). 44
Figure S30. ^{13}C NMR $[Ba(\mu^2\text{-}OC(CF_3)_2Ph)_2]_n\cdot$**14**$(OEt_2)$ (**6**). 45
Figure S31. ^{19}F NMR $[Ba(\mu^2\text{-}OC(CF_3)_2Ph)_2]_n\cdot$**14**$(OEt_2)$ (**6**). 46
Figure S32. IR $[Ba(\mu^2\text{-}OC(CF_3)_2Ph)_2]_n\cdot$**14**$(OEt_2)$ (**6**). 47
Figure S33. ^{1}H NMR $[Ba(\mu^2\text{-}OC(CF_3)_2Ph)_2]_n$ (**7**). 48
Figure S34. ^{13}C NMR $[Ba(\mu^2\text{-}OC(CF_3)_2Ph)_2]_n$ (**7**). 49
Figure S35. ^{19}F NMR $[Ba(\mu^2\text{-}OC(CF_3)_2Ph)_2]_n$ (**7**). 50
Figure S36. IR $[Ba(\mu^2\text{-}OC(CF_3)_2Ph)_2]_n$ (**7**). 51
TGA data 52

Figure S37. Thermogravimetric plot of the remaining weight percent of [*trans*-$Ca(OC(CF_3)_2Ph)_2(thf)_4$] (**2**) 52
Figure S38. Thermogravimetric plot of the remaining weight percent of [*cis*-$Sr(OC(CF_3)_2Ph)_2(thf)_4$] (**3**) 52
Cartesian Coordinates. 53
Compounds 1-7. 53
Optimized geometry for 4a based on crystal geometry of 4. 119
Optimized geometry for 4b based on calculated minima. 121
Optimized geometry for 4c based on calculated minima. 123
Optimized geometry for 4d based on calculated minima. 125
Optimized geometry for 4e based on calculated minima. 127
References 128

Supplemental Information for Introduction

Table S1. Detailed information to supplement **Table 1** regarding cutoff values used for establishing secondary noncovalent interactions in this paper and reported elsewhere. Interatomic distances below the cutoff values listed here are considered significant in this paper.

	**M···F interactions**				**M···π interactions**
	**van der Waals radii of metal ions**[1] **$r_{ionic\text{-}vdW}$M**	**Cutoff for M^{+2}···F interactions used in this work**† **($r_{ionic\text{-}vdW}$M) + (r_{vdW}F)** **where r_{vdW}F = 1.47 Å**[2]	**Crystallographically observed M···F intramolecular interactions for compounds 4-7**	**Crystallographically observed M···F interactions in alkaline earth metal fluoroalkoxides reported elsewhere**	**Cutoff for M^{2+}···π interactions used in this paper**†† **($r_{ionic\text{-}vdW}$M) + (r_{vdW}F)** **where r_{vdW}C = 1.70 Å**[2]
Mg^{2+}	1.12	2.59	—	2.17—2.74[3–6]	2.82
Ca^{2+}	1.52	2.99	—	2.61—3.12[7–14]	3.22
Sr^{2+}	1.71	3.18	2.65—3.12	2.81—3.17[7,9–11,13–15]	3.41
Ba^{2+}	1.91	3.38	2.90—3.31	2.99—3.36[13–18]	3.61
**Cutoffs used for additional secondary interactions:** ††† π ···π (3.9 Å X-ray data[19–22] and computational analysis[23,24]) π ···H (3.4 Å X-ray data[19–22] and 2.81-2.97 Å computational analysis[23,24]) F···H (2.7 Å X-ray data[2,25–27] and computational analysis)					

† One means to assess the presence of M···F interactions is by measuring the distance between a metal cation and a separate covalently-bound fluorine atom and comparing it to an established cutoff value. Below the cutoff value, the M···F interaction is considered significant in that it may provide stabilization of the metal cation.[14] Since the metal is cationic, not a neutral atom, we determined our cutoff values by adding the van der Waals radii of the metal ions (first column, **Table S1**) as calculated by Merz, Li, *et al.*,[1] to the consistent van der Waals radii of fluorine as confirmed by Truhlar, *et al.* (r_{vdW}F = 1.47 Å).[2] For example, Mg···F distances less than 2.59 Å would be considered a secondary interaction. For comparison, a CSD search was carried out to identify alkaline earth metal fluoroalkoxide complexes similar to those reported here. Any M···F interactions, of any length, that were reported in those publications by the authors are summarized in **Table S1** as a range listing smallest reported interaction to longest reported interaction. Detailed lists of each compound and the reported interactions are given in **Tables S2** and **S3**.

†† In a similar manner, we also explore the stabilizing role of M···π secondary interactions. The maximum distance considered to be a M···π interaction in this work is the sum of van der Waals radii of the metal ions given in **Table S1**[1] and the van der Waals radius of carbon (r_{vdW}C 1.70 Å).[2] The cutoff values for heavy alkaline earth metal M···π interactions are explored experimentally and through density functional theory (DFT) calculations in our recent work on tetraarylborates.[28]

†††For π ···π interactions, the cutoff of 3.9 Å is measured as a centroid-centroid distance regardless of displacement from the parallel stack of the phenyl rings. For π ···H and F···H interactions, the source of

the hydrogen atom is indicated with a subscript, where relevant. For example, $\pi \cdots H_{Ph}$ indicates a T-shaped interaction between two phenyl rings in which the *pi*-system of one phenyl interacts with a hydrogen atom of the second phenyl. For X-ray crystallographic data, the $\pi \cdots H_{Ph}$ interaction is the distance from the centroid of one phenyl ring to the carbon atom of the other. For computational studies, the $\pi \cdots H_{Ph}$ interaction is a range of distances from each of the six carbon atoms of one phenyl to one hydrogen atom on the other phenyl, which also gives an indication of how centered it is over the ring. Finally, the F···H cutoff is determined by the sum of the van der Waals radius of fluorine (see above) and hydrogen (r_{vdW}H = 1.10[2] – 1.20[23] Å).

Table S2. List of M···F interactions for L^1-L^3, L: selected published and presented compounds using fluoroalkoxide ligands that do not have neutral donor appendages. Definitions of L^1-L^3, L are found in **Figure 1** of the paper. *Indicates disorder on that -CF_3 moiety. **Also has three Sr···π interactions (3.27-3.42 Å) as discussed in the paper.

**Formula with ligands defined in Figure 1**	**M^{+2}···F (Å) reported**	**Reference in SI**
**L^3**		
cis-$Mg(L^3)_2(dme)_2$	—	29
cis-$Ca(L^3)_2(thf)_4$	—	7
cis-$Ca(L^3)_2(dme)_2$	3.03*	7
trans-$Ca(L^3)_2(diglyme)_2$ one diglyme not fully coordinated	—	7
cis-$Sr(L^3)_2(thf)_4$	3.25*	7
cis-$Sr(L^3)_2(dme)_2$	3.24*	7
trans-$Sr(L^3)_2(diglyme)_2$	—	7
trans-$Ba(L^3)_2(dme)_3$	—	7
trans-$Ba(L^3)_2(diglyme)_2$	—	7
**L^1**		
$[(dme)_2Mg(\mu\text{-}L^1)_2Mg(L^1)_2]$ Similar to **4**	2.972	3
$[Li_2(thf)_4Mg(\mu^2\text{-}L^1)_4]$	2.894(4)-2.984(5)	30
$[Li_3(thf)_2(^{n}BuMg)(\mu^3\text{-}L^1)_4]$	—	30
$Ba_5(\mu^5\text{-}OH)[\mu^3\text{-}L^1]_4[\mu^2\text{-}L^1]_4(L^1)(thf)_4H_2O\cdot(thf)$	2.99 – 3.31	16
**L^2**		
$[MgCl(thf)(\mu^2\text{-}L^2)_2Mg(thf)(\mu^2\text{-}Cl)_3Mg(thf)_3]$	3.131	31
**L**		
$Mg(L)_2(thf)_2$ (**1**)	—	
$Mg(L)_2(dme)_2$	—	32
trans-$Ca(L)_2(thf)_4$ (**2**)	—	
cis-$Sr(L)_2(thf)_4$ (**3**)	—	
$[Sr_2(L)_4(thf)_3]$ (**4**)	<u>Sr1</u>	

	2.65 (F7), 2.71 (F19), 2.73 (F13), 2.81 (F16), 2.88 (F10), 2.91 (F22)		
$[Sr_3(L)_6(ether)_2]$ (**5**)**	<u>Sr1</u><br><br>3.07 (F16)<br><br><u>Sr3</u><br><br>3.12 (F29)	<u>Sr2</u><br><br>2.75 (F7), 2.85 (F10), 2.85 (F25), 2.80 (F19), 2.89 (F13), 2.89 (F22), 3.11(F28)	
$[Ba(L)_2]_\infty$ ether (**6**)	<u>Ba1</u><br><br>2.99 (F1), 2.98 (F13), 3.00 (F19), 3.06 (F7), 3.32 (F20)	<u>Ba2</u><br><br>2.96 (F16), 2.97 (F11), 2.98 (F5), 2.98 (F22)	
$[Ba(L)_2]_\infty$ (**7**)	<u>Ba1</u><br><br>2.95 (F16), 2.96 (F7), 3.01 (F22), 3.08 (F1), 3.18 (F20), 3.31 (F19)<br><br><u>Ba3</u><br><br>2.90 (F46), 2.95 (F25), 3.02 (F31), 3.04 (F40)	<u>Ba2</u><br><br>2.99 (F4), 3.09 (F13), 3.13 (F11)<br><br><u>Ba4</u><br><br>2.96 (F28), 2.99 (F34), 3.04 (F37), 3.19 (F43)	
**Magnesium fluoroaryloxides**			
$Mg(2,4,6\text{-}(CF_3)_3C_6H_2O)_2(thf)_3$	—		5
$Mg(O(2\text{-}4\text{-}{}^tBu_2)\text{-}6\text{-}(C(OH)(CF_3)_2))_2(thf)_2$	2.5090(13)<br>2.6716(12)		6
$[Mg(OC(C_6F_5)_3)_2]_2$	2.199(2)<br>2.269(2)<br>2.356(2)<br>2.166(2)<br>2.255(2)<br>2.742(2)		4
$[(thf)_2Mg(\mu\text{-}O(2\text{-}4\text{-}{}^tBu_2)\text{-}6\text{-}(C(O)(CF_3)_2))_2Mg(thf)]$	2.3148(11)		6
$[Mg_5(\mu\text{-}O(2\text{-}4\text{-}{}^tBu_2)\text{-}6\text{-}(C(O)(CF_3)_2))_4(\mu\text{-}O^nBu)_2(thf)_4]$	2.4881(13)<br>2.5564(12)<br>2.3783(12)<br>2.4423(12)<br>2.4401(12)<br>2.5285(12)		6

Figure S1. Structures of fluoroalkoxides used as ligands (L^4-L^{15}) in compounds listed in **Table S3** for comparison of M···F interactions. Fluorinated alkoxides that include appendages with neutral donor atoms, such as those shown here, have been used to improve the volatility and stability of alkaline earth metal complexes.

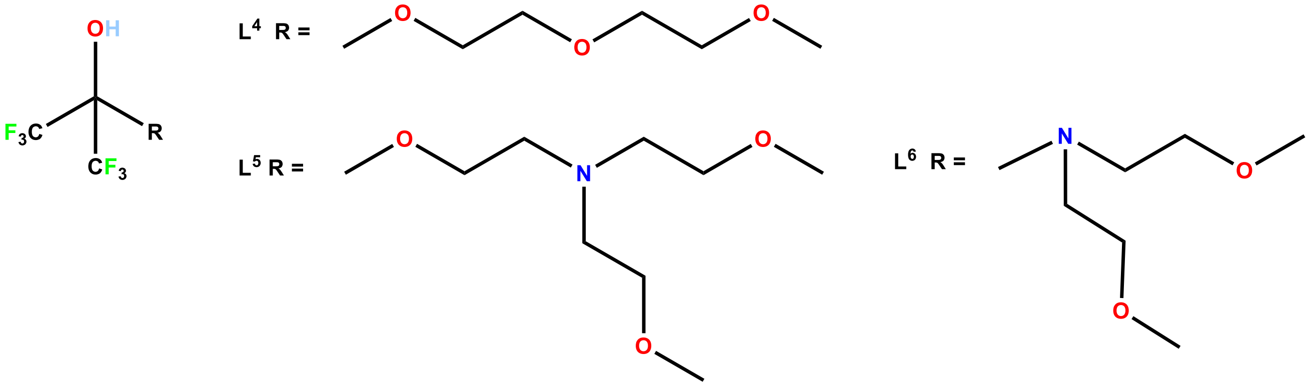

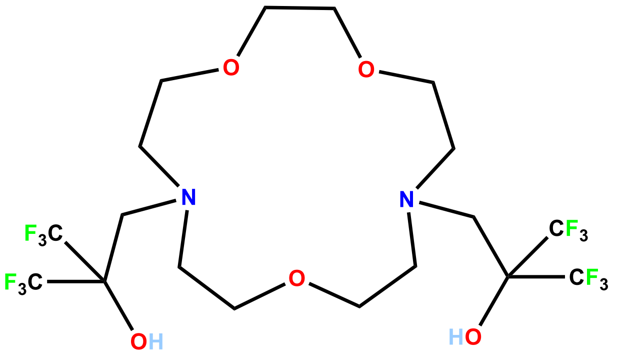

L^7

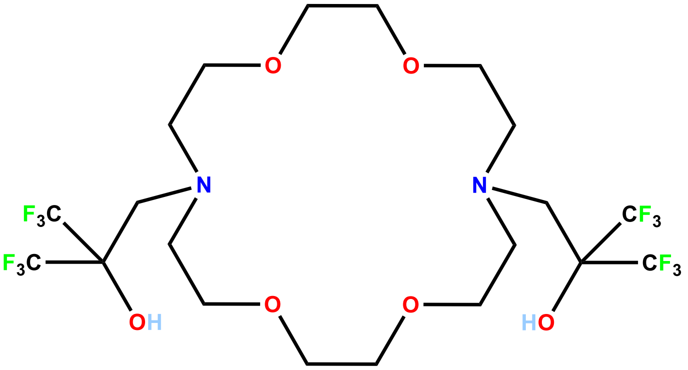

L^8

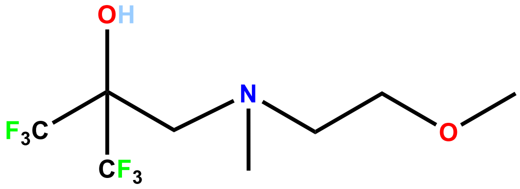

L^9 = HOC(CH$_2$N(CH$_2$CH$_2$OCH$_3$)(CH$_3$))(CF$_3$)$_2$

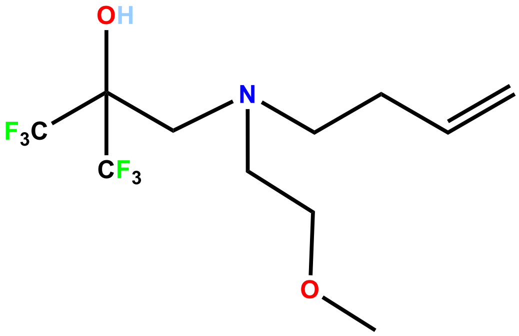

L^{10} = HOC(CH$_2$N(CH$_2$CH$_2$CHCH$_2$)(CH$_2$CH$_2$OCH$_3$))(CF$_3$)$_2$

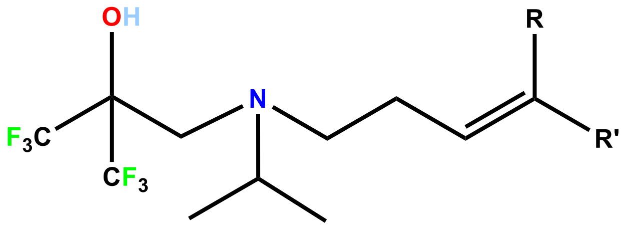

L^{11}-H_2 (R = H; R' = H)
L^{11}-Me (R = H; R' = Me)
L^{11}-Me_2 (R = Me; R' = Me)

L^{11} = HOC(CH$_2$N(CH$_2$CH$_2$CHCRR')(^{i}Pr))(CF$_3$)$_2$

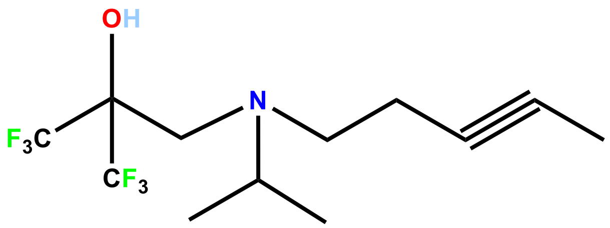

L^{12} = HOC(CH$_2$N(CH$_2$CH$_2$CCH$_3$')(^{i}Pr))(CF$_3$)$_2$

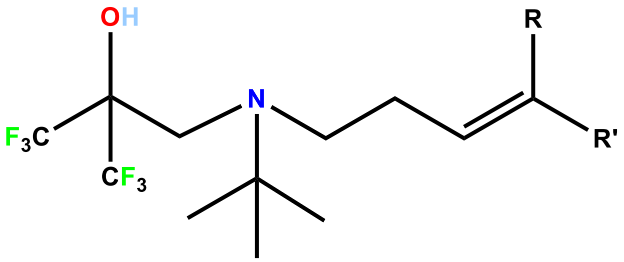

L^{13}-H_2 (R = H; R' = H)

L^{13} = HOC(CH$_2$N(CH$_2$CH$_2$CHCRR')(^{t}Bu))(CF$_3$)$_2$

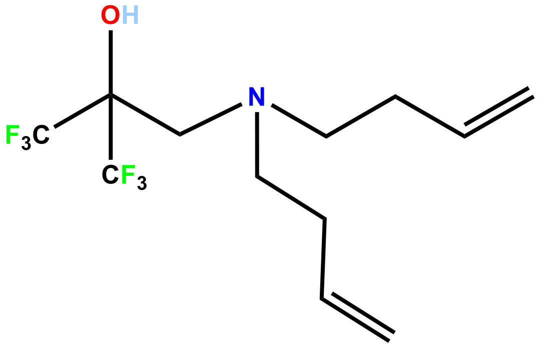

L^{14} = HOC(CH$_2$N(CH$_2$CH$_2$CHCH$_2$)$_2$(CF$_3$)$_2$

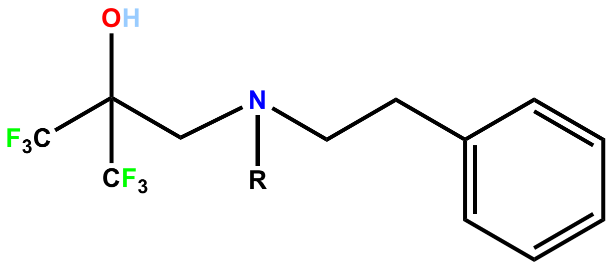

L^{15}-Me (R = Me)
L^{15}-^{i}Pr (R = ^{i}Pr)

L^{15} = HOC(CH$_2$N(CH$_2$CH$_2$Ph)(Me))(CF$_3$)$_2$

Table S3. List of M···F interactions for alkaline earth metal complexes of L[4]-L[15], fluoroalkoxide ligands that have a neutral donor appendage. Ligands defined above in **Figure S1**. *Compounds of this ligand also reported as having agostic interactions. ** Compounds of this ligand also reported as having both agostic interactions and M···π interactions.

**Formula with ligands defined in Figure S1**	**$M^{+2}\cdots F$ (Å) reported**	**Reference**
**L^4** 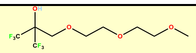		
$Sr(\eta^{1+3}L^4)_2$	—	15
$Ba(\eta^{1+3}L^4)_2$	—	15
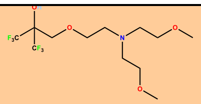 **L^5**		
$[Sr(\mu\text{-}\eta^{1+4}L^5)(\eta^{1+0}L^5)]_2$	3.133(4), 3.140(4)	15
$[Ba(\mu\text{-}\eta^{1+4}L^5)(\eta^{1+0}L^5)]_2$	3.212(4), 3.358(6)	15
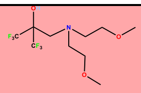 **L^6**	*	
$[(\mu\text{-}\eta^{1+2}L^6)CaN(SiMe_2H)_2]_2$	2.833(1)	8
$[(\mu\text{-}\eta^{1+2}L^6)CaN(SiMe_3)_2]_2$	2.920(1), 3.113(2)	8
$Sr(\eta^{1+3}L^6)_2$	—	17
$Ba(\eta^{1+3}L^6)_2$	3.132(2), 3.212(3)	17
**L^7** 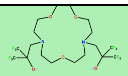		
$Sr(\eta^{2+5}\text{-}L^7)H_2O$	2.863(3)	13
$Sr(\eta^{2+5}\text{-}L^7)thf$	—	13
$[Ba(u\text{-}\eta^{2+5}\text{-}L^7)]_2$	2.998(3)	13
**L^8** 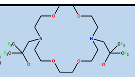		
$Ca(\eta^{2+6}\text{-}L^8)$	—	13
$Sr(\eta^{2+6}\text{-}L^8)$	3.1724(10), 3.1724(10)	13
$Ba(\eta^{2+6}\text{-}L^8)$	3.1351(15)	13
**L^9** 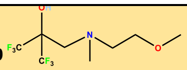	*	
$[(\mu\text{-}\eta^{1+2}L^9)CaN(SiMe_2H)_2]_2$	3.005(1)	8

$[(\mu\text{-}\eta^{1+2}L^9)CaN(SiMe_3)_2]_2$	2.712(6), 3.084(6), 3.056(6)	8
**L^{10}** 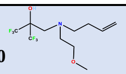	*	
$[(\mu\text{-}L^{10})CaN(SiMe_2H)_2]_2$	3.036(2), 3.119(2)	10
$[(\mu\text{-}L^{10})SrN(SiMe_2H)_2]_2$	2.996(3), 3.049(2)	10
**L^{11}-H_2** 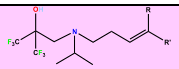	**	
$[(\mu\text{-}\eta^{1+1}L^{11}\text{-}H_2)CaN(SiMe_2H)_2]_2$	3.050(2)	9
$[(\mu\text{-}\eta^{1+1}L^{11}\text{-}H_2)SrN(SiMe_2H)_2]_2$	3.050(2), 3.136(2)	9
**L^{11}-Me** 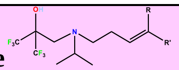	**	
$[(\mu\text{-}\eta^{1+1}L^{11}\text{-}Me)CaN(SiMe_2H)_2]_2$	2.834(1)–2.949(1)	9
**L^{11}-Me_2** 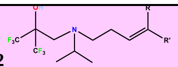	**	
$[(\mu\text{-}\eta^{1+1}L^{11}\text{-}Me_2)CaN(SiMe_2H)_2]_2$	2.605(1)	9
**L^{12}** 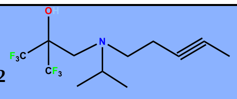	**	
$[(\mu\text{-}\eta^{1+1}L^{12})CaN(SiMe_2H)_2]_2$	2.753(1)	11
$[(\mu\text{-}\eta^{1+1}L^{12})SrN(SiMe_2H)_2]_2$	2.806(3)	11
**L^{13}** 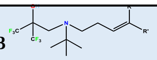	*	
$[Ca_3(\mu^2\text{-}\eta^{1+1}L^{13})_2(\mu^2\text{-}N(SiMe_2H)_2)_2(N(SiMe_2H)_2)_2]$	3.011(2), 3.052(2)	12
**L^{14}** 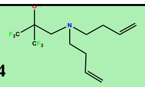	**	
$[Ca(\mu\text{-}\eta^{1+1}L^{14})(N(SiMe_2H)_2)]_2$	2.9309(15)	11
**L^{15}-Me** 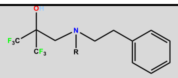	*	
$[Ca(thf)(\mu^2\text{-}\eta^{1+1}L^{15}\text{-}Me)(N(SiMe_2H)_2)]_2$	2.740(1), 2.949(1), 3.080(1)	11
$[Ca_3(\mu^2\text{-}\eta^{1+1}L^{15}\text{-}Me)_2(\mu^2\text{-}N(SiMe_2H)_2)_2(N(SiMe_2H)_2)_2]$	2.740(1), 2.949(1), 3.080(1)	11
**L^{15}-^{i}Pr** 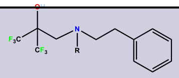	*	
$[Ca_3(\mu^2\text{-}\eta^{1+1}L^{15}\text{-}^{i}Pr)_2(\mu^2\text{-}N(SiMe_2H)_2)_2(N(SiMe_2H)_2)_2]$	2.745(4), 2.786(4)	12

Supplemental Information for X-ray crystallography results and discussion

Table S4. Intramolecular distances for **1** and a related compound, $Mg(HFPP)_2(dme)_2$,[32] based on solid-state X-ray crystallographic data. Cutoff values for interactions detailed in **Table S1**. The $\pi\cdots\pi$ centroid distances are provided only to establish that they are well-beyond the cutoff value (3.9 Å).

Intramolecular interaction distances given in Å							
	$\pi\cdots\pi$	$\pi\cdots H_{Ph}$		$\pi\cdots H_{donor}$		$F\cdots H_{Ph}$	$F\cdots H_{donor}$
**$Mg(HFPP)_2(thf)_2$**	7.26	—	—	—	—	—	2.65
**$Mg(HFPP)_2(dme)_2$**[29]	6.01	—	—	—	—	2.57<br>2.65<br>2.66	2.40<br>2.56<br>2.47<br>2.55

Figure S2. Images of intramolecular interactions in **1** and $Mg(HFPP)_2(dme)_2$[32] (a and b, respectively), generated from X-ray crystallographic data. The only intramolecular secondary noncovalent interactions between ligand and donor found were $F\cdots H_{donor}$ (green) and $F\cdots H_{Ph}$ (green). The $\pi\cdots\pi$ centroid distances (pink) are shown only to establish that they are well-beyond the cutoff value (3.9 Å).

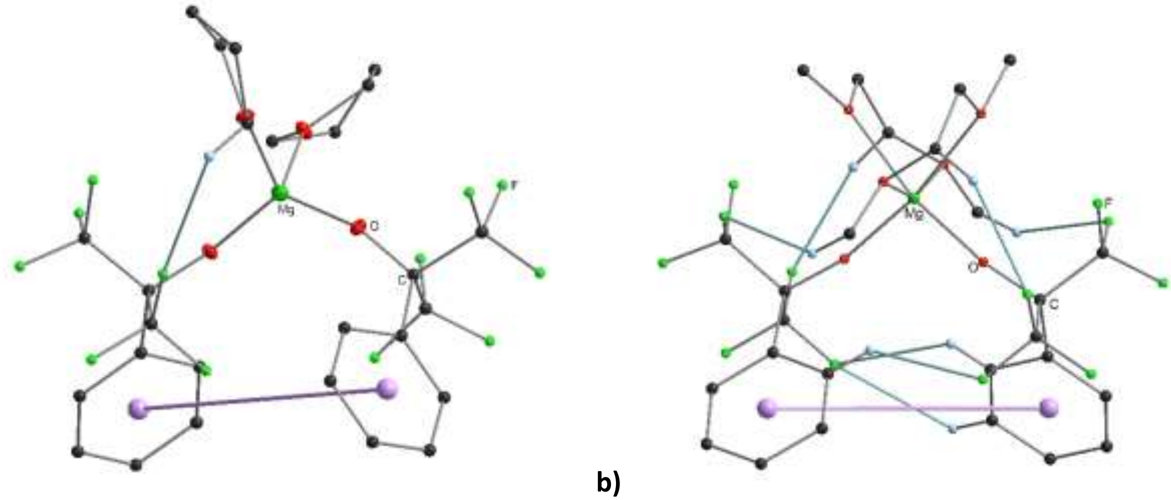

a) **b)**

Table S5. Intermolecular interactions in **1** and a related compound, Mg(HFPP)$_2$(dme)$_2$,[32] based on solid-state X-ray crystallographic data. Cutoff values for interactions detailed in **Table S1**. The $\pi\cdots\pi$ centroid distances are provided only to establish that they are well-beyond the cutoff value (3.9 Å).

**Intermolecular Interaction distances given in Å**					
	**$\pi\cdots\pi$**	**$\pi\cdots H_{Ph}$**	**$\pi\cdots H_{donor}$**	**$F\cdots H_{Ph}$**	**$F\cdots H_{donor}$**
			3.11 3.36		
			3.11 3.39		
			3.18 3.39		
			3.18 3.70	2.63 2.66	2.54 2.62
**Mg(HFPP)$_2$(thf)$_2$**	7.26	3.21	3.18 3.70	2.63 2.66	2.54 2.65
			3.18 3.72	2.67 2.67	2.62 2.65
			3.25 3.72		
			3.25 3.87		
			3.36 3.87		
			2.65 3.50	2.47	2.51 2.64
**Mg(HFPP)$_2$(dme)$_2$**[29]	6.01	3.02	2.65 3.71	2.47	2.57 2.64
		3.02	3.22 3.76	2.68	2.57 2.64
			3.22 3.86		

Figure S3. Images of $\pi\cdots$H intermolecular interactions in **1** and Mg(HFPP)$_2$(dme)$_2$[32] (a and b, respectively), generated from X-ray crystallographic data. Intermolecular interactions between ligand and donor shown here are $\pi\cdots H_{Ph}$ (gold) and $\pi\cdots H_{donor}$ (gold).

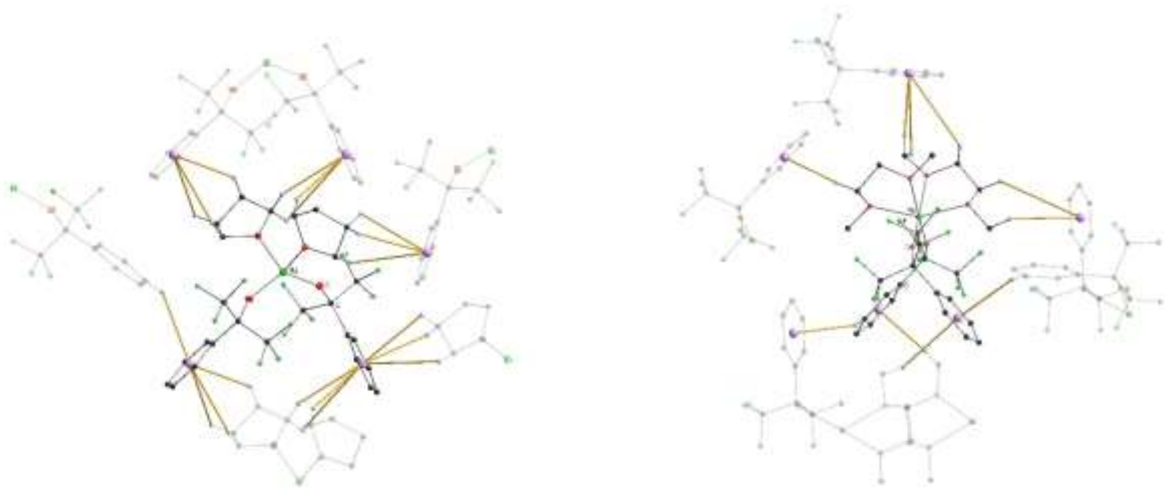

a) b)

Figure S4. Images of F⋯H intermolecular interactions in **1** and $Mg(HFPP)_2(dme)_2$[32] (a and b, respectively), generated from X-ray crystallographic data. Intermolecular interactions between ligand and donor shown here are $F\cdots H_{Ph}$ (green) and $F\cdots H_{donor}$ (green).

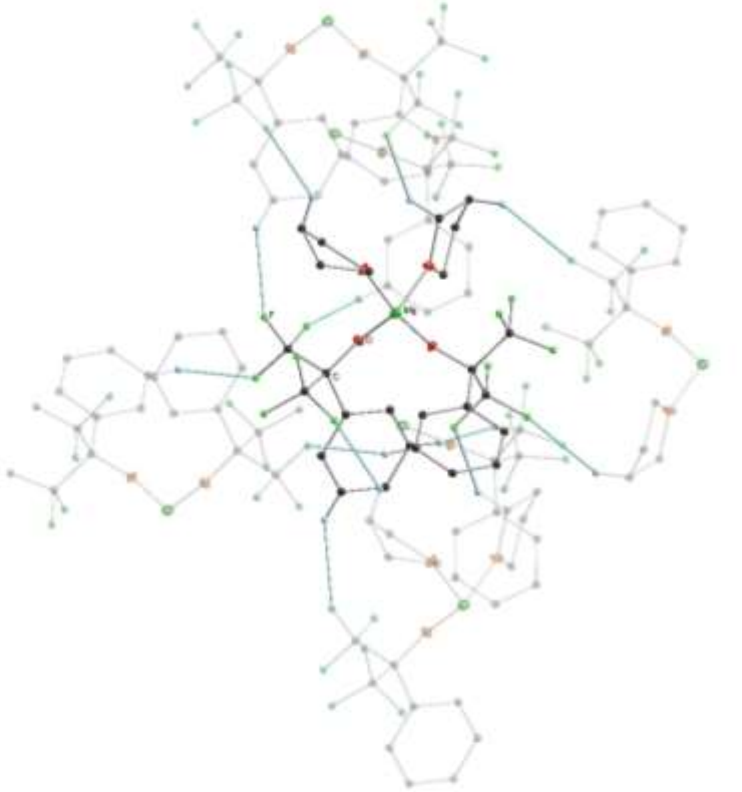

a)

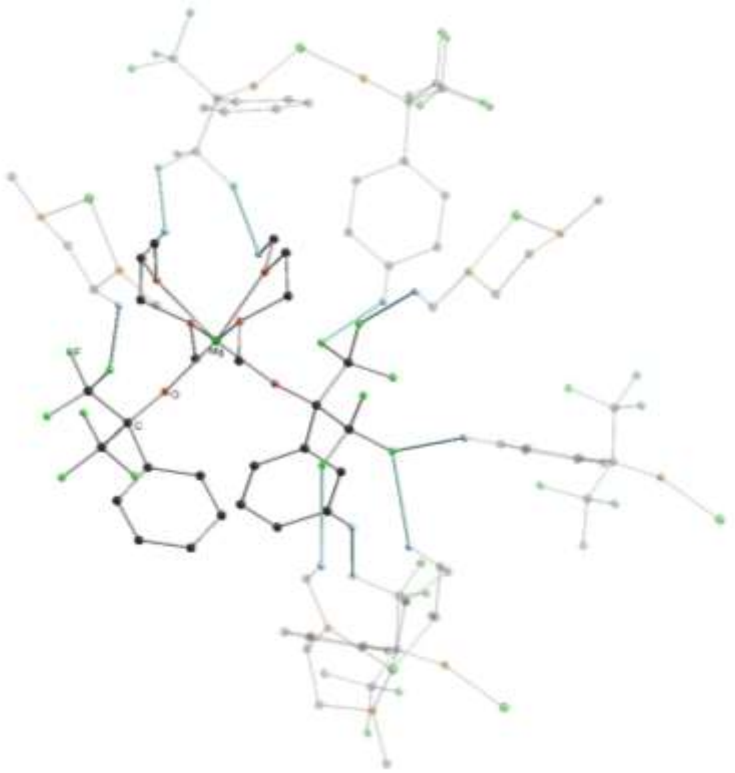

b)

Table S6. Intramolecular interactions in **2** and **3** based on solid-state X-ray crystallographic data. Cutoff values for interactions detailed in **Table S1**. The π···π centroid distances are provided only to establish that they are well-beyond the cutoff value (3.9 Å).

**Intramolecular Interaction distances given in Å**					
	**π···π**	**π···H_{Ph}**	**π···H_{THF}**	**F···H_{Ph}**	**F···H_{THF}**
**$Ca(HFPP)_2(thf)_4$**	10.41	—	3.74<br>3.74<br>3.81<br>3.81	—	2.42 2.73<br>2.42 2.73<br>2.46 2.73<br>2.46 2.73
**$Sr(HFPP)_2(thf)_4$**	7.62	—	3.86<br>3.86	—	2.42 2.64<br>2.57 2.72<br>2.63

Figure S5. Images of intramolecular interactions in **2** and **3** generated from X-ray crystallographic data. The intramolecular secondary noncovalent interactions between ligand and donor found were π···H_{THF} (gold) and F···H_{THF} (green). The π centroids are shown in pink.

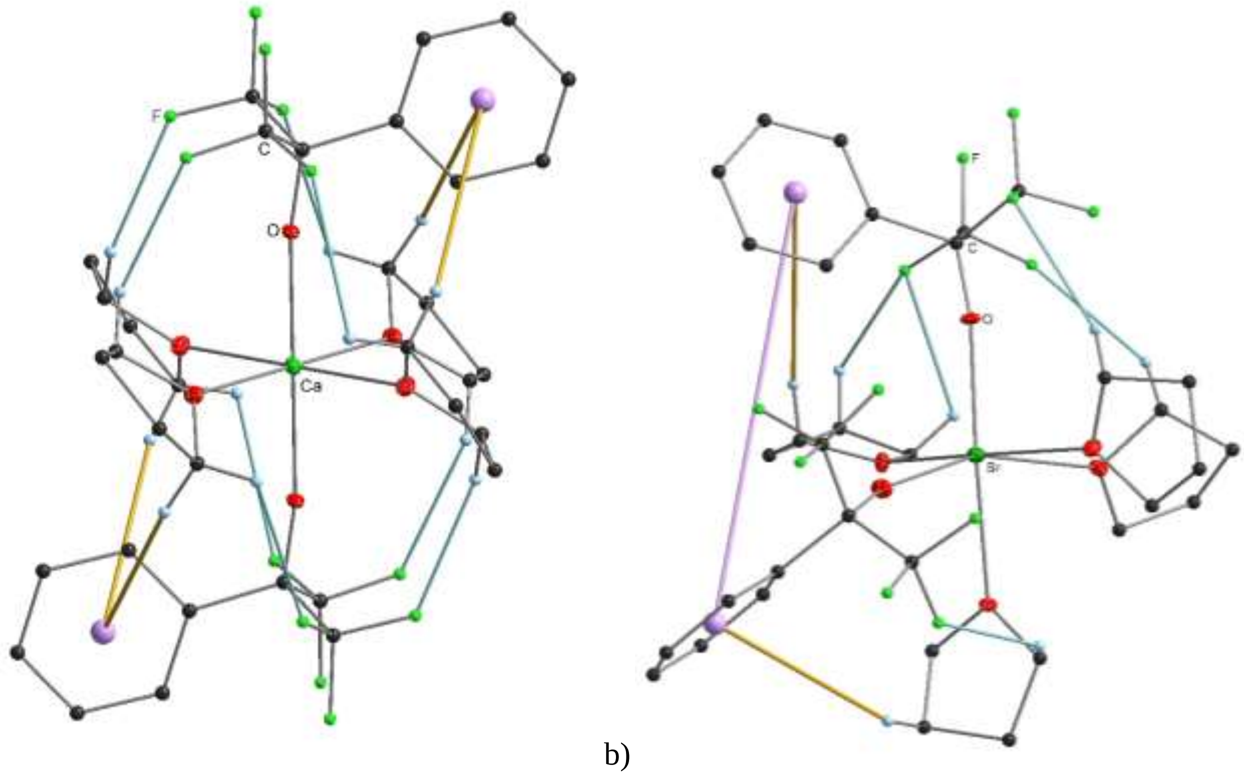

a) b)

Table S7. Intermolecular interactions in **2** and **3** based on solid-state X-ray crystallographic data. Cutoff values for interactions detailed in **Table S1**.

Intermolecular Interaction distances given in Å					
	$\pi\cdots\pi$	$\pi\cdots H_{Ph}$	$\pi\cdots H_{THF}$	$F\cdots H_{Ph}$	$F\cdots H_{THF}$
**$Ca(HFPP)_2(thf)_4$**	—	—	3.63 3.81<br>3.63 3.81<br>3.63 3.81<br>3.63 3.81	—	2.50<br>2.50<br>2.50<br>2.50
**$Sr(HFPP)_2(thf)_4$**			2.77 3.70<br>2.77 3.72<br>3.17 3.72<br>3.41 3.74<br>3.41 3.77<br>3.52 3.77<br>3.52 3.82<br>3.55 3.82<br>3.68 3.85<br>3.68 3.85<br>3.69 3.86<br>3.69 3.87	2.68	2.49 2.66<br>2.49 2.66<br>2.55 2.68<br>2.56 2.68<br>2.62

Figure S6. Images of π···H intermolecular interactions in **2** and **3** generated from X-ray crystallographic data. The intermolecular interactions between ligand and donor shown here are $\pi\cdots H_{THF}$ (gold). The π centroids are shown in pink.

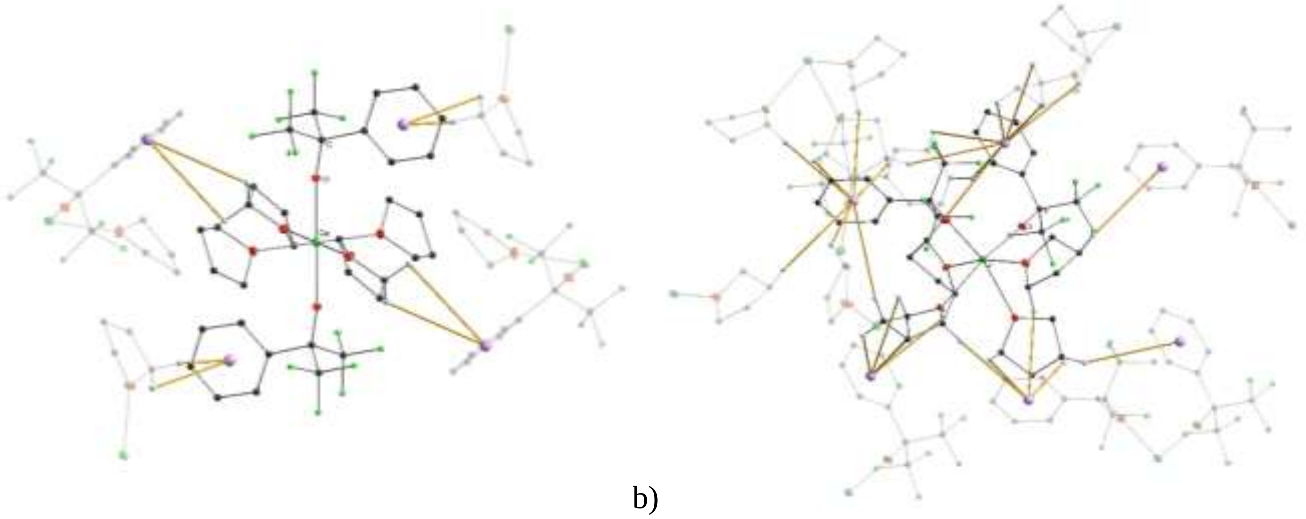

a) b)

Figure S7. Images of F···H intermolecular interactions in **2** and **3**, generated from X-ray crystallographic data. The intermolecular interactions between ligand and donor shown here are F···H_{Ph} (green), and F···H_{THF} (green).

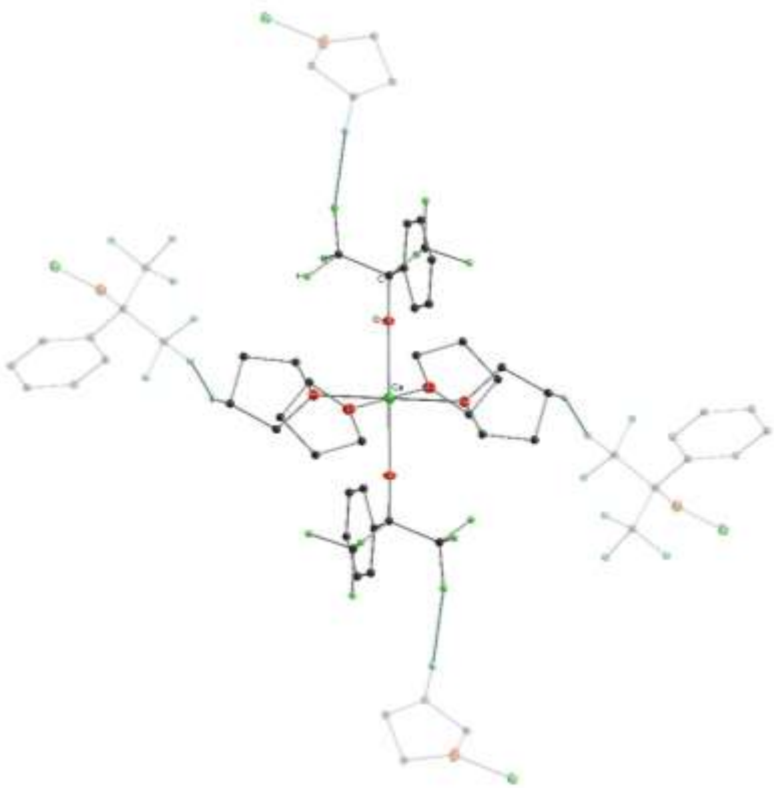

a)

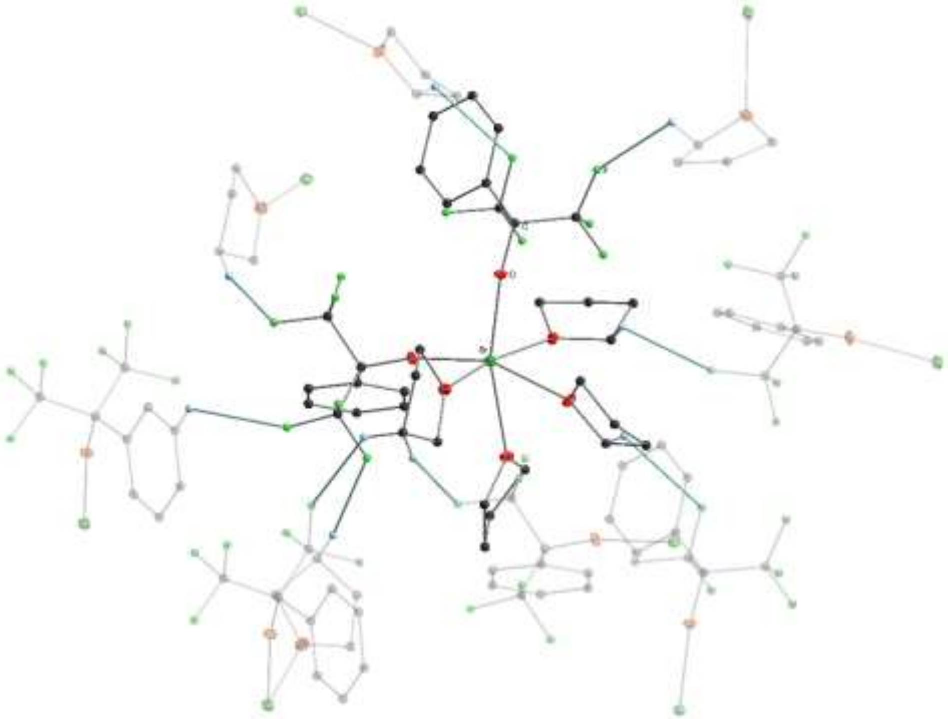

b)

Supplemental Information for computational studies results and discussion

[Mg(OC(Ph)(CF_3)$_2$)$_2$(thf)$_2$] (1). The DFT-based conformational survey further emphasizes the apparent absence of Mg···F interactions as structural influences in the isolated geometry of **1**. CREST-XTB-GFN2 sampling produced 77 initial configurations, from which B3LYP(GD3-BJ)/6-31G(d,p) optimizations and normal mode analyses yielded 89 unique configurations (with some GFN2 geometries initially optimizing to transition states between new B3LYP minima). Final optimizations at the lc-ωPBE(GD3-BJ)/Def2-TZVP level of theory and clustering of these 89 structures based on phenyl-phenyl centroid distance (within 0.25 Å), structure RMSD (less than 0.05 Å), and relative energy difference (less than 0.2 kJ/mol) criteria (which combined address the lack of persistent CF_3 orientations and some limited conformational variation among the THFs) resulted in 23 unique conformations. These geometries are summarized in **Table S8** with relevant geometries shown in **Figure S8**.

Table S8. Family of predicted gas-phase configurations of **1**. Relative energies, key structural parameters, significant intramolecular (*Sig. Intra.*) interactions observed, and the number of M···F distance less than 2.7 Å with the range of those distances among the 23 predicted minima for **1**.

Config.	Rel. E. (kJ/mol)	Ph-Ph Centroid (Å)	Sub-3 Å Mg···F	RMSD (from 1a)	Sig. Intra. Interactions	No. of sub-2.7 Å F···H Distances
1a	0.00	7.517		0.000	$\pi\cdots H_{THF}$	10 (2.279 – 2.673)
1b	2.16	3.837		2.507	$\pi\cdots\pi$	7 (2.259 – 2.644)
1c	2.80	3.825		2.433	$\pi\cdots\pi$	6 (2.251 – 2.655)
1d	4.58	4.874		2.214	$\pi\cdots H_{Ph}$	5 (2.287 – 2.692)
1e	5.05	4.932		2.134	$\pi\cdots H_{Ph}$	5 (2.286 – 2.675)
1f	6.86	4.979		2.368	$\pi\cdots H_{Ph}$	5 (2.292 – 2.544)
1g	7.16	5.871		1.900	$\pi\cdots H_{Ph}$	6 (2.267 – 2.570)
1h	7.80	4.914		2.204	$F\cdots H_{Ph/THF}$	6 (2.288 – 2.684)
1i	8.49	7.688		2.054	$F\cdots H_{Ph/THF}$	6 (2.284 – 2.656)
1j	9.28	7.197		1.262	$F\cdots H_{Ph/THF}$	8 (2.375 – 2.684)
1k	9.38	9.464		2.476	$\pi\cdots H_{THF}$	9 (2.371 – 2.699)
1l	9.63	8.741		2.084	$F\cdots H_{Ph/THF}$	10 (2.381 – 2.695)
1m	10.84	9.434	2.954	2.532	$F\cdots H_{Ph/THF}$	8 (2.367 – 2.630)
1n	11.49	8.758		2.086	$\pi\cdots H_{Ph}$	5 (2.291 – 2.629)
1o	11.58	4.845		2.185	$F\cdots H_{Ph/THF}$	8 (2.331 – 2.652)

1p	11.83	9.030		2.243	$F\cdots H_{Ph/THF}$	8 (2.335 – 2.664)
1q	11.94	6.013		2.214	$F\cdots H_{Ph/THF}$	9 (2.391 – 2.673)
1r	12.02	9.477	2.944	2.468	$F\cdots H_{Ph/THF}$	5 (2.269 – 2.609)
1s	12.64	7.443		1.793	$F\cdots H_{THF}$	6 (2.361 – 2.529)
1t	13.09	7.461		1.801	$F\cdots H_{THF}$	6 (2.371 – 2.569)
1u	13.23	7.400		1.908	$F\cdots H_{THF}$	6 (2.358 – 2.572)
1v	13.31	8.692		2.358	$F\cdots H_{Ph/THF}$	7 (2.342 – 2.682)
1w	13.47	9.045	2.923	2.306	$F\cdots H_{Ph/THF}$	8 (2.288 – 2.684)
Crystal	n/a	7.251		2.219	$F\cdots H_{Ph/THF}$	6 (2.333 – 2.646)

Figure S8. Key structural motifs among the final configurations of **1** from DFT-based conformational survey, including two views of the predicted global minimum geometry containing a pair of $\pi\cdots H_{THF}$ stacking interactions (**1a**, left top and bottom), the prominent $\pi\cdots\pi$ stacking motif from the solvent model calculations (**1b** as represntative, top middle) compared to the more separated phenyl rings in the crystal (**crystal**, bottom middle), and representative higher-energy families of interaction motifs among the series (**1d**, top right, containing $\pi\cdots H_{Ph}$ interactions and **1s**, bottom right, containing $F\cdots H_{THF}$ interactions). Labels, relative energies, and brief descriptors are provided in **Table S8** above.

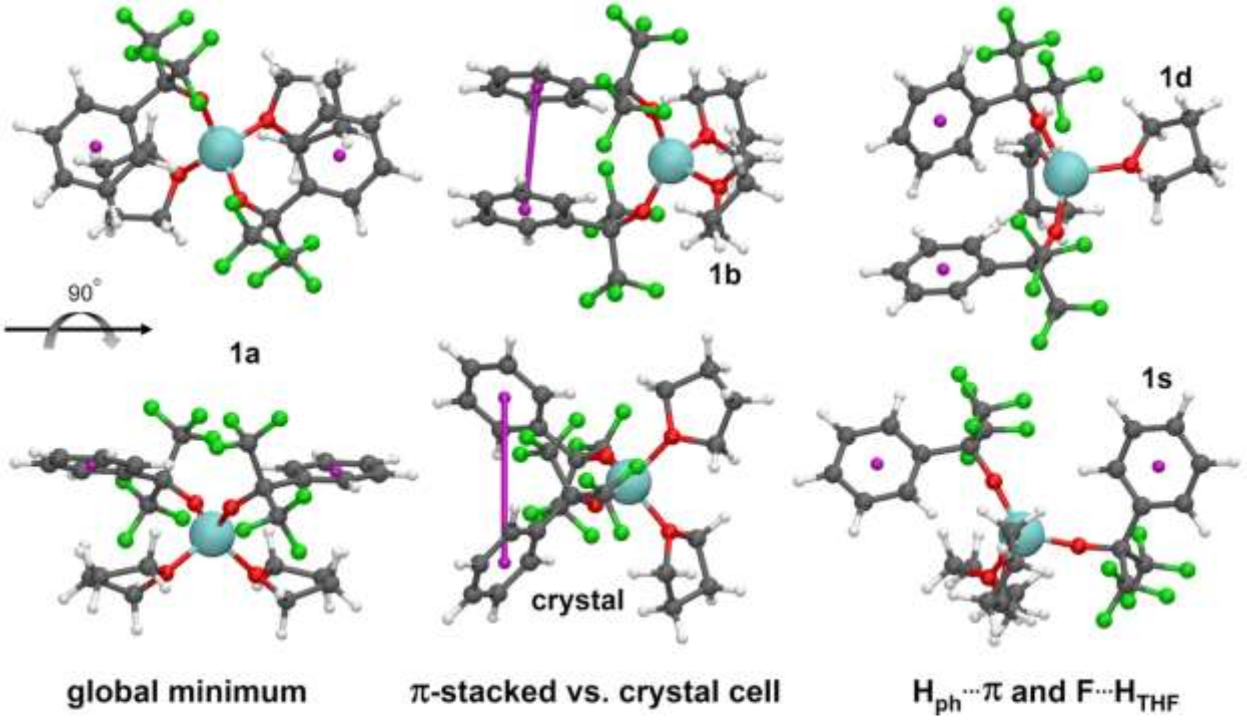

[*trans*-Ca(OC(CF_3)$_2$Ph)$_2$(thf)$_4$] (2) & [*cis*-Sr(OC(CF_3)$_2$Ph)$_2$(thf)$_4$] (3). This assessment began with CREST-XTB-GFN2 conformational sampling of **2** ("*trans*-Ca", producing 28 configurations) and **3** ("*cis*-Sr", producing 14 configurations), followed by swapping of the metal centers to produce **2**-Sr ("*trans*-Sr", 46 configurations) and **3**-Ca ("*cis*-Ca", 9 configurations). The DFT optimization workflow and uniqueness criteria used for **1** produced 16 *trans*-Ca (**Table S9**), and 21 *trans*-Sr configuration geometries (**Table S10**), while only producing four *cis*-Ca and two *cis*-Sr configurations (**Table S11**).

Table S9. Family of predicted gas-phase *trans*-configurations of **2** with Ca and their relative energies, phenyl-to-phenyl centroid distances, any M···F distances for a given geometry within 4.0 Å, RMSDs for the *trans*-Ca (sampled from the crystal geometry of **2**) conformational minima, and the number and range of F···H distances below 2.7 Å.

Config.	*Rel. E (kJ/mol)*	*Ph-Ph Centroid (Å)*	*Sub-4 Å M···F*	*RMSD (from a)*	*No. of sub-2.7 Å F···H Distances*
	Structure 2 With Ca (trans-Ca)				
a	*0.00*	*9.655*		*0.000*	*8 (2.251 – 2.565)*
b	*0.18*	*9.943*		*1.383*	*9 (2.267 – 2.694)*
c	*0.71*	*9.711*		*0.464*	*8 (2.249 – 2.680)*
d	*0.87*	*9.464*		*1.425*	*7 (2.241 – 2.621)*
e	*0.96*	*9.77*		*1.436*	*8 (2.240 – 2.664)*
f	*1.62*	*10.5*		*1.660*	*14 (2.321 – 2.698)*
g	*1.82*	*9.415*		*1.450*	*8 (2.250 – 2.653)*
h	*2.30*	*9.792*		*0.668*	*8 (2.244 – 2.638)*
i	*2.64*	*9.42*		*1.308*	*11 (2.241 – 2.672)*
j	*2.74*	*10.011*		*0.885*	*9 (2.254 – 2.686)*
k	*4.70*	*9.632*		*1.107*	*8 (2.245 – 2.684)*
l	*4.82*	*10.049*		*1.239*	*13 (2.266 – 2.694)*
m	*5.61*	*10.47*	*3.933, 3.938*	*1.686*	*8 (2.301 – 2.589)*
n	*5.73*	*10.381*	*3.911, 3.931*	*1.441*	*9 (2.330 – 2.616)*
o	*14.07*	*10.608*		*1.023*	*16 (2.288 – 2.680)*
p	*18.78*	*10.391*	*3.974, 3.997*	*0.988*	*12 (2.237 – 2.666)*
Crystal	*n/a*	*10.491*		*1.083*	*12 (2.354 – 2.673)*

Table S10. Family of predicted gas-phase *trans*-configurations of **2** with Sr and their relative energies, phenyl-to-phenyl centroid distances, any M···F distances for a given geometry within 4.0 Å, RMSDs for the *trans*-Sr (replacing Ca in structure **2** with Sr in the calculations) conformational minima, and the number and range of F···H distances below 2.7 Å.

Config.	*Rel. E (kJ/mol)*	*Ph-Ph Centroid (Å)*	*Sub-4 Å M···F*	*RMSD (from a)*	*No. of sub-2.7 Å F···H Distances*
	Structure 2 With Sr (trans-Sr)				
a	*0.00*	*9.648*		*0*	*5 (2.255 – 2.587)*
b	*0.79*	*9.946*	*3.955*	*1.243*	*9 (2.259 – 2.690)*
c	*1.92*	*9.725*	*3.977*	*1.679*	*8 (2.254 – 2.664)*
d	*1.93*	*9.872*		*0.399*	*10 (2.257 – 2.699)*
e	*2.38*	*10.182*		*0.629*	*10 (2.278 – 2.691)*
f	*2.69*	*10.023*	*3.912*	*0.559*	*9 (2.255 – 2.695)*
g	*2.80*	*10.138*	*3.804, 3.969*	*0.658*	*11 (2.252 – 2.693)*
h	*2.81*	*10.797*		*1.308*	*10 (2.344 – 2.664)*
i	*3.55*	*9.845*		*1.637*	*10 (2.251 – 2.599)*
j	*3.69*	*10.39*		*0.718*	*12 (2.262 – 2.612)*
k	*3.89*	*9.962*		*0.975*	*8 (2.262 – 2.686)*
l	*3.98*	*9.702*		*0.348*	*8 (2.256 – 2.686)*
m	*4.26*	*9.734*	*3.966*	*1.386*	*11 (2.261 – 2.692)*
n	*4.62*	*10.796*		*1.266*	*9 (2.331 – 2.650)*
o	*4.63*	*9.869*		*1.506*	*9 (2.263 – 2.669)*
p	*4.90*	*10.961*		*1.386*	*12 (2.317 – 2.693)*
q	*5.42*	*9.867*		*1.511*	*10 (2.264 – 2.699)*
r	*7.57*	*10.759*		*1.281*	*8 (2.311 – 2.637)*
s	*8.83*	*10.464*	*3.921*	*1.282*	*10 (2.242 – 2.648)*
t	*9.75*	*9.713*		*1.749*	*6 (2.248 – 2.636)*
u	*11.60*	*9.642*	*3.974*	*0.401*	*10 (2.265 – 2.689)*

Table S11. Families of predicted gas-phase *cis*-configurations of **3** with Ca and Sr and their relative energies, phenyl-to-phenyl centroid distances, any M···F distances for a given geometry within 4.0 Å, and RMSDs for the *cis*-Sr (sampled from the crystal geometry of **3**) and *cis*-Ca (replacing Sr with Ca in the calculations) conformational minima.

Config.	*Rel. E (kJ/mol)*	*Ph-Ph Centroid (Å)*	*Sub-4 Å M···F*	*RMSD (from a)*	*No. of sub-2.7 Å F···H Distances*
	Structure 3a With Ca (cis-Ca)				
a	*0.00*	*3.713*	*3.995, 3.995*	*0.000*	*18 (2.274 – 2.624)*
b	*0.41*	*3.848*	*3.990, 3.932*	*0.672*	*16 (2.231 – 2.679)*
c	*2.25*	*4.049*		*0.824*	*19 (2.232 – 2.688)*
d	*2.30*	*4.065*		*0.831*	*19 (2.231 – 2.685)*
	Structure 3a With Sr (cis-Sr)				
a	*0.0*	*3.828*	*3.997*	*0.000*	*14 (2.301 – 2.681)*
b	*2.22*	*3.898*	*3.994*	*0.719*	*15 (2.262 – 2.664)*
Crystal	*n/a*	*5.304*		*2.254*	*16 (2.271 – 2.675)*

X-ray crystallographic data collection and refinement details

All crystals suitable for single crystal X-ray diffractometry were removed under inert gas from a Schlenk and immediately covered with a layer of highly viscous Paratone oil. A single crystal was selected, mounted on a Mitegen microloop, and placed in the cold N_2 stream provided by an Oxford Cryosystems cryostream. XRD data collection was performed for compounds **1-7** on a Bruker Kappa Duo diffractometer[33] with Mo Kα radiation (λ= 0.71073 Å) and an Apex II CCD area detector. The unit-cell constants and the orientation matrices were determined by the program CELL_NOW.[34] Data integration was carried out using SAINT.[33] Empirical absorption corrections were applied using SADABS.[35,36] The structures were solved with use of the intrinsic phasing option in SHELXT[37] and refined by the full-matrix least-squares procedures in SHELXL[25,37–39] as implemented in the program SHELXLE.[40] The space group assignments and structural solutions were evaluated using PLATON.[41,42] Non-hydrogen atoms were refined anisotropically. Hydrogen atoms were placed in calculated positions corresponding to standard bond lengths and angles and refined using a riding model. Disorder was handled by modeling the occupancies of the individual orientations using free variables to refine the respective occupancy of the affected fragments (PART).[43] In some cases, the distances between arbitrary atom pairs were restrained to possess the same value using the SADI and the rigid-bond restraint RIGU and constraints (DELU, SIMU) were used to afford reasonable anisotropic displacement parameters for all fluorine and carbon atoms, in attempts to model thermal motion of -CF_3 groups, in order to make the ADP values of the atoms more reasonable. Disordered positions for one of the ethyl chains on one of the ether donor molecules in **[$Sr_3(\mu^2$-L)$_4$L$_2$(OEt$_2$)$_2$] (5)** were refined using 70/30 split positions. Compound **[*cis*-Sr(L)$_2$(thf)$_4$] (3)** was refined as a 2-component inversion twin (BASF 0.02). The severely disordered solvents of crystallization (ether) for compounds **[$Sr_3(\mu^2$-L)$_4$(L)$_2$(OEt$_2$)$_2$] (5)** and **[Ba(μ^2-**

L)$_2$]$_n$·$\frac{1}{4}$(OEt$_2$) (6) were removed from the refinement by using the "squeeze" option available in the PLATON program suite.[41,44] M···F and M···π interactions were determined by features of the programs Mercury[45] and Diamond.[46] All values for published compounds were based on a Cambridge Structural Database[47,48] search and all values for presented and published compounds fall within expected ranges.

All crystal structure representations were made with the program Diamond[46] with all non-carbon atoms, except fluorine atoms due to thermal movement, displayed as 30% ellipsoids. The next section of the SI contains crystallographic data and details of measurements and refinement for compounds **1-7**. CIF files were edited, validated and formatted either with the programs encifer,[49] publCIF,[50] or Olex2.[51] CCDC 2487076-2487082 contain the supplementary crystallographic data for compounds **1-7**. These data can be obtained free of charge from The Cambridge Crystallographic Data Centre *via* www.ccdc.cam.ac.uk/data_request/cif.

Computation software and other details

Conformational sampling of **1-3** was performed from the crystallographic geometries of each with CREST-XTB[52,53] using the GFN2-xTB method.[54] Conformers obtained from these calculations were optimized with the B3LYP hybrid density functional,[55] 6-31G(d,p) basis set,[56] and the D3 version of the Grimme dispersion correction with Becke-Johnson damping[57] as a preparative step before optimizations with the range-corrected lc-ωPBE density functional,[58] Def2-TZVP basis set,[59] and the D3 version of the Grimme dispersion correction with Becke-Johnson damping. All DFT optimizations were performed with an implicit benzene Polarizable Continuum Model (PCM) solvent reaction field (SCRF) applied.[60] Final geometries from the complete DFT survey were characterized as minima based on normal mode analyses. Calculations were performed with Gaussian09 ver. D.01[61] with program-option "ultrafine" grid sizes (integration grid of 99 radial shells and 590 angular points per shell) and "tight" convergence criteria (force criterion RMS $< 1.0 \times 10^{-5}$, density matrix RMS $< 1.0 \times 10^{-8}$). Images were generated with VMD[62] and POV-Ray.[63]

X-ray crystallographic data tables

Table S12. Crystallographic data and details of measurements for compounds **1-7** where L = $^{-}OC(Ph)(CF_3)_2$, and ether = OEt_2 Mo Kα (λ=0.71073Å). $R1= \Sigma/ |F_o|- |F_c|/|\Sigma|F_d$; $wR2 = [\Sigma_w(F_o^2-F_2^2)^2/\Sigma_w(F_o^2)^2]^{1/2}$

**Compound**	**[Mg(L)$_2$(thf)$_2$], (1)**	**[*trans*-Ca(L)$_2$(thf)$_4$], (2)**	**[*cis*-Sr(L)$_2$(thf)$_4$], (3)**	**[Sr$_2$(L)$_4$(thf)$_3$], (4)**	**[Sr$_3$(L)$_6$(ether)$_2$], (5)**	**[Ba(L)$_2$]$_\infty$·ether, (6)**	**[Ba(L)$_2$]$_\infty$, (7)**
Formula	$C_{26}H_{26}F_{12}MgO_4$	$C_{34}H_{42}F_{12}CaO_6$	$C_{34}H_{42}F_{12}SrO_6$	$C_{48}H_{44}F_{24}O_7Sr_2$	$C_{62}H_{50}F_{36}O_8Sr_3$[+ solvent]	$2(C_{36}H_{20}Ba_2F_{24}O_4)\cdot C_4H_{10}O$[+solvent]	$C_{36}H_{20}Ba_2F_{24}O_4$
Fw (g mol^{-1})	654.78	814.75	862.29	1364.07	1869.88	2568.52	1247.18
a (Å)	16.6777(15)	12.6977(10)	11.9947(6)	13.1727(11)	12.016(2)	24.382(6)	22.43(3)
b (Å)	8.6763(8)	11.1218(10)	16.9268(9)	23.1810(18)	17.386(3)	23.426(6)	16.325(18)
c (Å)	19.9669(18)	13.1986(11)	37.1150(19)	17.1105(13)	18.329(4)	16.401(4)	25.55(3)
α (°)	90	90	90	90	105.012(5)	90	90
β (°)	102.441(3)	99.732(2)	99.128(1)	91.859(2)	90.905(4)	90	114.905(13)
γ (°)	90	90	90	90	101.027(5)	90	90
V (Å^3)	2821.4(4)	1837.1(3)	7440.1(7)	5222.0(7)	3621.4(12)	9368(4)	8486(18)
Z	4	2	8	4	2	4	8
Crystal size (mm)	0.05 × 0.04 × 0.04	0.23 × 0.1 × 0.02	0.3 × 0.3 × 0.02	0.3 × 0.3 × 0.02	0.02 × 0.01 × 0.003	0.06 × 0.05 × 0.05	0.08 × 0.06 × 0.04
Crystal habit	Block, colourless	Plate, colourless	Plate, colourless	Plate, colourless	Plate, colourless	Block, colourless	Block, colourless
Crystal system	Monoclinic	Monoclinic	Monoclinic	Monoclinic	Triclinic	Orthorhombic	Monoclinic
Space group	$P2_1/n$	$P2_1/n$	*Cc*	$P2_1/c$	*P*-1	*Pbcn*	$P2_1/c$
d_{calc} (Mg m^{-3})	1.541	1.473	1.540	1.735	1.715	1.821	1.952
μ (mm^{-1})	0.17	0.27	1.55	2.18	2.35	1.81	1.99
T (K)	90(2)	90(2)	90(2)	90(2)	90(2)	90(2)	100(2)
θ range (°)	2.7–31.5	2.4–32.1	2.2–26.6	2.4–26.8	2.3–25.4	2.4–27.0	2.4–27.9
F(000)	1336	844	3520	2720	1848	4936	4768
T_{min}, T_{max}	0.638, 0.747	0.667, 0.746	0.666, 0.746	0.580, 0.746	0.295, 0.746	0.530, 0.746	0.467, 0.746
R_{int}	0.072	0.028	0.067	0.058	0.092	0.093	0.130
No. of measured, independent and observed [*I* > 2σ(*I*)] reflections	35994, 9908, 5031	18613, 5624, 4355	60221, 21865, 15167	48997, 11421, 8013	56822, 19125, 9364	136922, 12284, 8001	131746, 14921, 8737
independent reflections	9908	5624	21865	11421	19125	12284	14921
No. of parameters, restraints	388, 0	241, 0	956, 2	730, 0	1006, 12	619, 10	1189, 592
Δρmax, Δρmin (e Å^{-3})	0.58, -0.39	0.52, -0.39	0.49, -0.74	0.84, -0.73	0.79, -0.96	2.23, -1.65	1.25, -1.19
R1, wR2 (all data)	R1 = 0.1624 wR2 = 0.1248	R1 = 0.0592 wR2 = 0.1083	R1 = 0.0945 wR2 = 0.0873	R1 = 0.0739 wR2 = 0.0867	R1 = 0.1575 wR2 = 0.1515	R1 = 0.0975 wR2 = 0.1268	R1 = 0.1125 wR2 = 0.1265
R1, wR2 (>2*σ*)	R1 = 0.0661 wR2 = 0.1005	R1 = 0.0410 wR2 = 0.0996	R1 = 0.0489 wR2 = 0.0771	R1 = 0.0383 wR2 = 0.0757	R1 = 0.0596 wR2 = 0.1193	R1 = 0.0512 wR2 = 0.1087	R1 = 0.0504 wR2 = 0.0992

Characterization data

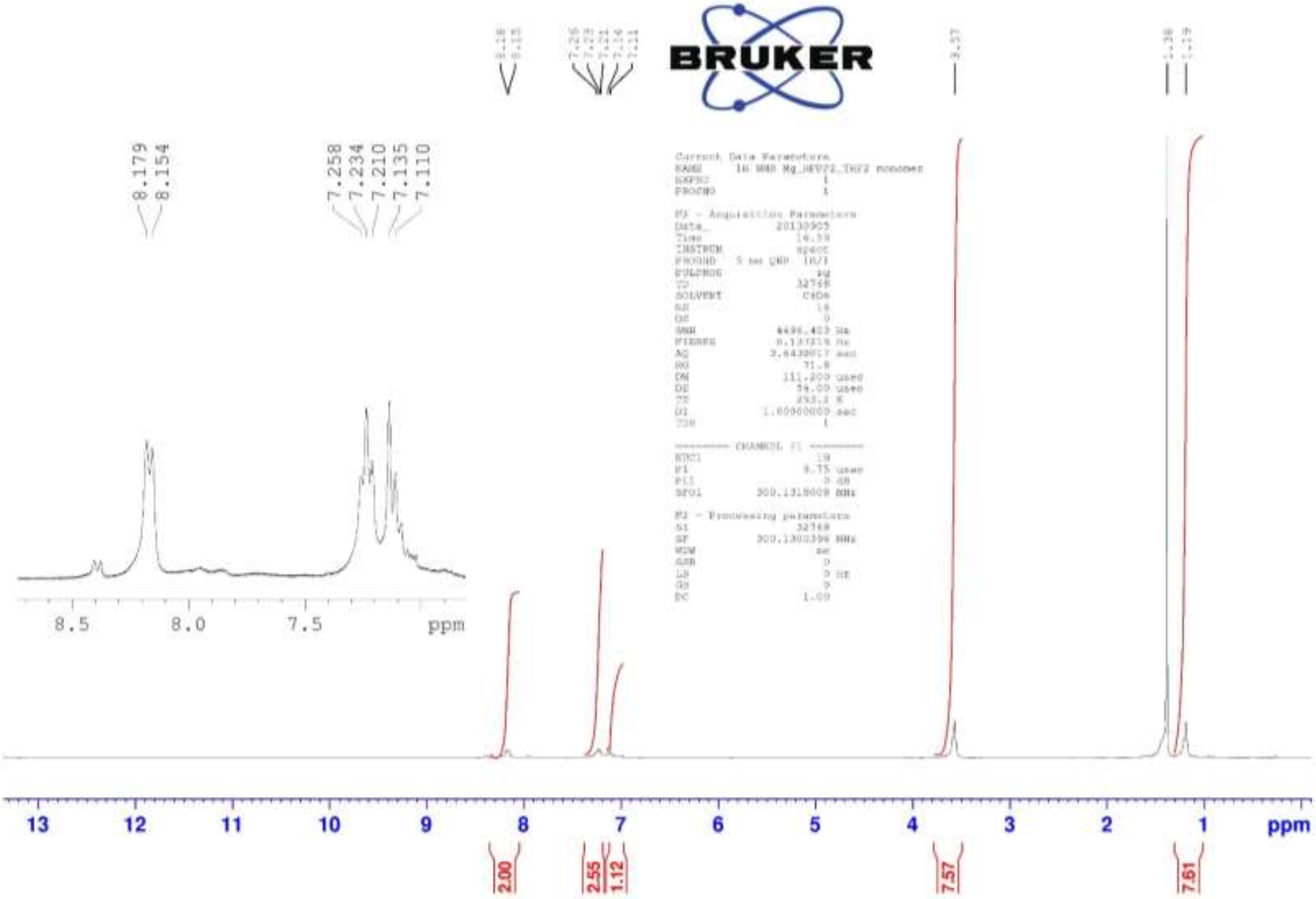

Figure S9. ^{1}H NMR $[Mg(OC(CF_3)_2Ph)_2(thf)_2]$ (**1**). 300 MHz, Solvent peak C_6D_6 at 7.14 ppm.

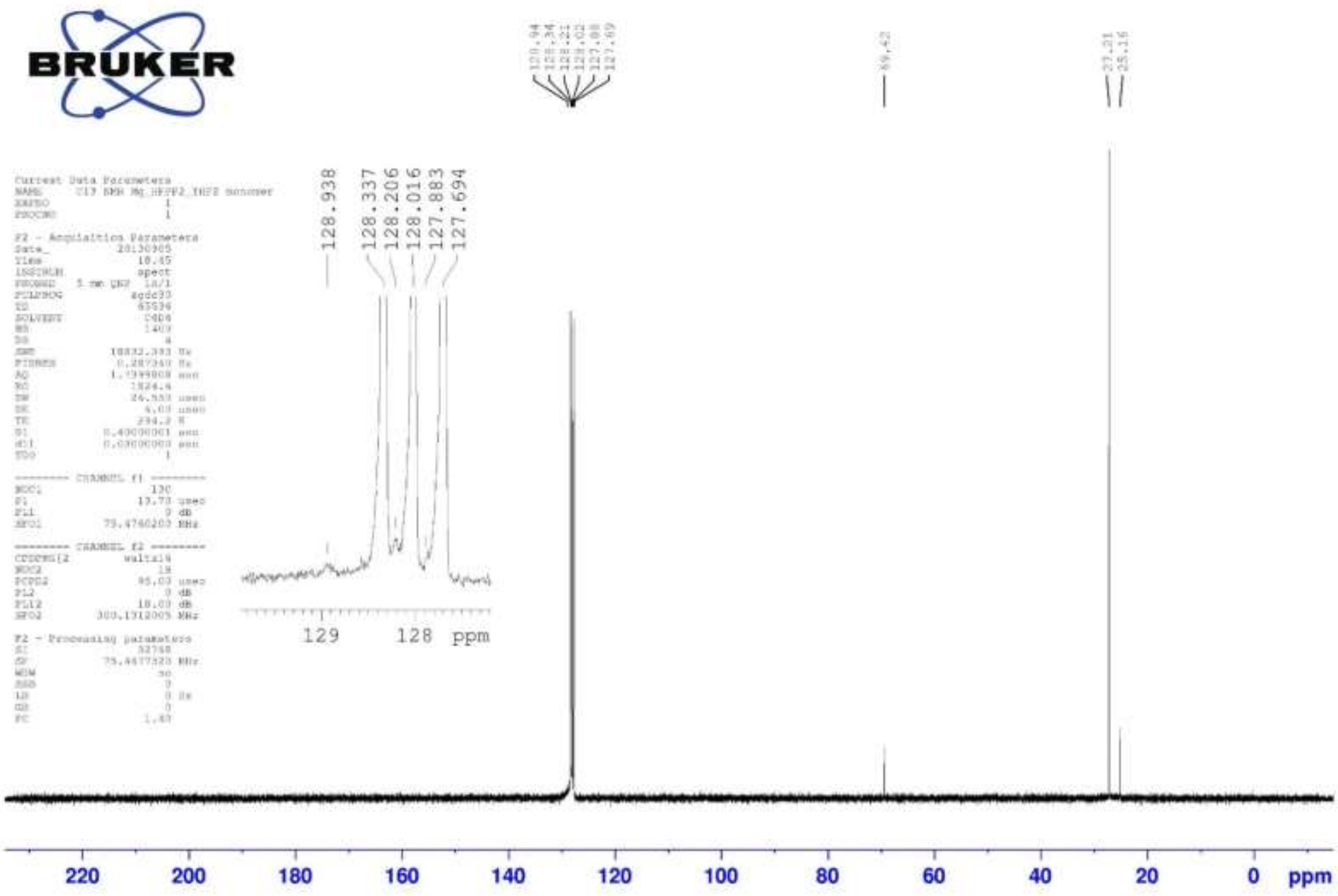

Figure S10. ^{13}C NMR $[Mg(OC(CF_3)_2Ph)_2(thf)_2]$ (**1**). 75 MHz, Solvent peak C_6D_6 at 128.02 ppm.

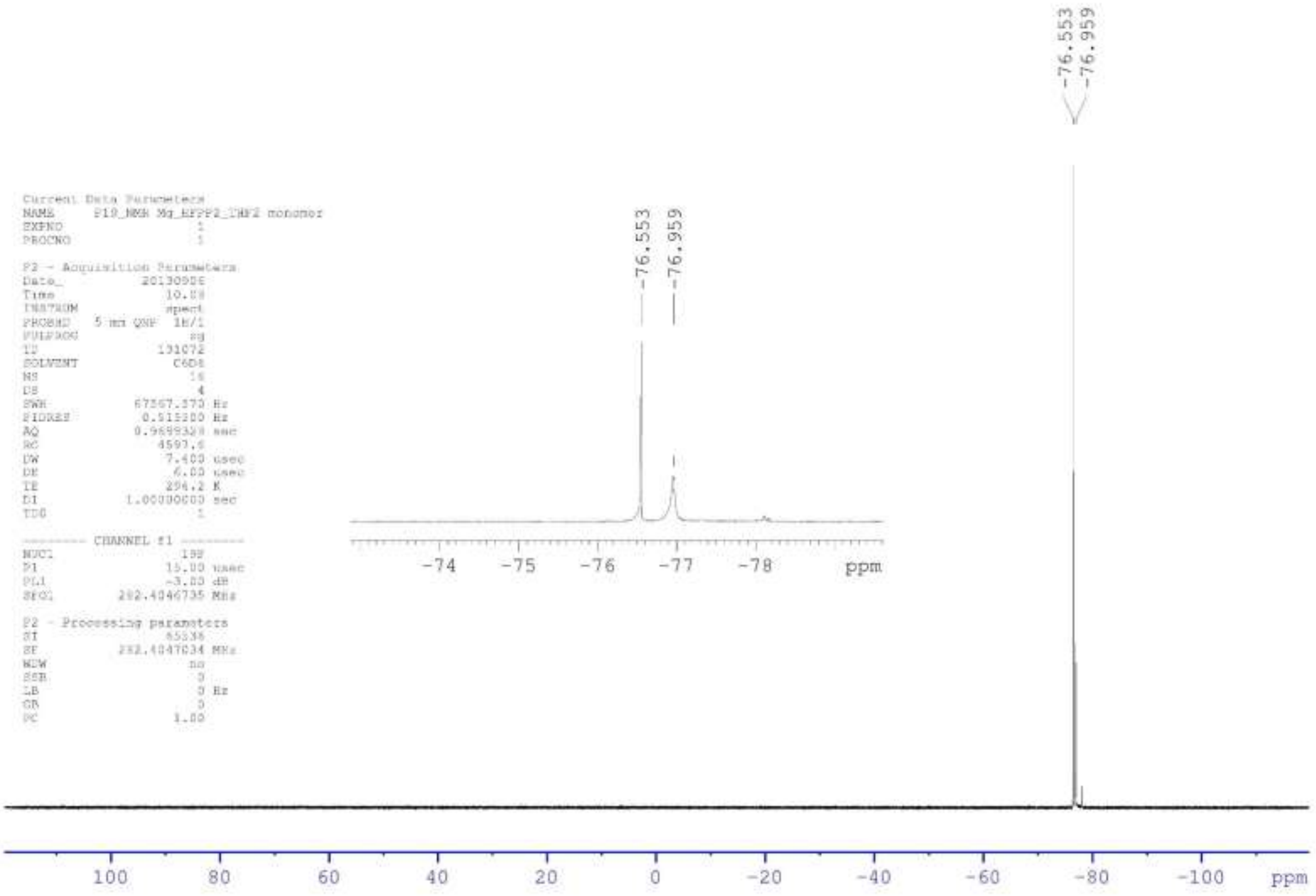

Figure S11. ^{19}F NMR $[Mg(OC(CF_3)_2Ph)_2(thf)_2]$ (**1**). 282 MHz, in C_6D_6 calibrated to internal standard, CF_3COOH in capillary tube.

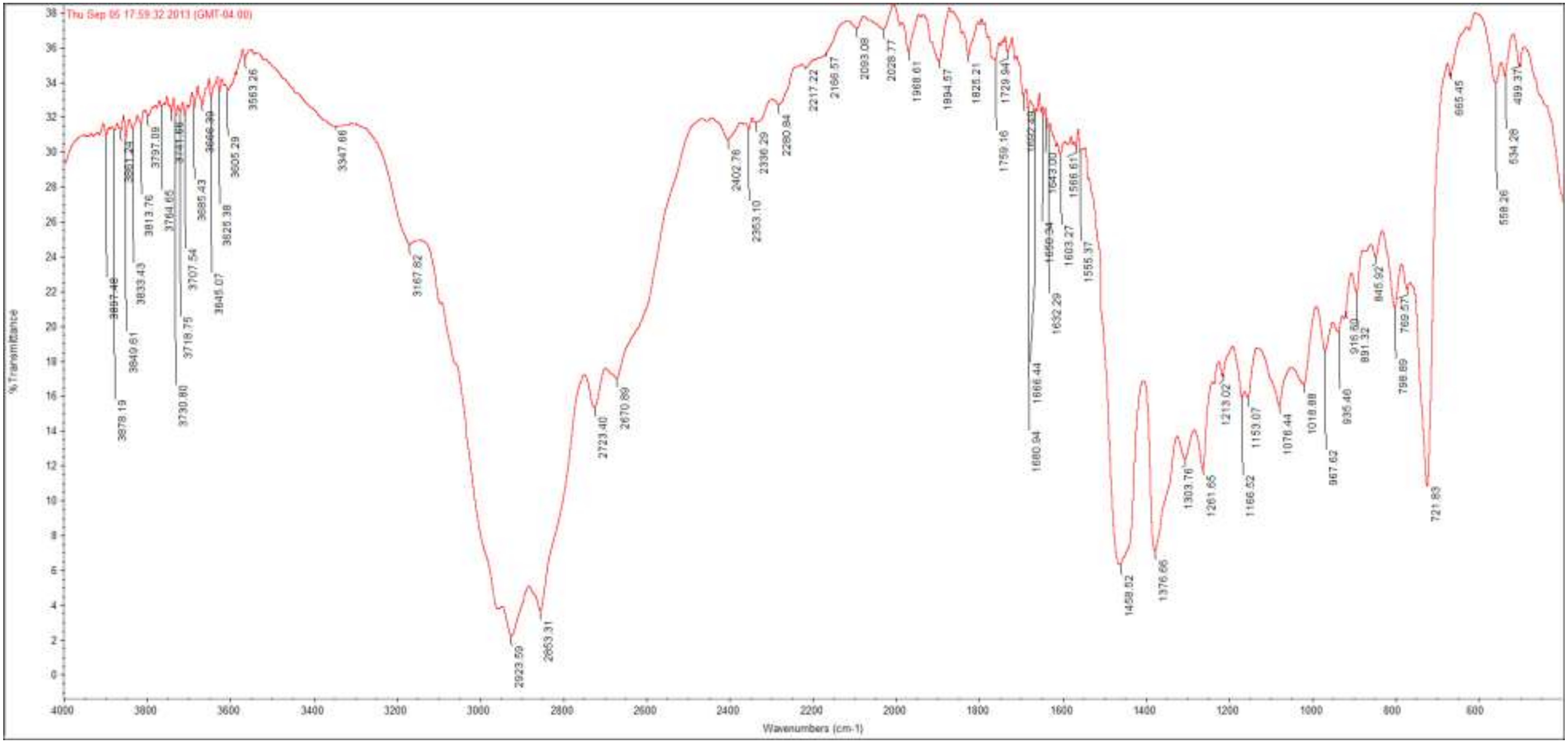

Figure S12. IR [Mg(OC(CF_3)$_2$Ph)$_2$(thf)$_2$] (**1**). Nujol mull, NaCl plates, transmission.

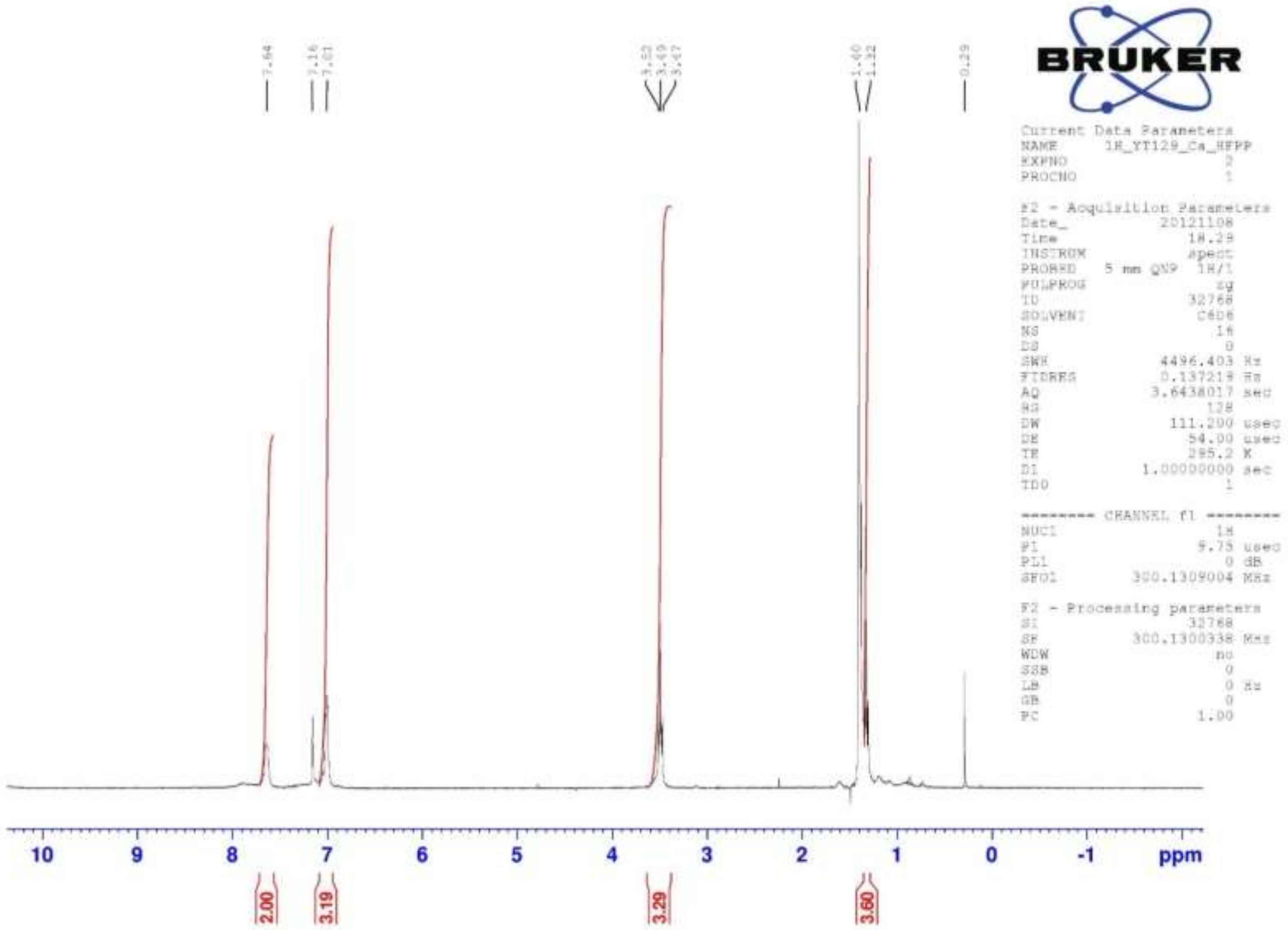

Figure S13. ^{1}H NMR [*trans*-Ca(OC(CF_3)$_2$Ph)$_2$(thf)$_4$] (**2**). 300 MHz, Solvent peak C_6D_6 at 7.16 ppm.

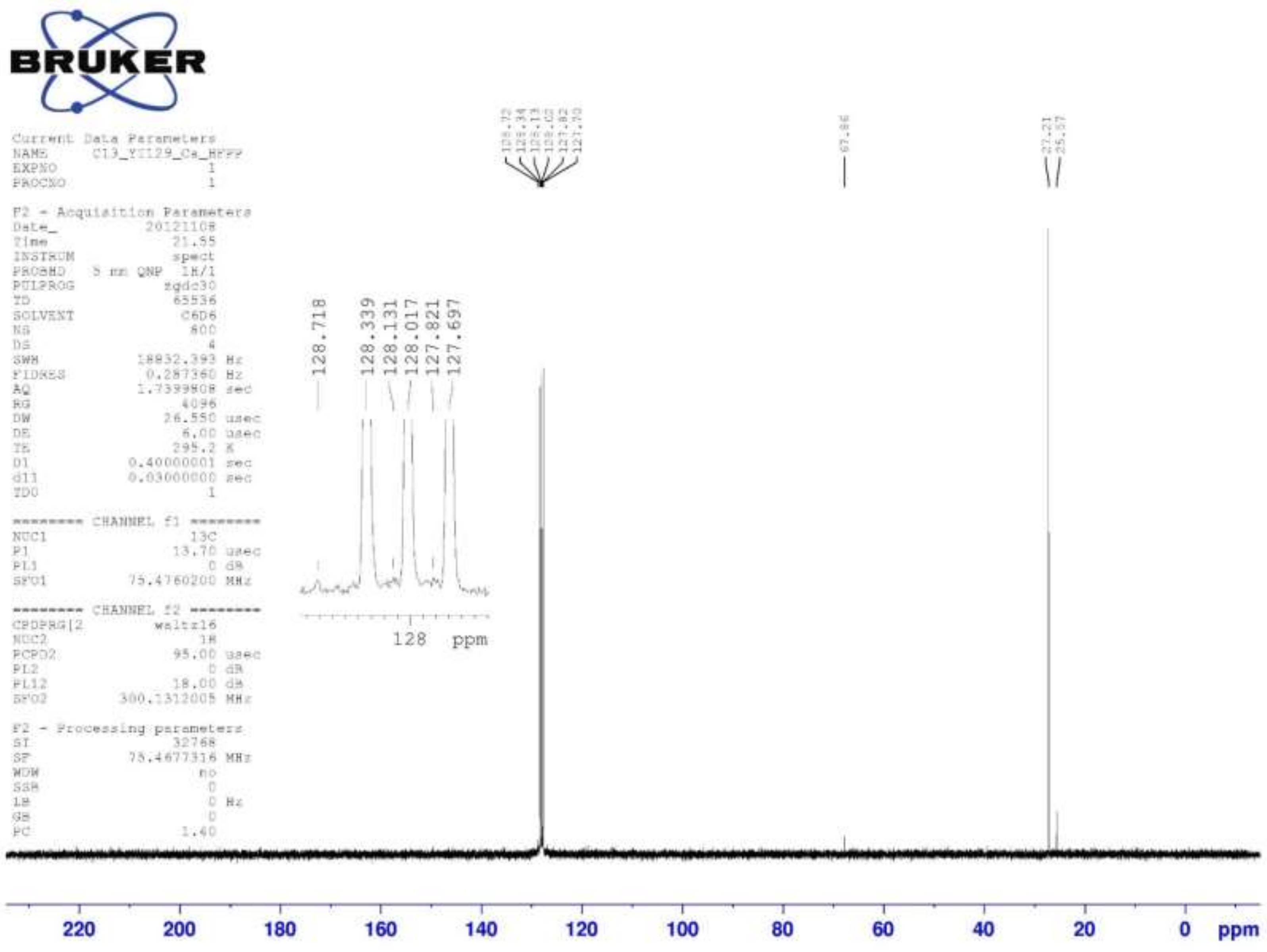

Figure S14. ^{13}C NMR [*trans*-Ca(OC(CF_3)$_2$Ph)$_2$(thf)$_4$] (**2**). 75 MHz, Solvent peak C_6D_6 at 128.02 ppm.

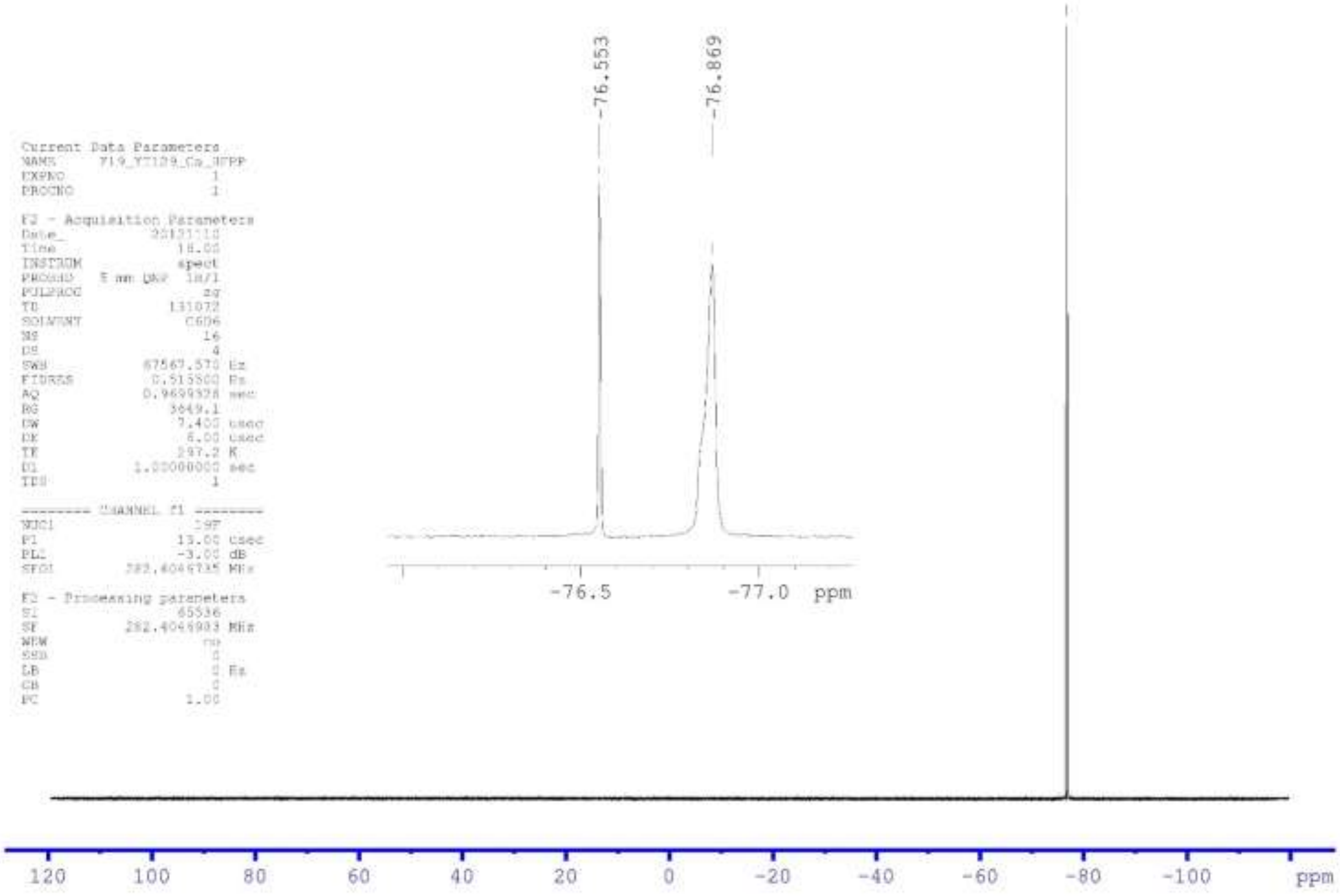

Figure S15. ^{19}F NMR [*trans*-$Ca(OC(CF_3)_2Ph)_2(thf)_4$] (**2**). in C_6D_6 calibrated to internal standard, CF_3COOH in capillary tube.

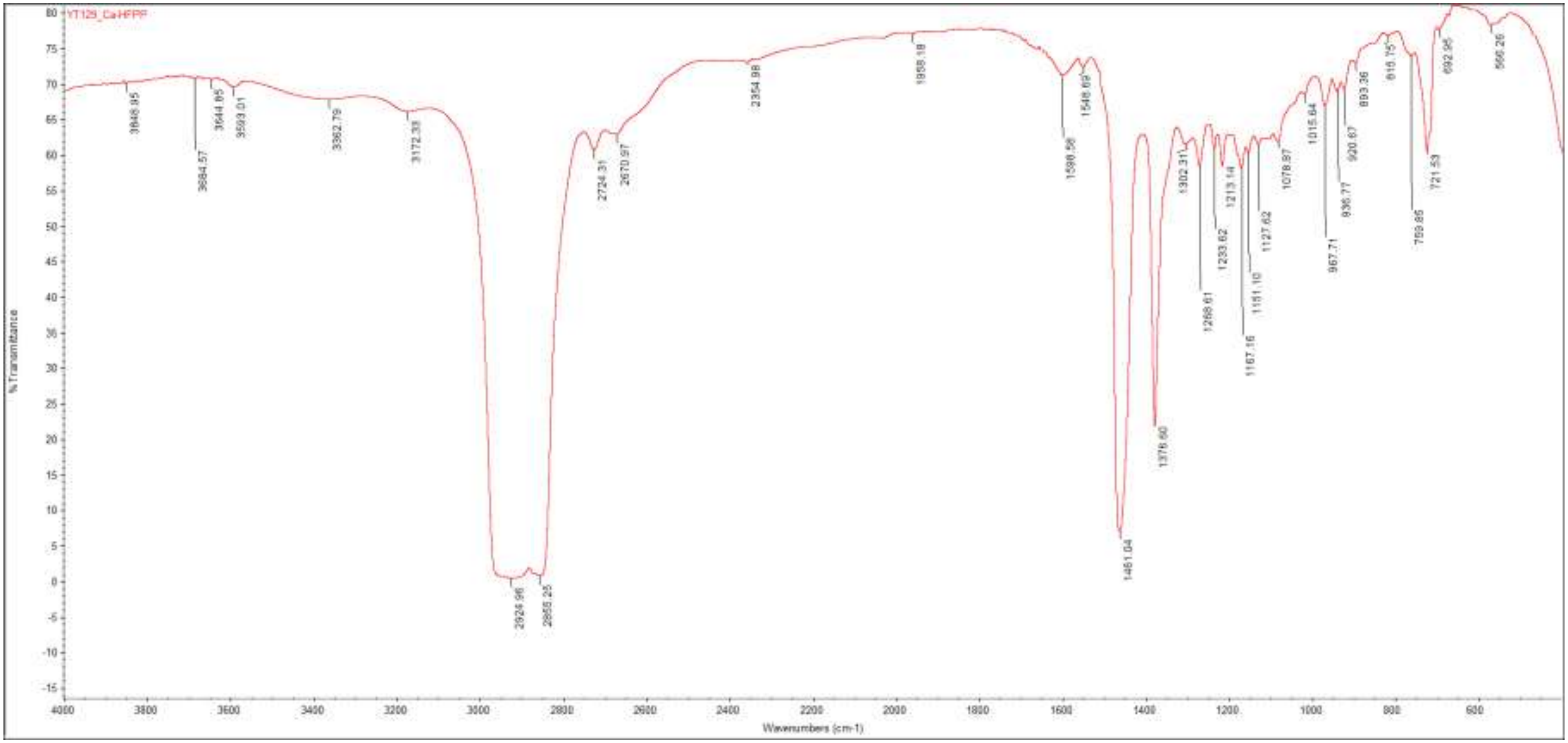

Figure S16. IR [*trans*-Ca(OC(CF_3)$_2$Ph)$_2$(thf)$_4$] (**2**). Nujol mull, NaCl plates, transmission

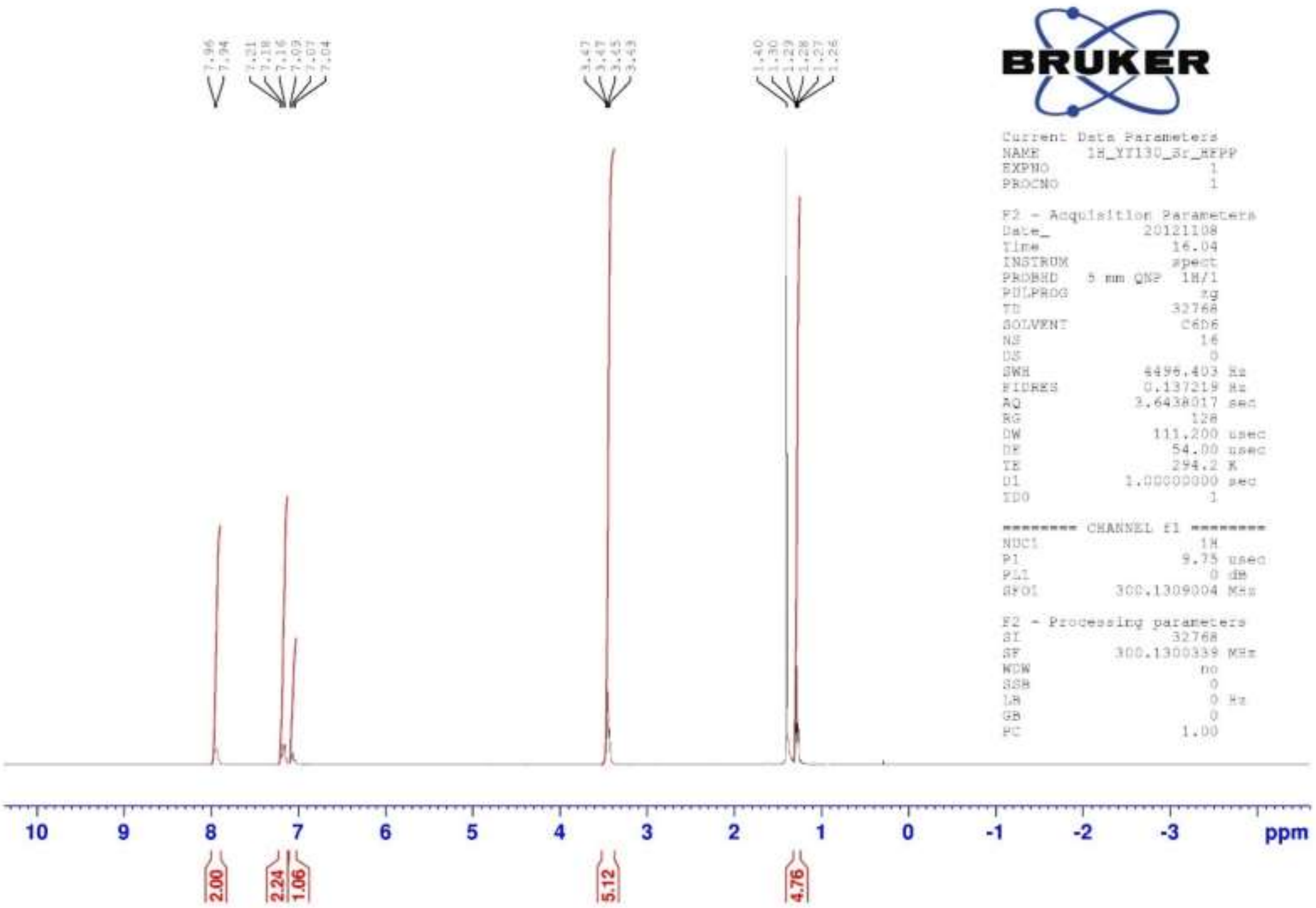

Figure S17. ^{1}H NMR [*cis*-Sr(OC(CF_3)$_2$Ph)$_2$(thf)$_4$] (**3**). 300 MHz, Solvent peak C_6D_6 at 7.16 ppm.

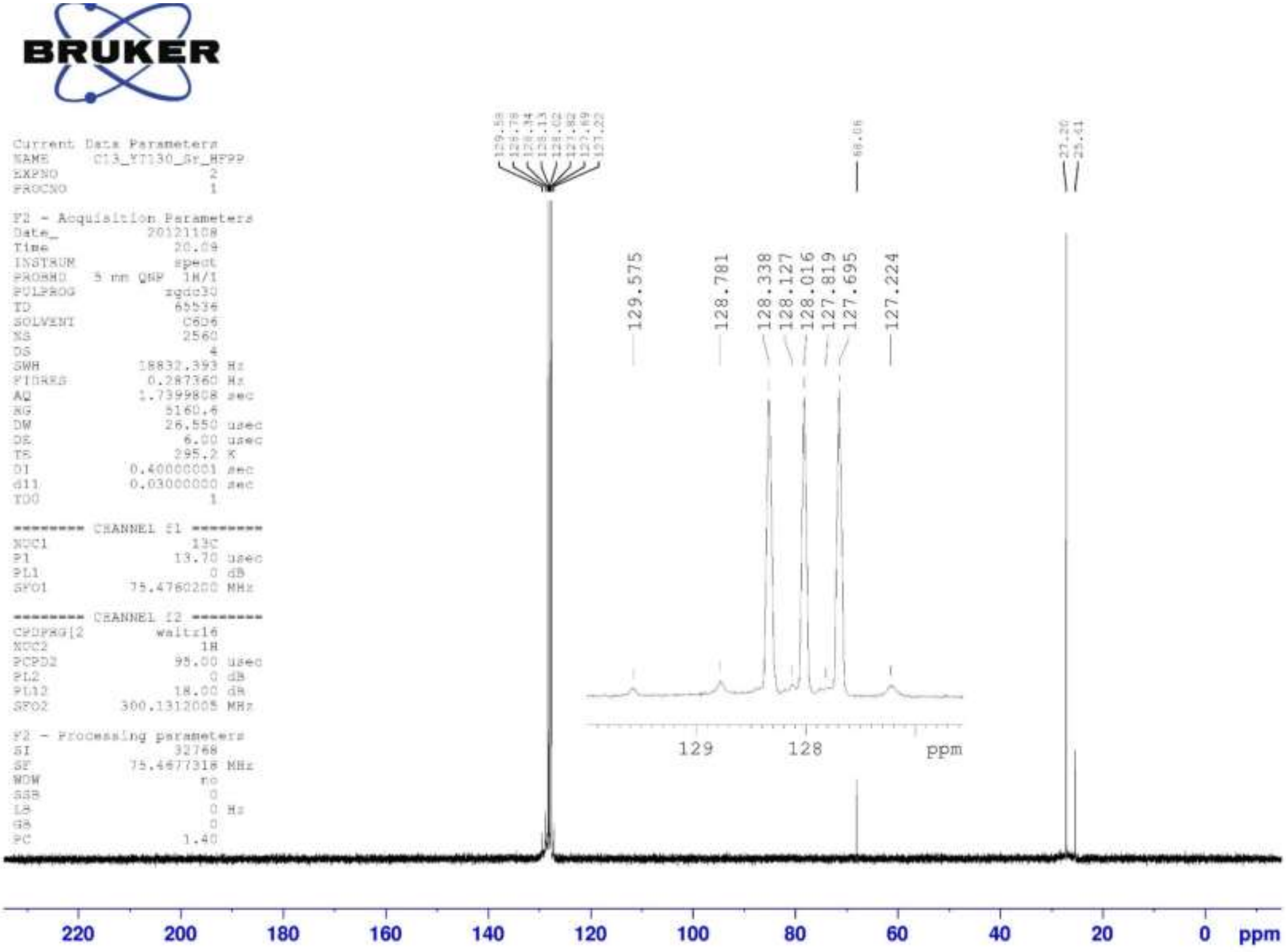

Figure S18. ^{13}C NMR [*cis*-$Sr(OC(CF_3)_2Ph)_2(thf)_4$] (**3**). 75 MHz, Solvent peak C_6D_6 at 128.02 ppm.

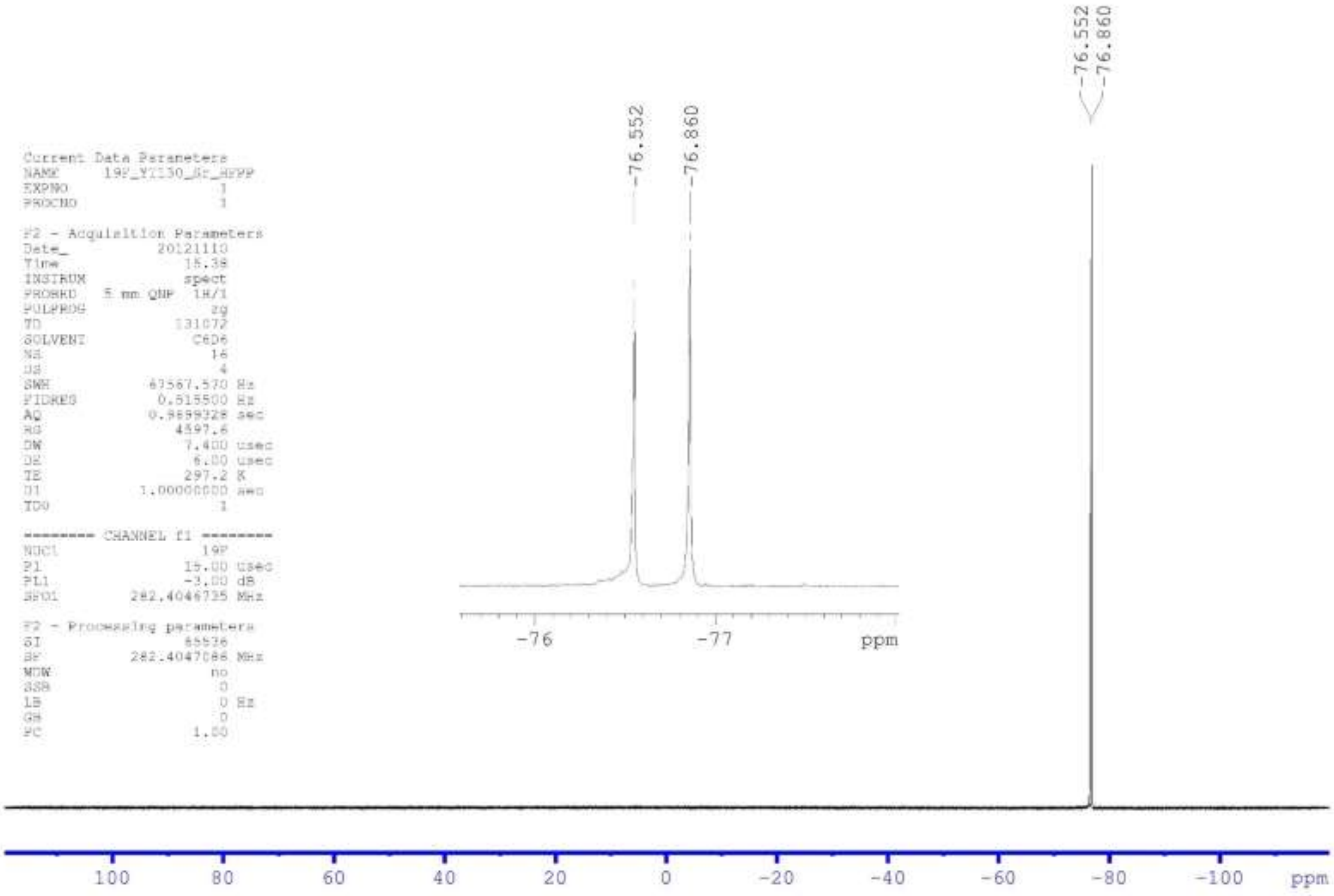

Figure S19. ^{19}F NMR [*cis*-Sr(OC(CF_3)$_2$Ph)$_2$(thf)$_4$] (**3**). in C_6D_6 calibrated to internal standard, CF_3COOH in capillary tube.

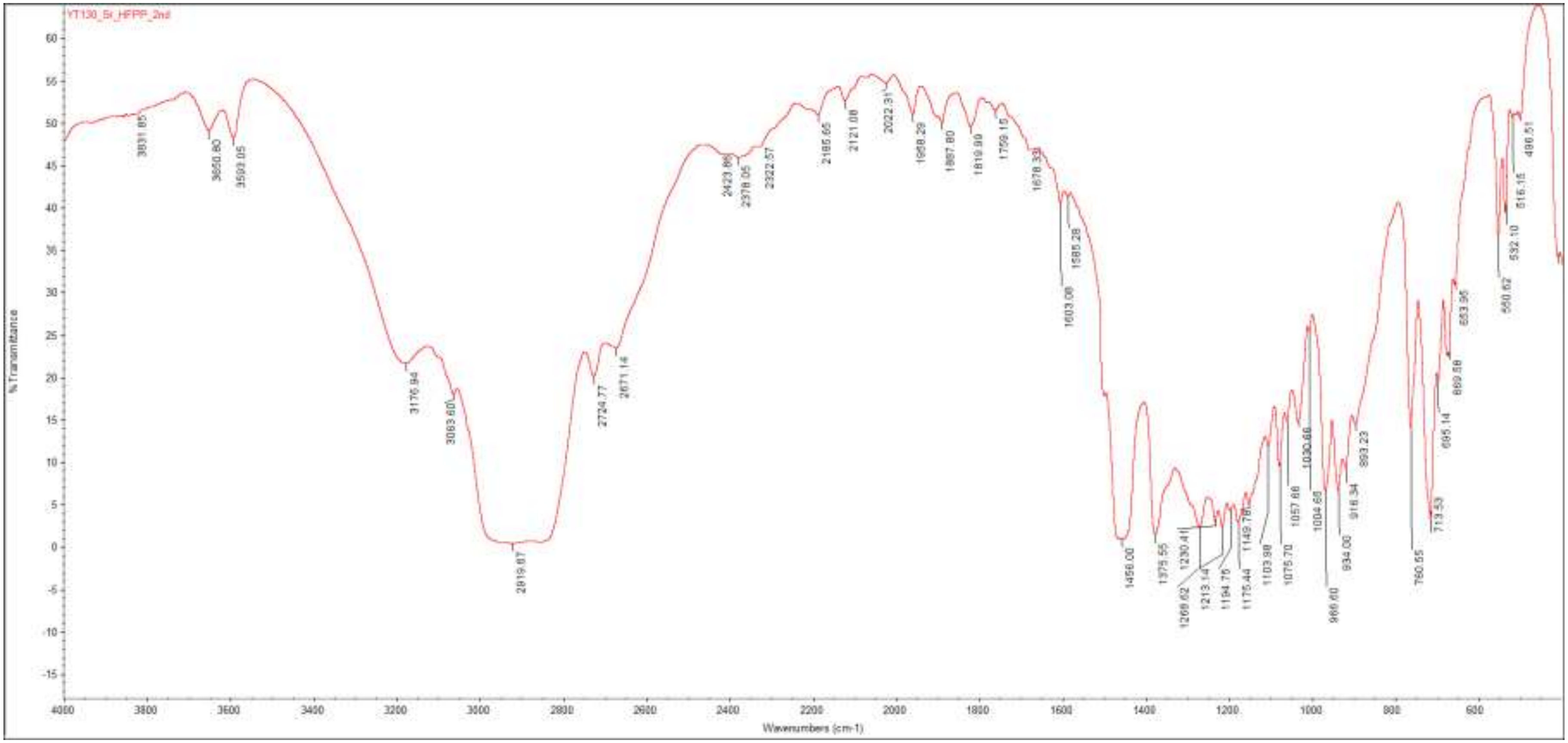

Figure S20. IR [*cis*-Sr(OC(CF$_3$)$_2$Ph)$_2$(thf)$_4$] (**3**). Nujol mull, NaCl plates, transmission

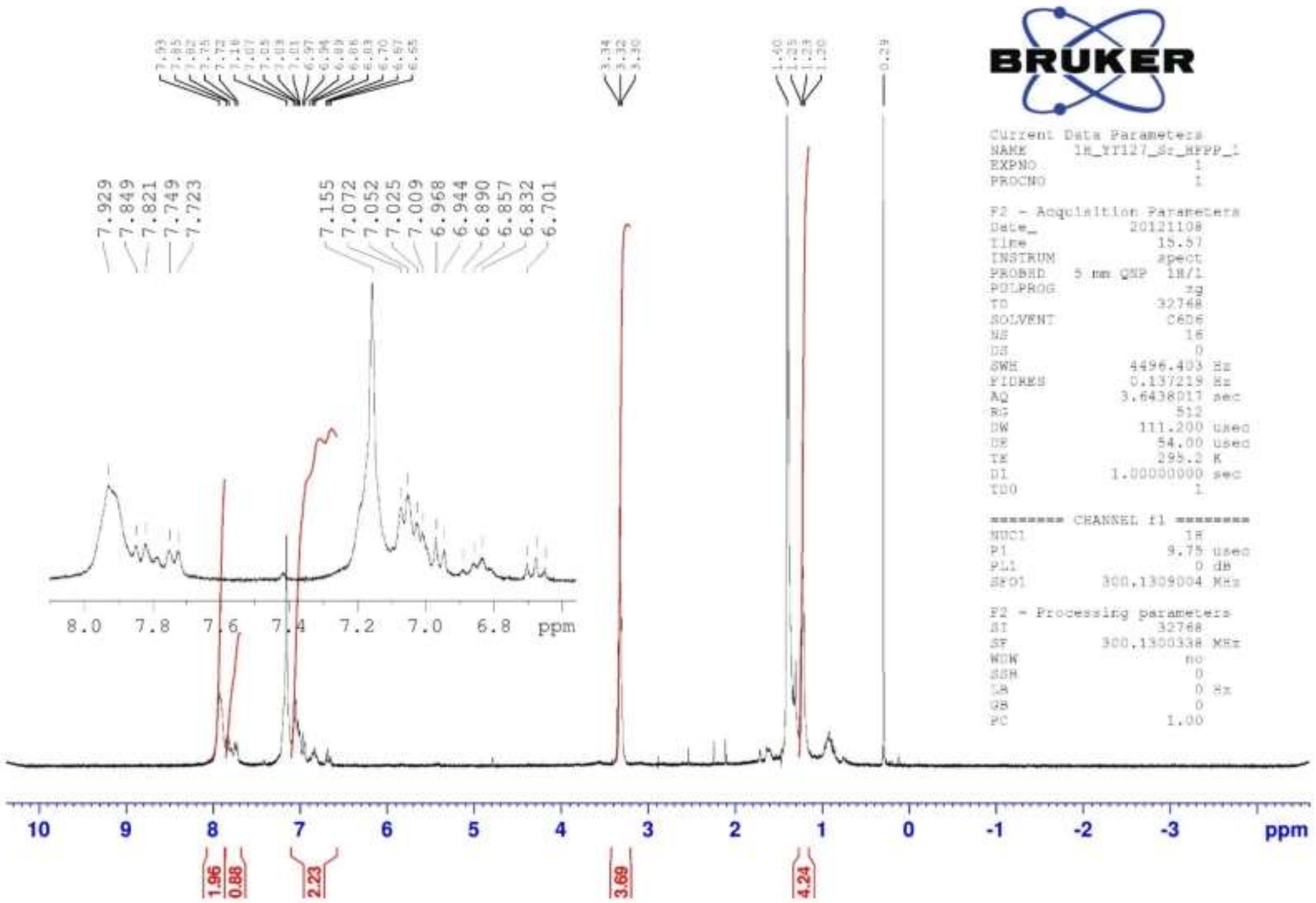

Figure S21. ^{1}H NMR [$Sr_2(\mu^2$-$OC(CF_3)_2Ph)_3(OC(CF_3)_2Ph)(thf)_3$] (**4**). 300 MHz, Solvent peak C_6D_6 at 7.16 ppm.

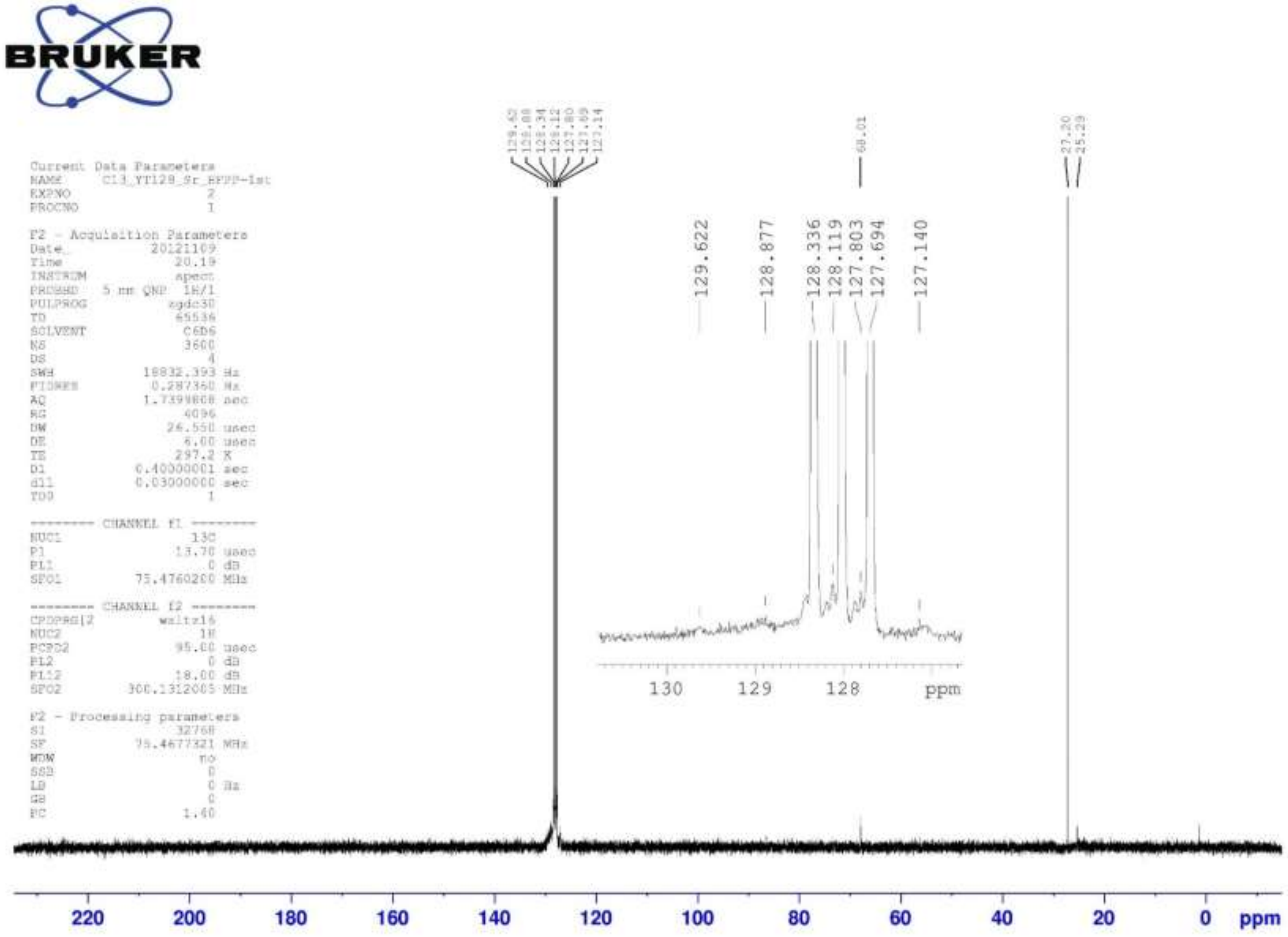

Figure S22. ^{13}C NMR [$Sr_2(\mu^2\text{-}OC(CF_3)_2Ph)_3(OC(CF_3)_2Ph)(thf)_3$] (**4**). 75 MHz, Solvent peak C_6D_6 at 128.02 ppm.

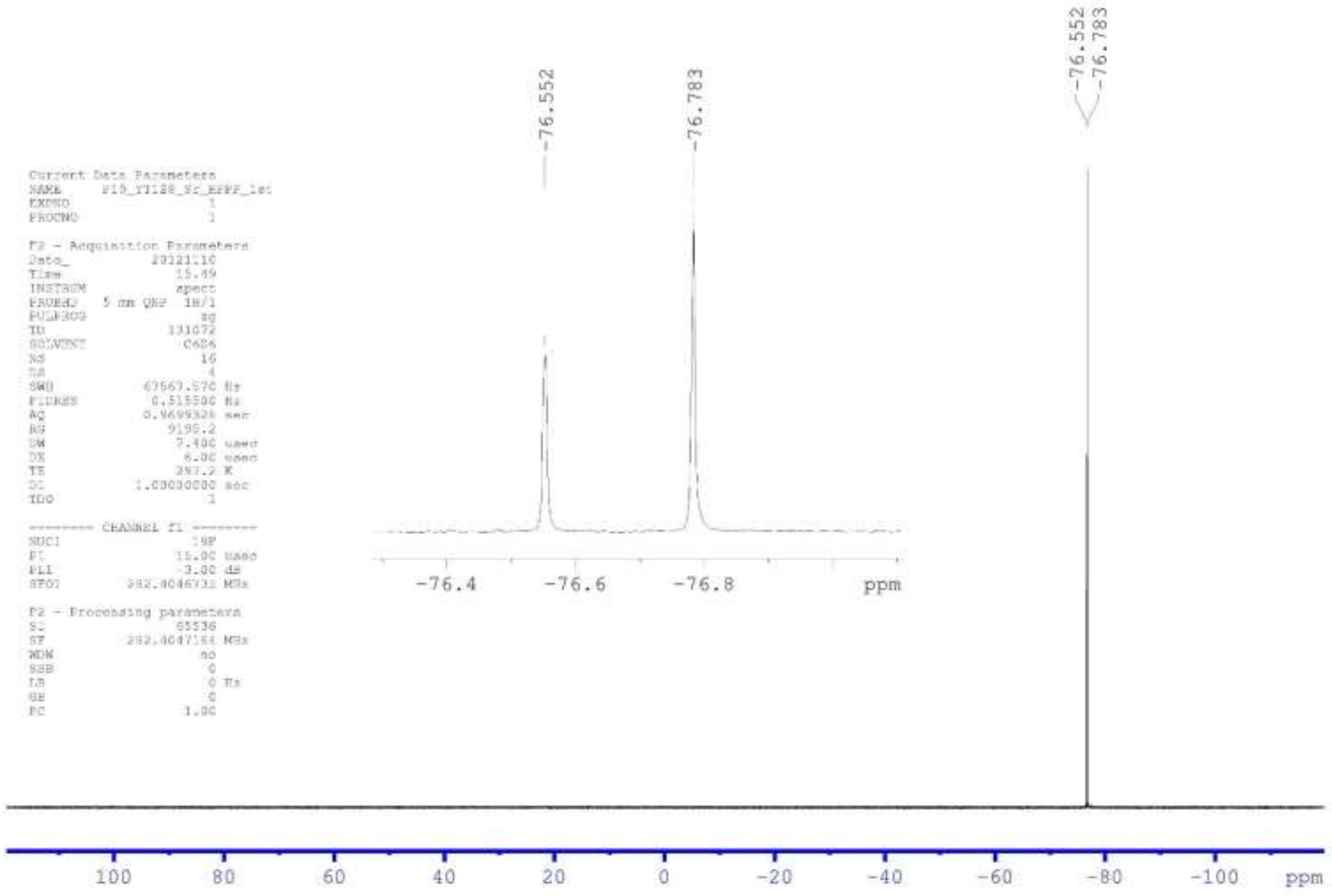

Figure S23. ^{19}F NMR $[Sr_2(\mu^2\text{-}OC(CF_3)_2Ph)_3(OC(CF_3)_2Ph)(thf)_3]$ (**4**). in C_6D_6 calibrated to internal standard, CF_3COOH in capillary tube.

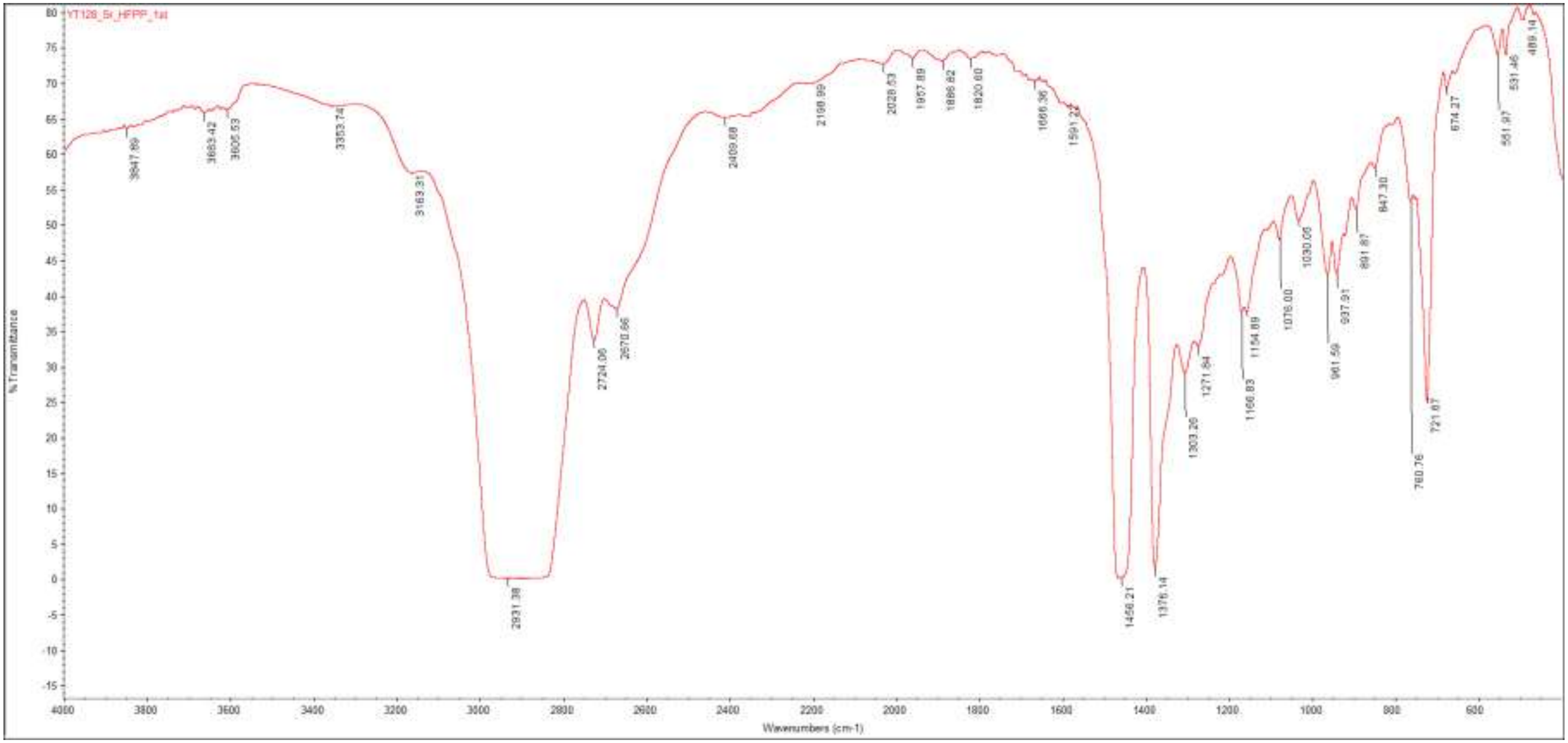

Figure S24. IR [$Sr_2(\mu^2-OC(CF_3)_2Ph)_3(OC(CF_3)_2Ph)(thf)_3$] (**4**). Nujol mull, NaCl plates, transmission

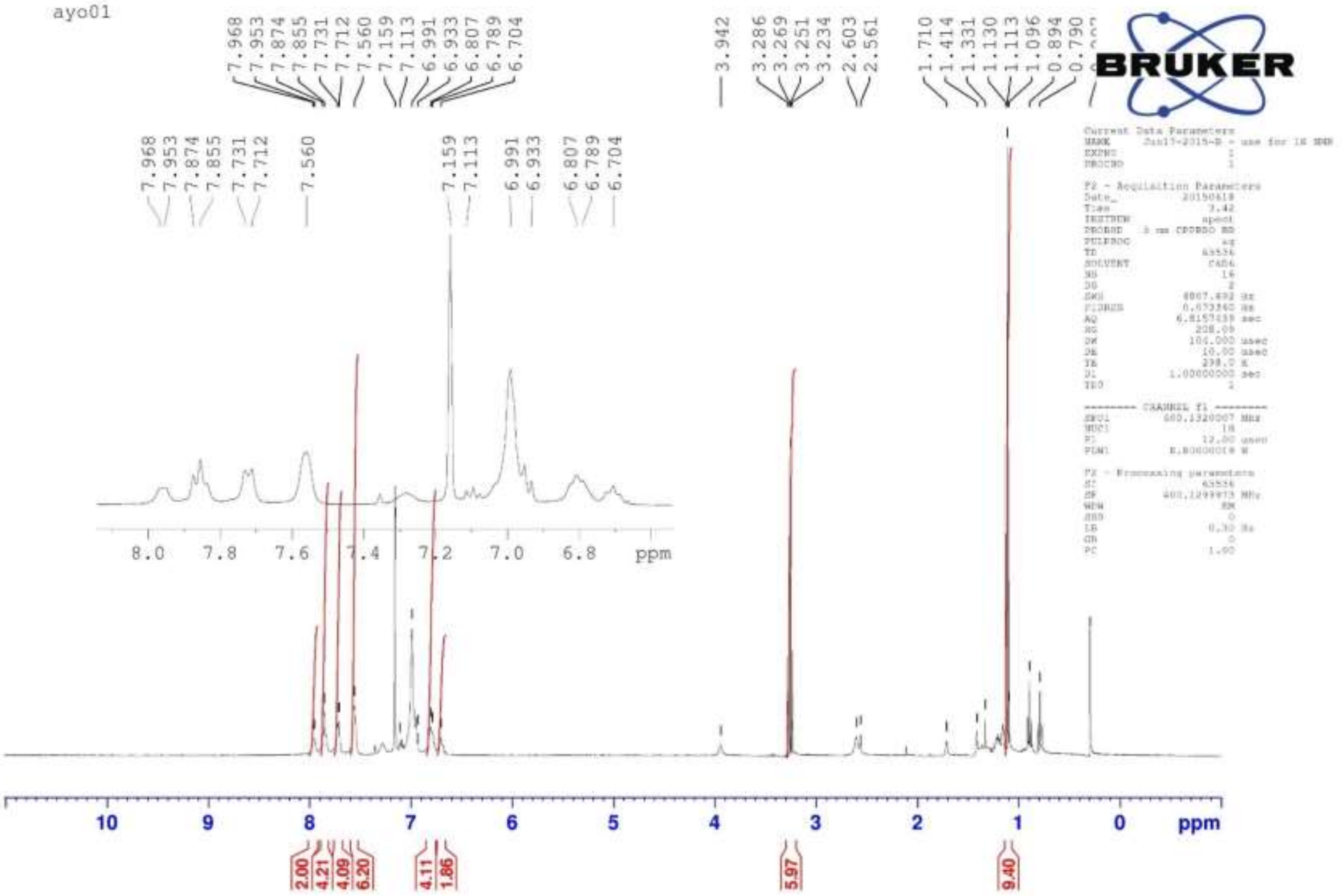

Figure S25. ^{1}H NMR [$Sr_3(\mu^2$-$(OC(CF_3)_2Ph)_4(OC(CF_3)_2Ph)_2(OEt_2)_2$] (**5**). 400 MHz, Solvent peak C_6D_6 at 7.16 ppm.

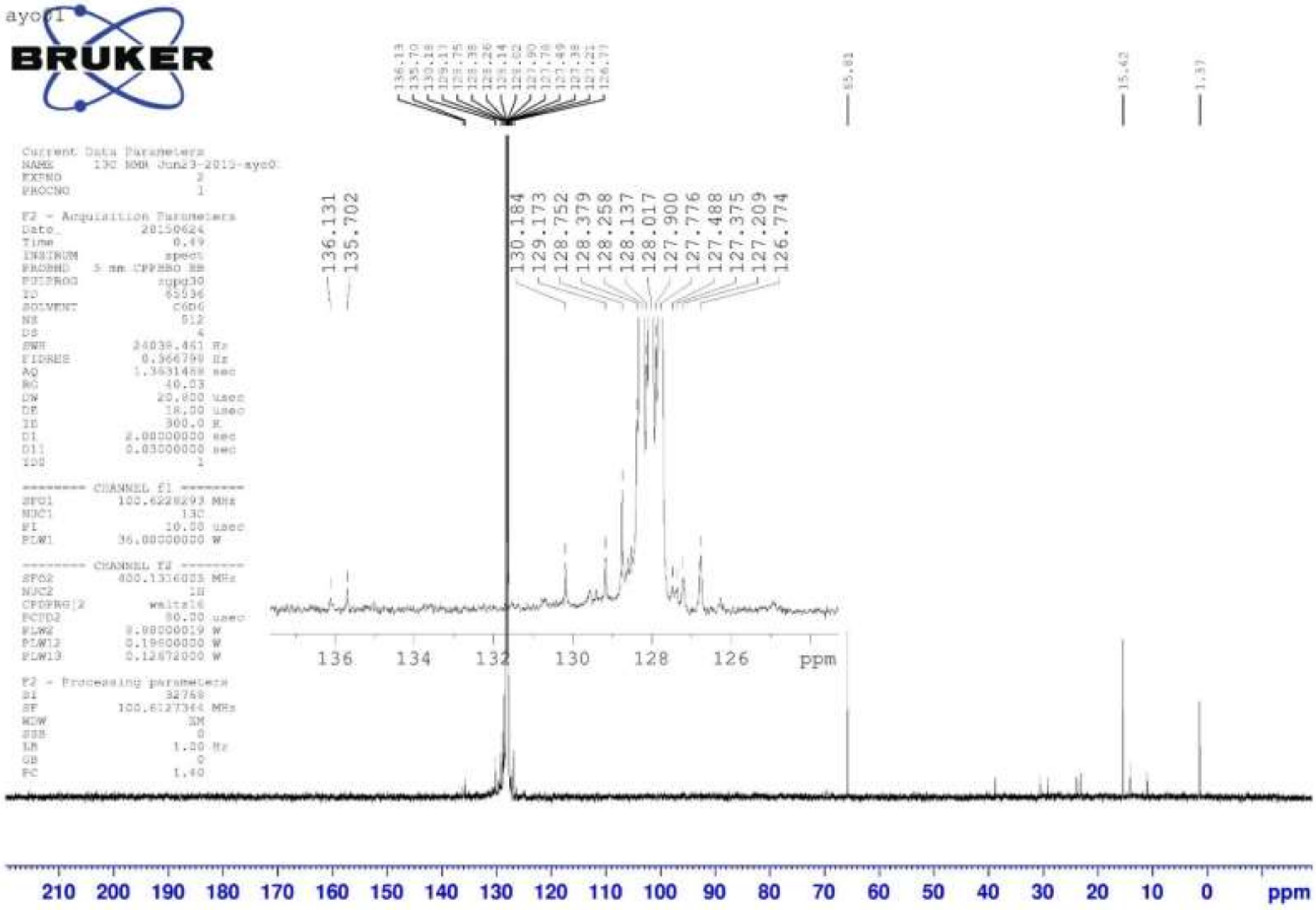

Figure S26. ^{13}C NMR [$Sr_3(\mu^2$-$(OC(CF_3)_2Ph)_4(OC(CF_3)_2Ph)_2(OEt_2)_2$] (**5**). 100 MHz, Solvent peak C_6D_6 at 128.02 ppm.

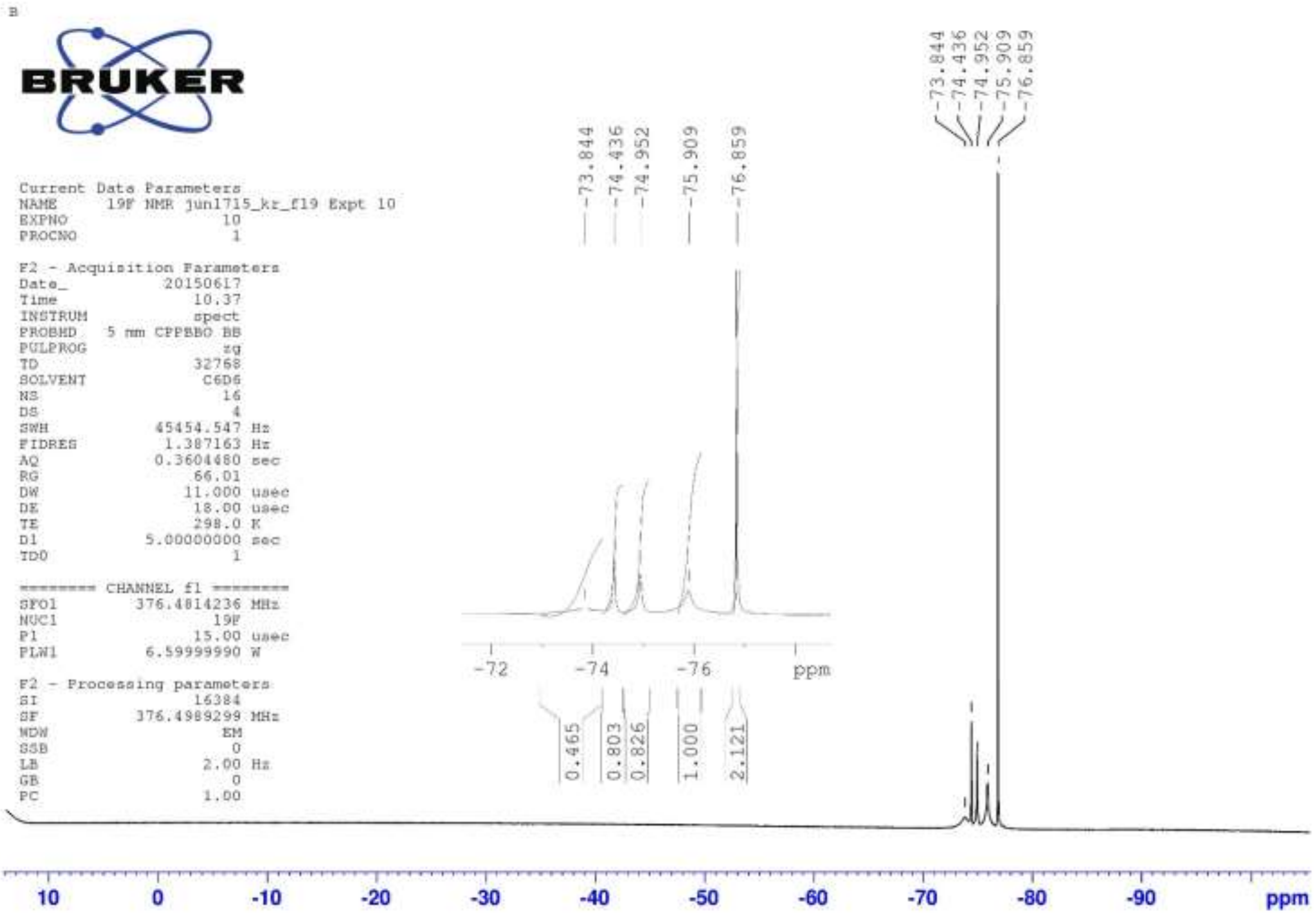

Figure S27. ^{19}F NMR $[Sr_3(\mu^2\text{-}(OC(CF_3)_2Ph)_4(OC(CF_3)_2Ph)_2(OEt_2)_2]$ (**5**). 376 MHz, manually calibrated to external standard, $C_6H_5CF_3$ in C_6D_6.

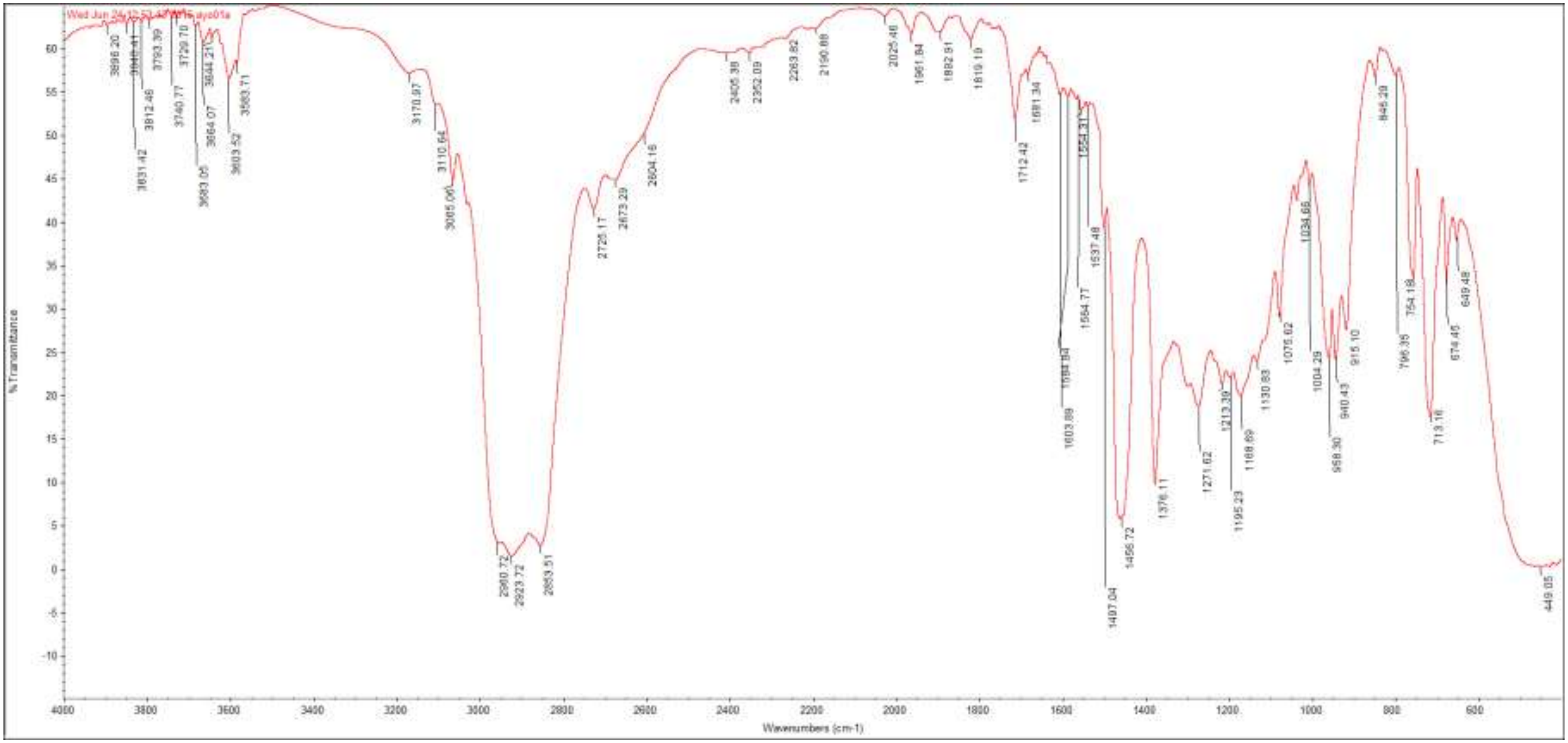

Figure S28. IR [$Sr_3(\mu^2\text{-}(OC(CF_3)_2Ph)_4(OC(CF_3)_2Ph)_2(OEt_2)_2$] (**5**). Nujol mull, NaCl plates, transmission

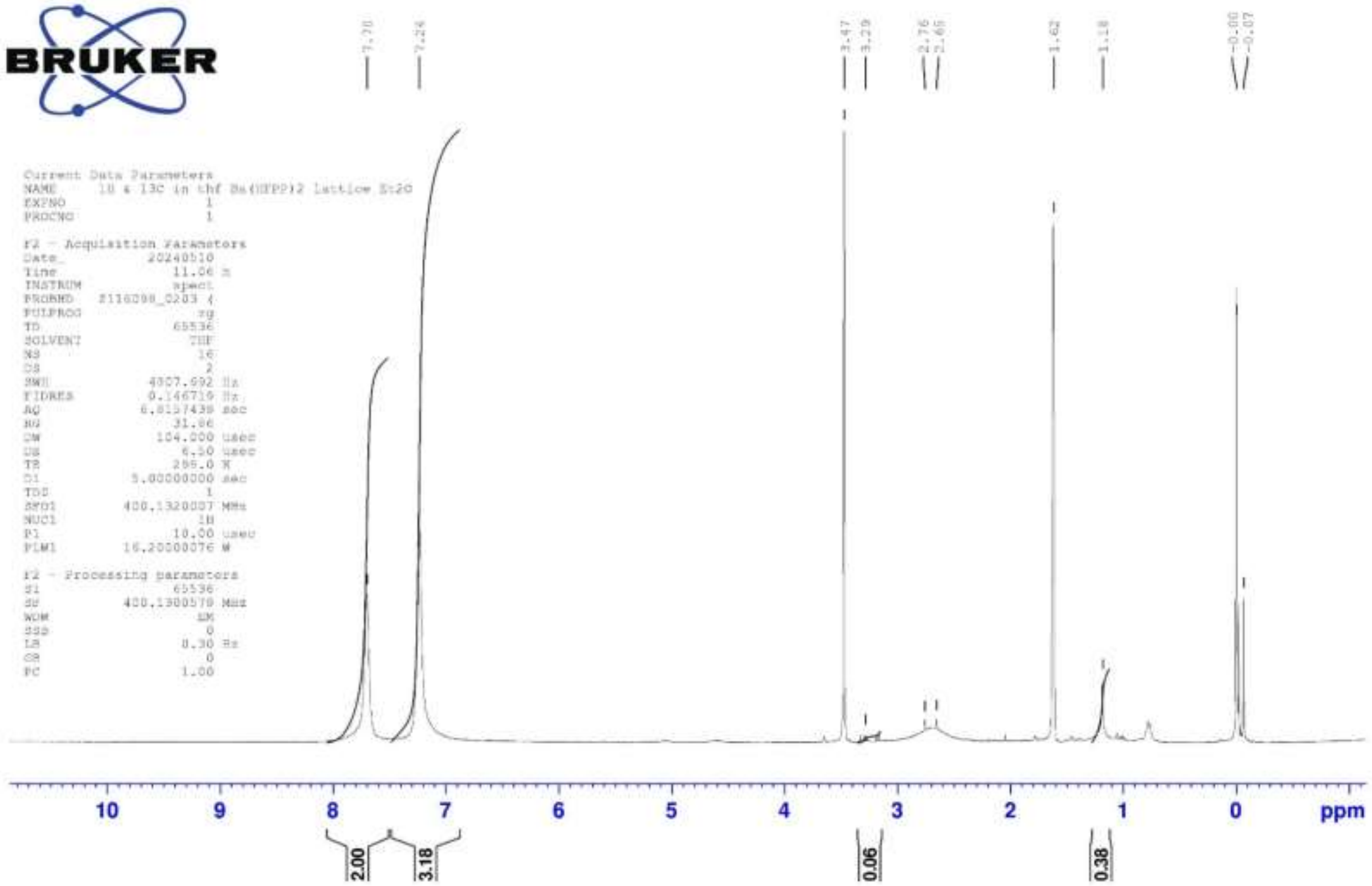

Figure S29. ^{1}H NMR $[Ba(\mu^2\text{-}OC(CF_3)_2Ph)_2]_n\cdot\frac{1}{4}(OEt_2)$ (**6**). 400 MHz, TMS 0.00ppm, d_8-THF solvent 3.47, 1.62 ppm.

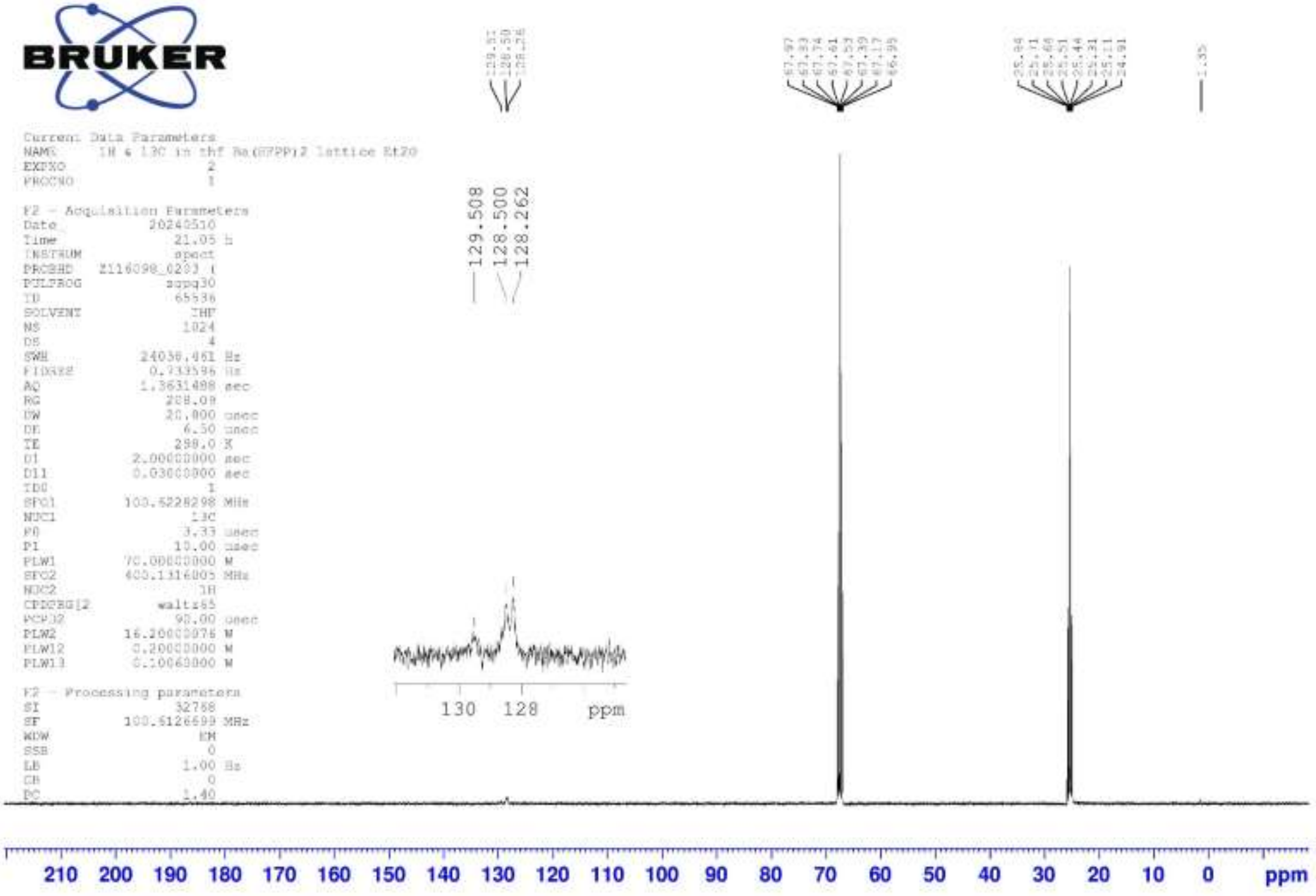

Figure S30. ^{13}C NMR [Ba(μ^2-OC(CF_3)$_2$Ph)$_2$]$_n$·$\frac{1}{4}$(OEt_2) (**6**). 100 MHz, Solvent peaks THF at 67.6 and 25.5 ppm.

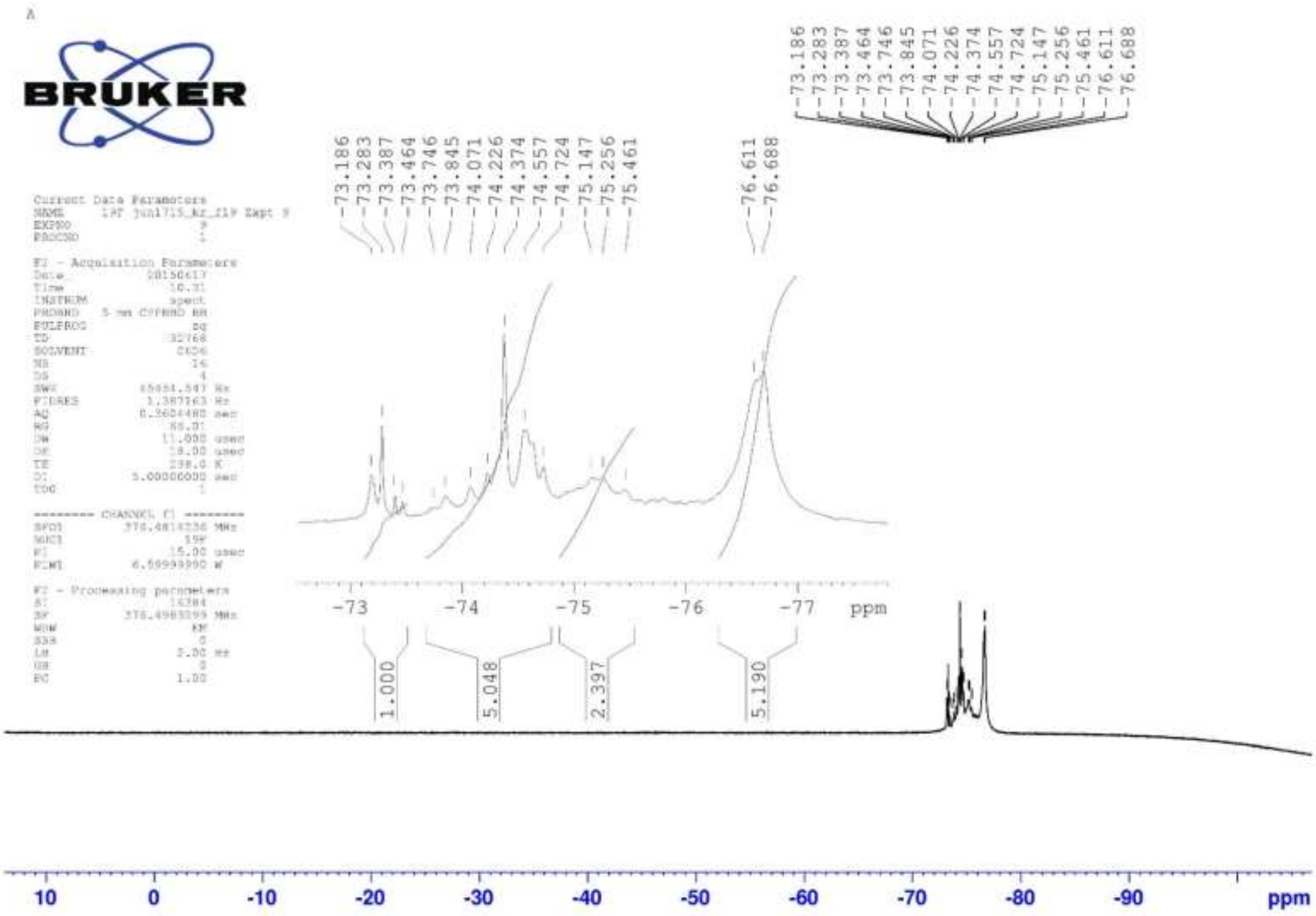

Figure S31. ^{19}F NMR $[Ba(\mu^2\text{-}OC(CF_3)_2Ph)_2]_n \cdot \frac{1}{4}(OEt_2)$ (**6**). 376 MHz in C_6D_6, manually calibrated to external standard, $C_6H_5CF_3$ in C_6D_6.

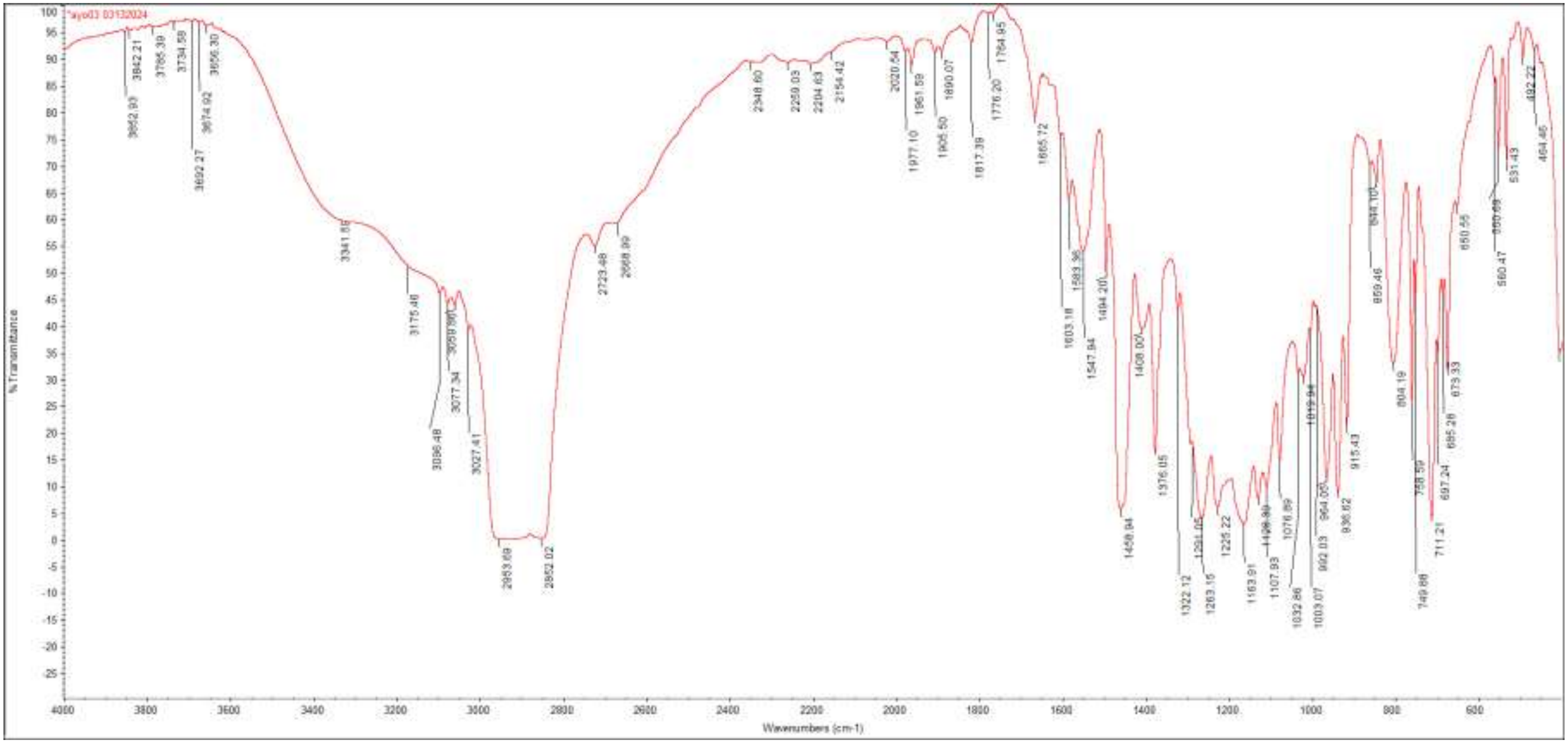

Figure S32. IR $[Ba(\mu^2\text{-}OC(CF_3)_2Ph)_2]_n \cdot \frac{1}{4}(OEt_2)$ (**6**). Nujol mull, NaCl plates, transmission

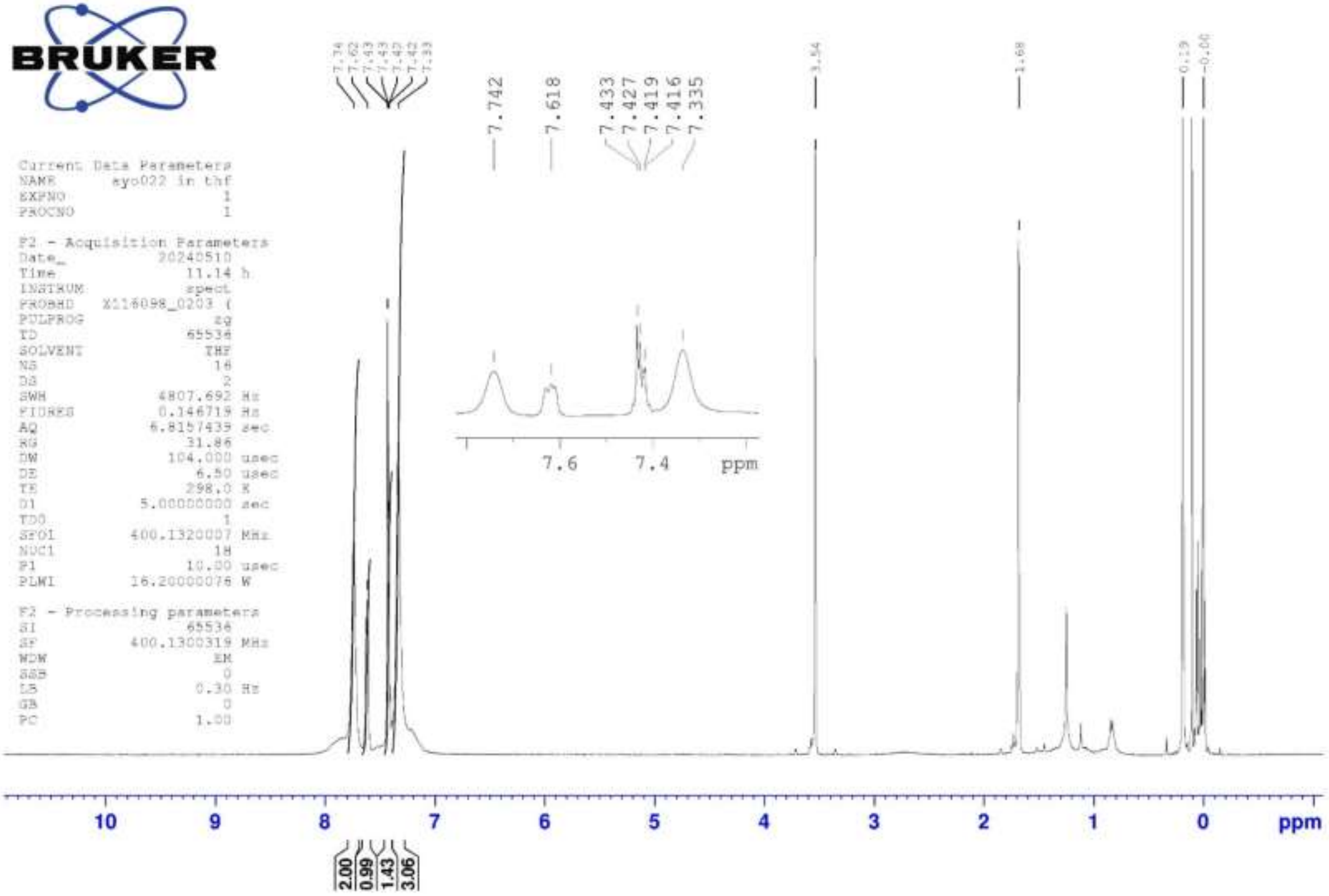

Figure S33. ^{1}H NMR [Ba(μ^2-OC(CF_3)$_2$Ph)$_2$]$_n$ (**7**). 400 MHz, TMS 0.00ppm, d_8-THF solvent 3.54, 1.68 ppm.

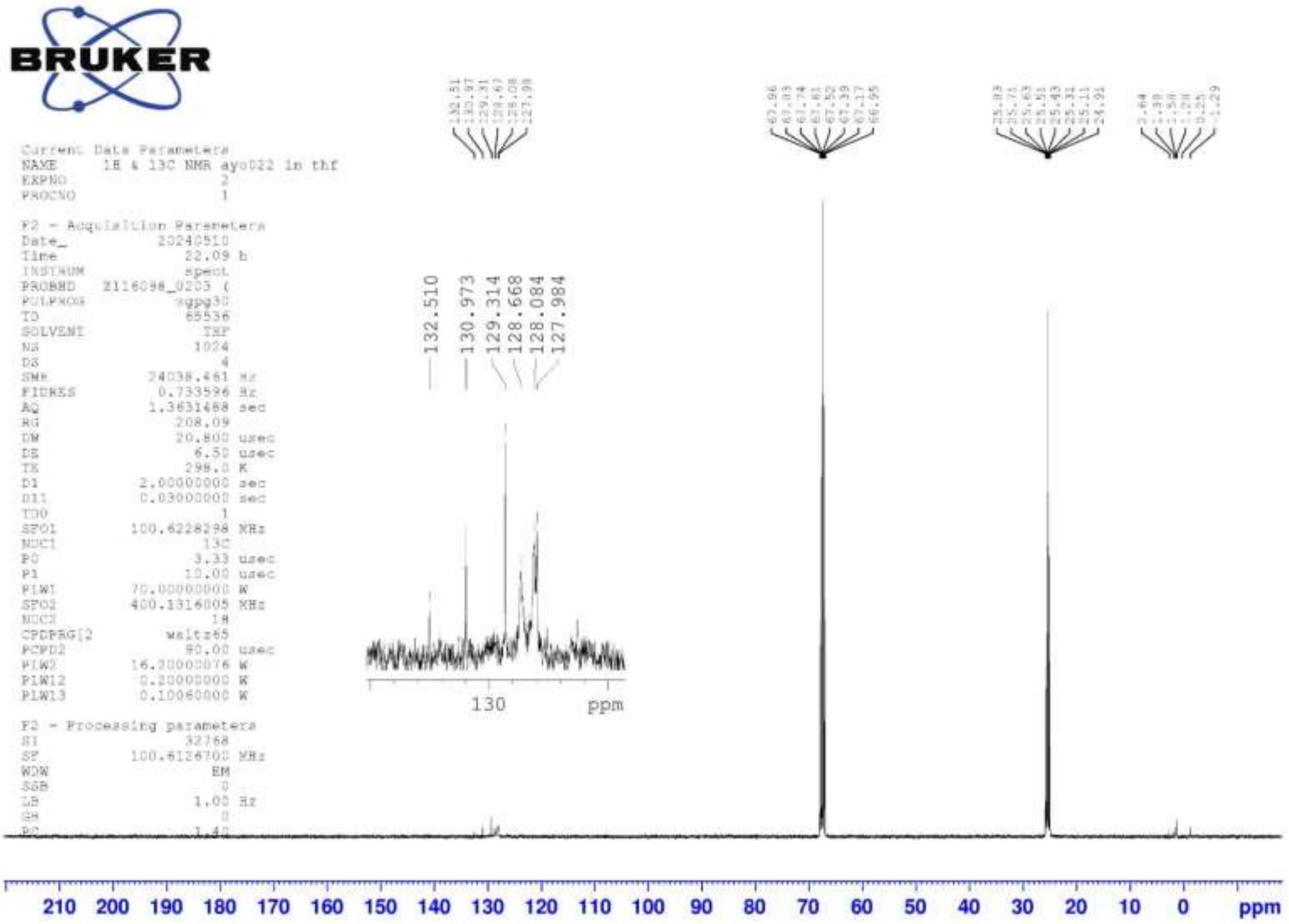

Figure S34. ^{13}C NMR $[Ba(\mu^2\text{-}OC(CF_3)_2Ph)_2]_n$ (**7**). 100 MHz, Solvent peaks THF at 67.6 and 25.5 ppm.

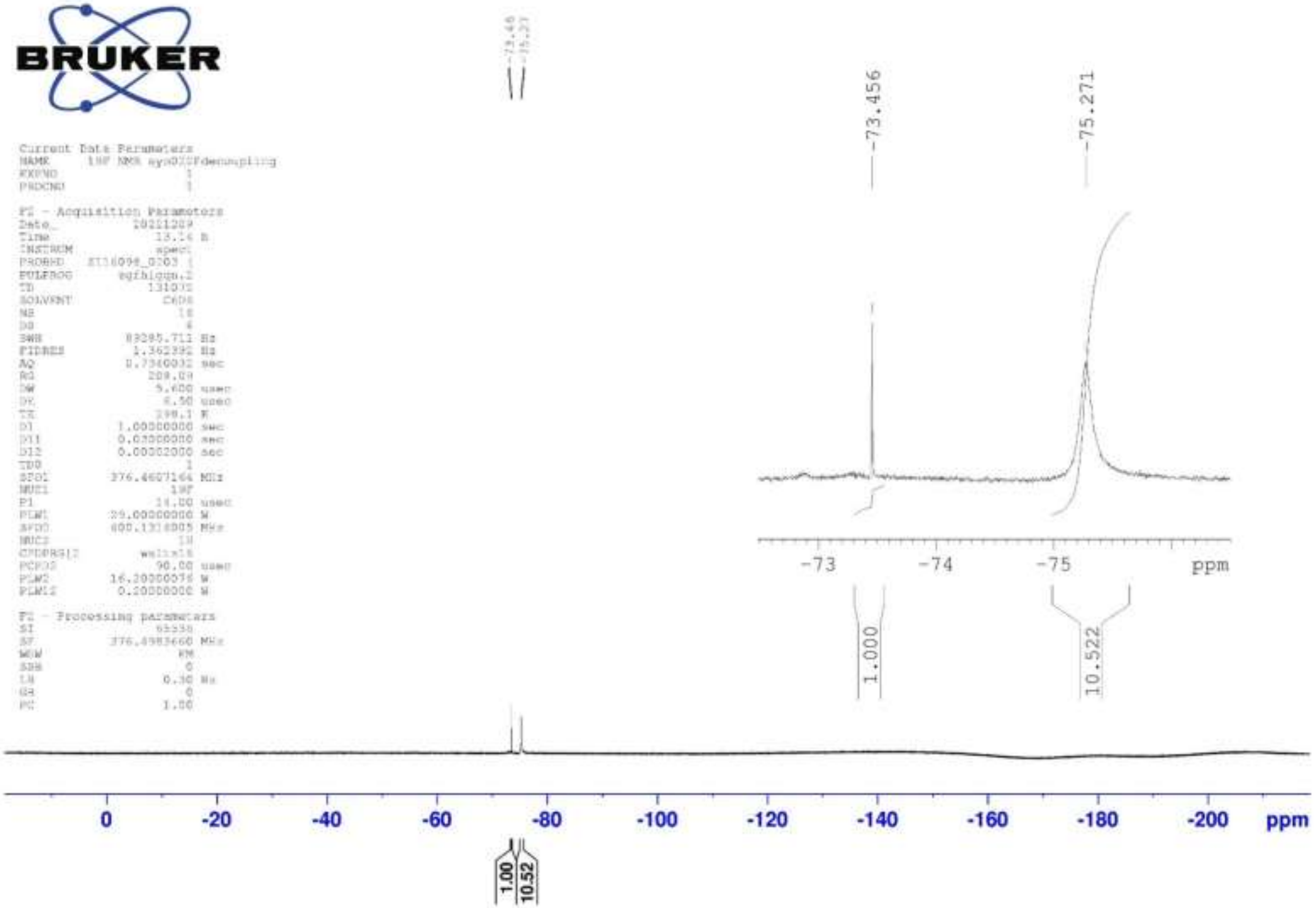

Figure S35. ^{19}F NMR $[Ba(\mu^2\text{-}OC(CF_3)_2Ph)_2]_n$ (**7**). 376 MHz in C_6D_6, manually calibrated to external standard, $C_6H_5CF_3$ in C_6D_6.

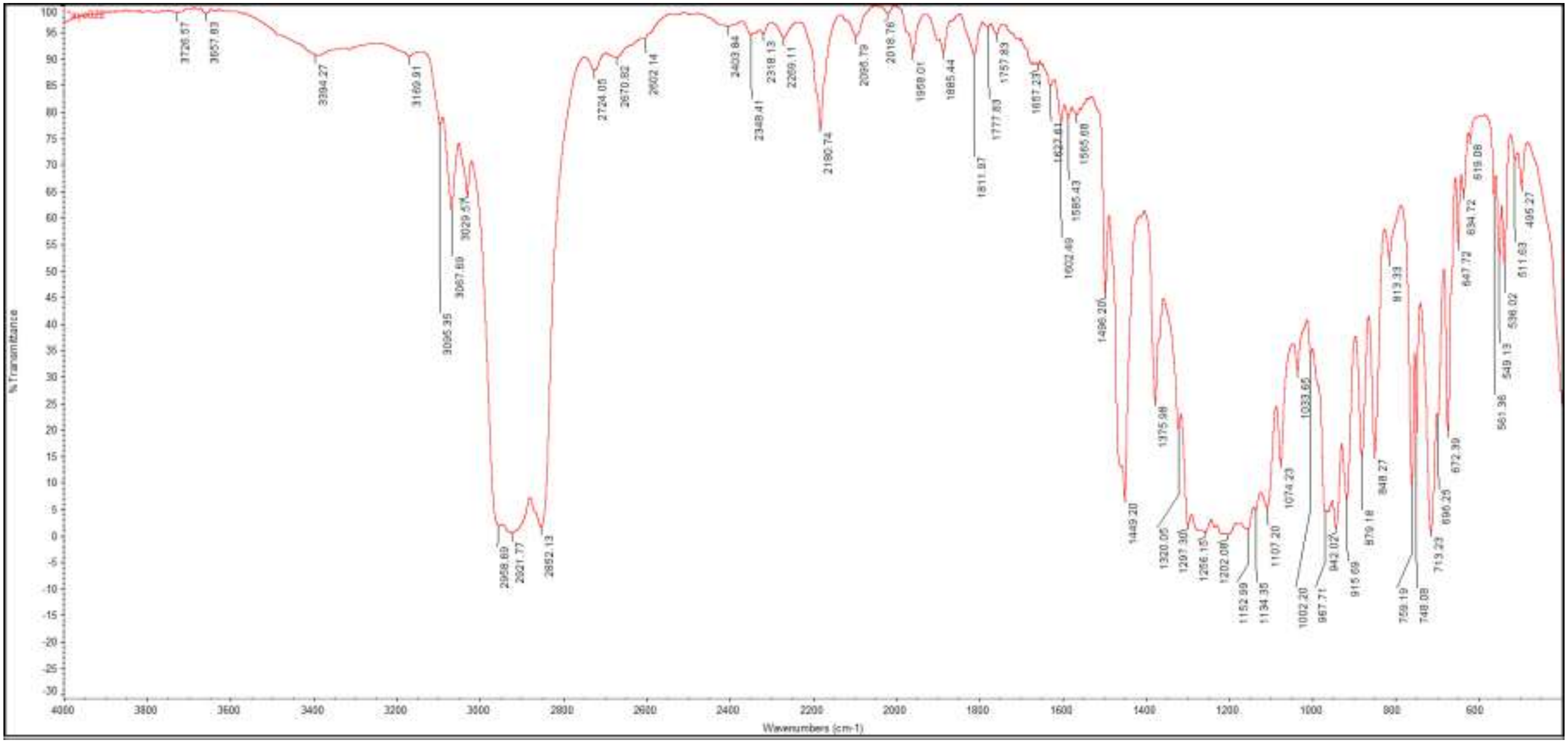

Figure S36. IR $[Ba(\mu^2\text{-}OC(CF_3)_2Ph)_2]_n$ (**7**). Nujol mull, NaCl plates, transmission

TGA data

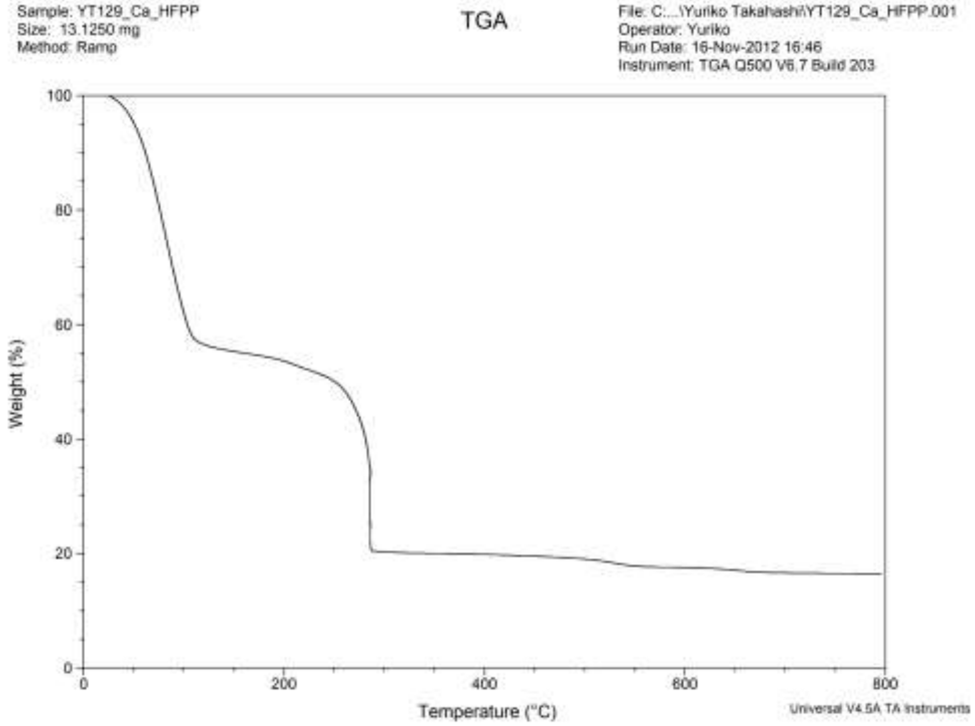

Figure S37. Thermogravimetric plot of the remaining weight percent of [*trans*-Ca(OC(CF_3)$_2$Ph)$_2$(thf)$_4$] (**2**) over temperature increase to 800 ºC.

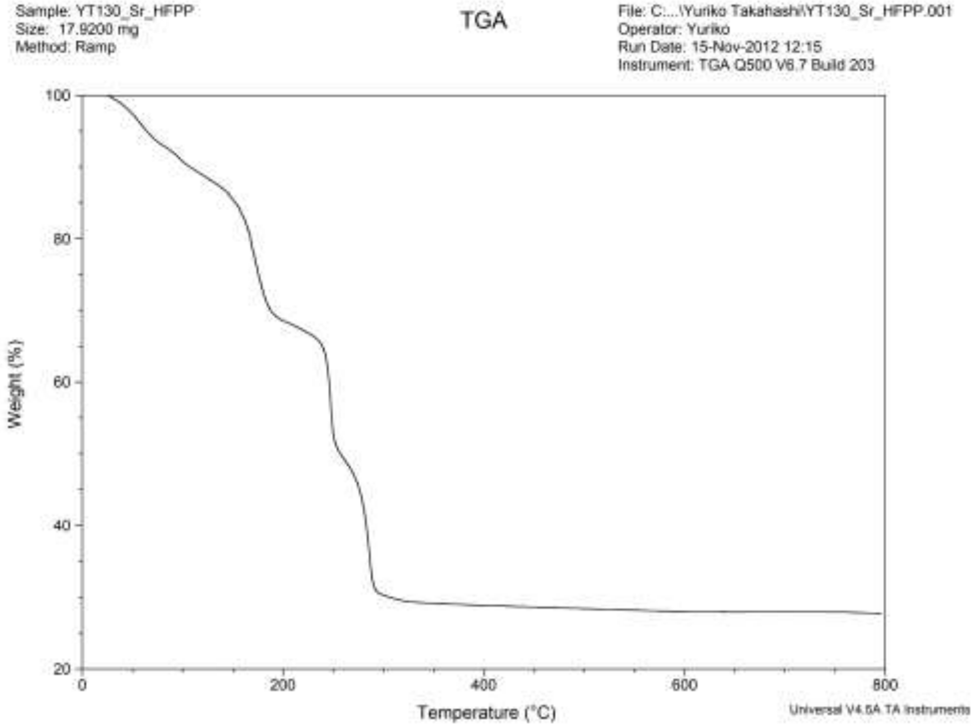

Figure S38. Thermogravimetric plot of the remaining weight percent of [*cis*-Sr(OC(CF_3)$_2$Ph)$_2$(thf)$_4$] (**3**) over temperature increase to 800 ºC.

Cartesian Coordinates.

Compounds 1-7. Formatting here places one structure on each page. The structure name is provided in the 2nd line of each .xyz file and are provided in order.

69
structure_1a.xyz

C	2.462462	0.973124	-0.528812
C	3.494836	1.791685	0.292014
C	2.255677	1.656377	-1.907956
C	2.950986	-0.457564	-0.790268
C	1.980470	-1.414727	-1.048114
C	2.325612	-2.732314	-1.276382
C	3.653258	-3.115734	-1.248868
C	4.627820	-2.166046	-1.009702
C	4.281811	-0.844537	-0.790824
C	-2.463249	-0.957122	-0.543682
C	-3.497588	-1.776469	0.273991
C	-2.260054	-1.631860	-1.927157
C	-2.947515	0.476789	-0.794334
C	-4.277797	0.865283	-0.801597
C	-4.619885	2.190646	-1.002328
C	-3.641753	3.142459	-1.217308
C	-2.314528	2.757260	-1.240801
C	-1.973453	1.435728	-1.030523
C	1.993081	-1.299295	2.936903
C	2.831395	-2.446248	2.433367
C	1.804003	-3.565465	2.325409
C	0.508608	-2.843902	1.975123
C	-0.513381	2.804950	2.041607
C	-1.833056	3.500999	2.356538
C	-2.836077	2.359918	2.474653
C	-1.973979	1.234680	2.987787
F	2.976091	2.943167	0.719623
F	4.618090	2.097434	-0.376189
F	3.862768	1.105691	1.388102
F	1.940870	2.952439	-1.785282
F	3.332666	1.580511	-2.700021
F	1.245018	1.068721	-2.556357
F	-2.981873	-2.931252	0.696712
F	-4.622030	-2.076630	-0.394747
F	-3.862800	-1.093634	1.372922
F	-1.248119	-1.042740	-2.572300
F	-1.949452	-2.929735	-1.813591
F	-3.337500	-1.547324	-2.717843
Mg	0.001992	-0.007295	1.022412
O	1.299423	1.017167	0.146754
O	-1.299933	-1.008998	0.130292
O	0.756510	-1.441221	2.224864
O	-0.742979	1.396165	2.270395
H	0.936227	-1.130648	-1.099048
H	1.548160	-3.458384	-1.483459
H	3.928622	-4.149326	-1.421805
H	5.672859	-2.451714	-0.992992
H	5.069057	-0.125501	-0.613920
H	-5.067282	0.144518	-0.642500
H	-5.664586	2.477772	-0.990451
H	-3.914079	4.179201	-1.375674
H	-1.534166	3.485065	-1.430166
H	-0.929522	1.150336	-1.077781
H	1.792519	-1.377117	4.009250
H	2.397802	-0.315127	2.712193
H	3.653103	-2.686685	3.105741
H	3.241556	-2.199445	1.454158
H	2.069142	-4.298744	1.566194
H	1.704587	-4.085134	3.278888
H	0.224945	-2.938248	0.929481
H	-0.333097	-3.157194	2.592447
H	-0.194565	2.912814	1.007456
H	0.300141	3.127914	2.691248
H	-2.105989	4.209416	1.576923
H	-1.760256	4.045049	3.298561
H	-3.240777	2.096252	1.497611
H	-3.663058	2.588944	3.144530
H	-1.771693	1.328091	4.058691
H	-2.357512	0.238998	2.775373

69
structure_1b.xyz

6	2.585201	0.406913	-0.136863
6	3.804349	0.000064	-1.003967
6	3.005456	0.485500	1.356005
6	2.091033	1.773173	-0.632632
6	2.707002	2.976224	-0.321019
6	2.197354	4.167082	-0.803128
6	1.075009	4.173680	-1.609291
6	0.468687	2.978153	-1.938372
6	0.979258	1.790426	-1.454512
6	-2.637320	0.297041	0.285554
6	-3.825966	-0.001813	1.233750
6	-3.063310	0.072392	-1.189580
6	-2.210963	1.756019	0.499721
6	-2.939731	2.847176	0.048725
6	-2.487419	4.131942	0.282892
6	-1.311751	4.344823	0.977932
6	-0.590822	3.263016	1.441324
6	-1.041792	1.980184	1.202153
6	0.335829	-2.133034	-2.860260
6	0.000363	-3.317619	-3.737076
6	-1.316502	-3.791207	-3.142010
6	-1.072866	-3.613828	-1.661289
6	-0.482746	-2.657948	2.738188
6	0.656634	-2.455309	3.705485
6	1.719181	-3.398738	3.151682
6	1.436993	-3.431729	1.651200
9	3.415296	-0.157105	-2.275065
9	4.335182	-1.164877	-0.609357
9	4.792661	0.903786	-1.002676
9	3.252691	-0.730000	1.860214
9	4.095948	1.224739	1.600671
9	2.003412	1.002748	2.083066
9	-4.281098	-1.252992	1.089695
9	-4.872367	0.817933	1.063758
9	-3.424769	0.126356	2.503331
9	-3.281147	-1.222574	-1.449937
9	-4.167252	0.727220	-1.570572
9	-2.069971	0.463117	-2.007110
12	-0.015532	-1.297909	0.067108
8	1.668777	-0.569630	-0.248677
8	-1.668763	-0.592143	0.570377
8	-0.206266	-2.469596	-1.567513
8	0.178796	-2.751637	1.466830
1	3.590925	3.001091	0.300205
1	2.684765	5.099016	-0.541745
1	0.671519	5.110094	-1.975787
1	-0.416508	2.965358	-2.562548
1	0.505917	0.856660	-1.718382
1	-3.868378	2.709842	-0.486466
1	-3.063680	4.973973	-0.082330
1	-0.956534	5.353488	1.152952
1	0.337775	3.411631	1.978379
1	-0.476790	1.133025	1.560978
1	1.399551	-1.940841	-2.738281
1	-0.156875	-1.222977	-3.209265
1	0.764266	-4.091190	-3.641160
1	-0.076075	-3.037726	-4.786046
1	-2.136186	-3.148783	-3.467313
1	-1.561016	-4.820959	-3.396161
1	-1.972150	-3.407605	-1.083684
1	-0.551771	-4.471409	-1.229277
1	-1.015110	-3.594748	2.925884
1	-1.197588	-1.840427	2.674891
1	0.378762	-2.685292	4.732549
1	0.996420	-1.419693	3.662234
1	1.604209	-4.394038	3.581184
1	2.729948	-3.055595	3.361435
1	2.179972	-2.895790	1.063033
1	1.336422	-4.448795	1.269435

69
structure_1c.xyz

C	2.738188	-0.107488	0.143028
C	3.052722	-0.410340	-1.346092
C	3.909373	-0.583952	1.038891
C	2.560582	1.401300	0.361107
C	3.395407	2.363814	-0.187585
C	3.169454	3.704930	0.057874
C	2.117122	4.102072	0.861006
C	1.293402	3.147689	1.422786
C	1.518673	1.808383	1.173913
C	-2.403523	0.862396	-0.072896
C	-2.753161	0.980311	1.435361
C	-3.699788	0.665292	-0.900316
C	-1.718207	2.142032	-0.572266
C	-0.642560	1.999230	-1.430034
C	0.026192	3.101893	-1.922354
C	-0.380765	4.371886	-1.565893
C	-1.464531	4.526519	-0.723024
C	-2.133711	3.420986	-0.232530
C	0.327190	-3.765216	-1.691613
C	-0.648052	-4.593767	-2.493223
C	-1.238670	-3.551656	-3.429470
C	-1.369735	-2.341681	-2.529939
C	0.130524	-2.593252	2.755432
C	-0.998652	-2.303765	3.712339
C	-2.134061	-3.134951	3.125952
C	-1.851377	-3.133629	1.626557
F	2.113163	0.150470	-2.126906
F	3.020657	-1.724130	-1.597422
F	4.241322	0.030746	-1.778641
F	4.173564	-1.886079	0.872889
F	5.053952	0.079410	0.824697
F	3.585575	-0.409202	2.324959
F	-1.651869	1.301095	2.131163
F	-3.189431	-0.186040	1.929428
F	-3.690553	1.889612	1.734828
F	-4.380613	-0.423633	-0.515303
F	-4.545728	1.701361	-0.834263
F	-3.385371	0.493005	-2.189365
Mg	-0.106632	-1.243015	0.029124
O	1.653914	-0.828810	0.483731
O	-1.657450	-0.243850	-0.227907
O	-0.316364	-2.487837	-1.552541
O	-0.516631	-2.612338	1.473762
H	4.232250	2.080918	-0.810131
H	3.825253	4.445213	-0.385286
H	1.937320	5.154861	1.043847
H	0.459577	3.442372	2.048055
H	0.875620	1.061648	1.615435
H	-0.323234	1.008846	-1.718772
H	0.878013	2.962397	-2.576647
H	0.148194	5.240314	-1.940165
H	-1.796568	5.518463	-0.439813
H	-2.984083	3.572517	0.416685
H	0.531081	-4.150153	-0.693157
H	1.271451	-3.622701	-2.219659
H	-1.420920	-5.006612	-1.841973
H	-0.158740	-5.415032	-3.013633
H	-0.548320	-3.345786	-4.248968
H	-2.196885	-3.845005	-3.854077
H	-1.233003	-1.390739	-3.041142
H	-2.320370	-2.315036	-1.997132
H	0.916808	-1.842354	2.713283
H	0.577793	-3.574455	2.937536
H	-1.242145	-1.241226	3.686024
H	-0.757360	-2.579316	4.737437
H	-3.114530	-2.719354	3.348819
H	-2.100423	-4.151740	3.518045
H	-1.881687	-4.135425	1.194825
H	-2.517493	-2.477498	1.068067

69

structure_1d.xyz

6	2.566863	-0.781649	-0.090010
6	3.809415	-0.822555	0.835485
6	2.943804	-0.214168	-1.483168
6	2.017894	-2.204749	-0.267606
6	2.763853	-3.256128	-0.783610
6	2.196682	-4.507974	-0.926483
6	0.880118	-4.727866	-0.563680
6	0.134532	-3.686265	-0.048334
6	0.707431	-2.438278	0.101388
6	-2.609271	0.161524	-0.616013
6	-2.234956	-0.186059	-2.082526
6	-3.872715	1.059558	-0.588459
6	-2.922325	-1.117133	0.174822
6	-2.403278	-1.224269	1.452927
6	-2.648239	-2.340531	2.229152
6	-3.424357	-3.370381	1.733266
6	-3.954241	-3.268541	0.460739
6	-3.710664	-2.148848	-0.312592
6	-1.119987	2.238640	2.916673
6	-1.199346	1.644253	4.303474
6	0.269510	1.475630	4.657041
6	0.867000	1.018467	3.344813
6	0.287101	3.399050	-1.716045
6	1.068840	4.675135	-1.923396
6	2.381587	4.360143	-1.223218
6	1.917462	3.594000	-0.005263
9	3.487227	-1.413052	1.992377
9	4.246932	0.407219	1.134468
9	4.855660	-1.489931	0.325975
9	1.839627	-0.135227	-2.239839
9	3.438930	1.030657	-1.395333
9	3.839355	-0.936970	-2.163744
9	-1.283831	-1.125240	-2.095713
9	-1.739541	0.879471	-2.722623
9	-3.256348	-0.637580	-2.826956
9	-3.708805	2.176127	-1.308419
9	-4.978091	0.455387	-1.042890
9	-4.122078	1.437007	0.672318
12	0.180957	1.139828	0.362447
8	1.693582	0.065169	0.478529
8	-1.600869	0.875419	-0.090685
8	0.047919	1.636075	2.328937
8	0.720603	2.913155	-0.429761
1	3.792530	-3.110612	-1.080590
1	2.791623	-5.318670	-1.330395
1	0.438248	-5.709929	-0.685229
1	-0.900688	-3.833058	0.236243
1	0.121821	-1.628269	0.507577
1	-1.796266	-0.416924	1.838524
1	-2.226488	-2.406503	3.225228
1	-3.615988	-4.250212	2.335925
1	-4.565791	-4.068703	0.061133
1	-4.144300	-2.091327	-1.300869
1	-1.972531	2.010988	2.279315
1	-0.968912	3.320345	2.948341
1	-1.701089	0.675228	4.272019
1	-1.740544	2.287631	4.994711
1	0.698786	2.432071	4.960705
1	0.443060	0.754435	5.453521
1	1.897496	1.329893	3.186437
1	0.809110	-0.063225	3.216508
1	-0.792866	3.526850	-1.678578
1	0.532731	2.647024	-2.468435
1	0.565686	5.513417	-1.438284
1	1.189926	4.909953	-2.979193
1	2.951534	5.247566	-0.954303
1	3.005893	3.724254	-1.851826
1	2.627414	2.845213	0.344212
1	1.650648	4.259253	0.818946

69

structure_1e.xyz

6	-2.515630	0.323928	-0.629869
6	-2.103057	0.086414	-2.107815
6	-3.718983	1.300074	-0.567171
6	-2.948966	-0.993684	0.027423
6	-3.758268	-1.935104	-0.589682
6	-4.115941	-3.092791	0.076121
6	-3.682708	-3.320908	1.368767
6	-2.885041	-2.380671	1.992846
6	-2.523784	-1.228650	1.322216
6	2.572008	-0.828025	-0.088317
6	3.806523	-1.131143	0.795914
6	2.997903	-0.102045	-1.390803
6	1.880198	-2.152609	-0.442601
6	0.607795	-2.367450	0.051617
6	-0.088119	-3.523052	-0.245440
6	0.494555	-4.493246	-1.035892
6	1.775552	-4.298083	-1.519193
6	2.466129	-3.137829	-1.226349
6	0.437838	3.552999	-1.342694
6	1.222742	4.841896	-1.419845
6	2.528549	4.459632	-0.740635
6	2.049939	3.585116	0.395511
6	-1.138031	1.865783	3.137782
6	-1.375407	0.881339	4.285837
6	-0.153870	-0.039249	4.255552
6	0.874276	0.788177	3.521716
9	-1.227564	-0.919443	-2.180072
9	-1.501817	1.165604	-2.622123
9	-3.124044	-0.208745	-2.928246
9	-4.842165	0.812357	-1.108105
9	-3.992417	1.586950	0.712709
9	-3.455109	2.459873	-1.182003
9	3.413058	-1.775165	1.901747
9	4.424140	-0.012702	1.194795
9	4.732002	-1.895187	0.198721
9	3.864504	-0.769950	-2.159785
9	1.906881	0.136803	-2.135405
9	3.553871	1.090970	-1.133958
12	0.281718	1.116654	0.521518
8	-1.488015	0.914823	0.000721
8	1.792176	0.018115	0.609201
8	0.865652	2.939456	-0.109878
8	0.098210	1.464673	2.524534
1	-4.119395	-1.777437	-1.596156
1	-4.741710	-3.822571	-0.423629
1	-3.965457	-4.229425	1.887096
1	-2.538086	-2.546354	3.006301
1	-1.898188	-0.489274	1.801190
1	0.149076	-1.615997	0.676996
1	-1.092625	-3.652793	0.139187
1	-0.046721	-5.400760	-1.276439
1	2.245102	-5.055174	-2.136173
1	3.465327	-3.011595	-1.617202
1	-0.642111	3.678914	-1.297501
1	0.684854	2.878518	-2.164582
1	0.716425	5.631121	-0.861295
1	1.353359	5.176300	-2.447289
1	3.157537	3.882590	-1.419719
1	3.097825	5.316362	-0.384732
1	2.758313	2.811697	0.689017
1	1.765613	4.172778	1.271280
1	-1.903366	1.837679	2.364504
1	-1.026338	2.890555	3.497100
1	-2.305011	0.330072	4.155371
1	-1.437968	1.414382	5.233675
1	-0.359213	-0.946746	3.686161
1	0.180600	-0.332101	5.249097
1	1.335233	1.537072	4.172850
1	1.642867	0.218586	3.005609

69
structure_1f.xyz

6	-2.493032	0.382745	-0.638566
6	-2.026678	0.296587	-2.118004
6	-3.707097	1.340353	-0.521333
6	-2.936449	-0.998249	-0.137197
6	-3.714350	-1.873779	-0.879305
6	-4.083940	-3.097477	-0.353033
6	-3.694956	-3.458409	0.923398
6	-2.928658	-2.585163	1.671392
6	-2.553928	-1.366863	1.139289
6	2.593172	-0.687929	-0.142033
6	3.863733	-0.936315	0.709308
6	2.948207	0.087719	-1.437368
6	1.970423	-2.038615	-0.525977
6	0.674959	-2.296274	-0.120668
6	0.042932	-3.478327	-0.454729
6	0.713524	-4.432860	-1.192800
6	2.016136	-4.193083	-1.590463
6	2.641583	-3.005459	-1.263312
6	2.058293	3.546472	0.654665
6	2.415128	4.370937	-0.581865
6	1.235929	4.155432	-1.526868
6	0.103488	3.882109	-0.568934
6	-1.249993	1.364358	3.400645
6	-1.497665	0.213235	4.379020
6	-0.282976	-0.700586	4.206438
6	0.755837	0.226082	3.621686
9	-1.159903	-0.707130	-2.266681
9	-1.396709	1.416694	-2.494202
9	-3.020692	0.104902	-2.999915
9	-4.022790	1.501614	0.770204
9	-3.431475	2.555495	-1.013846
9	-4.808582	0.902574	-1.142648
9	4.850195	-1.567288	0.056172
9	3.547487	-1.686945	1.772769
9	4.379489	0.205432	1.178838
9	3.513930	1.274456	-1.166898
9	3.777702	-0.550340	-2.269609
9	1.822048	0.344318	-2.116413
12	0.235556	1.121031	0.720897
8	-1.495320	0.914107	0.086007
8	1.785378	0.093608	0.595938
8	0.718124	3.062870	0.435956
8	-0.008548	1.062251	2.744262
1	-4.040830	-1.612799	-1.876180
1	-4.684231	-3.774091	-0.949448
1	-3.987431	-4.418139	1.332512
1	-2.616102	-2.856143	2.673348
1	-1.950247	-0.679937	1.714129
1	0.143401	-1.558489	0.460654
1	-0.980842	-3.641106	-0.139611
1	0.223604	-5.361728	-1.460366
1	2.554010	-4.935870	-2.167733
1	3.657693	-2.842744	-1.591991
1	2.694947	2.674585	0.792534
1	2.060819	4.145296	1.566634
1	3.357555	4.048867	-1.019131
1	2.507513	5.424841	-0.319999
1	1.043457	5.014247	-2.167400
1	1.398033	3.281390	-2.158574
1	-0.263745	4.798265	-0.097503
1	-0.733724	3.325467	-0.982726
1	-2.008971	1.457940	2.625570
1	-1.143357	2.320991	3.915352
1	-2.431465	-0.302857	4.162677
1	-1.558000	0.591256	5.398748
1	-0.490941	-1.502425	3.496882
1	0.042825	-1.152346	5.141566
1	1.220086	0.854563	4.387557
1	1.524168	-0.257968	3.024179

69
structure_1g.xyz

C	-2.498510	0.906972	-0.088908
C	-3.747973	1.159356	0.790769
C	-2.902450	0.256950	-1.437520
C	-1.799813	2.245687	-0.368181
C	-0.502354	2.400114	0.080477
C	0.202117	3.563994	-0.158279
C	-0.396700	4.602255	-0.843467
C	-1.700818	4.464863	-1.283084
C	-2.399702	3.295957	-1.050284
C	2.528721	-0.417813	-0.655471
C	2.123068	-0.128582	-2.126317
C	3.699438	-1.434036	-0.618731
C	3.005412	0.869767	0.031085
C	2.580597	1.095095	1.327431
C	2.977615	2.220604	2.022502
C	3.811823	3.143730	1.421291
C	4.245041	2.925125	0.126923
C	3.851201	1.793846	-0.563226
C	1.113768	-1.940338	3.110616
C	1.365244	-0.947360	4.248359
C	0.157604	-0.009168	4.207493
C	-0.883282	-0.831288	3.486290
C	-0.824282	-3.491302	-1.440309
C	-2.124577	-4.196793	-1.748576
C	-2.568280	-4.668487	-0.373081
C	-2.175571	-3.503899	0.508284
F	-3.378066	1.770043	1.923410
F	-4.349079	0.016230	1.142979
F	-4.682930	1.926527	0.212197
F	-3.490983	-0.935706	-1.257972
F	-3.732685	0.983978	-2.192215
F	-1.797075	0.033055	-2.164116
F	1.285820	0.910782	-2.171574
F	1.481383	-1.171256	-2.667117
F	3.153452	0.148593	-2.941025
F	3.398147	-2.567421	-1.263000
F	4.838532	-0.968994	-1.146998
F	3.962848	-1.761574	0.653660
Mg	-0.286066	-1.154760	0.494515
O	-1.730585	0.026401	0.576837
O	1.483019	-0.989994	-0.037334
O	-0.118529	-1.529429	2.494437
O	-1.011630	-2.934481	-0.123783
H	-0.031493	1.592172	0.619517
H	1.225254	3.646769	0.188693
H	0.150257	5.517513	-1.037384
H	-2.181874	5.274442	-1.819286
H	-3.415692	3.213928	-1.408474
H	1.926117	0.369102	1.787713
H	2.629943	2.379098	3.036936
H	4.122885	4.031798	1.958493
H	4.899140	3.641879	-0.355103
H	4.212825	1.642852	-1.570519
H	1.878633	-1.930565	2.336314
H	0.989036	-2.959808	3.480370
H	2.303251	-0.411748	4.112953
H	1.418912	-1.471208	5.201844
H	-0.170927	0.301867	5.197454
H	0.374552	0.887477	3.625223
H	-1.351896	-1.566960	4.146947
H	-1.644932	-0.256350	2.965885
H	0.021811	-4.179688	-1.399216
H	-0.589697	-2.679519	-2.126337
H	-1.988085	-5.010128	-2.458858
H	-2.846684	-3.491692	-2.161408
H	-2.022088	-5.567669	-0.083023
H	-3.634433	-4.879178	-0.314328
H	-2.949064	-2.735087	0.547625
H	-1.899440	-3.791941	1.521979

69
structure_1h.xyz

6	2.814491	-0.218004	0.206987
6	3.016486	-0.712655	1.664306
6	3.968663	0.734763	-0.199339
6	2.816290	-1.416021	-0.752466
6	1.773335	-1.528767	-1.653811
6	1.717630	-2.588699	-2.539910
6	2.709878	-3.548707	-2.537007
6	3.760569	-3.437833	-1.645250
6	3.817022	-2.378352	-0.760777
6	-2.516941	-0.396809	-0.845077
6	-2.665994	-1.110580	-2.213854
6	-3.722472	0.559788	-0.634040
6	-2.476481	-1.478972	0.241594
6	-1.231059	-1.962721	0.607309
6	-1.105469	-2.930177	1.586258
6	-2.229813	-3.428212	2.215338
6	-3.478146	-2.965624	1.842298
6	-3.603063	-2.004997	0.856563
6	-0.471918	0.441200	3.170333
6	-1.886953	0.231240	3.645710
6	-2.466834	1.643335	3.590271
6	-1.614530	2.358745	2.541445
6	1.215739	3.776756	-0.303572
6	1.536280	4.155172	-1.727050
6	0.154837	4.477699	-2.277452
6	-0.744985	3.471083	-1.578666
9	2.112371	-1.666054	1.941761
9	2.823164	0.270713	2.549488
9	4.225842	-1.229055	1.922247
9	4.055312	1.790477	0.623849
9	5.177140	0.159794	-0.221118
9	3.736557	1.214097	-1.426850
9	-1.561987	-1.816957	-2.474676
9	-2.827176	-0.240625	-3.218150
9	-3.699210	-1.962950	-2.256553
9	-3.619504	1.652216	-1.393125
9	-4.921966	0.020264	-0.896890
9	-3.752453	0.981927	0.643140
12	-0.066303	1.098560	0.197927
8	1.678417	0.491991	0.181105
8	-1.403617	0.357155	-0.885232
8	-0.619730	1.409319	2.122721
8	-0.008027	3.029813	-0.413712
1	0.991915	-0.781223	-1.663493
1	0.888279	-2.660962	-3.232530
1	2.666487	-4.381931	-3.228501
1	4.547268	-4.182996	-1.635259
1	4.652077	-2.311827	-0.078254
1	-0.343837	-1.595119	0.107150
1	-0.120462	-3.294445	1.851678
1	-2.135512	-4.181918	2.988032
1	-4.368181	-3.355618	2.321708
1	-4.592419	-1.669166	0.579392
1	0.172223	0.861936	3.947309
1	0.002010	-0.442193	2.751257
1	-1.930471	-0.205987	4.641549
1	-2.404686	-0.431985	2.952492
1	-3.519734	1.641022	3.316959
1	-2.371002	2.140089	4.555303
1	-2.175721	2.657120	1.658603
1	-1.103347	3.231816	2.950795
1	1.954817	3.134398	0.170101
1	1.034165	4.656088	0.320511
1	1.975250	3.300646	-2.243765
1	2.228779	4.992680	-1.789504
1	0.094414	4.384950	-3.360071
1	-0.128881	5.496065	-2.008248
1	-1.689487	3.899097	-1.244085
1	-0.956828	2.589986	-2.182261

69

structure_1i.xyz

6	-3.027875	0.197313	-0.542735
6	-3.766913	0.334449	-1.899717
6	-3.878744	0.866834	0.569974
6	-2.825919	-1.299236	-0.270426
6	-3.677502	-2.081294	0.495425
6	-3.391025	-3.415564	0.717899
6	-2.261557	-3.991743	0.166497
6	-1.421544	-3.224970	-0.617389
6	-1.703931	-1.889637	-0.833170
6	2.523006	-0.529129	-0.861621
6	2.742042	0.422445	-2.068035
6	2.486920	-2.003186	-1.340440
6	3.671866	-0.381127	0.145939
6	3.333143	-0.216839	1.477626
6	4.310891	-0.082290	2.444934
6	5.645618	-0.110472	2.089013
6	5.991538	-0.281892	0.761735
6	5.013214	-0.421532	-0.204616
6	-0.480506	3.669989	0.120521
6	0.571600	4.370636	-0.702061
6	1.796175	4.298256	0.205500
6	1.567087	3.037810	1.038010
6	-0.303117	-1.523052	2.352519
6	-0.103253	-1.633629	3.844866
6	-0.880826	-0.429856	4.352869
6	-0.541509	0.624101	3.322613
9	-4.886899	-0.400931	-1.959818
9	-2.973143	-0.073099	-2.892747
9	-4.110089	1.601478	-2.160984
9	-3.380117	0.555268	1.780422
9	-3.844678	2.197407	0.478527
9	-5.172854	0.512253	0.574993
9	2.952170	1.672402	-1.619425
9	1.665333	0.472522	-2.854354
9	3.782793	0.105814	-2.850781
9	1.530435	-2.207482	-2.253203
9	3.638303	-2.432357	-1.873808
9	2.215364	-2.799496	-0.298298
12	-0.192986	0.654235	0.220123
8	-1.863276	0.873654	-0.620209
8	1.328128	-0.246619	-0.328814
8	0.242756	2.576935	0.710339
8	-0.348220	-0.103056	2.099011
1	-4.571977	-1.661663	0.933773
1	-4.061294	-4.009013	1.328510
1	-2.040452	-5.037562	0.343844
1	-0.536173	-3.660429	-1.063180
1	-1.043175	-1.300958	-1.457364
1	2.284982	-0.196294	1.744512
1	4.027374	0.043958	3.483542
1	6.415784	-0.003025	2.843658
1	7.035088	-0.310078	0.471467
1	5.309467	-0.564172	-1.234223
1	-0.864073	4.307951	0.922165
1	-1.310964	3.245922	-0.439438
1	0.291848	5.391560	-0.955758
1	0.740040	3.818053	-1.627129
1	2.725707	4.242182	-0.356930
1	1.845644	5.174739	0.851442
1	2.259180	2.233062	0.800508
1	1.606189	3.238530	2.110194
1	0.502429	-1.940941	1.750755
1	-1.253208	-1.956813	2.035533
1	0.955177	-1.543284	4.097086
1	-0.471081	-2.580616	4.235213
1	-0.599768	-0.123325	5.358809
1	-1.951929	-0.637486	4.339922
1	-1.328525	1.359501	3.166501
1	0.391613	1.139754	3.563390

69

structure_1j.xyz

C	-2.901471	-0.114027	-0.142163
C	-3.066022	0.455065	-1.574672
C	-3.778854	0.679843	0.861504
C	-3.340013	-1.584205	-0.147229
C	-4.641295	-1.994268	-0.401017
C	-4.962043	-3.338300	-0.390010
C	-3.990300	-4.286923	-0.130576
C	-2.693667	-3.881604	0.119622
C	-2.372920	-2.537201	0.112489
C	2.523521	-0.860990	-0.292757
C	2.019717	-1.669158	-1.515500
C	2.309862	-1.662961	1.017371
C	4.018679	-0.573371	-0.461947
C	4.986192	-1.565957	-0.515138
C	6.318755	-1.230991	-0.664118
C	6.699981	0.094176	-0.762280
C	5.738540	1.085107	-0.708597
C	4.406019	0.750481	-0.558139
C	0.663161	2.723479	-2.069012
C	0.560965	4.193158	-2.401021
C	-0.709393	4.592411	-1.667011
C	-0.597215	3.803830	-0.380816
C	1.504054	2.029327	2.625354
C	1.543177	1.683965	4.105827
C	0.510644	0.573336	4.234750
C	-0.529185	0.997157	3.228019
F	-2.252864	-0.200230	-2.408832
F	-2.718250	1.753469	-1.633901
F	-4.304012	0.367972	-2.073913
F	-3.320658	1.928644	1.038429
F	-5.067358	0.792647	0.509557
F	-3.744451	0.084676	2.059440
F	2.192736	-0.944614	-2.625896
F	0.709562	-1.932310	-1.414259
F	2.638970	-2.841160	-1.707203
F	2.774379	-2.916002	0.997389
F	2.908020	-1.032569	2.037910
F	1.003634	-1.737149	1.331541
Mg	0.053904	0.836100	0.235015
O	-1.623314	0.047823	0.230744
O	1.789174	0.263381	-0.213083
O	0.136126	2.617006	-0.731998
O	0.243113	1.526407	2.136525
H	-5.417873	-1.272342	-0.610229
H	-5.982399	-3.644738	-0.588165
H	-4.244893	-5.340327	-0.125084
H	-1.921772	-4.615110	0.321075
H	-1.362542	-2.208062	0.306290
H	4.711594	-2.608926	-0.441299
H	7.064867	-2.015729	-0.704249
H	7.745698	0.352804	-0.880319
H	6.026856	2.127182	-0.784344
H	3.638548	1.511099	-0.510175
H	0.043128	2.116621	-2.733029
H	1.674572	2.322903	-2.059417
H	0.510655	4.366085	-3.474376
H	1.421631	4.734076	-2.003345
H	-0.785659	5.662610	-1.483656
H	-1.588475	4.273875	-2.228619
H	-0.023621	4.341395	0.377622
H	-1.557148	3.505213	0.036222
H	1.541757	3.103269	2.439249
H	2.290923	1.537768	2.054467
H	1.247612	2.544749	4.707101
H	2.539119	1.376961	4.419264
H	0.102168	0.485793	5.239948
H	0.936945	-0.387749	3.946164
H	-1.146889	0.192586	2.835590
H	-1.175156	1.789227	3.615663

69
structure_1k.xyz

6	-2.927967	-0.081383	-0.132980
6	-3.054050	0.505008	-1.562577
6	-3.825915	0.708582	0.855622
6	-3.372864	-1.549110	-0.166313
6	-4.671991	-1.949255	-0.445490
6	-4.999116	-3.291707	-0.458213
6	-4.035753	-4.248556	-0.197755
6	-2.740920	-3.853218	0.076541
6	-2.413838	-2.510409	0.093017
6	2.525233	-0.893179	-0.298238
6	2.057387	-1.636831	-1.575036
6	2.341938	-1.786523	0.955698
6	4.009284	-0.535773	-0.440751
6	4.343963	0.806018	-0.437622
6	5.661981	1.204276	-0.557188
6	6.662745	0.259581	-0.680194
6	6.334578	-1.083198	-0.683423
6	5.016413	-1.481595	-0.565169
6	-0.529957	3.804059	-0.326383
6	-0.611128	4.612769	-1.602744
6	0.640668	4.171572	-2.345000
6	0.683739	2.695071	-2.031330
6	-0.573775	0.998971	3.255938
6	0.439823	0.417236	4.208837
6	1.565592	1.439446	4.133037
6	1.517607	1.915355	2.687568
9	-2.215298	-0.137083	-2.381959
9	-2.709564	1.805188	-1.596519
9	-4.276999	0.419282	-2.097058
9	-3.360670	1.949967	1.060955
9	-5.102754	0.838595	0.468366
9	-3.831603	0.098110	2.046461
9	2.713357	-2.777021	-1.828953
9	2.225278	-0.839217	-2.636115
9	0.755042	-1.942197	-1.512843
9	1.042500	-1.982365	1.231633
9	2.906764	-2.996059	0.874437
9	2.869748	-1.172540	2.024828
12	0.035499	0.821919	0.275396
8	-1.659302	0.069173	0.276467
8	1.749635	0.194171	-0.148758
8	0.159675	2.595763	-0.692841
8	0.227484	1.514856	2.179697
1	-5.441955	-1.220625	-0.655983
1	-6.017869	-3.590381	-0.675521
1	-4.295394	-5.300656	-0.210473
1	-1.975333	-4.593144	0.278457
1	-1.404425	-2.189613	0.305643
1	3.546927	1.530423	-0.338359
1	5.908022	2.259851	-0.554146
1	7.697384	0.567896	-0.773886
1	7.111427	-1.832467	-0.779507
1	4.784492	-2.537200	-0.571398
1	0.061770	4.309142	0.440213
1	-1.501018	3.535302	0.085826
1	-0.643306	5.682810	-1.405966
1	-1.503221	4.337699	-2.166864
1	0.594956	4.359825	-3.415972
1	1.523565	4.671985	-1.943044
1	0.035458	2.122449	-2.698817
1	1.677717	2.252409	-2.031293
1	-1.273272	0.283099	2.830826
1	-1.130506	1.825542	3.705697
1	0.773325	-0.555260	3.845459
1	0.042335	0.295641	5.214919
1	2.537204	1.017854	4.383082
1	1.369167	2.267782	4.814775
1	1.602007	2.999096	2.597121
1	2.270340	1.440605	2.059708

69
structure_1l.xyz

6	-2.566178	-0.674252	-0.338113
6	-3.856948	-1.180595	0.354728
6	-1.970968	-1.769819	-1.260747
6	-2.909536	0.561176	-1.182981
6	-2.313644	1.763242	-0.850563
6	-2.574362	2.911541	-1.572698
6	-3.444562	2.871373	-2.644035
6	-4.047634	1.674772	-2.983322
6	-3.784369	0.527181	-2.260127
6	2.559855	0.677163	-0.357847
6	3.858833	1.165953	0.332788
6	1.984351	1.777518	-1.287501
6	2.883148	-0.569494	-1.193867
6	3.760821	-0.557724	-2.269181
6	4.006739	-1.714629	-2.983534
6	3.382591	-2.898453	-2.637569
6	2.508845	-2.916394	-1.568516
6	2.265846	-1.758910	-0.855027
6	-1.798949	1.950726	2.862350
6	-1.934726	3.433114	2.511421
6	-0.528855	3.853277	2.072208
6	0.351413	2.758496	2.629136
6	-0.320092	-2.743935	2.645554
6	0.559828	-3.837092	2.084796
6	1.968236	-3.408037	2.506817
6	1.829501	-1.924613	2.851931
9	-3.615406	-2.237463	1.141572
9	-4.836409	-1.539470	-0.484895
9	-4.346086	-0.211690	1.139810
9	-1.535220	-2.823676	-0.553740
9	-2.820989	-2.248838	-2.178808
9	-0.914960	-1.275218	-1.911702
9	3.634867	2.230544	1.114087
9	4.844464	1.503843	-0.508195
9	4.331865	0.193065	1.123345
9	0.919840	1.298350	-1.936250
9	1.567997	2.843327	-0.587284
9	2.843627	2.234996	-2.208192
12	0.001022	0.004425	1.307570
8	-1.675875	-0.430821	0.637516
8	1.664521	0.456089	0.618035
8	-0.458293	1.584018	2.496055
8	0.482711	-1.565943	2.500369
1	-1.631683	1.797821	-0.013980
1	-2.089008	3.841006	-1.298257
1	-3.650846	3.768296	-3.215889
1	-4.730934	1.629534	-3.823030
1	-4.268357	-0.394735	-2.548299
1	4.260802	0.353974	-2.562497
1	4.692954	-1.686759	-3.821614
1	3.575207	-3.802684	-3.202616
1	2.006288	-3.835313	-1.289587
1	1.580736	-1.777046	-0.020323
1	-1.919135	1.773789	3.933060
1	-2.471560	1.298753	2.309711
1	-2.263775	3.999417	3.381503
1	-2.665033	3.587648	1.719271
1	-0.446627	3.864391	0.985479
1	-0.245763	4.837078	2.442070
1	1.273231	2.588084	2.079219
1	0.571947	2.910128	3.689973
1	-1.247904	-2.580225	2.103535
1	-0.530730	-2.892429	3.708808
1	0.466620	-3.854406	0.999070
1	0.285408	-4.820324	2.462566
1	2.690377	-3.562793	1.707301
1	2.309468	-3.968683	3.375868
1	1.962061	-1.741193	3.920085
1	2.492059	-1.272594	2.287612

69
structure_1m.xyz

6	-2.899657	-0.100999	-0.172801
6	-3.024878	0.399451	-1.634522
6	-3.773082	0.761917	0.775674
6	-3.372184	-1.559364	-0.116743
6	-4.681107	-1.949943	-0.360968
6	-5.032916	-3.284640	-0.294303
6	-4.084529	-4.243167	0.010823
6	-2.779626	-3.857520	0.249288
6	-2.427700	-2.522415	0.186395
6	2.511772	-0.879788	-0.317015
6	2.029429	-1.683850	-1.551670
6	2.281540	-1.691978	0.984606
6	4.008956	-0.588896	-0.464093
6	4.392150	0.734708	-0.576081
6	5.725875	1.073094	-0.706439
6	6.692629	0.086075	-0.723420
6	6.315377	-1.239052	-0.609811
6	4.981653	-1.577706	-0.481711
6	0.802591	2.827262	-1.941857
6	0.099904	4.018447	-2.548838
6	-0.187102	4.871766	-1.323770
6	-0.571115	3.830948	-0.295039
6	-0.537647	0.926823	3.224993
6	0.497106	0.509118	4.239615
6	1.517908	1.632048	4.126722
6	1.496587	1.974239	2.645941
9	-2.227873	-0.327051	-2.422982
9	-2.626889	1.679276	-1.755416
9	-4.259992	0.332754	-2.144320
9	-3.287448	2.006996	0.895874
9	-5.053845	0.886950	0.400123
9	-3.769161	0.227350	2.002352
9	2.188795	-0.940458	-2.651790
9	0.726434	-1.982258	-1.459800
9	2.679714	-2.836775	-1.756392
9	2.725432	-2.952482	0.951554
9	2.886395	-1.083332	2.014618
9	0.973874	-1.748766	1.294798
12	0.053129	0.835830	0.226364
8	-1.624640	0.047634	0.216075
8	1.775160	0.243084	-0.240678
8	0.183950	2.658655	-0.651691
8	0.237502	1.478266	2.146282
1	-5.439459	-1.219953	-0.606152
1	-6.059129	-3.575933	-0.484899
1	-4.363477	-5.289226	0.060338
1	-2.025681	-4.599519	0.484759
1	-1.410258	-2.208378	0.368969
1	3.619523	1.491108	-0.556756
1	6.011127	2.114992	-0.795198
1	7.739367	0.347540	-0.825030
1	7.065610	-2.020805	-0.621238
1	4.710295	-2.620627	-0.395622
1	0.684109	1.902272	-2.503347
1	1.867741	3.010452	-1.788797
1	-0.831287	3.705563	-3.023284
1	0.716024	4.521981	-3.291462
1	0.712539	5.405366	-1.012094
1	-0.981451	5.599012	-1.480751
1	-0.311936	4.102790	0.727647
1	-1.631214	3.581994	-0.342080
1	-1.139695	0.117378	2.818541
1	-1.199623	1.705559	3.612562
1	0.937755	-0.445322	3.950374
1	0.079510	0.411495	5.240120
1	2.512782	1.338186	4.455806
1	1.201893	2.489818	4.722048
1	1.543887	3.047331	2.456562
1	2.287179	1.476563	2.085277

69
structure_1n.xyz

6	-2.663543	0.592737	0.216415
6	-3.366945	0.697972	1.594304
6	-3.592459	-0.086547	-0.821372
6	-2.312660	1.998296	-0.292738
6	-0.996387	2.246530	-0.629532
6	-0.599897	3.488308	-1.087522
6	-1.527556	4.502044	-1.219848
6	-2.850338	4.260645	-0.894075
6	-3.244070	3.018520	-0.434639
6	2.678498	0.085172	-0.464676
6	2.614915	0.086711	-2.014679
6	3.960185	-0.638977	0.021424
6	2.716966	1.524556	0.068821
6	1.863037	1.846428	1.107462
6	1.842326	3.119896	1.642091
6	2.684693	4.092784	1.141767
6	3.549383	3.778117	0.109812
6	3.571356	2.502371	-0.421225
6	-0.189114	-1.499750	3.172705
6	1.158506	-1.306365	3.821673
6	1.824121	-2.647650	3.554584
6	1.313505	-3.015430	2.171546
6	-1.455708	-3.854708	-0.567671
6	-1.859128	-4.568693	-1.838224
6	-1.780611	-3.456718	-2.871470
6	-0.548641	-2.703041	-2.430030
9	-2.580059	1.372631	2.443589
9	-3.577167	-0.508459	2.134308
9	-4.553485	1.321404	1.572110
9	-3.922892	-1.334912	-0.458914
9	-4.737758	0.565107	-1.053342
9	-2.947849	-0.183124	-1.993306
9	1.580325	0.836765	-2.418512
9	2.410325	-1.145439	-2.495730
9	3.711167	0.557674	-2.625252
9	4.012405	-1.906805	-0.407399
9	5.099265	-0.047623	-0.358887
9	3.964537	-0.680464	1.360909
12	-0.097650	-1.255071	0.204490
8	-1.596158	-0.198245	0.389980
8	1.627893	-0.618800	-0.018730
8	0.105770	-2.244181	1.974611
8	-0.537624	-2.825234	-0.991870
1	-0.266791	1.459347	-0.522270
1	0.442856	3.656133	-1.330917
1	-1.223790	5.479550	-1.575850
1	-3.587572	5.048319	-0.996512
1	-4.283850	2.856854	-0.188762
1	1.194057	1.087237	1.486303
1	1.153381	3.352632	2.445150
1	2.667281	5.095277	1.552644
1	4.217475	4.532340	-0.288915
1	4.263920	2.280679	-1.220533
1	-0.861211	-2.095143	3.795482
1	-0.697823	-0.583332	2.878251
1	1.078898	-1.070985	4.881461
1	1.703443	-0.503185	3.323602
1	2.910710	-2.594981	3.577337
1	1.500850	-3.383787	4.291925
1	2.013218	-2.729455	1.386608
1	1.064224	-4.072232	2.074484
1	-2.305949	-3.372960	-0.082073
1	-0.940921	-4.490614	0.150973
1	-2.850632	-5.009890	-1.756265
1	-1.146550	-5.360931	-2.073776
1	-2.658365	-2.812997	-2.808223
1	-1.689859	-3.822942	-3.892357
1	0.370305	-3.148538	-2.813818
1	-0.566479	-1.643908	-2.685336

69
structure_1o.xyz

6	-2.817390	0.124806	-0.332573
6	-2.738959	0.848933	-1.701734
6	-4.073539	0.587435	0.452401
6	-2.900848	-1.389056	-0.572361
6	-3.972251	-1.996944	-1.212762
6	-3.992840	-3.366379	-1.393865
6	-2.944756	-4.147849	-0.944367
6	-1.874221	-3.547979	-0.311455
6	-1.857435	-2.178691	-0.126442
6	2.547873	-0.753439	-0.318929
6	2.073331	-0.838873	-1.792793
6	2.693145	-2.173534	0.286258
6	3.912696	-0.054791	-0.250732
6	4.032760	1.034468	0.593466
6	5.232202	1.711116	0.709399
6	6.331559	1.301916	-0.020177
6	6.221681	0.208998	-0.859191
6	5.022636	-0.468692	-0.973373
6	-0.723091	3.708973	0.662896
6	-0.617047	4.667668	-0.519389
6	0.401971	4.004700	-1.438958
6	1.288658	3.282315	-0.456726
6	-1.291118	0.495664	3.480227
6	-1.449244	-1.007374	3.653407
6	-0.008060	-1.541703	3.612411
6	0.842685	-0.289681	3.489169
9	-3.754634	0.587819	-2.531914
9	-1.611857	0.491931	-2.326833
9	-2.694829	2.183171	-1.552430
9	-4.202980	-0.147299	1.564873
9	-3.969123	1.864397	0.843674
9	-5.223729	0.486521	-0.227481
9	2.028931	0.398362	-2.313678
9	0.842479	-1.344450	-1.881649
9	2.856827	-1.573765	-2.592679
9	1.523751	-2.824023	0.318422
9	3.561457	-2.959962	-0.361180
9	3.119015	-2.074681	1.553169
12	0.035664	0.790116	0.790372
8	-1.752302	0.497929	0.393012
8	1.601187	-0.109714	0.382620
8	0.362271	2.768168	0.511813
8	-0.017236	0.656476	2.836948
1	-4.802269	-1.409301	-1.577729
1	-4.837521	-3.824679	-1.894462
1	-2.962153	-5.221312	-1.091336
1	-1.039559	-4.144105	0.037388
1	-1.013059	-1.718533	0.364984
1	3.168547	1.343492	1.165090
1	5.307156	2.561766	1.376835
1	7.273667	1.830226	0.066769
1	7.078361	-0.124378	-1.432794
1	4.965324	-1.323781	-1.631263
1	-0.607575	4.212995	1.623092
1	-1.646494	3.132459	0.662885
1	-0.252654	5.640470	-0.188589
1	-1.580790	4.814211	-1.002409
1	-0.080461	3.282069	-2.098060
1	0.951275	4.718954	-2.049650
1	1.843118	2.441813	-0.866928
1	1.982485	3.962045	0.046040
1	-2.046828	0.948598	2.844647
1	-1.247668	1.016635	4.440301
1	-2.039896	-1.417943	2.836348
1	-1.956382	-1.242992	4.587459
1	0.143221	-2.182150	2.744034
1	0.256085	-2.115791	4.498799
1	1.111505	0.119037	4.467070
1	1.733061	-0.408320	2.878415

69
structure_1p.xyz

6	-2.820067	0.126860	-0.333273
6	-2.751800	0.860403	-1.697828
6	-4.077899	0.574639	0.457792
6	-2.891799	-1.386046	-0.582629
6	-3.965415	-1.999679	-1.213825
6	-3.975748	-3.368002	-1.403791
6	-2.914907	-4.142670	-0.972768
6	-1.841845	-3.537027	-0.349772
6	-1.835451	-2.168870	-0.155706
6	2.549952	-0.749779	-0.321033
6	2.074041	-0.833368	-1.794845
6	2.704599	-2.170726	0.279846
6	3.910255	-0.042197	-0.253168
6	5.023958	-0.451891	-0.972403
6	6.218685	0.233397	-0.858397
6	6.320289	1.330231	-0.023458
6	5.217010	1.735700	0.702266
6	4.022015	1.051149	0.586898
6	-0.724139	3.700785	0.683968
6	-0.632625	4.661302	-0.498039
6	0.380531	4.003208	-1.427399
6	1.278934	3.283987	-0.453598
6	0.845304	-0.299110	3.494762
6	0.010342	-1.560557	3.632122
6	-1.438035	-1.044935	3.662660
6	-1.298503	0.458844	3.482795
9	-3.761272	0.586566	-2.531450
9	-1.617557	0.526266	-2.322859
9	-2.730882	2.194212	-1.540398
9	-3.978252	1.846552	0.865594
9	-5.227653	0.478528	-0.223894
9	-4.205038	-0.174956	1.560709
9	2.015945	0.405613	-2.310098
9	0.848197	-1.351146	-1.885059
9	2.864366	-1.556332	-2.598738
9	3.135354	-2.073068	1.545070
9	1.538222	-2.826537	0.315598
9	3.573367	-2.951949	-0.373364
12	0.033590	0.782305	0.792295
8	-1.756203	0.505094	0.391248
8	1.600584	-0.114921	0.384891
8	0.363661	2.764216	0.522602
8	-0.027589	0.631007	2.837211
1	-4.805155	-1.417198	-1.564610
1	-4.822485	-3.830837	-1.896725
1	-2.924337	-5.215272	-1.126562
1	-0.996989	-4.127334	-0.016020
1	-0.988021	-1.704697	0.326509
1	4.972983	-1.309674	-1.627295
1	7.078529	-0.097029	-1.428960
1	7.259042	1.864514	0.063272
1	5.285498	2.589493	1.366374
1	3.154866	1.357242	1.155705
1	-0.602401	4.204064	1.643772
1	-1.645363	3.120757	0.690682
1	-0.269177	5.635255	-0.169595
1	-1.601294	4.804457	-0.972094
1	-0.104952	3.278983	-2.082509
1	0.921469	4.719957	-2.042634
1	1.834712	2.447574	-0.870014
1	1.973521	3.966785	0.043997
1	1.112427	0.121494	4.468086
1	1.735141	-0.412450	2.882075
1	0.279770	-2.119230	4.526704
1	0.172051	-2.209885	2.772316
1	-1.947890	-1.283022	4.594604
1	-2.018359	-1.466857	2.843856
1	-2.059926	0.900232	2.846157
1	-1.259644	0.984230	4.440699

69
structure_1q.xyz

6	-2.918107	0.038366	-0.225581
6	-2.934478	0.771444	-1.591665
6	-4.077198	0.539822	0.675090
6	-3.089751	-1.468788	-0.463666
6	-4.211313	-2.024069	-1.064622
6	-4.301083	-3.390942	-1.245589
6	-3.275705	-4.221279	-0.832741
6	-2.158376	-3.673438	-0.234103
6	-2.071476	-2.306667	-0.050305
6	2.522228	-0.672073	-0.433797
6	1.987465	-0.970167	-1.857467
6	2.517572	-1.956102	0.434958
6	3.961182	-0.149590	-0.528449
6	4.220385	1.104257	-0.005544
6	5.494093	1.638667	-0.051072
6	6.526875	0.920150	-0.621925
6	6.275272	-0.335045	-1.143278
6	5.001618	-0.869729	-1.097464
6	-0.832826	3.741274	0.564579
6	-0.855475	4.686799	-0.633874
6	0.085334	4.031939	-1.639481
6	1.058646	3.309877	-0.742066
6	-0.830237	-0.072816	3.460572
6	0.030695	-1.172062	4.030640
6	1.307910	-0.422615	4.394370
6	1.364934	0.719808	3.382383
9	-4.047900	0.592581	-2.311885
9	-1.906702	0.347113	-2.334123
9	-2.783590	2.097828	-1.442173
9	-4.097334	-0.168949	1.811071
9	-3.919376	1.823921	1.021677
9	-5.293360	0.437341	0.121501
9	2.037840	0.151558	-2.589388
9	0.709379	-1.361187	-1.825561
9	2.667537	-1.909873	-2.526615
9	1.271494	-2.397826	0.659226
9	3.205510	-2.981148	-0.080652
9	3.050399	-1.684887	1.634808
12	0.003748	0.810913	0.662118
8	-1.774231	0.354841	0.400861
8	1.691287	0.215218	0.137057
8	0.226693	2.797522	0.308796
8	0.098419	0.708192	2.693982
1	-5.026357	-1.398356	-1.398224
1	-5.182937	-3.808757	-1.716729
1	-3.348401	-5.292459	-0.979707
1	-1.343165	-4.309447	0.090192
1	-1.197070	-1.878185	0.414898
1	3.402172	1.652728	0.440343
1	5.679916	2.622832	0.363236
1	7.526894	1.335830	-0.660118
1	7.077956	-0.908287	-1.591814
1	4.831064	-1.853770	-1.510116
1	-0.613338	4.258659	1.499474
1	-1.751475	3.169024	0.676283
1	-0.482092	5.670412	-0.348367
1	-1.862441	4.810010	-1.026974
1	-0.448214	3.310208	-2.258237
1	0.576439	4.751810	-2.291874
1	1.573246	2.467008	-1.197515
1	1.795157	3.991959	-0.306776
1	-1.619174	-0.397845	2.786990
1	-1.257429	0.558775	4.244896
1	0.225082	-1.921185	3.262026
1	-0.431072	-1.664276	4.884752
1	2.192044	-1.053798	4.335022
1	1.241421	-0.028714	5.408663
1	1.487447	1.692915	3.860245
1	2.137818	0.585394	2.628059

69
structure_1r.xyz

C	2.588401	-0.118430	-0.465963
C	2.139514	-0.432729	-1.915554
C	3.603950	-1.174159	0.037924
C	3.250676	1.263634	-0.437780
C	4.366614	1.595237	-1.191944
C	4.905702	2.865599	-1.114128
C	4.341031	3.816506	-0.284791
C	3.228473	3.489557	0.466327
C	2.688211	2.220437	0.385999
C	-2.786857	0.419573	-0.476379
C	-4.064751	-0.106354	0.234291
C	-2.997392	0.362478	-2.013672
C	-2.497627	1.879675	-0.103936
C	-1.173544	2.289278	-0.127709
C	-0.830696	3.591150	0.182343
C	-1.809229	4.502880	0.529825
C	-3.132052	4.104644	0.547874
C	-3.476128	2.805402	0.223438
C	-1.171816	-3.523349	-0.710543
C	-0.848017	-4.998674	-0.710177
C	-0.551880	-5.259466	0.757886
C	0.208744	-4.013226	1.152979
C	0.979915	-0.914433	3.346582
C	1.375183	0.516608	3.689272
C	0.093567	1.329624	3.459591
C	-0.965971	0.277726	3.198862
F	3.139648	-0.593304	-2.789895
F	1.362891	0.554356	-2.367755
F	1.402637	-1.557702	-1.959714
F	4.723038	-1.273070	-0.688816
F	3.970845	-0.873940	1.290103
F	3.057790	-2.400366	0.076313
F	-4.176218	-1.431816	0.120957
F	-5.213280	0.415342	-0.225029
F	-4.006399	0.163050	1.548850
F	-3.914927	1.233560	-2.455284
F	-1.850495	0.645163	-2.635897
F	-3.378771	-0.855519	-2.422869
Mg	-0.177766	-0.972694	0.562513
O	1.509767	-0.191308	0.334121
O	-1.788094	-0.415103	-0.154677
O	-0.335351	-2.969603	0.328482
O	-0.243634	-0.810187	2.604814
H	4.825496	0.869549	-1.848499
H	5.776282	3.112629	-1.710023
H	4.767346	4.811030	-0.226674
H	2.776641	4.227221	1.119718
H	1.813622	1.946620	0.958639
H	-0.398129	1.587918	-0.408719
H	0.209127	3.892408	0.140953
H	-1.542946	5.523652	0.777643
H	-3.909817	4.811392	0.811997
H	-4.520582	2.526876	0.232445
H	-0.935077	-3.013332	-1.642201
H	-2.211652	-3.321527	-0.451690
H	0.036350	-5.194170	-1.318869
H	-1.672269	-5.595639	-1.095797
H	-1.480534	-5.334172	1.326032
H	0.030854	-6.162910	0.927973
H	0.072545	-3.729908	2.196504
H	1.274839	-4.099255	0.936691
H	1.709226	-1.425170	2.721979
H	0.779805	-1.509384	4.241443
H	2.175734	0.854096	3.034726
H	1.725944	0.583981	4.717822
H	0.191571	1.973903	2.586654
H	-0.168052	1.958244	4.308860
H	-1.425174	-0.082277	4.123513
H	-1.744410	0.583236	2.504429

69
structure_1s.xyz

C	-2.627241	-0.369970	-0.257759
C	-2.102970	-1.270952	-1.404991
C	-3.644094	0.665516	-0.802818
C	-3.318851	-1.254186	0.787193
C	-2.805537	-1.242715	2.070681
C	-3.370811	-2.020402	3.063234
C	-4.459343	-2.822462	2.779581
C	-4.974437	-2.842173	1.497007
C	-4.409176	-2.064321	0.504331
C	2.698921	-0.583043	0.248183
C	2.305709	-0.956985	1.700521
C	4.105448	0.066627	0.213414
C	2.725529	-1.852401	-0.614602
C	1.935385	-1.870678	-1.748184
C	1.906060	-2.978477	-2.573327
C	2.676075	-4.084954	-2.273669
C	3.474595	-4.072219	-1.145162
C	3.502208	-2.964412	-0.319835
C	0.919519	2.103450	-2.789288
C	0.748945	3.353499	-3.623517
C	-0.614984	3.854986	-3.175693
C	-0.573593	3.577418	-1.691710
C	-0.702455	2.299180	2.633198
C	-0.212139	3.392791	3.552412
C	1.273355	3.430588	3.229817
C	1.278910	3.229978	1.730828
F	-1.334108	-2.238149	-0.904359
F	-1.335043	-0.568938	-2.261828
F	-3.058981	-1.852527	-2.140311
F	-4.050944	1.458142	0.194458
F	-3.085691	1.460221	-1.731543
F	-4.741117	0.139615	-1.361986
F	1.110403	-1.560783	1.692842
F	2.175662	0.136517	2.471557
F	3.161313	-1.768941	2.331205
F	4.424476	0.371561	-1.048751
F	4.146572	1.209037	0.914474
F	5.084151	-0.713635	0.693019
Mg	0.102638	1.045363	-0.041105
O	-1.589077	0.316922	0.247574
O	1.827926	0.342562	-0.191033
O	0.197283	2.371121	-1.569758
O	0.120491	2.417896	1.456950
H	-1.948060	-0.616834	2.272294
H	-2.953932	-2.002165	4.063451
H	-4.904189	-3.435048	3.554977
H	-5.825000	-3.471127	1.262141
H	-4.827438	-2.100442	-0.491493
H	1.336715	-1.000817	-1.975318
H	1.272907	-2.975663	-3.452789
H	2.654451	-4.957100	-2.916492
H	4.083796	-4.934273	-0.900321
H	4.136443	-2.979589	0.554753
H	0.462943	1.234761	-3.267806
H	1.948553	1.868095	-2.525012
H	0.801791	3.139946	-4.689372
H	1.522764	4.083456	-3.379953
H	-0.781415	4.908696	-3.392002
H	-1.410744	3.273550	-3.644057
H	-0.057680	4.372384	-1.147323
H	-1.549666	3.405747	-1.242698
H	-0.542426	1.309813	3.067479
H	-1.742185	2.388465	2.326047
H	-0.421353	3.170835	4.597245
H	-0.685233	4.343402	3.299421
H	1.791408	2.606625	3.721995
H	1.755585	4.362788	3.518253
H	1.160395	4.174794	1.195479
H	2.156535	2.706182	1.356448

69
structure_1t.xyz

6	-2.575849	-0.347522	-0.383867
6	-1.982066	-1.056064	-1.627828
6	-3.642752	0.694807	-0.805744
6	-3.226258	-1.399891	0.522085
6	-4.273200	-2.215629	0.117844
6	-4.805583	-3.147651	0.988392
6	-4.299975	-3.278288	2.268243
6	-3.253442	-2.471773	2.671841
6	-2.721105	-1.539822	1.801488
6	2.674183	-0.541053	0.260752
6	2.166565	-1.260854	1.537382
6	4.057112	0.110771	0.514740
6	2.814169	-1.568319	-0.870747
6	2.124907	-1.330737	-2.044663
6	2.196550	-2.219571	-3.100149
6	2.966456	-3.361002	-2.992882
6	3.662867	-3.603735	-1.823571
6	3.589672	-2.714902	-0.768358
6	0.996178	2.527587	-2.455963
6	0.923356	3.910279	-3.062365
6	-0.465727	4.366987	-2.645124
6	-0.561901	3.828915	-1.236224
6	-1.171328	2.293310	2.672480
6	-0.548185	1.759696	3.939681
6	0.824057	2.434529	3.952495
6	1.081337	2.797244	2.488682
9	-1.157978	-2.030446	-1.242173
9	-1.253165	-0.201834	-2.371210
9	-2.892302	-1.596023	-2.448324
9	-3.115317	1.646978	-1.592512
9	-4.694029	0.193220	-1.465654
9	-4.117213	1.309170	0.284279
9	1.038152	-1.921941	1.259374
9	1.864956	-0.380872	2.507637
9	3.026354	-2.137271	2.070589
9	5.026424	-0.753852	0.842785
9	4.456023	0.750921	-0.589016
9	4.004645	1.020413	1.499810
12	0.086723	1.135708	0.122444
8	-1.589013	0.325640	0.230794
8	1.818663	0.456578	-0.025038
8	0.201262	2.610582	-1.256930
8	-0.067795	2.335022	1.758971
1	-4.682754	-2.135531	-0.879065
1	-5.622811	-3.778654	0.659542
1	-4.719062	-4.011195	2.947624
1	-2.843357	-2.570389	3.670145
1	-1.894652	-0.909988	2.099152
1	1.522021	-0.437982	-2.120859
1	1.641720	-2.018942	-4.009143
1	3.023230	-4.062383	-3.816965
1	4.269679	-4.496297	-1.727163
1	4.142922	-2.928669	0.134874
1	0.546104	1.777589	-3.110029
1	1.996498	2.206128	-2.171965
1	1.064732	3.888937	-4.141285
1	1.686370	4.557874	-2.627059
1	-0.595508	5.447099	-2.680656
1	-1.225812	3.905209	-3.277413
1	-0.105134	4.505460	-0.510102
1	-1.576058	3.590968	-0.920427
1	-1.938366	1.663349	2.228644
1	-1.554387	3.310059	2.803343
1	-0.444628	0.676787	3.866491
1	-1.141752	1.988557	4.823010
1	1.596598	1.774780	4.341455
1	0.810128	3.333408	4.567993
1	1.166749	3.876203	2.344276
1	1.952756	2.306504	2.061937

69
structure_1u.xyz

6	-2.576915	-0.345351	-0.374656
6	-1.986143	-1.086472	-1.600916
6	-3.616762	0.712740	-0.824111
6	-3.258311	-1.369649	0.540735
6	-4.324430	-2.162792	0.141689
6	-4.884191	-3.070303	1.020938
6	-4.386869	-3.199140	2.304198
6	-3.321134	-2.415481	2.702616
6	-2.761625	-1.507814	1.823691
6	2.681258	-0.548866	0.258798
6	2.191723	-1.242423	1.556945
6	4.074494	0.093836	0.476051
6	2.792952	-1.594151	-0.859545
6	2.101739	-1.356957	-2.032305
6	2.149114	-2.259108	-3.077744
6	2.897402	-3.413916	-2.961671
6	3.597180	-3.655844	-1.794179
6	3.548355	-2.753347	-0.749161
6	-0.542307	3.824599	-1.237134
6	-0.453238	4.373082	-2.642591
6	0.918310	3.887457	-3.084450
6	0.967222	2.498334	-2.490525
6	-1.135506	2.301745	2.668041
6	-0.493767	1.776816	3.929456
6	0.874320	2.459565	3.921399
6	1.115180	2.806836	2.451132
9	-1.180519	-2.066268	-1.191032
9	-1.238631	-0.258103	-2.355289
9	-2.899479	-1.627159	-2.417347
9	-3.060043	1.642609	-1.617375
9	-4.670181	0.224027	-1.490320
9	-4.091472	1.352375	0.251175
9	1.048060	-1.891062	1.312565
9	1.923625	-0.344270	2.520277
9	3.049608	-2.123448	2.085349
9	4.049114	1.021597	1.444930
9	5.040209	-0.775618	0.802104
9	4.459805	0.709690	-0.646163
12	0.093250	1.126128	0.113692
8	-1.581668	0.315824	0.239737
8	1.829006	0.451929	-0.026766
8	0.194213	2.590678	-1.278387
8	-0.044539	2.341716	1.739387
1	-4.727889	-2.083977	-0.857767
1	-5.716474	-3.683506	0.696194
1	-4.827506	-3.912878	2.990325
1	-2.917451	-2.513097	3.703623
1	-1.920520	-0.895692	2.117271
1	1.516970	-0.453008	-2.115512
1	1.592535	-2.058137	-3.985594
1	2.935024	-4.126083	-3.777568
1	4.187821	-4.558481	-1.691387
1	4.104983	-2.966340	0.152154
1	-0.061815	4.484907	-0.511440
1	-1.557217	3.605882	-0.910178
1	-0.557489	5.456277	-2.666462
1	-1.233190	3.935714	-3.267966
1	1.043156	3.872333	-4.165504
1	1.702882	4.512796	-2.655009
1	0.489044	1.764442	-3.142965
1	1.964251	2.151769	-2.224731
1	-1.906680	1.667303	2.237888
1	-1.520081	3.317906	2.798801
1	-0.384865	0.694181	3.859382
1	-1.077082	2.006280	4.819443
1	1.654781	1.809408	4.310620
1	0.860146	3.365489	4.526523
1	1.203018	3.883911	2.294867
1	1.979407	2.308192	2.018843

69
structure_1v.xyz

C	-2.900140	-0.059502	-0.540523
C	-3.386154	0.155924	-1.996859
C	-3.601512	0.944503	0.413977
C	-3.250251	-1.490805	-0.109312
C	-4.551043	-1.930675	0.092291
C	-4.793756	-3.229970	0.495004
C	-3.744657	-4.108196	0.695013
C	-2.449292	-3.678064	0.483672
C	-2.206824	-2.377136	0.083825
C	2.627615	-0.792069	-0.443652
C	2.642430	-0.077633	-1.820671
C	2.224709	-2.278837	-0.618093
C	4.026622	-0.725900	0.177960
C	4.132565	-0.195730	1.450624
C	5.362825	-0.102350	2.073937
C	6.502939	-0.537263	1.425866
C	6.402932	-1.066990	0.152746
C	5.172742	-1.163725	-0.469272
C	-0.206241	3.223057	-1.377059
C	0.489626	4.560918	-1.475558
C	1.876054	4.241529	-0.939152
C	1.563842	3.297612	0.199004
C	-0.275511	-0.332038	3.078370
C	-0.431205	0.174927	4.492242
C	-1.281122	1.419187	4.287072
C	-0.708686	1.993178	3.009042
F	-2.725658	-0.666646	-2.813051
F	-3.150005	1.408460	-2.414035
F	-4.695464	-0.072128	-2.177984
F	-3.147078	2.193563	0.239919
F	-4.933979	1.003028	0.296843
F	-3.331834	0.617171	1.688860
F	1.393542	0.129243	-2.269965
F	3.300052	-0.726952	-2.786702
F	3.206151	1.133694	-1.699798
F	1.032037	-2.393881	-1.217875
F	3.091295	-3.009779	-1.330896
F	2.120435	-2.854387	0.584779
Mg	0.029095	0.655326	0.260484
O	-1.585799	0.198399	-0.517794
O	1.698253	-0.197471	0.324566
O	0.381976	2.589014	-0.220182
O	-0.227550	0.859316	2.269417
H	-5.388858	-1.265856	-0.063699
H	-5.814759	-3.557688	0.651410
H	-3.938714	-5.127094	1.009004
H	-1.616950	-4.357653	0.624469
H	-1.195362	-2.044038	-0.097390
H	3.228240	0.141363	1.939279
H	5.430249	0.312779	3.072905
H	7.469146	-0.464777	1.911212
H	7.290901	-1.410839	-0.364379
H	5.118182	-1.584226	-1.463822
H	-1.280351	3.279868	-1.218798
H	-0.008088	2.595166	-2.247478
H	-0.007047	5.297109	-0.840964
H	0.499271	4.937981	-2.496463
H	2.471658	3.732412	-1.697434
H	2.422304	5.120559	-0.601895
H	2.344664	2.564886	0.398498
H	1.326010	3.838668	1.117976
H	0.639564	-0.892588	2.893461
H	-1.136886	-0.926665	2.763646
H	0.541917	0.437329	4.911135
H	-0.899276	-0.562886	5.141046
H	-1.219653	2.124976	5.113457
H	-2.326447	1.145118	4.142310
H	-1.440602	2.520073	2.398774
H	0.140238	2.651856	3.206055

69
structure_1w.xyz

6	-2.561350	1.013643	-0.143545
6	-2.453729	1.532158	-1.603089
6	-2.651227	2.208325	0.840549
6	-3.825595	0.161401	0.033671
6	-5.072787	0.523093	-0.453191
6	-6.165784	-0.299075	-0.253668
6	-6.031999	-1.484722	0.443994
6	-4.795347	-1.841177	0.946621
6	-3.701955	-1.021387	0.741723
6	2.977482	0.040950	-0.323225
6	3.766057	-0.173308	-1.640351
6	3.606620	-0.790447	0.825933
6	3.021879	1.531482	0.040874
6	1.840966	2.249550	-0.014996
6	1.816394	3.594365	0.304988
6	2.977257	4.239087	0.683475
6	4.163189	3.530118	0.733467
6	4.188343	2.186783	0.412167
6	0.392009	-2.842169	2.391835
6	0.086336	-2.839835	3.871546
6	0.362082	-1.391946	4.244740
6	-0.183647	-0.640793	3.050977
6	0.655721	-3.174124	-1.695258
6	0.443312	-2.756836	-3.133905
6	-1.080033	-2.599740	-3.244790
6	-1.573912	-2.667851	-1.802011
9	-1.263031	2.086209	-1.837666
9	-3.378030	2.440325	-1.947044
9	-2.577400	0.500835	-2.455445
9	-2.656034	1.749210	2.097941
9	-1.602989	3.030949	0.724624
9	-3.755924	2.950803	0.688524
9	3.188338	0.522126	-2.623112
9	3.764232	-1.461503	-2.014280
9	5.048931	0.210298	-1.581644
9	4.916051	-0.594576	1.021441
9	2.986011	-0.495633	1.980111
9	3.442152	-2.106755	0.629882
12	0.013540	-0.801859	0.049872
8	-1.437329	0.338964	0.131540
8	1.738625	-0.432782	-0.528240
8	-0.016500	-1.542930	1.935852
8	-0.401194	-2.505698	-0.989329
1	-5.206375	1.450030	-0.992730
1	-7.131800	-0.006095	-0.647377
1	-6.891417	-2.126248	0.599014
1	-4.679405	-2.763256	1.504371
1	-2.731547	-1.291751	1.136263
1	0.928153	1.759235	-0.323113
1	0.879922	4.136113	0.253859
1	2.960493	5.292969	0.935352
1	5.081977	4.025867	1.023947
1	5.129552	1.657605	0.452289
1	1.460203	-2.959480	2.199250
1	-0.162179	-3.586857	1.821970
1	0.703761	-3.550600	4.417653
1	-0.962659	-3.087547	4.043582
1	-0.119898	-1.085882	5.171253
1	1.435386	-1.222832	4.340185
1	-1.247130	-0.418092	3.144091
1	0.352877	0.281611	2.831024
1	0.529061	-4.251582	-1.554404
1	1.601144	-2.846309	-1.271152
1	0.846850	-3.487461	-3.832547
1	0.944617	-1.805598	-3.309624
1	-1.530562	-3.392741	-3.839589
1	-1.343966	-1.648950	-3.703029
1	-2.278150	-1.885531	-1.531257
1	-2.012671	-3.640598	-1.567519

95

structure_2a.xyz

Ca	-0.076203	-0.120268	-0.083324
O	-2.199435	-0.606618	0.289986
C	-3.480141	-0.300667	0.522947
C	-3.636099	0.006606	2.038377
F	-3.080097	1.191471	2.334818
F	-4.905650	0.048202	2.481283
F	-3.009892	-0.906676	2.790204
C	-4.367739	-1.528519	0.173000
F	-4.127853	-1.917307	-1.086351
F	-5.684756	-1.289822	0.261823
F	-4.107858	-2.583224	0.960379
C	-3.986160	0.873546	-0.332270
C	-3.392509	1.031469	-1.573378
H	-2.606952	0.347368	-1.859502
C	-4.757338	2.945941	-2.027690
H	-5.055762	3.754958	-2.684125
C	-5.355837	2.795348	-0.791611
H	-6.126698	3.487149	-0.472955
C	-4.982791	1.758908	0.045993
H	-5.479662	1.658589	1.000810
O	-0.415247	-0.755613	-2.403821
C	0.830501	-1.757572	-4.081807
H	1.099893	-1.675106	-5.133465
C	-0.417209	-2.618576	-3.850044
H	-0.155513	-3.590241	-3.434473
H	-0.964650	-2.796756	-4.774499
C	-1.264048	-1.798775	-2.875234
H	-1.622719	-2.347176	-2.007596
H	-2.127888	-1.355175	-3.379037
O	0.359223	0.638381	2.177830
O	0.258425	-2.468940	0.491165
O	-0.566517	2.198739	-0.550838
O	2.049079	0.104903	-0.663915
C	-3.777879	2.051811	-2.420013
C	0.450882	-0.419115	-3.482819
H	1.689418	-2.162447	-3.547804
C	1.017953	1.874401	2.454389
C	0.371180	-0.190340	3.346227
C	-0.651621	-3.192809	1.318714
C	0.950764	-3.406695	-0.338002
C	-1.351669	3.133770	0.182297
C	-0.131419	2.900768	-1.708002
C	3.374300	0.259472	-0.568330
H	-3.306324	2.152659	-3.390979
H	-0.097810	0.202828	-4.199649
H	1.289879	0.135398	-3.070536
C	1.979764	1.559202	3.573179
H	0.274483	2.614440	2.769354
H	1.496425	2.209370	1.537035
C	1.180257	0.558993	4.395206
H	0.834889	-1.139224	3.073514
H	-0.656801	-0.376597	3.656022
H	-0.111519	-3.595780	2.183447
H	-1.422196	-2.498290	1.642577
C	-1.157272	-4.293299	0.418445
H	1.941486	-3.594865	0.081035
H	1.081629	-2.941908	-1.313351
C	0.094206	-4.674228	-0.367236
C	-0.579199	4.441158	0.086029
H	-1.477431	2.750447	1.190975
H	-2.336407	3.213690	-0.286778
C	0.237580	4.289886	-1.206787
H	0.699249	2.351510	-2.141913
H	-0.959100	2.942044	-2.423671
C	3.933781	-0.097215	0.820279
C	3.722006	1.724998	-0.950113
C	4.079633	-0.689075	-1.581910
H	2.261113	2.446155	4.139001
H	2.882160	1.100354	3.171040
H	1.812779	-0.112956	4.973167
H	0.515605	1.079209	5.086489
H	-1.582295	-5.128234	0.973149
H	-1.930454	-3.891267	-0.236012
H	0.619404	-5.495567	0.120024
H	-0.135106	-4.988914	-1.383957
H	0.080551	4.563564	0.944214
H	-1.253475	5.295792	0.059638
H	1.304903	4.346286	-1.000166
H	-0.001780	5.053470	-1.945339
C	5.063856	0.468798	1.389757
C	3.263806	-1.086184	1.520601
F	3.339853	2.565092	0.025320
F	5.026916	1.957264	-1.176608
F	3.079010	2.108844	-2.058627
F	5.416181	-0.700190	-1.474631
F	3.793867	-0.376896	-2.855437
F	3.664301	-1.947805	-1.390603
C	5.499411	0.062863	2.638863
H	5.622058	1.235207	0.870910
C	3.709932	-1.509342	2.757280
H	2.377811	-1.521755	1.081766
C	4.829134	-0.930957	3.325967
H	6.377027	0.526858	3.073276
H	3.175458	-2.292439	3.283218
H	5.177875	-1.252107	4.300394

95
structure_2b.xyz

Ca	-0.050289	-0.082856	0.123895
O	2.071191	-0.692703	0.367376
C	3.339750	-0.876508	-0.012599
C	3.371237	-1.996684	-1.090007
F	2.888824	-1.536524	-2.255015
F	4.590229	-2.502499	-1.348415
F	2.603225	-3.033536	-0.738741
C	4.176184	-1.346858	1.212153
F	4.057286	-0.458638	2.209739
F	5.487937	-1.472822	0.964280
F	3.754192	-2.526337	1.689589
C	4.009643	0.405731	-0.536813
C	3.477827	1.604562	-0.096225
H	2.608842	1.570582	0.544740
C	5.126024	2.836357	-1.318139
H	5.561378	3.780178	-1.624670
C	5.658594	1.644087	-1.771564
H	6.513853	1.649936	-2.436935
C	5.113909	0.435960	-1.374457
H	5.564515	-0.480850	-1.728448
O	0.390430	1.611637	1.808406
C	0.683416	3.847947	2.361510
H	1.049779	4.341545	1.461220
C	1.802195	3.029093	3.024692
H	1.938447	3.285295	4.074175
H	2.755169	3.185258	2.521108
C	1.338109	1.584628	2.869107
H	0.837801	1.233554	3.778147
H	2.119984	0.880694	2.597143
O	-0.647923	-1.827307	-1.463134
O	-0.511159	-1.620555	1.956245
O	0.442284	1.426380	-1.732502
O	-2.114332	0.656823	0.258466
C	4.034066	2.811658	-0.471405
C	-0.361843	2.804803	2.002729
H	0.275698	4.616027	3.016715
C	-1.288408	-1.545050	-2.709380
C	-0.832583	-3.207456	-1.121219
C	0.401073	-2.663912	2.295827
C	-1.150008	-1.171943	3.157323
C	0.912244	1.042849	-3.023472
C	0.585095	2.843472	-1.579728
C	-3.420750	0.819033	0.034122
H	3.606896	3.739113	-0.106019
H	-0.928708	3.000065	1.096687
H	-1.070536	2.647297	2.822306
C	-2.350625	-2.606515	-2.855165
H	-0.547973	-1.610627	-3.512909
H	-1.683113	-0.532424	-2.661779
C	-1.663803	-3.815640	-2.239084
H	-1.350397	-3.245608	-0.162204
H	0.145875	-3.674909	-1.014639
H	-0.144704	-3.611304	2.374831
H	1.144210	-2.722284	1.504662
C	0.958143	-2.229176	3.628774
H	-2.156956	-1.591603	3.205838
H	-1.239989	-0.089093	3.091649
C	-0.274417	-1.651642	4.316397
C	1.980185	2.055214	-3.354599
H	0.083072	1.088504	-3.737785
H	1.275489	0.020715	-2.956661
C	1.376925	3.331828	-2.786896
H	-0.405054	3.294060	-1.517103
H	1.113932	3.019192	-0.642941
C	-4.156171	-0.502908	-0.247442
C	-3.592161	1.817118	-1.144141
C	-4.087282	1.433518	1.299836
H	-2.637606	-2.762674	-3.894003
H	-3.236891	-2.329652	-2.285274
H	-2.367450	-4.556133	-1.861854
H	-1.017946	-4.300973	-2.972543
H	1.413854	-3.047242	4.184217
H	1.714340	-1.462321	3.461534
H	-0.791296	-2.424509	4.885672
H	-0.030150	-0.844197	5.004830
H	2.178398	2.117093	-4.423617
H	2.906097	1.799018	-2.839745
H	0.709795	3.793917	-3.515717
H	2.131808	4.064377	-2.506269
C	-5.299465	-0.627576	-1.021008
C	-3.637647	-1.634744	0.358333
F	-3.259607	1.232263	-2.306130
F	-4.837087	2.304488	-1.295815
F	-2.790441	2.878864	-1.008025
F	-5.424905	1.511162	1.225642
F	-3.640460	2.671237	1.561177
F	-3.801108	0.683846	2.371659
C	-5.898516	-1.863018	-1.193136
H	-5.740166	0.234885	-1.501259
C	-4.247489	-2.864794	0.207705
H	-2.737152	-1.534807	0.947147
C	-5.380477	-2.985228	-0.575080
H	-6.784274	-1.943921	-1.812159
H	-3.830061	-3.736538	0.699108
H	-5.857578	-3.949500	-0.705160

95
structure_2c.xyz

Ca	0.047506	-0.058283	0.089052
O	-2.071863	0.397302	0.543605
C	-3.392330	0.539085	0.384838
C	-3.685865	2.031044	0.065934
F	-3.283167	2.330532	-1.179184
F	-4.979782	2.386587	0.154388
F	-3.020100	2.848018	0.890454
C	-4.116066	0.172950	1.714016
F	-3.748347	-1.052740	2.108682
F	-5.453810	0.165400	1.618772
F	-3.802455	1.011904	2.713170
C	-3.981613	-0.383952	-0.696338
C	-3.341550	-1.596795	-0.887425
H	-2.455965	-1.810646	-0.306575
C	-4.933620	-2.223355	-2.562584
H	-5.304301	-2.935921	-3.290020
C	-5.574168	-1.011989	-2.385142
H	-6.450185	-0.769045	-2.974898
C	-5.110635	-0.103300	-1.450175
H	-5.645705	0.827046	-1.322502
O	-0.346451	-2.409487	0.639720
C	1.047281	-4.011215	1.548451
H	1.458152	-5.014393	1.446984
C	-0.199342	-3.962234	2.426830
H	0.037641	-3.771688	3.472230
H	-0.740438	-4.907201	2.379987
C	-1.039973	-2.839855	1.814460
H	-1.152805	-1.971509	2.460598
H	-2.038437	-3.184392	1.537315
O	0.647044	2.211903	-0.523563
O	0.403407	0.447426	2.443379
O	-0.405430	-0.377431	-2.283487
O	2.155674	-0.727468	0.016080
C	-3.816008	-2.515159	-1.803350
C	0.542331	-3.452184	0.241073
H	1.831016	-3.359213	1.933448
C	1.445089	2.658210	-1.620602
C	0.348178	3.328389	0.324456
C	-0.460682	1.245994	3.246053
C	1.218617	-0.301367	3.342029
C	-1.045833	0.602430	-3.100492
C	-0.460200	-1.653839	-2.933273
C	3.441340	-0.605650	-0.330724
H	-3.304759	-3.462956	-1.929396
H	-0.015429	-4.203607	-0.330201
H	1.316771	-3.004398	-0.375688
C	0.992336	4.079894	-1.843550
H	1.271858	1.984723	-2.455563
H	2.502368	2.615332	-1.347604
C	0.842805	4.567437	-0.410079
H	0.849409	3.188471	1.283747
H	-0.728105	3.336529	0.486115
H	0.101518	2.105732	3.629337
H	-1.277909	1.583690	2.614040
C	-0.883007	0.317301	4.364273
H	2.101523	0.290767	3.599927
H	1.551518	-1.192824	2.815985
C	0.351671	-0.566467	4.574679
C	-2.061697	-0.169869	-3.904813
H	-0.301446	1.068761	-3.754913
H	-1.473814	1.356595	-2.444152
C	-1.313988	-1.466315	-4.180288
H	0.555212	-1.977530	-3.160674
H	-0.906827	-2.360608	-2.232995
C	4.009954	0.800289	-0.070318
C	3.572313	-0.981484	-1.833077
C	4.290979	-1.599867	0.508701
H	0.031453	4.092355	-2.361748
H	1.709471	4.661776	-2.420429
H	1.814405	4.875184	-0.021398
H	0.152888	5.403370	-0.308545
H	-1.175366	0.859456	5.262047
H	-1.737179	-0.274577	4.037982
H	0.889598	-0.298777	5.483079
H	0.078716	-1.617224	4.656071
H	-2.353987	0.353421	-4.814061
H	-2.952143	-0.352866	-3.304933
H	-0.679801	-1.360255	-5.061583
H	-1.982184	-2.310421	-4.342788
C	5.061164	1.370184	-0.771621
C	3.412279	1.531411	0.941730
F	3.055254	-0.011114	-2.601464
F	4.831052	-1.191174	-2.259550
F	2.892854	-2.098706	-2.119335
F	5.613814	-1.478693	0.320567
F	3.976633	-2.878565	0.252283
F	4.064417	-1.398213	1.813505
C	5.478988	2.657775	-0.485957
H	5.563745	0.822861	-1.556696
C	3.839908	2.809761	1.241527
H	2.586289	1.087335	1.477730
C	4.870234	3.383716	0.519942
H	6.291378	3.093466	-1.055619
H	3.360560	3.364884	2.040001
H	5.201539	4.391037	0.743757

95
structure_2d.xyz

Ca	-0.024404	0.189124	0.012637
O	-2.158482	0.361963	-0.550423
C	-3.458769	0.047475	-0.528665
C	-3.729337	-0.959617	-1.679798
F	-3.186537	-2.153097	-1.390121
F	-5.027776	-1.175404	-1.954694
F	-3.170037	-0.554842	-2.826709
C	-4.303266	1.327972	-0.787300
F	-3.975671	2.276464	0.099929
F	-5.626008	1.133498	-0.677819
F	-4.083574	1.845752	-2.004953
C	-3.918897	-0.521966	0.824257
C	-3.272441	-0.033809	1.948163
H	-2.487519	0.697355	1.818697
C	-4.586644	-1.445663	3.367841
H	-4.844374	-1.809054	4.355734
C	-5.238428	-1.936099	2.253200
H	-6.010834	-2.688361	2.361953
C	-4.916999	-1.468634	0.990799
H	-5.454689	-1.863501	0.140513
O	-0.338636	1.922158	1.688020
C	0.996871	3.610216	2.534280
H	1.353853	4.085351	3.446561
C	-0.264424	4.269784	1.971176
H	-0.031900	4.943005	1.147860
H	-0.782435	4.852158	2.732162
C	-1.133692	3.094185	1.517070
H	-1.435392	3.133431	0.472883
H	-2.036572	3.006386	2.127234
O	0.473016	-1.626214	-1.512672
O	0.261507	1.890640	-1.700665
O	-0.455560	-1.632664	1.547666
O	2.105674	0.419537	0.570941
C	-3.606729	-0.482402	3.210236
C	0.564297	2.178988	2.760041
H	1.807897	3.627243	1.806928
C	1.275733	-2.769935	-1.214273
C	0.128681	-1.634285	-2.904195
C	-0.653006	2.106663	-2.772632
C	1.022146	3.087764	-1.533753
C	-1.274767	-2.776087	1.318354
C	-0.072476	-1.703521	2.914648
C	3.412220	0.132315	0.553248
H	-3.094994	-0.079782	4.077005
H	0.033517	2.061231	3.711987
H	1.374853	1.458670	2.685903
C	0.837705	-3.800348	-2.225708
H	1.097958	-3.039094	-0.175390
H	2.331771	-2.517361	-1.336180
C	0.673350	-2.937985	-3.467893
H	0.567655	-0.756872	-3.382057
H	-0.955621	-1.569536	-2.976258
H	-0.125348	1.985318	-3.726059
H	-1.444728	1.367541	-2.681159
C	-1.113298	3.531294	-2.570501
H	1.957459	2.999270	-2.091807
H	1.268676	3.170997	-0.477398
C	0.152016	4.228109	-2.069768
C	-0.618255	-3.900876	2.111742
H	-1.318716	-2.943846	0.244806
H	-2.284102	-2.567058	1.681453
C	0.250755	-3.169959	3.146305
H	0.769904	-1.033229	3.063876
H	-0.914676	-1.378327	3.533830
C	3.891974	-0.426513	-0.798031
C	3.695533	-0.877164	1.699456
C	4.227278	1.428667	0.820856
H	-0.116462	-4.241298	-1.929674
H	1.567785	-4.598320	-2.351467
H	1.645925	-2.772533	-3.933292
H	0.005467	-3.371071	-4.210515
H	-1.510865	3.975377	-3.481672
H	-1.899206	3.549153	-1.815932
H	0.663259	4.743277	-2.882378
H	-0.068090	4.967686	-1.301840
H	-0.006437	-4.532110	1.468216
H	-1.370907	-4.536099	2.576204
H	1.307869	-3.352939	2.961158
H	0.030935	-3.471678	4.169186
C	4.941413	-1.317757	-0.958888
C	3.207741	0.003056	-1.922844
F	3.186043	-2.079982	1.391727
F	4.995485	-1.063057	1.988950
F	3.113401	-0.496387	2.843680
F	5.554722	1.263702	0.719283
F	3.989134	1.936894	2.039240
F	3.884010	2.371899	-0.065859
C	5.272563	-1.793864	-2.215143
H	5.511081	-1.662640	-0.107672
C	3.548773	-0.456030	-3.179478
H	2.385984	0.693308	-1.797864
C	4.577677	-1.367414	-3.330294
H	6.085281	-2.503103	-2.318993
H	3.003111	-0.103804	-4.047564
H	4.841044	-1.739318	-4.313585

95
structure_2e.xyz

Ca	-0.002641	-0.103165	0.143903
O	-2.078498	0.636990	0.280271
C	-3.405517	0.738645	0.150766
C	-3.710592	1.863094	-0.876831
F	-3.390857	1.460672	-2.116953
F	-4.994131	2.263965	-0.913930
F	-2.988141	2.962359	-0.632851
C	-4.030814	1.143090	1.518227
F	-3.642613	0.282198	2.467521
F	-5.372616	1.144116	1.519930
F	-3.641042	2.362589	1.918211
C	-4.081152	-0.581303	-0.262996
C	-3.481119	-1.746826	0.182745
H	-2.570284	-1.672921	0.758606
C	-5.172893	-3.074774	-0.869691
H	-5.597310	-4.042707	-1.109110
C	-5.775474	-1.917842	-1.324899
H	-6.675683	-1.973996	-1.925527
C	-5.242569	-0.679314	-1.013469
H	-5.748937	0.205906	-1.370953
O	-0.420276	-1.875752	1.769287
C	1.091559	-2.687839	3.312486
H	1.569817	-3.567059	3.741106
C	-0.132153	-2.228988	4.097726
H	0.117391	-1.523023	4.888218
H	-0.629763	-3.080971	4.561524
C	-1.034970	-1.602106	3.034144
H	-1.128546	-0.521159	3.122215
H	-2.038998	-2.031300	3.044625
O	0.595534	1.621418	-1.464990
O	0.395718	1.344966	2.050394
O	-0.568645	-1.581900	-1.694522
O	2.124145	-0.716009	0.367252
C	-4.024126	-2.983177	-0.106416
C	0.510001	-2.944678	1.944697
H	1.833913	-1.893575	3.234178
C	1.294875	1.543303	-2.707293
C	0.359139	2.998076	-1.142151
C	-0.373736	2.477823	2.438668
C	1.393882	1.203052	3.054887
C	-1.209504	-1.099188	-2.875486
C	-0.720087	-3.004578	-1.609717
C	3.369812	-0.817828	-0.108974
H	-3.541738	-3.881764	0.261623
H	-0.025434	-3.900361	1.904892
H	1.237814	-2.884120	1.139964
C	0.837875	2.766994	-3.462640
H	1.037865	0.597731	-3.177626
H	2.372303	1.565617	-2.523610
C	0.808377	3.805405	-2.351607
H	0.924269	3.251725	-0.243610
H	-0.703606	3.105581	-0.935761
H	-0.981961	2.780382	1.590913
H	-1.043211	2.187813	3.255387
C	0.659784	3.492775	2.900920
H	2.172488	0.557026	2.657853
H	0.942797	0.727656	3.932900
C	1.837911	2.625971	3.373413
C	-2.293043	-2.107774	-3.167419
H	-0.478215	-1.060542	-3.690062
H	-1.578683	-0.096448	-2.669425
C	-1.614767	-3.411244	-2.772289
H	0.265774	-3.466704	-1.653646
H	-1.173501	-3.230346	-0.644128
C	3.963493	0.531975	-0.549442
C	3.353446	-1.820518	-1.296625
C	4.294644	-1.378949	1.006560
H	-0.161753	2.605574	-3.870520
H	1.510651	3.030638	-4.277203
H	1.811052	4.203505	-2.190659
H	0.137378	4.637662	-2.556981
H	0.960927	4.132734	2.071697
H	0.265628	4.135044	3.686827
H	2.749710	2.875243	2.832293
H	2.042805	2.744616	4.436209
H	-2.604515	-2.089788	-4.210793
H	-3.161947	-1.914859	-2.539507
H	-1.013174	-3.793061	-3.598451
H	-2.325228	-4.184140	-2.483621
C	5.006278	0.678258	-1.451353
C	3.408473	1.667480	0.013665
F	2.758445	-1.264769	-2.362228
F	4.563371	-2.246080	-1.701994
F	2.654254	-2.921576	-0.996273
F	5.589176	-1.445635	0.661774
F	3.929998	-2.608858	1.395148
F	4.224201	-0.591849	2.090002
C	5.459232	1.937715	-1.801114
H	5.476052	-0.187090	-1.897452
C	3.871577	2.925206	-0.318530
H	2.587828	1.546559	0.705543
C	4.895003	3.066229	-1.237186
H	6.265495	2.034025	-2.518710
H	3.426404	3.802047	0.138723
H	5.255620	4.051266	-1.509400

95

structure_2f.xyz

Ca	-0.001657	0.000727	0.067560
O	-2.141622	0.518746	0.034253
C	-3.425682	0.723750	-0.274019
C	-3.504405	1.429015	-1.656008
F	-2.922639	0.658107	-2.586472
F	-4.750671	1.680232	-2.086019
F	-2.855552	2.600785	-1.665129
C	-4.032085	1.649235	0.815661
F	-4.073051	0.996011	1.988069
F	-5.279629	2.081411	0.567705
F	-3.287366	2.744252	1.009932
C	-4.257462	-0.571872	-0.358369
C	-3.571756	-1.768833	-0.267538
H	-2.499028	-1.734024	-0.147785
C	-5.610347	-3.004931	-0.494406
H	-6.138430	-3.950000	-0.544529
C	-6.304019	-1.813218	-0.592876
H	-7.379948	-1.819941	-0.722279
C	-5.635060	-0.605198	-0.528339
H	-6.202698	0.309955	-0.614049
O	-0.478793	-1.509003	1.923548
C	-0.025154	-2.258594	4.126392
H	-0.229988	-3.314383	4.306512
C	-1.328886	-1.473425	4.075778
H	-1.161914	-0.421357	4.311272
H	-2.088128	-1.859955	4.753727
C	-1.716262	-1.617105	2.624163
H	-2.380262	-0.846287	2.241595
H	-2.163354	-2.598309	2.428976
O	0.387159	1.975115	-1.297078
O	0.475818	1.540599	1.898590
O	-0.387777	-1.996810	-1.262053
O	2.136719	-0.518498	0.030146
C	-4.238610	-2.977676	-0.333863
C	0.565790	-2.062484	2.732369
H	0.650583	-1.915132	4.908212
C	0.880501	2.056101	-2.637861
C	0.097566	3.282022	-0.791216
C	-0.569252	2.114789	2.692263
C	1.711961	1.664047	2.598622
C	-0.861980	-2.104597	-2.607525
C	-0.091015	-3.292373	-0.730835
C	3.422392	-0.729101	-0.266886
H	-3.681464	-3.904749	-0.256517
H	0.901788	-3.001233	2.290399
H	1.401588	-1.364553	2.710614
C	1.167754	3.523101	-2.874210
H	0.105623	1.684634	-3.314525
H	1.762417	1.422896	-2.717346
C	0.121574	4.193239	-1.998341
H	0.872210	3.554637	-0.067738
H	-0.865041	3.242618	-0.284914
H	-0.899918	3.045573	2.229870
H	-1.407861	1.420027	2.681629
C	0.018078	2.337905	4.083860
H	2.159472	2.640710	2.382785
H	2.376835	0.885160	2.234262
C	1.322198	1.552187	4.052422
C	-1.149508	-3.575482	-2.816182
H	-0.076886	-1.749619	-3.281411
H	-1.741076	-1.471045	-2.712493
C	-0.110071	-4.228304	-1.919442
H	0.871833	-3.238568	-0.226003
H	-0.864097	-3.553412	-0.001530
C	4.256560	0.564070	-0.361149
C	3.509113	-1.452550	-1.639074
C	4.020235	-1.641298	0.838621
H	1.090706	3.793145	-3.926084
H	2.173294	3.768312	-2.528118
H	0.370933	5.219847	-1.734257
H	-0.850539	4.188860	-2.492765
H	0.222070	3.397034	4.244125
H	-0.659546	2.009319	4.870429
H	2.080234	1.953755	4.722935
H	1.155245	0.505550	4.310852
H	-1.065111	-3.867139	-3.861714
H	-2.157832	-3.812255	-2.472336
H	0.864831	-4.236458	-2.408185
H	-0.362949	-5.248662	-1.635252
C	5.634781	0.593204	-0.526424
C	3.572958	1.763079	-0.282570
F	2.937760	-0.692010	-2.584334
F	4.757705	-1.714291	-2.055998
F	2.855655	-2.621806	-1.637809
F	5.268714	-2.078484	0.604697
F	3.272153	-2.732475	1.041764
F	4.054530	-0.973183	2.002790
C	6.306431	1.799285	-0.598531
H	6.200806	-0.323826	-0.602279
C	4.242593	2.969984	-0.355459
H	2.499684	1.730989	-0.166533
C	5.614943	2.993166	-0.511547
H	7.382799	1.802804	-0.724350
H	3.687353	3.898864	-0.286359
H	6.145165	3.936739	-0.566953

95
structure_2g.xyz

Ca	-0.000131	0.213514	0.001519
O	2.138722	0.382667	0.543165
C	3.441261	0.081662	0.486879
C	3.754820	-0.917214	1.634339
F	3.223889	-2.119796	1.361736
F	5.062805	-1.111568	1.877663
F	3.217750	-0.517339	2.793712
C	4.277525	1.372960	0.717490
F	3.908933	2.316970	-0.158357
F	5.598264	1.195387	0.565042
F	4.090715	1.888350	1.941819
C	3.870734	-0.489921	-0.875285
C	3.197336	-0.006644	-1.985366
H	2.413709	0.723041	-1.840947
C	4.483025	-1.417787	-3.431643
H	4.719363	-1.782564	-4.424352
C	5.161223	-1.903896	-2.330954
H	5.933098	-2.654250	-2.455484
C	4.867164	-1.434536	-1.062637
H	5.425335	-1.826366	-0.224205
O	0.264431	1.926399	-1.701217
C	-1.111020	3.572067	-2.564201
H	-1.496476	4.020112	-3.478581
C	0.150328	4.265433	-2.044995
H	-0.076688	4.969011	-1.245823
H	0.651037	4.821479	-2.836705
C	1.038202	3.116867	-1.558295
H	1.333967	3.189772	-0.513938
H	1.945504	3.029088	-2.161536
O	-0.403855	-1.596390	1.553471
O	-0.263904	1.955014	1.675560
O	0.407122	-1.604635	-1.533026
O	-2.136891	0.367029	-0.550137
C	3.504036	-0.457185	-3.253762
C	-0.656858	2.144503	-2.766565
H	-1.905921	3.591047	-1.819128
C	-1.230975	-2.735012	1.335918
C	0.017170	-1.684688	2.908569
C	0.658323	2.176723	2.739643
C	-1.029575	3.148960	1.515830
C	1.223721	-2.750516	-1.315406
C	-0.015351	-1.687290	-2.887951
C	-3.441116	0.074865	-0.487478
H	2.971358	-0.058060	-4.109444
H	-0.136958	2.015842	-3.723049
H	-1.453300	1.411579	-2.665590
C	-0.510184	-3.871372	2.044066
H	-1.345418	-2.864273	0.262727
H	-2.215326	-2.548740	1.774632
C	0.300645	-3.163642	3.140644
H	-0.795290	-1.331264	3.551145
H	0.884482	-1.042088	3.032523
H	0.138356	2.056707	3.697219
H	1.451961	1.440076	2.643391
C	1.117035	3.600916	2.527145
H	-1.949560	3.065911	2.099897
H	-1.303445	3.221809	0.465414
C	-0.146866	4.295229	2.017510
C	0.491411	-3.879420	-2.023027
H	1.336625	-2.880817	-0.242136
H	2.209759	-2.573769	-1.754362
C	-0.304946	-3.164565	-3.125478
H	-0.880593	-1.041288	-3.008485
H	0.797457	-1.334316	-3.530137
C	-3.871140	-0.473730	0.883935
C	-3.762453	-0.939051	-1.619483
C	-4.269978	1.367579	-0.735778
H	0.151687	-4.396658	1.355957
H	-1.215578	-4.597623	2.444811
H	0.000449	-3.468407	4.142037
H	1.363709	-3.370535	3.032355
H	1.512696	4.051870	3.435728
H	1.904690	3.613333	1.774140
H	-0.649266	4.836334	2.818554
H	0.075444	5.012156	1.228905
H	-0.180834	-4.392508	-1.335758
H	1.188299	-4.617004	-2.417891
H	-1.370079	-3.366260	-3.028484
H	0.003740	-3.468719	-4.124481
C	-4.871625	-1.410607	1.087599
C	-3.192711	0.022719	1.985180
F	-3.237678	-2.140436	-1.330398
F	-5.072062	-1.129637	-1.857146
F	-3.225696	-0.559306	-2.785729
F	-5.591601	1.199884	-0.580374
F	-4.080802	1.864751	-1.967270
F	-3.895448	2.321506	0.126702
C	-5.164179	-1.860272	2.363384
H	-5.434065	-1.811687	0.256405
C	-3.497435	-0.408401	3.260693
H	-2.406991	0.747682	1.828608
C	-4.480241	-1.361954	3.455019
H	-5.939247	-2.605042	2.500896
H	-2.960311	0.000491	4.108945
H	-4.715211	-1.711640	4.453475

95

structure_2h.xyz

Ca	0.087699	0.051507	0.159000
O	2.154568	-0.700708	0.279839
C	3.448557	-0.785626	-0.041273
C	3.563311	-1.612810	-1.351201
F	3.110022	-0.896605	-2.391498
F	4.807732	-2.016783	-1.665610
F	2.817818	-2.723316	-1.296087
C	4.208348	-1.530598	1.091625
F	4.002658	-0.908550	2.259447
F	5.537135	-1.584707	0.908542
F	3.784640	-2.793840	1.244300
C	4.122830	0.588492	-0.201917
C	3.581693	1.629929	0.533026
H	2.723643	1.428279	1.158149
C	5.178449	3.160602	-0.385596
H	5.586479	4.161692	-0.462554
C	5.728790	2.126141	-1.117416
H	6.571834	2.311837	-1.772532
C	5.213868	0.845694	-1.017111
H	5.673729	0.054573	-1.592409
O	0.515205	1.253837	2.231728
C	-0.919629	1.501735	4.022607
H	-1.374122	2.191487	4.731984
C	0.343336	0.836147	4.560215
H	0.134584	-0.095741	5.083247
H	0.855532	1.497575	5.259237
C	1.202586	0.614462	3.313616
H	1.330390	-0.431953	3.042481
H	2.194986	1.057732	3.418891
O	-0.381077	-1.091601	-1.947943
O	-0.345287	-1.921861	1.511586
O	0.780532	2.017443	-1.094657
O	-2.041365	0.624274	0.467746
C	4.106997	2.904121	0.450121
C	-0.410146	2.190109	2.780123
H	-1.666486	0.761273	3.736269
C	-0.843673	-0.468647	-3.143557
C	-0.499208	-2.512053	-2.090043
C	0.443396	-3.108006	1.504516
C	-1.278106	-2.097826	2.570613
C	1.600799	2.056303	-2.264146
C	0.643659	3.347108	-0.573388
C	-3.326338	0.812803	0.146631
H	3.672184	3.704818	1.037802
H	0.115718	3.121181	3.021821
H	-1.175670	2.373880	2.030450
C	-1.929308	-1.386309	-3.648136
H	-0.018193	-0.404273	-3.861611
H	-1.182899	0.532708	-2.889885
C	-1.336306	-2.754927	-3.342429
H	-0.981555	-2.889834	-1.188630
H	0.497931	-2.945420	-2.164519
H	1.005249	-3.137365	0.575581
H	1.157907	-3.062347	2.332501
C	-0.567043	-4.227092	1.698118
H	-2.079380	-1.377459	2.432168
H	-0.772622	-1.895640	3.521192
C	-1.708189	-3.557565	2.480340
C	1.329501	3.417688	-2.854802
H	1.319625	1.220047	-2.899195
H	2.650206	1.946683	-1.980396
C	1.276483	4.276475	-1.600205
H	-0.419334	3.536794	-0.436978
H	1.140496	3.399287	0.396651
C	-3.956794	-0.386703	-0.583734
C	-3.426000	2.100395	-0.716930
C	-4.155815	1.024257	1.446140
H	-2.139784	-1.241247	-4.706698
H	-2.845834	-1.222795	-3.081523
H	-2.099713	-3.513265	-3.178671
H	-0.699973	-3.086238	-4.163875
H	-0.921929	-4.588757	0.732983
H	-0.131024	-5.072953	2.227193
H	-2.654516	-3.647674	1.948723
H	-1.843917	-3.988837	3.470904
H	0.367063	3.420165	-3.370042
H	2.102818	3.730682	-3.554546
H	0.701619	5.191954	-1.728920
H	2.287950	4.545835	-1.294397
C	-5.076888	-0.318667	-1.398034
C	-3.370712	-1.621101	-0.365319
F	-2.899559	1.883220	-1.932241
F	-4.671677	2.567079	-0.910623
F	-2.734339	3.107816	-0.171652
F	-5.473083	1.161491	1.235402
F	-3.756261	2.102380	2.134349
F	-4.004400	-0.032055	2.258403
C	-5.581760	-1.461325	-1.992801
H	-5.572036	0.624416	-1.580619
C	-3.887869	-2.766838	-0.936752
H	-2.491244	-1.665620	0.260271
C	-4.994366	-2.690550	-1.761241
H	-6.449739	-1.387027	-2.637230
H	-3.418808	-3.725201	-0.742916
H	-5.398666	-3.584527	-2.221285

95
structure_2i.xyz

Ca	0.008933	0.215472	-0.030209
O	2.151076	0.503081	0.444503
C	3.450332	0.182719	0.457119
C	3.726293	-0.649511	1.739152
F	3.168741	-1.866091	1.628417
F	5.024716	-0.839424	2.031423
F	3.183794	-0.076851	2.820815
C	4.298389	1.484879	0.519286
F	3.995187	2.276868	-0.517265
F	5.621694	1.271260	0.470264
F	4.056633	2.195708	1.631116
C	3.896570	-0.578720	-0.802987
C	3.237918	-0.259516	-1.979419
H	2.450788	0.480057	-1.948308
C	4.546388	-1.856114	-3.193746
H	4.797755	-2.356146	-4.121749
C	5.208409	-2.180786	-2.025958
H	5.982513	-2.939108	-2.032384
C	4.895962	-1.537742	-0.840763
H	5.442903	-1.804822	0.052265
O	0.277072	1.721968	-1.917719
C	-1.048820	3.327652	-2.936935
H	-1.367515	3.700488	-3.908970
C	0.213404	4.025111	-2.413846
H	-0.021247	4.705195	-1.596349
H	0.708344	4.607193	-3.189907
C	1.110416	2.877273	-1.949643
H	1.538540	3.001519	-0.957763
H	1.929293	2.705679	-2.654818
O	-0.392050	-1.433024	1.695568
O	-0.324120	2.072099	1.501088
O	0.427107	-1.767087	-1.346058
O	-2.124085	0.305004	-0.613320
C	3.563744	-0.883036	-3.167110
C	-0.651064	1.866568	-2.988903
H	-1.880140	3.451196	-2.243636
C	-1.230782	-2.578844	1.581155
C	0.020601	-1.397836	3.055446
C	0.546249	2.341116	2.612356
C	-1.004478	3.268589	1.129207
C	1.239824	-2.884788	-1.003233
C	0.009029	-1.992882	-2.686171
C	-3.429684	0.023922	-0.529421
H	3.043614	-0.610177	-4.078280
H	-0.147383	1.618606	-3.930429
H	-1.472535	1.178747	-2.806735
C	-0.530948	-3.650490	2.402697
H	-1.337619	-2.809821	0.524434
H	-2.216324	-2.340217	1.990382
C	0.289853	-2.850640	3.425937
H	-0.792888	-0.980142	3.657311
H	0.892583	-0.753258	3.125205
H	0.181523	1.793500	3.483666
H	1.531675	1.967143	2.339790
C	0.512352	3.849262	2.824633
H	-1.944417	3.346363	1.685333
H	-1.238603	3.202884	0.068608
C	-0.047662	4.372806	1.510166
C	0.509592	-4.081620	-1.591499
H	1.345745	-2.900583	0.078621
H	2.228948	-2.757625	-1.452094
C	-0.281397	-3.486772	-2.766648
H	-0.855100	-1.362318	-2.877481
H	0.824141	-1.710633	-3.359561
C	-3.868871	-0.385977	0.886819
C	-3.747389	-1.095483	-1.558159
C	-4.253943	1.287654	-0.908404
H	0.122604	-4.254946	1.774294
H	-1.250514	-4.319575	2.871907
H	-0.009665	-3.056439	4.452428
H	1.350580	-3.077014	3.334917
H	-0.157779	4.107392	3.645895
H	1.498934	4.246740	3.055705
H	-0.539311	5.339587	1.607176
H	0.743628	4.459014	0.764032
H	-0.166077	-4.519622	-0.857260
H	1.207764	-4.856752	-1.903478
H	-1.347118	-3.676172	-2.653182
H	0.030798	-3.895506	-3.726476
C	-4.865088	-1.304655	1.175906
C	-3.202287	0.223266	1.937734
F	-3.226406	-2.263544	-1.149772
F	-5.056184	-1.306476	-1.782729
F	-3.203810	-0.832889	-2.753079
F	-5.576494	1.139129	-0.741694
F	-4.059597	1.659452	-2.182753
F	-3.879541	2.322097	-0.144372
C	-5.164857	-1.626828	2.488135
H	-5.418350	-1.791017	0.385083
C	-3.514372	-0.080976	3.247643
H	-2.419232	0.934008	1.716035
C	-4.492428	-1.017502	3.529376
H	-5.936259	-2.359623	2.693764
H	-2.986597	0.414352	4.054751
H	-4.732777	-1.267679	4.556060

95

structure_2j.xyz

Ca	0.029152	-0.107559	0.061984
O	-2.069431	0.581532	0.051284
C	-3.386525	0.739056	0.213529
C	-3.871143	1.883208	-0.721334
F	-3.796896	1.486791	-2.000579
F	-5.138531	2.275459	-0.510542
F	-3.110498	2.979618	-0.615288
C	-3.640337	1.135139	1.693355
F	-3.195598	0.160931	2.498820
F	-4.923049	1.351491	2.024207
F	-2.974831	2.252742	2.022911
C	-4.212449	-0.519046	-0.118814
C	-3.527520	-1.623031	-0.591005
H	-2.455393	-1.553512	-0.703564
C	-5.566553	-2.866915	-0.770068
H	-6.093708	-3.780514	-1.018879
C	-6.262067	-1.763380	-0.312469
H	-7.339404	-1.806661	-0.203590
C	-5.592773	-0.597803	0.010452
H	-6.162553	0.249713	0.362561
O	-0.510630	-2.347676	0.829481
C	0.514186	-4.055467	2.024234
H	0.858294	-5.088175	1.997740
C	-0.900493	-3.907197	2.564284
H	-0.947174	-3.888578	3.652005
H	-1.523250	-4.732437	2.215506
C	-1.369861	-2.594857	1.953024
H	-1.268330	-1.752550	2.637301
H	-2.403107	-2.630639	1.609049
O	0.647008	2.081197	-0.719108
O	0.352452	0.380308	2.431548
O	-0.298521	-0.740710	-2.249973
O	2.144905	-0.808041	0.100652
C	-4.194483	-2.791056	-0.909873
C	0.376534	-3.454679	0.646881
H	1.223216	-3.470960	2.612221
C	1.365905	2.464036	-1.887067
C	0.170434	3.263648	-0.073399
C	-0.073092	1.455016	3.259314
C	1.239984	-0.399415	3.231428
C	-1.040747	0.088760	-3.138075
C	-0.111536	-1.971691	-2.935876
C	3.418462	-0.705312	-0.299311
H	-3.636647	-3.648918	-1.268194
H	-0.078045	-4.167886	-0.049892
H	1.303293	-3.064060	0.231336
C	0.585612	3.647680	-2.407470
H	1.398661	1.606261	-2.554138
H	2.389636	2.739555	-1.615342
C	0.225411	4.378337	-1.118212
H	0.814292	3.483574	0.782163
H	-0.838409	3.050461	0.275521
H	-0.529297	2.212126	2.628216
H	-0.826079	1.088118	3.964527
C	1.191470	1.897759	3.970389
H	1.865244	-0.972020	2.551853
H	0.646511	-1.079648	3.851217
C	2.004114	0.602038	4.095491
C	-2.058670	-0.836457	-3.788038
H	-0.354531	0.508977	-3.881845
H	-1.481671	0.888837	-2.549608
C	-1.433423	-2.231638	-3.644703
H	0.711426	-1.859060	-3.647669
H	0.164013	-2.728078	-2.204907
C	3.959160	0.733196	-0.230550
C	3.517773	-1.268996	-1.743945
C	4.315098	-1.567605	0.633085
H	-0.313892	3.301534	-2.918694
H	1.162757	4.261640	-3.097234
H	1.001288	5.100252	-0.862229
H	-0.720581	4.911596	-1.190673
H	1.724930	2.626998	3.360710
H	0.977231	2.360537	4.932367
H	3.021331	0.736314	3.731884
H	2.064164	0.255841	5.126226
H	-2.235083	-0.561558	-4.826806
H	-3.008451	-0.783415	-3.259651
H	-1.276271	-2.723324	-4.603560
H	-2.072416	-2.874939	-3.042066
C	4.975007	1.236557	-1.027553
C	3.382432	1.564087	0.715160
F	2.942326	-0.422400	-2.611833
F	4.768212	-1.490057	-2.187970
F	2.872413	-2.435103	-1.856188
F	5.623616	-1.497900	0.348827
F	3.974421	-2.863953	0.604357
F	4.175002	-1.159566	1.902902
C	5.383343	2.552250	-0.896639
H	5.460004	0.612739	-1.765177
C	3.800695	2.871557	0.860636
H	2.583937	1.168461	1.326790
C	4.799436	3.374966	0.047138
H	6.169494	2.932898	-1.537991
H	3.341600	3.506166	1.610115
H	5.124085	4.403666	0.151022

95
structure_2k.xyz

Ca	0.000224	0.000660	0.000402
O	2.083565	0.679863	-0.281258
C	3.417872	0.592166	-0.238465
C	4.004558	1.942470	0.263924
F	3.472224	2.258433	1.451807
F	5.336024	1.923819	0.426859
F	3.724838	2.959502	-0.564499
C	3.927002	0.340794	-1.683672
F	3.589532	-0.896461	-2.084288
F	5.256067	0.457730	-1.849127
F	3.369286	1.188871	-2.556086
C	3.916718	-0.500406	0.724236
C	3.167088	-0.691887	1.874168
H	2.289356	-0.080564	2.027339
C	4.623318	-2.455678	2.582423
H	4.896560	-3.219719	3.300770
C	5.378373	-2.270611	1.440984
H	6.248766	-2.889900	1.258574
C	5.035200	-1.292942	0.523013
H	5.654514	-1.168350	-0.353787
O	0.599926	-2.272245	0.528551
C	-0.291647	-4.408386	0.410558
H	-0.458548	-5.311590	0.995255
C	1.049975	-4.418105	-0.333794
H	0.907868	-4.570794	-1.402981
H	1.706467	-5.210189	0.023641
C	1.656208	-3.045966	-0.035704
H	2.022091	-2.513962	-0.910874
H	2.471105	-3.115939	0.688599
O	-0.599275	2.272576	-0.532789
O	-0.077340	-0.716020	-2.314189
O	0.080152	0.715396	2.315511
O	-2.083186	-0.677126	0.283837
C	3.516990	-1.654271	2.799526
C	-0.203228	-3.172346	1.283115
H	-1.124668	-4.303028	-0.284345
C	-1.657276	3.046202	0.028321
C	0.206438	3.173227	-1.284073
C	0.373735	0.039217	-3.434774
C	-0.501281	-1.973606	-2.822829
C	0.500742	1.975032	2.821837
C	-0.373303	-0.037829	3.436541
C	-3.417592	-0.591340	0.240538
H	2.917037	-1.786253	3.692659
H	0.298821	-3.385520	2.234060
H	-1.160254	-2.687487	1.462918
C	-1.050953	4.417084	0.331531
H	-2.027587	2.512938	0.900853
H	-2.468910	3.118355	-0.699449
C	0.291918	4.409007	-0.410721
H	-0.292456	3.386824	-2.236572
H	1.164055	2.688480	-1.460898
H	-0.482607	0.560232	-3.874882
H	1.089016	0.770264	-3.069199
C	0.970349	-0.975054	-4.412321
H	-1.479946	-1.861324	-3.301758
H	-0.601600	-2.657829	-1.984185
C	0.572098	-2.334977	-3.827748
C	-0.575929	2.337070	3.822847
H	0.602068	2.657180	1.981612
H	1.478343	1.865709	3.303608
C	-0.970707	0.978196	4.411919
H	-1.088701	-0.768684	3.070810
H	0.481863	-0.559056	3.878646
C	-3.917540	0.498516	-0.724667
C	-3.927442	-0.337491	1.685072
C	-4.002386	-1.943538	-0.259033
H	-0.910308	4.566146	1.401418
H	-1.706585	5.210646	-0.024182
H	0.458681	5.312547	-0.994943
H	1.123962	4.304607	0.285478
H	0.560130	-0.831895	-5.411139
H	2.051402	-0.872313	-4.477054
H	0.212778	-3.033485	-4.581770
H	1.419316	-2.792663	-3.316950
H	-1.423548	2.789569	3.308097
H	-0.220816	3.039882	4.574862
H	-2.051437	0.873418	4.478828
H	-0.558468	0.839017	5.410478
C	-5.036840	1.290394	-0.525378
C	-3.168022	0.688103	-1.874990
F	-3.591236	0.900890	2.083296
F	-5.256464	-0.455276	1.850248
F	-3.369299	-1.183238	2.559455
F	-5.333825	-1.926941	-0.422412
F	-3.721667	-2.958386	0.571712
F	-3.469284	-2.261496	-1.446035
C	-5.381054	2.265411	-1.445762
H	-5.656014	1.167334	0.351732
C	-3.519029	1.647723	-2.802799
H	-2.289439	0.077568	-2.026425
C	-4.626276	2.448382	-2.587725
H	-6.252091	2.884242	-1.264883
H	-2.919239	1.778176	-3.696262
H	-4.900426	3.210272	-3.308006

95
structure_2l.xyz

Ca	0.037318	-0.033650	-0.062455
O	2.179060	0.501146	-0.163854
C	3.488723	0.604700	0.085360
C	3.664605	1.516593	1.330290
F	3.155039	0.907016	2.410107
F	4.931712	1.844083	1.632147
F	3.003828	2.674598	1.189958
C	4.178277	1.262455	-1.142024
F	4.039218	0.471113	-2.215072
F	5.493688	1.482327	-0.991806
F	3.627394	2.442734	-1.459053
C	4.185807	-0.746406	0.338554
C	3.446157	-1.893207	0.115651
H	2.420983	-1.791073	-0.206509
C	5.306329	-3.267475	0.738236
H	5.742823	-4.246736	0.896642
C	6.056227	-2.126991	0.956481
H	7.085466	-2.207980	1.285931
C	5.504118	-0.875625	0.754117
H	6.117245	-0.002421	0.923298
O	0.475092	-1.658722	-1.811982
C	-0.602441	-2.412105	-3.717784
H	-0.874135	-3.309356	-4.271487
C	0.750100	-1.829278	-4.147044
H	0.622945	-0.923135	-4.738071
H	1.322808	-2.532816	-4.749656
C	1.469287	-1.543345	-2.828150
H	1.905330	-0.551752	-2.748579
H	2.256898	-2.277670	-2.636966
O	-0.478970	1.210788	1.952970
O	-0.303837	1.850940	-1.560773
O	0.449246	-1.770614	1.554980
O	-2.087888	-0.641426	-0.260210
C	3.997560	-3.145093	0.312772
C	-0.411955	-2.687105	-2.240100
H	-1.401398	-1.683194	-3.849998
C	-1.456649	0.698643	2.855749
C	-0.028481	2.435223	2.517120
C	0.388074	3.089126	-1.335711
C	-0.512952	1.673887	-2.960428
C	1.215906	-1.680232	2.758880
C	-0.185895	-3.042381	1.523988
C	-3.426282	-0.603508	-0.277576
H	3.397694	-4.030178	0.132664
H	0.063995	-3.659977	-2.070292
H	-1.319774	-2.608018	-1.648435
C	-2.209598	1.915352	3.379829
H	-0.944067	0.170654	3.667022
H	-2.074457	-0.003964	2.304968
C	-1.287664	3.096536	3.049135
H	0.485143	2.998240	1.742294
H	0.684125	2.218381	3.320030
H	-0.272382	3.763659	-0.786195
H	1.265877	2.860985	-0.733048
C	0.758099	3.626816	-2.711510
H	-1.471244	2.118734	-3.246016
H	-0.555453	0.604970	-3.160580
C	0.647529	2.396529	-3.600046
C	0.637655	-2.727387	3.713363
H	1.125328	-0.658963	3.123054
H	2.265366	-1.868317	2.528559
C	-0.570465	-3.282838	2.964296
H	-1.027039	-2.975611	0.839049
H	0.523674	-3.797418	1.165301
C	-4.009052	0.714543	0.263578
C	-3.957357	-1.825521	0.522093
C	-3.936928	-0.732725	-1.744704
H	-2.396555	1.824782	4.448971
H	-3.169083	2.019741	2.877459
H	-1.737801	3.721377	2.279189
H	-1.075983	3.725384	3.912676
H	0.044276	4.386843	-3.032782
H	1.752473	4.068305	-2.717154
H	0.468736	2.637203	-4.646889
H	1.551962	1.790411	-3.527261
H	0.366578	-2.296486	4.675886
H	1.369471	-3.514006	3.897354
H	-1.471401	-2.713648	3.195289
H	-0.761488	-4.332650	3.181195
C	-5.192667	0.827206	0.976151
C	-3.301693	1.868440	-0.034817
F	-3.753506	-1.649756	1.838128
F	-5.267339	-2.078550	0.358260
F	-3.315030	-2.950084	0.185907
F	-5.255896	-0.531792	-1.875118
F	-3.673756	-1.937597	-2.274778
F	-3.326324	0.170369	-2.522172
C	-5.650327	2.066867	1.387356
H	-5.779574	-0.046510	1.221648
C	-3.770155	3.107948	0.353817
H	-2.376745	1.786992	-0.588037
C	-4.946458	3.213066	1.072474
H	-6.572254	2.132978	1.953011
H	-3.209313	3.998768	0.094565
H	-5.312872	4.183197	1.387011

95
structure_2m.xyz

Ca	0.001039	-0.003678	0.089925
O	-2.139349	0.504877	0.077951
C	-3.425716	0.673790	-0.242083
C	-3.514231	1.314648	-1.654750
F	-2.875161	0.535572	-2.538481
F	-4.763787	1.480147	-2.115060
F	-2.928592	2.519841	-1.704887
C	-4.048042	1.636045	0.805195
F	-4.114828	1.021982	1.997386
F	-5.288189	2.067016	0.517480
F	-3.301314	2.732859	0.977915
C	-4.234579	-0.638324	-0.279976
C	-3.522011	-1.820518	-0.209642
H	-2.447074	-1.762674	-0.126395
C	-5.540191	-3.099502	-0.364108
H	-6.049923	-4.055388	-0.395701
C	-6.261841	-1.922532	-0.437285
H	-7.341414	-1.951938	-0.527176
C	-5.616211	-0.700758	-0.400529
H	-6.205343	0.202208	-0.470651
O	-0.467735	-1.534362	1.926483
C	0.015849	-2.306435	4.114849
H	-0.193315	-3.363616	4.281680
C	-1.283784	-1.513230	4.093055
H	-1.107588	-0.464682	4.337208
H	-2.034703	-1.902981	4.778397
C	-1.694759	-1.639507	2.646470
H	-2.359149	-0.860102	2.281518
H	-2.152750	-2.614906	2.448612
O	0.383563	1.936101	-1.313721
O	0.467200	1.532174	1.922570
O	-0.389433	-1.947047	-1.303775
O	2.141320	-0.506464	0.076828
C	-4.164350	-3.043457	-0.253516
C	0.589544	-2.092242	2.716747
H	0.704265	-1.978725	4.892416
C	0.852750	2.024791	-2.656101
C	0.065235	3.246226	-0.826489
C	-0.589542	2.099822	2.706355
C	1.694005	1.646559	2.641234
C	-0.848080	-2.029392	-2.650491
C	-0.072547	-3.258600	-0.819401
C	3.428356	-0.674240	-0.240967
H	-3.584348	-3.958129	-0.199645
H	0.925001	-3.023427	2.258484
H	1.421750	-1.389957	2.693532
C	0.156005	3.242661	-3.213515
H	0.602148	1.094921	-3.164295
H	1.940853	2.141952	-2.656498
C	0.188876	4.185600	-2.019268
H	0.757699	3.501717	-0.021728
H	-0.944653	3.203385	-0.421424
H	-0.919404	3.029752	2.241482
H	-1.424792	1.401226	2.684848
C	-0.018468	2.320752	4.104741
H	2.149288	2.621644	2.435578
H	2.360526	0.866222	2.282259
C	1.283374	1.530702	4.088825
C	-0.156893	-3.251539	-3.205463
H	-0.585505	-1.100001	-3.153855
H	-1.937030	-2.137596	-2.659672
C	-0.204351	-4.195551	-2.012711
H	0.939680	-3.220101	-0.419418
H	-0.761747	-3.512344	-0.011367
C	4.234829	0.639177	-0.282123
C	3.518614	-1.319098	-1.651491
C	4.051172	-1.631727	0.810297
H	-0.871291	2.993210	-3.480074
H	0.658556	3.646635	-4.090990
H	1.141049	4.717434	-1.987936
H	-0.610861	4.923815	-2.038472
H	0.187496	3.379079	4.268019
H	-0.707232	1.994218	4.882499
H	2.033290	1.927814	4.771029
H	1.110101	0.483647	4.341363
H	0.873958	-3.009734	-3.465283
H	-0.657191	-3.650412	-4.086539
H	0.586405	-4.943430	-2.030244
H	-1.163097	-4.715702	-1.984615
C	5.616296	0.703814	-0.403229
C	3.520025	1.820192	-0.214424
F	2.884147	-0.540220	-2.538857
F	4.768776	-1.489814	-2.108299
F	2.929095	-2.522414	-1.699675
F	5.292799	-2.060618	0.526080
F	3.306732	-2.729684	0.985419
F	4.114693	-1.013414	2.000493
C	6.259609	1.926741	-0.442907
H	6.207075	-0.198241	-0.471415
C	4.160013	3.044231	-0.261481
H	2.445197	1.760292	-0.130894
C	5.535761	3.102517	-0.372455
H	7.339105	1.958017	-0.533110
H	3.578309	3.957984	-0.210007
H	6.043716	4.059261	-0.406515

95
structure_2n.xyz

Ca	0.037341	0.075263	0.155270
O	-2.100758	0.586978	0.153446
C	-3.374357	0.800440	-0.189842
C	-3.405258	1.569780	-1.539144
F	-2.777723	0.847275	-2.478014
F	-4.635428	1.830993	-2.008228
F	-2.769347	2.746933	-1.466911
C	-4.025221	1.666595	0.921812
F	-4.100704	0.954526	2.057308
F	-5.267699	2.100491	0.650438
F	-3.297851	2.755729	1.195775
C	-4.194210	-0.493673	-0.366590
C	-3.507271	-1.691091	-0.293613
H	-2.442355	-1.654761	-0.117116
C	-5.521713	-2.923643	-0.689386
H	-6.039576	-3.867247	-0.815337
C	-6.217994	-1.731567	-0.761959
H	-7.286085	-1.736801	-0.945075
C	-5.561478	-0.525076	-0.606097
H	-6.130229	0.390663	-0.676339
O	-0.440810	-1.473160	1.975780
C	-0.449632	-3.675825	2.797407
H	-0.342819	-4.452543	2.041558
C	-1.872204	-3.098115	2.805206
H	-2.438228	-3.374742	3.693161
H	-2.434931	-3.434478	1.934994
C	-1.646974	-1.600898	2.720441
H	-1.500888	-1.166155	3.716179
H	-2.423016	-1.047412	2.198229
O	0.495221	2.108222	-1.083213
O	0.533000	1.488473	2.091250
O	-0.393714	-1.765169	-1.377440
O	2.147065	-0.568465	0.121697
C	-4.160407	-2.898327	-0.456749
C	0.437888	-2.472313	2.484308
H	-0.180002	-4.114823	3.756856
C	1.007160	2.268312	-2.404261
C	0.241574	3.395033	-0.503196
C	-0.554965	2.047137	2.842874
C	1.598122	1.118895	2.974942
C	-0.856466	-1.721570	-2.725661
C	-0.144468	-3.121861	-0.993197
C	3.428908	-0.689669	-0.237956
H	-3.601464	-3.826090	-0.402413
H	1.205819	-2.649863	1.737247
H	0.918622	-2.091988	3.391345
C	0.438410	3.587249	-2.869053
H	0.688383	1.412595	-2.997252
H	2.100410	2.286634	-2.371023
C	0.523652	4.415248	-1.596047
H	0.887301	3.521714	0.367064
H	-0.797440	3.404940	-0.175653
H	-0.795056	3.028831	2.435000
H	-1.424899	1.403203	2.709501
C	-0.085848	2.095107	4.285126
H	2.351024	1.912886	2.976223
H	2.055214	0.210782	2.587007
C	0.947443	0.981411	4.330287
C	-0.265786	-2.954725	-3.366009
H	-0.519883	-0.785212	-3.168140
H	-1.950006	-1.742550	-2.735810
C	-0.381564	-3.966777	-2.236137
H	0.883196	-3.176548	-0.635976
H	-0.819402	-3.386054	-0.176621
C	4.225967	0.629194	-0.172437
C	3.483219	-1.212377	-1.700151
C	4.092701	-1.721661	0.712524
H	-0.599905	3.457770	-3.176708
H	1.000176	4.015471	-3.697746
H	1.528700	4.825072	-1.483796
H	-0.185184	5.241145	-1.573731
H	0.387081	3.054245	4.503349
H	-0.908197	1.955222	4.984926
H	1.660696	1.084357	5.146521
H	0.459810	0.009320	4.417580
H	0.779622	-2.779015	-3.620920
H	-0.801619	-3.254445	-4.265259
H	0.335722	-4.781660	-2.316090
H	-1.384687	-4.395900	-2.219788
C	5.583528	0.732386	-0.446709
C	3.533598	1.773694	0.175518
F	2.870175	-0.336233	-2.509004
F	4.719387	-1.394112	-2.191065
F	2.845875	-2.382393	-1.843440
F	5.356244	-2.051708	0.399183
F	3.405236	-2.872010	0.759637
F	4.114728	-1.232917	1.960585
C	6.223168	1.955641	-0.372960
H	6.158551	-0.139438	-0.722271
C	4.170047	2.998464	0.246909
H	2.478670	1.691227	0.385407
C	5.520461	3.094814	-0.027292
H	7.283277	2.015875	-0.589698
H	3.605332	3.882645	0.520926
H	6.025192	4.052262	0.028309

95
structure_2o.xyz

Ca	0.026421	-0.055985	0.025120
O	-2.136179	0.374327	0.231372
C	-3.429267	0.708573	0.280654
C	-3.631732	2.064258	-0.453280
F	-3.344561	1.925798	-1.756861
F	-4.878365	2.558438	-0.387689
F	-2.820310	3.016979	0.020022
C	-3.833835	0.876895	1.771493
F	-3.720936	-0.302073	2.404944
F	-5.090191	1.301785	1.978773
F	-3.032767	1.740011	2.407123
C	-4.359159	-0.334739	-0.368993
C	-3.759366	-1.438616	-0.945380
H	-2.680582	-1.503919	-0.911716
C	-5.894052	-2.322904	-1.574511
H	-6.492427	-3.096795	-2.040858
C	-6.502424	-1.218923	-1.006786
H	-7.581617	-1.122566	-1.027462
C	-5.743335	-0.231174	-0.408047
H	-6.245391	0.620173	0.027884
O	-0.540764	-2.385724	0.508441
C	0.456239	-3.705970	2.123565
H	0.847986	-4.699617	2.334464
C	-1.021510	-3.546985	2.502537
H	-1.140552	-2.955215	3.408637
H	-1.501903	-4.508861	2.676398
C	-1.640571	-2.848080	1.291224
H	-2.263526	-1.990071	1.525041
H	-2.226387	-3.548621	0.688561
O	0.298446	2.283377	-0.538234
O	0.393446	0.529425	2.347067
O	-0.287515	-0.735187	-2.300101
O	2.230189	-0.441512	-0.041089
C	-4.517282	-2.428561	-1.542008
C	0.465857	-3.384170	0.643984
H	1.074533	-2.980214	2.651519
C	0.297897	2.774680	-1.880903
C	0.262012	3.368296	0.395296
C	-0.448405	0.230283	3.456036
C	1.635407	0.945214	2.905035
C	-1.309707	-0.275035	-3.189341
C	0.175997	-1.987718	-2.793289
C	3.537189	-0.558070	-0.311304
H	-4.028776	-3.289168	-1.985261
H	0.184744	-4.256377	0.041773
H	1.401173	-2.970254	0.280103
C	0.674045	4.234736	-1.769161
H	-0.704599	2.650554	-2.298553
H	1.006287	2.180147	-2.458934
C	0.048448	4.617222	-0.436579
H	1.218526	3.395493	0.925254
H	-0.534808	3.178315	1.112673
H	-0.906671	1.156674	3.810483
H	-1.239537	-0.428945	3.108155
C	0.465894	-0.378152	4.518989
H	1.529383	1.969622	3.280967
H	2.382079	0.915683	2.116917
C	1.879775	-0.036296	4.033195
C	-1.840161	-1.510670	-3.920068
H	-0.875326	0.447988	-3.884295
H	-2.062761	0.219851	-2.583253
C	-1.081541	-2.667751	-3.277118
H	0.884682	-1.820438	-3.609992
H	0.689208	-2.501736	-1.986405
C	4.295043	0.782453	-0.387509
C	3.693219	-1.274694	-1.678628
C	4.207680	-1.415297	0.803274
H	0.296850	4.821886	-2.604772
H	1.758912	4.341765	-1.735780
H	0.506534	5.494226	0.018075
H	-1.017066	4.810429	-0.560522
H	0.253411	0.050910	5.497243
H	0.327749	-1.455335	4.597570
H	2.508503	0.386840	4.814965
H	2.379903	-0.920513	3.642038
H	-1.613756	-1.450520	-4.984521
H	-2.918497	-1.608043	-3.810876
H	-0.880275	-3.483710	-3.969626
H	-1.627004	-3.066554	-2.420550
C	5.672293	0.883511	-0.536071
C	3.552647	1.941809	-0.281151
F	3.044410	-0.579562	-2.620471
F	4.954831	-1.420966	-2.109765
F	3.158536	-2.507501	-1.652543
F	5.437929	-1.863752	0.499726
F	3.482796	-2.495475	1.115670
F	4.324534	-0.694674	1.928160
C	6.284574	2.121784	-0.566424
H	6.284503	-0.002007	-0.626904
C	4.164479	3.181826	-0.305948
H	2.481346	1.850501	-0.182325
C	5.534716	3.277266	-0.447483
H	7.360623	2.181384	-0.680323
H	3.567154	4.081149	-0.208036
H	6.018440	4.246874	-0.465428

95
structure_2p.xyz

Ca	0.024533	0.097359	-0.160046
O	2.215436	0.544769	-0.034827
C	3.470534	0.416322	0.416036
C	3.457554	0.586373	1.958674
F	2.885379	-0.482594	2.537033
F	4.666106	0.736621	2.529605
F	2.741940	1.654632	2.323196
C	4.356227	1.547745	-0.187749
F	4.298291	1.511146	-1.526094
F	5.651760	1.456280	0.144842
F	3.943159	2.769671	0.180575
C	4.138837	-0.910344	0.011061
C	3.783187	-1.427424	-1.222792
H	3.026168	-0.911629	-1.794097
C	5.319659	-3.242941	-0.963745
H	5.781179	-4.147857	-1.340976
C	5.667726	-2.748304	0.278220
H	6.403777	-3.265688	0.882215
C	5.092348	-1.584602	0.758104
H	5.405912	-1.214008	1.723487
O	0.226850	0.826568	-2.489652
C	-0.078693	2.802341	-3.766677
H	-0.239931	2.811523	-4.844296
C	1.396136	2.580702	-3.437578
H	1.685159	3.119864	-2.535067
H	2.064272	2.883491	-4.242184
C	1.451239	1.096567	-3.161834
H	2.259410	0.811622	-2.497830
H	1.493578	0.514175	-4.089680
O	-0.376631	-0.042520	2.284043
O	-0.461299	2.452471	0.225388
O	0.560133	-2.269212	-0.279092
O	-2.140028	-0.278578	-0.412230
C	4.372881	-2.573863	-1.716505
C	-0.786484	1.600139	-3.135139
H	-0.449650	3.747337	-3.373965
C	-1.141569	-1.061036	2.933359
C	-0.061933	0.938609	3.264637
C	0.507623	3.484116	0.380464
C	-1.730581	3.089200	0.304161
C	0.936162	-3.000961	0.888179
C	0.758214	-3.070321	-1.449453
C	-3.384224	-0.733010	-0.597408
H	4.086030	-2.950518	-2.691738
H	-1.276152	0.983292	-3.889699
H	-1.524118	1.865519	-2.381780
C	-1.797342	-0.406037	4.151305
H	-0.472361	-1.870662	3.233884
H	-1.855607	-1.438561	2.205822
C	-1.319982	1.042877	4.093975
H	0.216419	1.852009	2.747123
H	0.786899	0.598717	3.865052
H	0.612903	3.715955	1.446059
H	1.456818	3.102175	0.014738
C	-0.054539	4.678106	-0.380195
H	-1.971381	3.274059	1.357471
H	-2.470495	2.411212	-0.114013
C	-1.562156	4.394668	-0.458156
C	1.874488	-4.075061	0.395101
H	0.037234	-3.430761	1.340912
H	1.398268	-2.305705	1.587583
C	1.270552	-4.413701	-0.958887
H	-0.184573	-3.143774	-1.987838
H	1.488767	-2.566560	-2.083223
C	-4.377219	-0.284242	0.492430
C	-3.350687	-2.285408	-0.626025
C	-3.896812	-0.202416	-1.963956
H	-1.460327	-0.887612	5.068836
H	-2.881980	-0.482889	4.111506
H	-2.045641	1.672577	3.578999
H	-1.136279	1.470285	5.078589
H	0.167479	5.610637	0.136170
H	0.381293	4.744070	-1.375602
H	-2.162665	5.190018	-0.019725
H	-1.882837	4.273497	-1.492025
H	1.915931	-4.928794	1.069971
H	2.879116	-3.668789	0.281509
H	0.442795	-5.114913	-0.841380
H	1.991320	-4.849079	-1.649467
C	-5.725094	-0.619093	0.505191
C	-3.882253	0.514626	1.505437
F	-2.954506	-2.752622	0.567695
F	-4.530340	-2.869376	-0.894825
F	-2.484146	-2.751278	-1.532098
F	-5.119476	-0.629538	-2.319138
F	-3.075857	-0.533372	-2.966726
F	-3.954366	1.138573	-1.927060
C	-6.553203	-0.153001	1.508493
H	-6.145307	-1.245178	-0.267955
C	-4.711290	0.987691	2.505569
H	-2.827512	0.752228	1.487531
C	-6.051893	0.656436	2.510665
H	-7.602410	-0.424131	1.502363
H	-4.309173	1.620248	3.288779
H	-6.704650	1.025650	3.292989

95
structure_Sr_replacing_Ca_2a.xyz

38	0.003769	0.161646	-0.001449
8	-2.255399	0.489216	-0.600266
6	-3.537444	0.112234	-0.634448
6	-3.686044	-0.958011	-1.751235
9	-3.113849	-2.109778	-1.360703
9	-4.949968	-1.249648	-2.105553
9	-3.061060	-0.585100	-2.874497
6	-4.419868	1.340073	-0.986438
9	-4.202175	2.321162	-0.101151
9	-5.737987	1.086930	-0.971018
9	-4.132234	1.838317	-2.198150
6	-4.040414	-0.439594	0.712231
6	-3.382258	0.002098	1.847448
1	-2.567150	0.701956	1.732255
6	-4.772447	-1.365256	3.236880
1	-5.054514	-1.731279	4.217241
6	-5.442069	-1.804262	2.110616
1	-6.253147	-2.516903	2.203730
6	-5.088171	-1.335806	0.857825
1	-5.638199	-1.687641	-0.003678
8	-0.412179	2.052619	1.739491
6	0.870468	3.936594	2.122835
1	1.409440	4.536668	2.854167
6	-0.556898	4.415186	1.889551
1	-0.627863	5.201415	1.139480
1	-0.978153	4.805687	2.816488
6	-1.295951	3.147402	1.464775
1	-1.536036	3.115613	0.402676
1	-2.225586	3.008727	2.020005
8	0.581170	-1.934625	-1.411415
8	0.375076	1.755224	-2.013667
8	-0.553644	-1.736337	1.669411
8	2.274329	0.608502	0.490697
6	-3.745921	-0.449115	3.100809
6	0.646360	2.515878	2.579406
1	1.444542	3.929599	1.194622
6	1.423071	-3.021768	-1.021116
6	0.274641	-2.037144	-2.808451
6	-0.652753	2.114311	-2.933801
6	1.261895	2.868011	-1.973196
6	-1.394598	-2.860886	1.398994
6	-0.249674	-1.684452	3.069599
6	3.552043	0.225923	0.589104
1	-3.219921	-0.085921	3.976501
1	0.323914	2.478359	3.626171
1	1.499349	1.859309	2.424465
6	1.114493	-4.112684	-2.018182
1	1.189277	-3.271073	0.012966
1	2.469401	-2.711576	-1.081171
6	0.952224	-3.306584	-3.297564
1	0.637855	-1.141555	-3.316116
1	-0.810245	-2.082533	-2.909694
1	-0.274013	1.978094	-3.952894
1	-1.499649	1.456659	-2.756825
6	-0.942055	3.578920	-2.640276
1	1.910916	2.836021	-2.855342
1	1.878974	2.769605	-1.082336
6	0.357283	4.092859	-2.000626
6	-1.091797	-3.832924	2.513682
1	-1.155725	-3.223777	0.400361
1	-2.440787	-2.545627	1.418581
6	-0.935199	-2.887994	3.694941
1	0.834747	-1.725044	3.178246
1	-0.608410	-0.736014	3.474060
6	4.060184	-0.505132	-0.666727
6	3.676309	-0.687970	1.839701
6	4.445577	1.480158	0.784964
1	0.181315	-4.615413	-1.755673
1	1.906757	-4.857175	-2.078102
1	1.932698	-3.076196	-3.715714
1	0.363557	-3.815650	-4.058826
1	-1.207338	4.122152	-3.545935
1	-1.779397	3.661251	-1.948579
1	0.810633	4.906122	-2.565264
1	0.174547	4.456359	-0.990665
1	-0.157747	-4.362241	2.314122
1	-1.885037	-4.565637	2.652902
1	-0.353542	-3.309146	4.513167
1	-1.917775	-2.608907	4.077052
6	5.109793	-1.410771	-0.682877
6	3.406692	-0.223896	-1.854501
9	3.092824	-1.874572	1.598275
9	4.932002	-0.945023	2.244741
9	3.044127	-0.161789	2.895763
9	5.759793	1.212460	0.829621
9	4.143181	2.145061	1.909509
9	4.257475	2.331473	-0.232542
6	5.471067	-2.044881	-1.858240
1	5.655974	-1.639848	0.221440
6	3.777611	-0.841578	-3.032485
1	2.589983	0.483502	-1.839592
6	4.806438	-1.765200	-3.037096
1	6.283935	-2.761495	-1.849386
1	3.255407	-0.602915	-3.952123
1	5.094316	-2.260734	-3.956986

95
structure_Sr_replacing_Ca_2b.xyz

Sr	0.048722	-0.081276	0.022075
O	-2.205952	-0.048426	0.701432
C	-3.512595	0.228832	0.653677
C	-3.704950	1.697209	1.122238
F	-3.260208	2.544085	0.178281
F	-4.972757	2.050375	1.397336
F	-2.997683	1.954704	2.228742
C	-4.273309	-0.714030	1.627763
F	-4.000192	-1.990001	1.326352
F	-5.607823	-0.581073	1.585690
F	-3.904281	-0.528676	2.904397
C	-4.128475	0.015562	-0.740899
C	-3.496033	-0.901523	-1.562794
H	-2.606112	-1.392653	-1.196226
C	-5.113610	-0.535413	-3.289289
H	-5.497099	-0.745761	-4.280798
C	-5.749534	0.384744	-2.477641
H	-6.634639	0.900515	-2.831016
C	-5.269609	0.649727	-1.207296
H	-5.799384	1.360476	-0.588661
O	-0.430617	-2.533374	-0.733253
C	0.925776	-4.364917	-0.349260
H	1.457017	-5.211593	-0.780848
C	-0.459655	-4.726461	0.169448
H	-0.449335	-5.115642	1.186334
H	-0.913296	-5.485153	-0.469242
C	-1.233650	-3.414269	0.063828
H	-1.398828	-2.928952	1.025151
H	-2.206513	-3.546814	-0.412907
O	0.712914	2.369460	0.535461
O	0.476692	-0.649451	2.514542
O	-0.448489	0.949148	-2.304829
O	2.297009	-0.761822	-0.242368
C	-3.986321	-1.184984	-2.822530
C	0.601238	-3.295904	-1.362916
H	1.546668	-3.933662	0.437982
C	1.569753	3.226784	-0.223047
C	0.406814	2.984474	1.794322
C	-0.523890	-0.536802	3.522711
C	1.357941	-1.674652	2.961344
C	-1.089920	2.210341	-2.487351
C	-0.599217	0.163217	-3.494158
C	3.563587	-0.473409	-0.558950
H	-3.480538	-1.914605	-3.444976
H	0.217435	-3.728567	-2.293778
H	1.428120	-2.622045	-1.572416
C	1.270749	4.611849	0.297184
H	1.342988	3.079839	-1.276966
H	2.611822	2.949869	-0.044791
C	1.098063	4.337998	1.783001
H	0.760083	2.336099	2.598305
H	-0.677716	3.074653	1.867053
H	-0.111794	0.030479	4.364900
H	-1.369436	-0.005555	3.093220
C	-0.833945	-1.972049	3.919747
H	2.032608	-1.256414	3.716466
H	1.948570	-2.000003	2.107125
C	0.450311	-2.738597	3.565429
C	-2.211872	1.928425	-3.455832
H	-0.373697	2.924407	-2.909001
H	-1.417263	2.564707	-1.511920
C	-1.556095	0.930145	-4.399544
H	0.382366	0.015325	-3.946949
H	-0.999010	-0.808319	-3.199021
C	4.153977	0.669002	0.287669
C	3.604145	-0.100836	-2.067964
C	4.441651	-1.730172	-0.317820
H	0.343058	4.988726	-0.138269
H	2.070461	5.318590	0.081496
H	2.075480	4.269118	2.261942
H	0.514437	5.098233	2.299366
H	-1.091585	-2.048155	4.974934
H	-1.682570	-2.341189	3.345419
H	0.913484	-3.204655	4.433743
H	0.246146	-3.525396	2.840908
H	-2.560761	2.826896	-3.962656
H	-3.051509	1.473954	-2.930280
H	-1.002205	1.452564	-5.180724
H	-2.277077	0.270209	-4.878581
C	5.235266	1.450696	-0.089973
C	3.547430	0.917623	1.506322
F	3.054222	1.109840	-2.255646
F	4.830668	-0.063602	-2.617950
F	2.890441	-0.960677	-2.805564
F	5.752271	-1.533068	-0.529045
F	4.072028	-2.759692	-1.094052
F	4.309895	-2.133526	0.953090
C	5.674770	2.479270	0.723748
H	5.744902	1.272131	-1.026562
C	3.996019	1.932127	2.329300
H	2.702564	0.309233	1.793382
C	5.057190	2.725723	1.935640
H	6.511761	3.090033	0.406255
H	3.508710	2.106705	3.281938
H	5.406551	3.529989	2.572609

95
structure_Sr_replacing_Ca_2c.xyz

Sr	-0.051094	0.069919	0.285178
O	2.254976	-0.054345	0.803601
C	3.502420	-0.476552	0.576490
C	3.501663	-2.027974	0.661256
F	2.867774	-2.541451	-0.406824
F	4.713851	-2.605768	0.710740
F	2.838180	-2.459752	1.741329
C	4.439406	0.094349	1.674914
F	4.367929	1.432953	1.675372
F	5.733257	-0.224098	1.512871
F	4.087688	-0.315697	2.901172
C	4.060421	-0.018623	-0.784174
C	3.438427	1.063964	-1.380010
H	2.598652	1.512262	-0.869600
C	4.920012	0.939727	-3.255803
H	5.253411	1.308593	-4.218723
C	5.551807	-0.139018	-2.666418
H	6.384196	-0.620456	-3.166071
C	5.134308	-0.608220	-1.434017
H	5.657298	-1.443041	-0.989113
O	0.320053	2.617092	0.548180
C	0.219167	4.530822	-0.787068
H	0.140161	4.244473	-1.835248
C	1.655586	4.342489	-0.272277
H	2.106154	5.272000	0.072125
H	2.298754	3.934780	-1.051596
C	1.500280	3.344010	0.865781
H	1.351283	3.860891	1.820656
H	2.315626	2.632841	0.971997
O	-0.717725	-2.393532	-0.153963
O	-0.434625	-0.056728	2.848223
O	0.511281	-0.100931	-2.236934
O	-2.289010	0.815468	0.109677
C	3.865053	1.547313	-2.601261
C	-0.607609	3.597138	0.087716
H	-0.124698	5.560483	-0.701320
C	-1.587359	-2.859228	-1.189311
C	-0.453953	-3.459512	0.768482
C	0.681988	-0.224805	3.718788
C	-1.206702	1.014496	3.395216
C	1.225387	-1.151670	-2.890544
C	0.262136	0.960893	-3.165499
C	-3.575041	0.644610	-0.215127
H	3.368414	2.401588	-3.047586
H	-1.419237	3.078029	-0.414669
H	-1.015527	4.126482	0.955916
C	-1.338591	-4.346833	-1.247717
H	-1.340858	-2.319256	-2.102173
H	-2.623218	-2.640093	-0.920493
C	-1.186196	-4.677087	0.228849
H	-0.801176	-3.162862	1.760015
H	0.625258	-3.610898	0.803996
H	0.354504	-0.753983	4.621019
H	1.434735	-0.808421	3.194741
C	1.119030	1.192093	4.023364
H	-1.898962	0.603923	4.136469
H	-1.784633	1.450581	2.582063
C	-0.201392	1.970497	4.040137
C	0.817439	-1.031824	-4.338933
H	0.948006	-2.090419	-2.414006
H	2.300047	-0.996919	-2.763716
C	0.781146	0.479060	-4.512052
H	-0.811494	1.148638	-3.176652
H	0.773574	1.861842	-2.819673
C	-4.120275	-0.731004	0.207770
C	-3.704172	0.843713	-1.750296
C	-4.436927	1.717315	0.505594
H	-0.414664	-4.558081	-1.790193
H	-2.155088	-4.885823	-1.725563
H	-2.170068	-4.763682	0.691156
H	-0.636010	-5.598269	0.412975
H	1.669949	1.264811	4.959705
H	1.767844	1.536392	3.218591
H	-0.508985	2.223115	5.054184
H	-0.123690	2.899013	3.477516
H	-0.173990	-1.463437	-4.490503
H	1.518475	-1.522938	-5.011864
H	0.146056	0.804855	-5.333956
H	1.789882	0.855100	-4.686653
C	-5.143806	-1.409450	-0.434963
C	-3.523375	-1.318901	1.311143
F	-3.146919	-0.194044	-2.396991
F	-4.960772	0.963765	-2.212958
F	-3.051651	1.939929	-2.157507
F	-5.756432	1.578788	0.302121
F	-4.112128	2.963597	0.132806
F	-4.238621	1.643252	1.828214
C	-5.532701	-2.665493	-0.003588
H	-5.646497	-0.973764	-1.286990
C	-3.922089	-2.564036	1.754501
H	-2.729415	-0.787839	1.817300
C	-4.922660	-3.249415	1.089853
H	-6.323340	-3.188432	-0.528747
H	-3.443922	-3.003447	2.622522
H	-5.231212	-4.232062	1.427259

95
structure_Sr_replacing_Ca_2d.xyz

Sr	-0.069747	0.138475	0.021509
O	-2.325546	0.518466	-0.523372
C	-3.595042	0.119776	-0.647732
C	-3.686085	-0.796104	-1.900288
F	-3.121333	-1.987439	-1.643365
F	-4.933458	-1.041436	-2.340165
F	-3.019798	-0.275923	-2.937889
C	-4.496060	1.366122	-0.862365
F	-4.312577	2.232649	0.142286
F	-5.809154	1.092172	-0.905761
F	-4.195914	2.016530	-1.997116
C	-4.123839	-0.612542	0.598784
C	-3.511769	-0.307973	1.802364
H	-2.700399	0.405231	1.801326
C	-4.935841	-1.843734	2.962902
H	-5.250827	-2.326416	3.880698
C	-5.554901	-2.151928	1.766222
H	-6.358338	-2.878768	1.741401
C	-5.161062	-1.532226	0.593701
H	-5.673855	-1.781329	-0.324979
O	-0.492284	1.700700	2.046779
C	0.795875	3.466209	2.790944
H	1.329646	3.915277	3.626853
C	-0.625244	3.994392	2.634024
H	-0.679647	4.897654	2.028428
H	-1.049969	4.227777	3.610738
C	-1.377257	2.826096	1.995954
H	-1.639045	2.988079	0.950989
H	-2.295412	2.588119	2.536852
O	0.392692	-1.756667	-1.688302
O	0.297065	2.046812	-1.691666
O	-0.610068	-1.908954	1.495018
O	2.209928	0.440754	0.576729
C	-3.916182	-0.910091	2.977812
C	0.555898	1.987237	2.972548
H	1.381718	3.626661	1.884290
C	1.026329	-2.998817	-1.391651
C	0.470559	-1.512222	-3.098634
C	-0.732688	2.523894	-2.554945
C	1.142590	3.165225	-1.445703
C	-1.429534	-3.026455	1.161029
C	-0.210930	-2.121066	2.842151
C	3.511037	0.133903	0.610909
H	-3.428275	-0.650565	3.910469
H	0.217358	1.758258	3.989468
H	1.406294	1.361738	2.709946
C	2.104124	-3.130831	-2.438978
H	0.293079	-3.809464	-1.471745
H	1.397406	-2.950285	-0.370212
C	1.401949	-2.575783	-3.670404
H	0.860905	-0.502702	-3.236026
H	-0.533663	-1.560166	-3.522270
H	-0.336925	2.578874	-3.575128
H	-1.557034	1.817095	-2.509172
C	-1.079110	3.904122	-2.017553
H	1.805701	3.304401	-2.306580
H	1.749998	2.937349	-0.572124
C	0.196159	4.347344	-1.283318
C	-0.759463	-4.224706	1.826949
H	-1.488639	-3.085869	0.077104
H	-2.434934	-2.857001	1.555857
C	0.139324	-3.597656	2.903576
H	0.621836	-1.456123	3.060865
H	-1.050997	-1.880741	3.502752
C	4.080573	-0.252404	-0.766184
C	3.700578	-1.024470	1.628594
C	4.314367	1.371008	1.100391
H	2.438991	-4.159093	-2.567352
H	2.961022	-2.513848	-2.168403
H	2.097145	-2.157761	-4.396194
H	0.825255	-3.358697	-4.164656
H	-1.360064	4.585389	-2.819134
H	-1.922740	3.834295	-1.332195
H	0.626120	5.256730	-1.700279
H	-0.007162	4.533520	-0.230032
H	-0.167461	-4.792565	1.110528
H	-1.503273	-4.898586	2.249360
H	1.191252	-3.739963	2.659241
H	-0.034868	-4.013251	3.894793
C	5.207350	-1.035487	-0.964361
C	3.420489	0.252560	-1.873376
F	3.204693	-2.166451	1.122661
F	4.971641	-1.275754	1.985260
F	3.034185	-0.790697	2.766043
F	5.644099	1.193785	1.102182
F	3.968112	1.739769	2.342564
F	4.063484	2.417025	0.301354
C	5.645506	-1.322452	-2.244992
H	5.758605	-1.433969	-0.124258
C	3.868470	-0.014508	-3.152261
H	2.543979	0.864125	-1.714877
C	4.981243	-0.811662	-3.343942
H	6.520094	-1.947622	-2.381191
H	3.341197	0.399686	-4.004263
H	5.331672	-1.033107	-4.345215

95
structure_Sr_replacing_Ca_2e.xyz

Sr	-0.044529	-0.092509	0.147808
O	-2.255551	0.677507	0.356597
C	-3.540650	0.832751	0.026517
C	-3.603742	1.718520	-1.248305
F	-3.206932	1.015048	-2.321933
F	-4.819143	2.217254	-1.537372
F	-2.780770	2.769210	-1.155167
C	-4.280471	1.562821	1.182715
F	-4.098673	0.892837	2.328085
F	-5.605748	1.671645	1.001904
F	-3.812251	2.804852	1.379282
C	-4.274260	-0.501672	-0.197333
C	-3.761055	-1.606815	0.459288
H	-2.871313	-1.479462	1.058735
C	-5.491464	-2.996757	-0.437793
H	-5.965530	-3.966452	-0.534716
C	-6.007401	-1.900177	-1.102551
H	-6.888405	-2.007113	-1.724438
C	-5.411708	-0.658095	-0.974276
H	-5.848629	0.185607	-1.490359
O	-0.560170	-1.691253	2.120555
C	0.997853	-1.637239	3.826939
H	1.513281	-2.229656	4.581116
C	-0.275383	-0.968563	4.354569
H	-0.114236	0.084789	4.575721
H	-0.629623	-1.445793	5.267585
C	-1.290717	-1.153669	3.224603
H	-1.762418	-0.232022	2.887273
H	-2.074756	-1.863087	3.505786
O	0.467112	1.302849	-1.986454
O	0.394332	1.744734	1.920267
O	-0.702819	-2.024088	-1.451032
O	2.231478	-0.597835	0.519878
C	-4.366771	-2.843484	0.350903
C	0.491103	-2.477809	2.675801
H	1.697300	-0.898702	3.436590
C	0.959838	0.795031	-3.225405
C	0.674302	2.719801	-1.937294
C	-0.455146	2.878456	2.074614
C	1.528794	2.010154	2.735623
C	-1.313632	-1.833630	-2.726542
C	-0.937621	-3.365825	-1.002234
C	3.478680	-0.865725	0.123334
H	-3.953228	-3.693377	0.882091
H	0.080851	-3.434910	3.018003
H	1.233498	-2.645934	1.899151
C	2.074588	1.737794	-3.605635
H	0.154390	0.807113	-3.967677
H	1.283613	-0.230716	-3.063565
C	1.503444	3.077724	-3.164539
H	1.200663	2.943895	-1.007868
H	-0.294897	3.219299	-1.921796
H	-1.193965	2.855033	1.277800
H	-0.977343	2.798058	3.034240
C	0.489026	4.072609	2.056952
H	2.320871	1.326044	2.440477
H	1.264768	1.821605	3.781952
C	1.834585	3.485345	2.512680
C	-2.470010	-2.802421	-2.746023
H	-0.586366	-2.061237	-3.513458
H	-1.612875	-0.790341	-2.804273
C	-1.878685	-4.000655	-2.017872
H	0.018879	-3.885730	-0.931370
H	-1.380732	-3.306666	-0.007537
C	4.167194	0.328795	-0.561954
C	3.427199	-2.085447	-0.838183
C	4.335378	-1.233456	1.365999
H	2.305889	1.703161	-4.669161
H	2.975332	1.493902	-3.041855
H	2.275906	3.809041	-2.933211
H	0.864792	3.493731	-3.944866
H	0.565621	4.484129	1.051042
H	0.134966	4.867715	2.711232
H	2.595222	3.607063	1.742547
H	2.210830	3.952756	3.421291
H	-2.798696	-3.038009	-3.757144
H	-3.311515	-2.386837	-2.191918
H	-1.320313	-4.629813	-2.712519
H	-2.636992	-4.616174	-1.536913
C	5.281356	0.234651	-1.381922
C	3.637664	1.580064	-0.303248
F	2.905548	-1.716297	-2.020212
F	4.614177	-2.659694	-1.099813
F	2.638524	-3.054403	-0.359258
F	5.632360	-1.448890	1.097878
F	3.878399	-2.331137	1.985313
F	4.286213	-0.233254	2.257132
C	5.836849	1.371528	-1.940888
H	5.730374	-0.725630	-1.594536
C	4.204310	2.719553	-0.840167
H	2.763682	1.635890	0.329771
C	5.305386	2.618461	-1.669056
H	6.700129	1.279370	-2.589357
H	3.780267	3.692052	-0.615687
H	5.749428	3.507174	-2.101843

95
structure_Sr_replacing_Ca_2f.xyz

38	-0.009191	-0.108254	0.180053
8	-2.241226	0.616605	0.410964
6	-3.543474	0.730893	0.130866
6	-3.692071	1.741188	-1.039223
9	-3.253117	1.190363	-2.183312
9	-4.944195	2.173379	-1.269929
9	-2.952177	2.837846	-0.834982
6	-4.291253	1.291077	1.373908
9	-4.063080	0.502023	2.432102
9	-5.622684	1.361949	1.222124
9	-3.871510	2.520217	1.708116
6	-4.208780	-0.613461	-0.215145
6	-3.634286	-1.746884	0.335092
1	-2.751964	-1.632469	0.948165
6	-5.291635	-3.137725	-0.689699
1	-5.712861	-4.118315	-0.877925
6	-5.869949	-2.012936	-1.246373
1	-6.747579	-2.107848	-1.874917
6	-5.341169	-0.757855	-1.001540
1	-5.826719	0.104719	-1.435862
8	-0.479645	-1.818646	2.073209
6	1.085566	-1.757469	3.774241
1	1.614965	-2.352880	4.516320
6	-0.200508	-1.125611	4.316820
1	-0.064342	-0.069378	4.541317
1	-0.535517	-1.616200	5.229998
6	-1.219342	-1.329241	3.193630
1	-1.728642	-0.420062	2.879017
1	-1.973186	-2.073097	3.468407
8	0.646808	1.432357	-1.807809
8	0.401777	1.646380	2.040768
8	-0.607320	-1.835377	-1.658055
8	2.271684	-0.600862	0.561817
6	-4.172829	-2.998508	0.110135
6	0.595308	-2.587786	2.608318
1	1.768223	-0.997481	3.395432
6	1.378496	1.103595	-2.990070
6	0.461364	2.851911	-1.741758
6	-0.485148	2.734404	2.288436
6	1.567043	1.926368	2.806188
6	-1.233411	-1.509741	-2.897533
6	-0.796443	-3.229759	-1.382210
6	3.503940	-0.842656	0.105406
1	-3.711489	-3.870656	0.559560
1	0.211170	-3.561759	2.932614
1	1.338290	-2.719331	1.825816
6	1.028068	2.205113	-3.960505
1	1.074247	0.108249	-3.306287
1	2.448548	1.091813	-2.766608
6	1.015876	3.417114	-3.041334
1	0.984932	3.237291	-0.863992
1	-0.605609	3.041616	-1.626233
1	-1.249001	2.726000	1.515100
1	-0.970946	2.576266	3.257308
6	0.405585	3.971791	2.313339
1	2.364371	1.276821	2.452599
1	1.362689	1.702420	3.859255
6	1.807677	3.414452	2.606346
6	-2.379451	-2.484248	-3.011617
1	-0.514938	-1.645639	-3.713715
1	-1.539299	-0.465990	-2.855955
6	-1.760567	-3.752125	-2.440743
1	0.172441	-3.730215	-1.412420
1	-1.203421	-3.313009	-0.373480
6	4.138902	0.370277	-0.599944
6	3.423289	-2.046452	-0.874655
6	4.419818	-1.214297	1.302119
1	0.038462	2.030887	-4.387204
1	1.748834	2.291384	-4.772031
1	2.033813	3.779536	-2.894115
1	0.410407	4.239219	-3.418921
1	0.382270	4.485260	1.352940
1	0.072327	4.679477	3.070907
1	2.476438	3.580012	1.762238
1	2.267776	3.866668	3.483468
1	-2.724760	-2.603668	-4.037480
1	-3.214582	-2.148417	-2.397418
1	-1.216049	-4.292373	-3.216386
1	-2.502560	-4.425470	-2.015365
6	5.222300	0.300215	-1.462800
6	3.579092	1.607378	-0.336769
9	2.829127	-1.665263	-2.016878
9	4.603712	-2.594462	-1.211959
9	2.679993	-3.040158	-0.371415
9	5.704926	-1.416056	0.971475
9	4.001635	-2.321503	1.931512
9	4.403607	-0.222494	2.203758
6	5.711328	1.443783	-2.067880
1	5.696034	-0.647714	-1.676620
6	4.076995	2.753815	-0.924507
1	2.730322	1.647865	0.330259
6	5.141915	2.674995	-1.802084
1	6.550295	1.369277	-2.749719
1	3.628433	3.715146	-0.699945
1	5.531175	3.569805	-2.273514

95
structure_Sr_replacing_Ca_2g.xyz

Sr	-0.005307	-0.109706	0.168104
O	2.283078	-0.616551	0.479759
C	3.495432	-0.866200	-0.023699
C	3.334647	-1.941577	-1.132144
F	2.688242	-1.409590	-2.182855
F	4.479455	-2.464675	-1.602396
F	2.595410	-2.975989	-0.710863
C	4.401445	-1.411862	1.111472
F	4.477089	-0.505049	2.095664
F	5.661477	-1.682386	0.734356
F	3.910870	-2.535394	1.652645
C	4.185714	0.381802	-0.606694
C	3.630036	1.607409	-0.289835
H	2.741444	1.612973	0.324474
C	5.306350	2.746833	-1.562781
H	5.743312	3.665542	-1.936263
C	5.870791	1.526381	-1.883567
H	6.753331	1.484520	-2.511047
C	5.322186	0.351873	-1.401908
H	5.793547	-0.587412	-1.654110
O	0.443683	1.444281	2.188178
C	0.396305	3.740340	2.663963
H	0.275144	4.354303	1.772616
C	1.834024	3.210244	2.780224
H	2.367677	3.616365	3.637975
H	2.411937	3.453450	1.888672
C	1.651286	1.706228	2.892341
H	1.526035	1.405110	3.938729
H	2.438610	1.106973	2.440808
O	-0.643451	-1.893801	-1.594432
O	-0.502716	-1.901373	1.978218
O	0.630795	1.679485	-1.596904
O	-2.227679	0.643726	0.368120
C	4.185120	2.783435	-0.755811
C	-0.457239	2.477983	2.577190
H	0.108842	4.347470	3.521012
C	-1.221566	-1.583417	-2.860353
C	-0.857108	-3.280616	-1.300848
C	0.567469	-2.719545	2.445331
C	-1.206468	-1.458274	3.139687
C	1.239448	1.485027	-2.873818
C	0.533703	3.081945	-1.321817
C	-3.527347	0.769491	0.081000
H	3.740189	3.735330	-0.487857
H	-1.259978	2.511105	1.844577
H	-0.883870	2.221652	3.553072
C	-2.376154	-2.545587	-2.995486
H	-0.475361	-1.738223	-3.647742
H	-1.522118	-0.536937	-2.847938
C	-1.795036	-3.810122	-2.379395
H	-1.293345	-3.339844	-0.303493
H	0.106052	-3.792531	-1.295079
H	0.171057	-3.702481	2.724655
H	1.289863	-2.823604	1.639364
C	1.100936	-1.963759	3.642422
H	-1.971545	-2.199105	3.390519
H	-1.701829	-0.521678	2.887677
C	-0.159598	-1.338767	4.249058
C	0.870732	2.724989	-3.651884
H	0.850831	0.559027	-3.294316
H	2.322283	1.392350	-2.752009
C	1.000158	3.798117	-2.581368
H	-0.505615	3.298951	-1.077019
H	1.156378	3.317935	-0.456295
C	-4.214708	-0.572508	-0.231197
C	-3.661917	1.746276	-1.119442
C	-4.264033	1.376109	1.308204
H	-2.688878	-2.680231	-4.029861
H	-3.226269	-2.186201	-2.415551
H	-2.560773	-4.463281	-1.964597
H	-1.234910	-4.374366	-3.126272
H	1.635125	-2.608665	4.338218
H	1.789584	-1.197094	3.288772
H	-0.484900	-1.874218	5.140279
H	0.003506	-0.299887	4.529304
H	-0.158918	2.652914	-4.006619
H	1.524719	2.892479	-4.506145
H	0.405877	4.686312	-2.788449
H	2.043781	4.098980	-2.483057
C	-5.357478	-0.715030	-1.003400
C	-3.651088	-1.703599	0.333198
F	-3.244552	1.150269	-2.248873
F	-4.907862	2.196106	-1.353242
F	-2.899434	2.832999	-0.956998
F	-5.595772	1.451934	1.164366
F	-3.833162	2.613747	1.597065
F	-4.032442	0.622174	2.391011
C	-5.906461	-1.966307	-1.220032
H	-5.835369	0.146296	-1.448470
C	-4.209925	-2.951396	0.137095
H	-2.759838	-1.590586	0.932606
C	-5.338552	-3.089226	-0.648582
H	-6.791913	-2.059812	-1.837737
H	-3.756411	-3.821754	0.597855
H	-5.775776	-4.066864	-0.814380

95
structure_Sr_replacing_Ca_2h.xyz

Sr	-0.001980	-0.002463	0.047245
O	-2.275503	-0.542339	0.036461
C	-3.567603	-0.760449	0.292718
C	-3.681005	-1.447462	1.680122
F	-3.186978	-0.630085	2.622787
F	-4.931640	-1.760318	2.054827
F	-2.969410	-2.581308	1.741302
C	-4.114715	-1.701482	-0.813063
F	-4.099335	-1.061003	-1.993200
F	-5.371498	-2.137483	-0.624453
F	-3.354158	-2.794295	-0.956933
C	-4.415735	0.526087	0.329026
C	-3.742913	1.729183	0.217777
H	-2.667722	1.706586	0.112526
C	-5.799854	2.944090	0.386675
H	-6.340159	3.883348	0.407209
C	-6.480431	1.746548	0.503646
H	-7.558141	1.743123	0.617619
C	-5.795712	0.545695	0.477344
H	-6.352170	-0.375302	0.575581
O	-0.507269	1.597845	-1.928909
C	-0.042822	2.352359	-4.126824
H	-0.274881	3.402946	-4.304600
C	-1.325455	1.531649	-4.094074
H	-1.127111	0.484591	-4.327630
H	-2.085257	1.897831	-4.782593
C	-1.736809	1.664130	-2.647606
H	-2.381892	0.871204	-2.274676
H	-2.220564	2.629273	-2.460212
O	0.521483	-2.175255	1.340170
O	0.507977	-1.585514	-1.940937
O	-0.508346	2.163439	1.357260
O	2.273227	0.535409	0.032449
C	-4.425491	2.930573	0.245489
C	0.537074	2.165959	-2.727025
H	0.650875	2.031054	-4.902366
C	1.149376	-2.367925	2.612202
C	0.101465	-3.430341	0.795889
C	-0.536718	-2.135629	-2.751258
C	1.737708	-1.632365	-2.661340
C	-1.138724	2.344723	2.629607
C	-0.089295	3.423567	0.824116
C	3.563775	0.753992	0.296173
H	-3.878464	3.862238	0.153244
H	0.856556	3.109688	-2.281963
H	1.383847	1.480121	-2.699091
C	1.324977	-3.865337	2.754832
H	0.490530	-1.966665	3.387396
H	2.089183	-1.816434	2.621235
C	0.146515	-4.398790	1.955983
H	0.799645	-3.719710	0.004010
H	-0.890902	-3.298191	0.366403
H	-0.862939	-3.084144	-2.321625
H	-1.379953	-1.445545	-2.716696
C	0.046987	-2.303073	-4.151499
H	2.226724	-2.597929	-2.490714
H	2.378335	-0.842489	-2.274536
C	1.325329	-1.476808	-4.105167
C	-1.315715	3.840735	2.785052
H	-0.481024	1.937276	3.402549
H	-2.078261	1.792512	2.632470
C	-0.136516	4.381888	1.992485
H	0.903410	3.296018	0.394165
H	-0.787471	3.719120	0.034465
C	4.415234	-0.530706	0.320323
C	3.671550	1.424086	1.692414
C	4.111527	1.710972	-0.795474
H	1.323822	-4.182302	3.796347
H	2.267281	-4.178432	2.301542
H	0.274115	-5.429918	1.630085
H	-0.773965	-4.330154	2.537975
H	0.284821	-3.350381	-4.340824
H	-0.646424	-1.975951	-4.924841
H	2.086816	-1.826936	-4.800146
H	1.121148	-0.426929	-4.320246
H	-1.316277	4.148550	3.829309
H	-2.257596	4.157263	2.333245
H	0.783221	4.308865	2.575100
H	-0.264399	5.415706	1.675355
C	5.795512	-0.548430	0.466162
C	3.744687	-1.734429	0.202173
F	3.171776	0.596327	2.622933
F	4.920927	1.730068	2.076641
F	2.961543	2.558364	1.764251
F	5.364586	2.152503	-0.594587
F	3.345080	2.800748	-0.930492
F	4.106559	1.084658	-1.983235
C	6.482795	-1.747976	0.483226
H	6.350265	0.372984	0.570103
C	4.429808	-2.934563	0.221125
H	2.669270	-1.713215	0.099077
C	5.804429	-2.946152	0.359931
H	7.560693	-1.743069	0.595346
H	3.884571	-3.866801	0.124036
H	6.346687	-3.884415	0.373559

95
structure_Sr_replacing_Ca_2i.xyz

38	0.086879	-0.077592	0.316648
8	2.334543	-0.762534	0.013997
6	3.626789	-0.516972	-0.233582
6	3.836338	-0.586043	-1.770654
9	3.247534	0.465216	-2.365124
9	5.117728	-0.597650	-2.177102
9	3.271849	-1.683210	-2.291236
6	4.501723	-1.614117	0.436215
9	4.249395	-1.658377	1.751078
9	5.821039	-1.409264	0.300879
9	4.242866	-2.837237	-0.048152
6	4.094808	0.835232	0.332661
6	3.484223	1.253404	1.504760
1	2.733860	0.619613	1.957013
6	4.768167	3.274646	1.501809
1	5.027531	4.225731	1.951935
6	5.387856	2.862867	0.337983
1	6.136649	3.490582	-0.130708
6	5.062890	1.646394	-0.237212
1	5.573614	1.348072	-1.141861
8	0.462302	-0.320063	2.866285
6	-1.126481	-1.709636	3.810444
1	-1.710987	-1.913005	4.706192
6	0.188289	-2.494126	3.757880
1	0.116310	-3.348537	3.087376
1	0.473418	-2.866937	4.741019
6	1.211821	-1.474803	3.252487
1	1.779533	-1.801553	2.383009
1	1.914510	-1.188334	4.040329
8	-0.322785	0.229781	-2.218351
8	-0.259058	-2.640457	0.303152
8	0.661823	2.435507	0.128292
8	-2.241125	-0.009466	0.717115
6	3.819319	2.457761	2.090093
6	-0.669558	-0.269248	3.731226
1	-1.747271	-1.926938	2.941749
6	-1.049036	1.289039	-2.858592
6	0.007772	-0.777810	-3.172963
6	0.707590	-3.569405	-0.182243
6	-1.475672	-3.373009	0.381022
6	1.558382	3.019218	-0.814264
6	0.292592	3.494156	1.001854
6	-3.511994	0.386342	0.595071
1	3.333569	2.760356	3.010622
1	-0.357012	0.115028	4.708801
1	-1.405006	0.398001	3.287794
6	-1.152748	0.900640	-4.323112
1	-0.484535	2.212552	-2.718486
1	-2.020054	1.393527	-2.376771
6	0.014654	-0.058197	-4.499935
1	-0.759665	-1.559642	-3.154309
1	0.965237	-1.209268	-2.888721
1	1.569688	-3.001490	-0.523175
1	1.014399	-4.217598	0.646425
6	-0.022317	-4.364042	-1.258334
1	-2.290069	-2.661427	0.489106
1	-1.444063	-4.018096	1.266718
6	-1.507081	-4.200966	-0.894709
6	0.991626	4.406906	-1.101853
1	1.609433	2.361343	-1.678851
1	2.552398	3.080122	-0.364495
6	0.048009	4.675120	0.079686
1	-0.582135	3.182802	1.568230
1	1.121219	3.690080	1.690883
6	-4.141391	0.030930	-0.766305
6	-3.548466	1.924700	0.801302
6	-4.362540	-0.306136	1.694648
1	-1.104016	1.767482	-4.979919
1	-2.096145	0.385073	-4.507723
1	-0.106574	-0.735304	-5.344014
1	0.952777	0.486117	-4.622163
1	0.184815	-3.953408	-2.245732
1	0.294796	-5.405801	-1.262492
1	-2.044461	-3.667650	-1.678407
1	-2.011837	-5.153366	-0.740422
1	0.447648	4.426837	-2.045308
1	1.791665	5.142806	-1.167275
1	-0.990203	4.679234	-0.249235
1	0.249246	5.623591	0.575176
6	-5.370706	0.501810	-1.205591
6	-3.432921	-0.832129	-1.582359
9	-2.924087	2.530870	-0.222073
9	-4.772141	2.469963	0.887906
9	-2.897172	2.286812	1.915749
9	-5.665069	0.019033	1.676338
9	-3.915617	-0.031380	2.927015
9	-4.293557	-1.636098	1.541581
6	-5.864407	0.126405	-2.441431
1	-5.959276	1.164793	-0.587922
6	-3.929594	-1.218631	-2.812435
1	-2.474846	-1.189253	-1.233980
6	-5.147896	-0.735609	-3.250053
1	-6.823291	0.509103	-2.770668
1	-3.362195	-1.902509	-3.433798
1	-5.540832	-1.032626	-4.215338

95
structure_Sr_replacing_Ca_2j.xyz

Sr	-0.004037	0.001440	-0.048135
O	2.261272	-0.598950	0.239977
C	3.574815	-0.723262	0.024140
C	3.766672	-1.657835	-1.201365
F	3.261278	-1.069540	-2.296508
F	5.036546	-1.985059	-1.488555
F	3.108442	-2.816138	-1.047116
C	4.224658	-1.366107	1.279687
F	4.082206	-0.545094	2.330386
F	5.537640	-1.620098	1.161495
F	3.639116	-2.524185	1.613444
C	4.292388	0.615993	-0.238826
C	3.557772	1.774365	-0.062124
H	2.524054	1.688797	0.238260
C	5.453691	3.118657	-0.641428
H	5.906645	4.090717	-0.797786
C	6.196835	1.966808	-0.818618
H	7.237102	2.031499	-1.115366
C	5.624877	0.724939	-0.613157
H	6.233985	-0.157358	-0.745622
O	0.480092	1.754501	1.790803
C	-0.716208	2.222483	3.728544
H	-1.001455	3.057232	4.366758
C	0.629907	1.597825	4.126471
H	0.495750	0.580916	4.492791
H	1.134800	2.162857	4.908574
C	1.440352	1.613177	2.833605
H	2.012967	0.710703	2.635873
H	2.121388	2.470093	2.803439
O	-0.437293	-1.190097	-2.286147
O	-0.507065	-2.147965	1.317109
O	0.501613	1.958828	-1.645449
O	-2.260002	0.530002	0.354979
C	4.130768	3.016921	-0.256653
C	-0.498653	2.660337	2.290789
H	-1.516764	1.485100	3.770578
C	-1.628056	-0.919630	-3.035107
C	0.028859	-2.483915	-2.650927
C	0.365546	-3.288441	1.266023
C	-0.835095	-1.861652	2.676707
C	1.312805	1.834661	-2.821471
C	0.263285	3.336595	-1.350719
C	-3.588647	0.651610	0.265598
H	3.538642	3.912190	-0.103077
H	-0.096524	3.678690	2.238375
H	-1.381625	2.576789	1.662945
C	-2.169584	-2.274029	-3.494675
H	-1.376832	-0.273194	-3.879650
H	-2.312144	-0.388967	-2.376804
C	-1.239432	-3.284550	-2.829950
H	0.686531	-2.842143	-1.861420
H	0.602494	-2.418886	-3.581921
H	-0.133621	-4.086881	0.713692
H	1.267984	-2.983784	0.737079
C	0.656611	-3.665496	2.710985
H	-1.757776	-2.383808	2.949296
H	-1.016215	-0.790142	2.764525
C	0.348924	-2.377565	3.458959
C	1.723273	3.246161	-3.189456
H	0.707878	1.369176	-3.603178
H	2.158434	1.186731	-2.594060
C	0.574610	4.072845	-2.633827
H	-0.768414	3.439458	-1.018183
H	0.930531	3.646733	-0.540369
C	-4.276999	-0.596577	-0.315875
C	-3.898391	1.907989	-0.595079
C	-4.185841	0.862190	1.686440
H	-2.115737	-2.358341	-4.580111
H	-3.206545	-2.410901	-3.194350
H	-1.626361	-3.574562	-1.852614
H	-1.090401	-4.184339	-3.424959
H	-0.011157	-4.461535	3.044543
H	1.682951	-4.001814	2.841562
H	0.125200	-2.531938	4.513424
H	1.185824	-1.681736	3.372871
H	1.863299	3.365268	-4.262555
H	2.657103	3.506864	-2.689663
H	-0.285504	4.038636	-3.305029
H	0.835752	5.115678	-2.460405
C	-5.494064	-0.588917	-0.979921
C	-3.635167	-1.807226	-0.115482
F	-3.627454	1.664910	-1.887291
F	-5.171121	2.338913	-0.535329
F	-3.136462	2.948567	-0.235833
F	-5.526731	0.859470	1.719985
F	-3.787624	2.018703	2.239838
F	-3.773879	-0.118562	2.499844
C	-6.047211	-1.768432	-1.444843
H	-6.029984	0.335823	-1.142276
C	-4.198205	-2.988331	-0.559242
H	-2.683945	-1.812257	0.398944
C	-5.405639	-2.973646	-1.231865
H	-6.994144	-1.742084	-1.971176
H	-3.687844	-3.927698	-0.379190
H	-5.846238	-3.897513	-1.588069

95
structure_Sr_replacing_Ca_2k.xyz

Sr	0.088970	0.040748	0.220370
O	-2.202719	0.453101	0.632813
C	-3.460546	0.754093	0.296381
C	-3.456114	2.163706	-0.355156
F	-2.874563	2.105119	-1.565182
F	-4.664919	2.723490	-0.530961
F	-2.743616	3.035183	0.369601
C	-4.335178	0.790528	1.580149
F	-4.267887	-0.394077	2.204150
F	-5.634913	1.034616	1.352055
F	-3.912793	1.714984	2.453490
C	-4.099965	-0.276605	-0.652547
C	-3.529719	-1.536838	-0.672203
H	-2.659350	-1.707168	-0.054914
C	-5.150327	-2.296480	-2.260445
H	-5.560026	-3.079860	-2.887126
C	-5.722175	-1.038222	-2.254981
H	-6.583268	-0.830676	-2.879238
C	-5.210961	-0.038172	-1.447221
H	-5.693411	0.929030	-1.446255
O	-0.349018	-2.143527	1.546149
C	-0.407495	-4.435001	1.124897
H	-0.460539	-4.577486	0.045876
C	-1.769096	-3.995531	1.687043
H	-2.138855	-4.665874	2.461360
H	-2.523087	-3.954868	0.902023
C	-1.498234	-2.603902	2.245907
H	-1.264865	-2.652880	3.315167
H	-2.297262	-1.883269	2.090322
O	0.834886	2.239531	-0.947075
O	0.557424	1.222720	2.478437
O	-0.384982	-0.836114	-2.179423
O	2.298758	-0.764028	0.360250
C	-4.053172	-2.544808	-1.457763
C	0.515894	-3.273742	1.461809
H	-0.052282	-5.366583	1.562640
C	1.692692	2.384159	-2.081689
C	0.673204	3.513047	-0.305421
C	-0.523947	1.813179	3.194728
C	1.321746	0.495701	3.441478
C	-0.841363	-0.049607	-3.277224
C	-0.567047	-2.222755	-2.487967
C	3.563197	-0.804415	-0.070991
H	-3.598138	-3.529011	-1.448812
H	1.283782	-3.054270	0.724666
H	0.998630	-3.418895	2.434482
C	1.506977	3.819162	-2.510925
H	1.392875	1.648816	-2.825401
H	2.726147	2.187130	-1.786199
C	1.420086	4.521293	-1.164729
H	1.068622	3.448841	0.709805
H	-0.394960	3.725123	-0.255028
H	-0.153862	2.686324	3.744232
H	-1.279578	2.117878	2.474315
C	-0.984286	0.713403	4.128523
H	2.038547	1.179078	3.907293
H	1.871697	-0.277801	2.907338
C	0.313297	-0.032601	4.462687
C	-1.956673	-0.864164	-3.884421
H	-0.020669	0.090674	-3.989766
H	-1.152725	0.918770	-2.890712
C	-1.399651	-2.276013	-3.764827
H	0.410385	-2.692520	-2.604148
H	-1.078944	-2.682592	-1.641263
C	4.213318	0.588029	-0.155202
C	3.566391	-1.496841	-1.461632
C	4.412599	-1.646168	0.919389
H	0.574934	3.930239	-3.068532
H	2.329021	4.177855	-3.128353
H	2.422646	4.687760	-0.769829
H	0.904441	5.479088	-1.209305
H	-1.491634	1.103288	5.009448
H	-1.680659	0.068258	3.593958
H	0.650459	0.174395	5.477589
H	0.187833	-1.109755	4.370300
H	-2.171724	-0.577724	-4.912779
H	-2.863923	-0.751302	-3.290535
H	-0.764505	-2.507352	-4.620815
H	-2.180958	-3.031715	-3.709243
C	5.259206	0.922430	-1.001200
C	3.698718	1.562079	0.683592
F	3.039781	-0.676195	-2.384989
F	4.776361	-1.871268	-1.914554
F	2.811334	-2.602636	-1.458825
F	5.722144	-1.675825	0.625825
F	3.998912	-2.920188	0.986119
F	4.302368	-1.135968	2.152958
C	5.753832	2.214322	-1.028100
H	5.697793	0.182773	-1.656473
C	4.204082	2.846990	0.673013
H	2.881416	1.298781	1.339362
C	5.228753	3.181981	-0.192885
H	6.561084	2.462303	-1.707247
H	3.789912	3.592719	1.342127
H	5.621218	4.192036	-0.213187

95

structure_Sr_replacing_Ca_2l.xyz

Sr	0.009118	0.208193	-0.055956
O	-2.287613	0.474196	-0.586381
C	-3.542756	0.016985	-0.649939
C	-3.582065	-1.124958	-1.703440
F	-2.962469	-2.215072	-1.219593
F	-4.810155	-1.512403	-2.089004
F	-2.930924	-0.783069	-2.821838
C	-4.481934	1.164287	-1.111023
F	-4.371346	2.203049	-0.271620
F	-5.780604	0.827576	-1.142026
F	-4.165548	1.616019	-2.333068
C	-4.070947	-0.485146	0.706323
C	-3.479656	0.050810	1.837100
H	-2.695515	0.781702	1.704703
C	-4.856506	-1.302478	3.251723
H	-5.159357	-1.626222	4.240668
C	-5.459960	-1.836617	2.129175
H	-6.239569	-2.581968	2.234049
C	-5.080069	-1.422375	0.864942
H	-5.578805	-1.848255	0.005639
O	-0.258075	2.412008	1.293024
C	0.310477	4.529849	2.165425
H	0.127050	4.512847	3.241215
C	-1.008810	4.616059	1.413972
H	-0.851693	4.946210	0.385748
H	-1.730442	5.284238	1.881293
C	-1.457959	3.174631	1.438601
H	-2.136183	2.887585	0.637301
H	-1.925255	2.932544	2.399493
O	0.630684	-2.025454	-1.229719
O	0.403718	1.521468	-2.272863
O	-0.574225	-1.525864	1.789813
O	2.291148	0.667607	0.352909
C	-3.869717	-0.345882	3.100670
C	0.874412	3.199283	1.699616
H	0.987326	5.355384	1.951286
C	1.441762	-3.073500	-0.695064
C	0.368912	-2.280946	-2.616186
C	-0.704403	1.986776	-3.039661
C	1.367600	2.573545	-2.303634
C	-1.354047	-2.710211	1.607996
C	-0.309041	-1.336244	3.185703
C	3.553446	0.286210	0.568064
H	-3.396852	0.090992	3.972967
H	1.419952	2.659344	2.471769
H	1.537474	3.304728	0.840814
C	1.118880	-4.271228	-1.555137
H	1.190210	-3.186449	0.358193
H	2.496071	-2.798096	-0.776918
C	1.006733	-3.625851	-2.927859
H	0.787372	-1.466797	-3.210732
H	-0.712611	-2.295819	-2.753366
H	-0.454050	1.918896	-4.104615
H	-1.558287	1.354449	-2.812928
C	-0.872424	3.426080	-2.597204
H	1.963819	2.476713	-3.216537
H	2.020405	2.444139	-1.442667
C	0.564683	3.875148	-2.304721
C	-0.998161	-3.576397	2.791692
H	-1.097349	-3.132969	0.638232
H	-2.415884	-2.451961	1.609174
C	-0.908188	-2.538166	3.899478
H	0.771676	-1.275784	3.314394
H	-0.754792	-0.392732	3.507202
C	4.104795	-0.618780	-0.548681
C	3.601890	-0.443384	1.938950
C	4.468801	1.539577	0.634276
H	0.165729	-4.710724	-1.253466
H	1.888769	-5.039543	-1.504607
H	2.001681	-3.484986	-3.351418
H	0.411937	-4.205637	-3.631617
H	-1.364930	4.036276	-3.352570
H	-1.482250	3.452210	-1.693938
H	0.939991	4.558003	-3.065797
H	0.636823	4.384259	-1.345124
H	-0.031896	-4.059803	2.632979
H	-1.745029	-4.344854	2.984553
H	-0.298329	-2.856700	4.743120
H	-1.907839	-2.302408	4.266127
C	5.130124	-1.537650	-0.387459
C	3.516870	-0.490482	-1.795589
F	3.016156	-1.649398	1.837792
F	4.833280	-0.648449	2.438991
F	2.925163	0.229714	2.876767
F	5.775259	1.254254	0.749933
F	4.153879	2.346049	1.657829
F	4.327854	2.260912	-0.486602
C	5.531863	-2.333323	-1.445600
H	5.624623	-1.651516	0.567178
C	3.929816	-1.269087	-2.858572
H	2.714415	0.223128	-1.915667
C	4.933645	-2.203865	-2.684589
H	6.324039	-3.057831	-1.297478
H	3.459401	-1.148741	-3.827879
H	5.253284	-2.825539	-3.512831

95

structure_Sr_replacing_Ca_2m.xyz

Sr	-0.075952	-0.150233	-0.300823
O	2.240233	-0.188511	-0.771261
C	3.495568	0.253279	-0.649868
C	3.522504	1.735121	-1.115049
F	2.873102	2.500979	-0.222449
F	4.743446	2.271784	-1.274350
F	2.889687	1.893693	-2.285598
C	4.419127	-0.591856	-1.567966
F	4.338105	-1.883549	-1.218944
F	5.716880	-0.254317	-1.504921
F	4.059337	-0.512349	-2.856051
C	4.046440	0.140976	0.784572
C	3.362425	-0.688896	1.654417
H	2.479574	-1.192151	1.288352
C	4.899691	-0.182879	3.417935
H	5.231855	-0.305864	4.442118
C	5.592924	0.645436	2.555480
H	6.472496	1.175620	2.901263
C	5.177539	0.798758	1.245250
H	5.748656	1.439109	0.588075
O	0.260301	-2.683826	0.112485
C	0.079404	-4.179324	1.897836
H	-0.049175	-3.629639	2.829610
C	1.540643	-4.123668	1.422768
H	2.002758	-5.107671	1.361020
H	2.146991	-3.517589	2.095411
C	1.447612	-3.464722	0.054373
H	1.337873	-4.216912	-0.735269
H	2.273099	-2.804105	-0.197871
O	-0.674419	2.352561	-0.535036
O	-0.437968	-0.705485	-2.803405
O	0.420131	0.673036	2.106959
O	-2.323767	-0.774348	0.081521
C	3.784667	-0.857963	2.958626
C	-0.696941	-3.511413	0.769953
H	-0.267080	-5.197735	2.066959
C	-1.539769	3.089285	0.327324
C	-0.337332	3.240551	-1.592268
C	0.693358	-0.795374	-3.666445
C	-1.222942	-1.872738	-3.054979
C	1.129102	1.857830	2.473582
C	0.155045	-0.108369	3.278322
C	-3.608901	-0.503016	0.335127
H	3.238816	-1.519779	3.621826
H	-1.525206	-2.879785	1.079796
H	-1.067801	-4.252254	0.052852
C	-1.005242	4.520863	0.316407
H	-1.521651	2.609097	1.303308
H	-2.557456	3.039117	-0.066648
C	-0.064216	4.559772	-0.895574
H	-1.188172	3.317589	-2.277589
H	0.518830	2.830656	-2.123938
H	0.388993	-0.522862	-4.683357
H	1.450632	-0.103940	-3.304775
C	1.106847	-2.248642	-3.571139
H	-1.895537	-1.668047	-3.893355
H	-1.821738	-2.063403	-2.165896
C	-0.227366	-2.983098	-3.395949
C	0.692975	2.125599	3.893443
H	0.866661	2.637112	1.760635
H	2.204931	1.673435	2.413237
C	0.648551	0.714241	4.459465
H	-0.918012	-0.292741	3.319142
H	0.673285	-1.065919	3.191238
C	-4.116041	0.738471	-0.419056
C	-3.761922	-0.325114	1.870426
C	-4.489184	-1.700981	-0.118198
H	-0.470271	4.755292	1.235889
H	-1.823657	5.233040	0.222057
H	-0.248428	5.411701	-1.548116
H	0.976365	4.597504	-0.575416
H	1.665124	-2.579433	-4.445439
H	1.741571	-2.373722	-2.694418
H	-0.527258	-3.500546	-4.306419
H	-0.172876	-3.721605	-2.598196
H	-0.299841	2.580051	3.904986
H	1.382287	2.778720	4.426165
H	-0.003883	0.615990	5.325283
H	1.652441	0.400670	4.748261
C	-5.098690	1.600811	0.040234
C	-3.528700	0.994104	-1.647766
F	-3.191627	0.827415	2.261482
F	-5.027408	-0.308163	2.323947
F	-3.138951	-1.302966	2.539868
F	-5.806857	-1.486478	0.017726
F	-4.203248	-2.824483	0.555842
F	-4.270042	-1.960437	-1.413954
C	-5.460087	2.712203	-0.701149
H	-5.591890	1.424858	0.985951
C	-3.901188	2.091441	-2.398232
H	-2.764448	0.320192	-2.009197
C	-4.863505	2.963267	-1.921682
H	-6.219376	3.384172	-0.318611
H	-3.433901	2.267413	-3.360328
H	-5.151684	3.831897	-2.502118

95

structure_Sr_replacing_Ca_2n.xyz

Sr	0.009603	0.023366	-0.007147
O	2.278259	-0.533445	-0.014078
C	3.560048	-0.740300	-0.325384
C	3.624995	-1.348266	-1.752189
F	3.082504	-0.486961	-2.626405
F	4.863340	-1.620790	-2.192934
F	2.925802	-2.487967	-1.848698
C	4.136820	-1.743181	0.708326
F	4.179364	-1.163532	1.918727
F	5.377626	-2.187824	0.445783
F	3.365210	-2.830997	0.828604
C	4.411661	0.544437	-0.322932
C	3.742179	1.744433	-0.168175
H	2.667831	1.719697	-0.057624
C	5.800131	2.960452	-0.312302
H	6.342249	3.898892	-0.306151
C	6.477480	1.766101	-0.472739
H	7.554370	1.764427	-0.594339
C	5.790288	0.566373	-0.482076
H	6.343862	-0.351794	-0.616882
O	0.555192	1.571715	1.994634
C	0.086009	2.207872	4.225873
H	0.352299	3.246504	4.426110
C	1.341931	1.350129	4.164877
H	1.105793	0.299238	4.338145
H	2.103896	1.653356	4.881033
C	1.776443	1.549383	2.732984
H	2.397517	0.755851	2.321748
H	2.297548	2.504654	2.606613
O	-0.500272	-2.113532	-1.370942
O	-0.479144	-1.611968	1.938892
O	0.517731	2.210589	-1.278163
O	-2.271522	0.521536	0.020016
C	4.426566	2.944995	-0.162981
C	-0.500456	2.069974	2.825904
H	-0.615763	1.889994	4.995538
C	-1.124263	-2.258333	-2.650934
C	-0.095255	-3.389487	-0.865979
C	0.551870	-2.240686	2.706671
C	-1.714779	-1.735661	2.637511
C	0.950586	2.317796	-2.634916
C	0.268768	3.512529	-0.733279
C	-3.557789	0.758932	-0.246745
H	3.881761	3.874395	-0.039655
H	-0.854941	3.019808	2.423144
H	-1.325365	1.357926	2.778465
C	-1.320893	-3.747859	-2.839980
H	-0.455506	-1.844126	-3.410946
H	-2.056192	-1.693454	-2.647799
C	-0.152631	-4.322243	-2.054681
H	-0.796980	-3.694464	-0.082922
H	0.898880	-3.282658	-0.433724
H	0.828442	-3.184532	2.232928
H	1.423505	-1.587191	2.683178
C	-0.019559	-2.448074	4.109321
H	-2.173437	-2.703853	2.406188
H	-2.373468	-0.943239	2.288199
C	-1.319474	-1.652278	4.092274
C	0.477826	3.680536	-3.085537
H	0.514543	1.493285	-3.199648
H	2.040671	2.233389	-2.671182
C	0.665327	4.497618	-1.817404
H	-0.792337	3.576771	-0.485763
H	0.851483	3.622870	0.182149
C	-4.412139	-0.520296	-0.343925
C	-3.645030	1.500714	-1.607778
C	-4.115904	1.660018	0.886295
H	-1.320747	-4.032826	-3.890707
H	-2.269165	-4.060716	-2.399306
H	-0.296659	-5.360487	-1.759701
H	0.770107	-4.250615	-2.632598
H	-0.227007	-3.504482	4.281378
H	0.670036	-2.117616	4.884609
H	-2.078030	-2.059438	4.758790
H	-1.145704	-0.610892	4.367239
H	-0.576851	3.639404	-3.362458
H	1.047745	4.059789	-3.932182
H	0.057984	5.400719	-1.791196
H	1.712712	4.784758	-1.708256
C	-5.787781	-0.527942	-0.528563
C	-3.747865	-1.729637	-0.249474
F	-3.146578	0.714919	-2.575398
F	-4.886829	1.841883	-1.986951
F	-2.920408	2.627908	-1.616714
F	-5.371446	2.101133	0.700148
F	-3.357705	2.747012	1.076450
F	-4.112105	0.977720	2.042701
C	-6.476953	-1.723983	-0.607855
H	-6.337151	0.398595	-0.614068
C	-4.435040	-2.926027	-0.329135
H	-2.675525	-1.713844	-0.116654
C	-5.805238	-2.927998	-0.507251
H	-7.551221	-1.711640	-0.750220
H	-3.895080	-3.862925	-0.248588
H	-6.349192	-3.863354	-0.568912

95

structure_Sr_replacing_Ca_2o.xyz

38	-0.113089	0.027532	0.288613
8	-2.347163	0.767896	0.169465
6	-3.611081	0.635864	-0.245872
6	-3.655187	0.994794	-1.756851
9	-3.088393	0.017347	-2.483220
9	-4.885994	1.193085	-2.262012
9	-2.961524	2.110415	-2.014168
6	-4.511086	1.634309	0.533713
9	-4.368423	1.437804	1.850973
9	-5.819807	1.514613	0.261925
9	-4.177296	2.911141	0.292927
6	-4.179666	-0.772016	0.003857
6	-3.657068	-1.465182	1.083165
1	-2.885550	-0.994864	1.675942
6	-5.069007	-3.341356	0.613468
1	-5.413611	-4.342037	0.846736
6	-5.597808	-2.656359	-0.463834
1	-6.359751	-3.118265	-1.080678
6	-5.165732	-1.375604	-0.760270
1	-5.608218	-0.858632	-1.600485
8	-0.536345	-0.498499	2.786416
6	0.978740	0.545918	4.191481
1	1.503644	0.457482	5.141284
6	-0.352973	1.297457	4.308077
1	-0.278651	2.303839	3.900229
1	-0.680516	1.383790	5.343353
6	-1.334523	0.445754	3.501366
1	-1.918856	1.002570	2.770145
1	-2.019862	-0.100418	4.156746
8	0.294024	0.185703	-2.271488
8	0.242506	2.498140	0.982029
8	-0.764185	-2.402462	-0.286014
8	2.189853	-0.222967	0.744288
6	-4.100781	-2.735499	1.393368
6	0.571116	-0.800679	3.630886
1	1.644166	1.035327	3.480934
6	0.777928	-0.866463	-3.102147
6	0.404240	1.427799	-2.974761
6	-0.695198	3.523355	0.658682
6	1.394570	3.186104	1.452960
6	-1.597115	-2.866747	-1.344799
6	-0.434890	-3.564874	0.463297
6	3.458679	-0.551740	0.484028
1	-3.683732	-3.256279	2.247708
1	0.242651	-1.488055	4.418662
1	1.341289	-1.269820	3.022773
6	1.849577	-0.218022	-3.943145
1	-0.040718	-1.244582	-3.725024
1	1.136684	-1.664735	-2.455995
6	1.232143	1.145135	-4.224791
1	0.890557	2.141004	-2.306811
1	-0.596063	1.794237	-3.208426
1	-1.485980	3.076106	0.062288
1	-1.130357	3.904206	1.589360
6	0.134103	4.591386	-0.039611
1	2.227541	2.487581	1.457549
1	1.209417	3.525182	2.478323
6	1.553005	4.371831	0.510275
6	-0.949520	-4.165565	-1.806838
1	-1.646304	-2.088435	-2.101800
1	-2.604062	-3.036408	-0.953591
6	-0.139860	-4.627297	-0.584731
1	0.409007	-3.329007	1.107491
1	-1.295880	-3.840601	1.081080
6	4.048247	0.203476	-0.721796
6	3.515344	-2.085166	0.244048
6	4.337096	-0.196025	1.715189
1	2.067555	-0.780858	-4.849536
1	2.766553	-0.118351	-3.362080
1	1.980693	1.915617	-4.399805
1	0.585948	1.095562	-5.102006
1	0.108727	4.446999	-1.119310
1	-0.247258	5.589951	0.167921
1	2.250779	4.132928	-0.291420
1	1.941250	5.244272	1.033461
1	-0.294497	-3.991206	-2.659695
1	-1.701924	-4.892327	-2.109217
1	0.925100	-4.647052	-0.811372
1	-0.427889	-5.618977	-0.239345
6	5.170466	-0.194373	-1.432539
6	3.417039	1.379408	-1.085282
9	2.929669	-2.385642	-0.927595
9	4.747586	-2.619613	0.211149
9	2.844014	-2.754309	1.189626
9	5.645681	-0.439732	1.544879
9	3.957069	-0.859798	2.815438
9	4.219574	1.111057	1.988788
6	5.632258	0.562998	-2.494113
1	5.700064	-1.098550	-1.167547
6	3.890631	2.151490	-2.127733
1	2.539423	1.674319	-0.528506
6	4.999022	1.740831	-2.843528
1	6.503043	0.230397	-3.046686
1	3.387754	3.076258	-2.387740
1	5.370042	2.336145	-3.669468

95
structure_Sr_replacing_Ca_2p.xyz

Sr	0.018808	0.068038	0.144205
O	-2.263881	-0.464052	0.065470
C	-3.539096	-0.834755	0.212850
C	-3.656072	-1.674413	1.513633
F	-3.378727	-0.900435	2.573712
F	-4.866620	-2.213058	1.733293
F	-2.779685	-2.686808	1.536104
C	-3.932369	-1.702011	-1.012915
F	-3.863898	-0.956051	-2.127169
F	-5.169128	-2.221634	-0.971928
F	-3.093601	-2.733127	-1.184914
C	-4.519203	0.351069	0.301267
C	-3.980673	1.621043	0.211879
H	-2.909007	1.709044	0.102371
C	-6.154715	2.604224	0.405669
H	-6.792846	3.479339	0.443086
C	-6.701714	1.338508	0.505862
H	-7.772076	1.217390	0.624439
C	-5.892797	0.218752	0.452195
H	-6.347253	-0.758565	0.527817
O	-0.500715	1.963046	-1.578037
C	0.052741	2.880477	-3.686299
H	-0.256077	3.926324	-3.722344
C	-1.161387	1.973751	-3.810672
H	-0.864954	0.967151	-4.108061
H	-1.901865	2.338346	-4.520703
C	-1.676065	1.962500	-2.391635
H	-2.266682	1.087709	-2.125904
H	-2.264318	2.861259	-2.178134
O	0.554389	-2.267540	1.098541
O	0.550361	-1.168358	-2.083460
O	-0.412889	1.915946	1.904287
O	2.303295	0.578073	0.196626
C	-4.788079	2.741636	0.258069
C	0.594557	2.533444	-2.308859
H	0.794486	2.722705	-4.467774
C	1.131827	-2.582505	2.370540
C	0.207304	-3.464490	0.397027
C	-0.419874	-1.745551	-2.959743
C	1.821499	-1.236253	-2.722900
C	-1.542472	2.050316	2.773332
C	0.082211	3.221995	1.625322
C	3.604399	0.756124	0.443640
H	-4.345976	3.728443	0.176872
H	0.966965	3.404349	-1.769236
H	1.395166	1.793170	-2.347194
C	1.388400	-4.074094	2.338581
H	0.414976	-2.316067	3.152291
H	2.035678	-1.986955	2.495402
C	0.271588	-4.565896	1.431777
H	0.936581	-3.623709	-0.403974
H	-0.780414	-3.328120	-0.040917
H	-0.643073	-2.762634	-2.630575
H	-1.331504	-1.156893	-2.872475
C	0.194681	-1.725403	-4.361750
H	2.250234	-2.235492	-2.585213
H	2.471927	-0.507297	-2.245912
C	1.508375	-0.971623	-4.176056
C	-2.025392	3.495473	2.634104
H	-1.233266	1.821515	3.795855
H	-2.285172	1.320556	2.457401
C	-1.171768	4.053605	1.499683
H	0.708674	3.562572	2.455544
H	0.696109	3.167154	0.728381
C	4.449279	-0.519925	0.258925
C	3.771505	1.220838	1.915505
C	4.119606	1.860514	-0.517778
H	1.367009	-4.516645	3.333003
H	2.363190	-4.278498	1.892237
H	0.470451	-5.539868	0.987365
H	-0.670216	-4.621038	1.979989
H	0.384652	-2.741930	-4.705922
H	-0.462166	-1.250895	-5.089235
H	2.295389	-1.316387	-4.844739
H	1.373225	0.099454	-4.333051
H	-1.839603	4.049583	3.554354
H	-3.091766	3.543694	2.423416
H	-0.983862	5.122255	1.593029
H	-1.634214	3.864341	0.529118
C	5.836282	-0.550324	0.304332
C	3.766187	-1.704570	0.054031
F	3.282709	0.280756	2.737965
F	5.041621	1.441726	2.291949
F	3.095715	2.348360	2.168644
F	5.373285	2.285042	-0.285931
F	3.342398	2.949690	-0.484221
F	4.092588	1.404732	-1.781426
C	6.517182	-1.741327	0.133178
H	6.401835	0.354138	0.476233
C	4.444962	-2.896627	-0.114862
H	2.686247	-1.673920	0.030972
C	5.825909	-2.919517	-0.078885
H	7.600341	-1.745631	0.167832
H	3.890124	-3.813742	-0.278821
H	6.363043	-3.851220	-0.213025

95
structure_Sr_replacing_Ca_2q.xyz

38	0.115779	-0.040661	0.274023
8	2.353384	-0.771418	0.185373
6	3.613186	-0.644798	-0.244281
6	3.642948	-1.033679	-1.748043
9	3.067891	-0.070795	-2.487539
9	4.868666	-1.241197	-2.261681
9	2.947344	-2.154410	-1.976244
6	4.522193	-1.625393	0.547300
9	4.390015	-1.403104	1.861412
9	5.828234	-1.508146	0.261875
9	4.189398	-2.907581	0.334713
6	4.181627	0.768544	-0.028245
6	3.669976	1.482015	1.043011
1	2.903302	1.023673	1.651192
6	5.079042	3.347797	0.524804
1	5.427138	4.352056	0.736550
6	5.595683	2.643072	-0.545609
1	6.351370	3.092999	-1.178792
6	5.159761	1.357410	-0.813773
1	5.593290	0.824666	-1.648796
8	0.541560	0.576609	2.751447
6	-0.977836	-0.411423	4.191364
1	-1.505601	-0.288803	5.135774
6	0.350669	-1.162875	4.337851
1	0.271986	-2.185062	3.972437
1	0.678613	-1.206737	5.375704
6	1.334957	-0.349076	3.495839
1	1.910263	-0.937015	2.781842
1	2.028980	0.213718	4.127575
8	-0.311338	-0.235688	-2.280873
8	-0.229707	-2.473911	1.092515
8	0.771668	2.377964	-0.315053
8	-2.186305	0.228875	0.735157
6	4.118286	2.757259	1.325404
6	-0.563404	0.913210	3.585926
1	-1.642375	-0.922068	3.495173
6	-0.796856	0.814928	-3.113388
6	-0.435760	-1.481694	-2.975044
6	0.704247	-3.511216	0.798673
6	-1.394012	-3.145561	1.557082
6	1.582162	2.854178	-1.385294
6	0.453339	3.532254	0.451301
6	-3.455231	0.552414	0.469238
1	3.711101	3.294078	2.174588
1	-0.230825	1.625071	4.349980
1	-1.331728	1.365320	2.962755
6	-1.872069	0.165573	-3.948979
1	0.020373	1.189962	-3.739842
1	-1.152939	1.615805	-2.469054
6	-1.255909	-1.198101	-4.229300
1	-0.937453	-2.183399	-2.306328
1	0.560483	-1.863725	-3.201195
1	1.509284	-3.079032	0.210070
1	1.118638	-3.885061	1.741679
6	-0.123376	-4.579050	0.098981
1	-2.221803	-2.441404	1.534167
1	-1.228025	-3.465398	2.591827
6	-1.545881	-4.348849	0.634912
6	0.916524	4.150897	-1.825913
1	1.622597	2.081502	-2.148739
1	2.594977	3.028126	-1.011290
6	0.138159	4.605088	-0.580709
1	-0.378559	3.287826	1.107907
1	1.324223	3.802925	1.056982
6	-4.043885	-0.226109	-0.721904
6	-3.513720	2.081407	0.201073
6	-4.334239	0.220504	1.706910
1	-2.093214	0.726675	-4.855661
1	-2.786891	0.067169	-3.364165
1	-2.004540	-1.967887	-4.407534
1	-0.605322	-1.148729	-5.103346
1	-0.087753	-4.439034	-0.981101
1	0.251029	-5.578633	0.314062
1	-2.238536	-4.126083	-0.175806
1	-1.937225	-5.211292	1.172179
1	0.238660	3.974064	-2.660296
1	1.656166	4.882402	-2.147707
1	-0.931688	4.632323	-0.781951
1	0.439484	5.592187	-0.233521
6	-5.158380	0.164188	-1.448660
6	-3.418976	-1.414545	-1.055236
9	-2.936570	2.361267	-0.979868
9	-4.746556	2.614625	0.167099
9	-2.836103	2.767746	1.129387
9	-5.643361	0.455936	1.529664
9	-3.957601	0.909328	2.792861
9	-4.213112	-1.079960	2.009042
6	-5.618789	-0.612647	-2.496717
1	-5.682560	1.078160	-1.207384
6	-3.890861	-2.205013	-2.084614
1	-2.547989	-1.705268	-0.485308
6	-4.991448	-1.801893	-2.816660
1	-6.483370	-0.285804	-3.062303
1	-3.393291	-3.138820	-2.321633
1	-5.360945	-2.412054	-3.632392

95
structure_Sr_replacing_Ca_2r.xyz

Sr	0.000961	0.005034	0.004531
O	-2.286219	0.482634	-0.003722
C	-3.590010	0.638113	-0.247646
C	-3.755729	1.294303	-1.644851
F	-3.190452	0.509877	-2.574522
F	-5.027265	1.493091	-2.025003
F	-3.144197	2.485820	-1.720648
C	-4.162823	1.575665	0.848279
F	-4.147379	0.943721	2.033033
F	-5.424656	1.991695	0.644698
F	-3.421896	2.681430	0.991901
C	-4.380264	-0.685170	-0.259292
C	-3.648249	-1.857759	-0.227816
H	-2.570681	-1.789288	-0.192895
C	-5.654923	-3.163025	-0.298670
H	-6.152623	-4.125686	-0.312472
C	-6.395062	-1.995940	-0.334747
H	-7.477047	-2.039887	-0.377848
C	-5.764615	-0.765618	-0.320164
H	-6.367441	0.130343	-0.359662
O	-0.463690	-1.653999	1.932943
C	0.008989	-2.418319	4.125986
H	-0.196562	-3.475015	4.299685
C	-1.294119	-1.629919	4.093612
H	-1.124049	-0.580272	4.337826
H	-2.047634	-2.021693	4.774900
C	-1.695443	-1.758953	2.644072
H	-2.358526	-0.978862	2.275421
H	-2.153466	-2.733684	2.443725
O	0.422360	2.150006	-1.349927
O	0.474677	1.601139	1.980463
O	-0.432645	-2.105935	-1.394844
O	2.286659	-0.477771	-0.019469
C	-4.276175	-3.088784	-0.248683
C	0.588012	-2.212688	2.728027
H	0.692197	-2.082126	4.904507
C	0.894537	2.319153	-2.681849
C	-0.098006	3.395795	-0.869293
C	-0.587447	2.101634	2.801182
C	1.699772	1.648526	2.710287
C	-0.933658	-2.251446	-2.718430
C	0.113936	-3.357106	-0.961164
C	3.589627	-0.624991	-0.272270
H	-3.682109	-3.995727	-0.225693
H	0.921341	-3.148550	2.276233
H	1.424760	-1.514322	2.706138
C	-0.019933	3.371463	-3.262604
H	0.845532	1.352942	-3.182992
H	1.938434	2.650054	-2.663663
C	-0.189008	4.318277	-2.081359
H	0.572706	3.785427	-0.100161
H	-1.069217	3.188902	-0.420677
H	-0.940500	3.049482	2.391992
H	-1.410321	1.387942	2.749711
C	-0.011118	2.251979	4.205909
H	2.174454	2.625145	2.566792
H	2.355360	0.878984	2.307502
C	1.281152	1.448785	4.146850
C	0.006414	-3.247893	-3.354282
H	-0.936692	-1.269161	-3.188596
H	-1.962884	-2.624656	-2.684961
C	0.240834	-4.226711	-2.210038
H	1.074156	-3.144208	-0.492853
H	-0.555138	-3.796322	-0.217338
C	4.380861	0.697053	-0.232999
C	3.751379	-1.225307	-1.694800
C	4.163469	-1.606244	0.784405
H	-0.975114	2.920714	-3.534200
H	0.401067	3.856654	-4.141856
H	0.620194	5.049516	-2.065809
H	-1.132723	4.859883	-2.107604
H	0.208516	3.299294	4.416208
H	-0.701151	1.897874	4.970347
H	2.032399	1.793658	4.855440
H	1.093188	0.390502	4.333884
H	0.936574	-2.750938	-3.631975
H	-0.415791	-3.720152	-4.239983
H	1.213113	-4.712422	-2.266927
H	-0.525158	-5.003020	-2.213822
C	5.765516	0.778024	-0.283715
C	3.649686	1.868649	-0.162586
F	3.184633	-0.404221	-2.591128
F	5.021949	-1.409723	-2.085204
F	3.138290	-2.412357	-1.816497
F	5.423284	-2.018129	0.560663
F	3.419522	-2.714571	0.887326
F	4.153765	-1.021025	1.993015
C	6.397348	2.007251	-0.248606
H	6.367547	-0.116675	-0.353327
C	4.279036	3.098740	-0.133851
H	2.571796	1.800487	-0.136348
C	5.658194	3.172999	-0.172670
H	7.479613	2.051479	-0.283657
H	3.685861	4.004925	-0.079985
H	6.156944	4.134867	-0.146800

95

structure_Sr_replacing_Ca_2s.xyz

38	0.028156	0.074234	-0.092467
8	2.325967	0.614028	0.217928
6	3.589983	0.333496	0.553336
6	3.611375	-0.068988	2.053200
9	3.023269	-1.266302	2.213370
9	4.831814	-0.159539	2.611424
9	2.925809	0.801286	2.801374
6	4.467988	1.602894	0.364475
9	4.401506	2.013875	-0.910556
9	5.767634	1.415153	0.640845
9	4.051214	2.626182	1.122481
6	4.215887	-0.772193	-0.316816
6	3.657221	-0.965431	-1.568605
1	2.810966	-0.354310	-1.849845
6	5.238275	-2.694966	-2.056916
1	5.635972	-3.444543	-2.730975
6	5.800642	-2.512741	-0.808006
1	6.642315	-3.120488	-0.497303
6	5.303136	-1.549572	0.051742
1	5.777151	-1.415972	1.013681
8	0.372527	1.274008	-2.372326
6	-0.253741	3.121292	-3.720335
1	-0.082896	2.953153	-4.784367
6	1.068368	3.392149	-3.016764
1	0.912660	3.927857	-2.079107
1	1.768789	3.962858	-3.624398
6	1.553439	1.991855	-2.730628
1	2.249201	1.910546	-1.899520
1	1.998725	1.539155	-3.623791
8	-0.405118	-0.724974	2.335145
8	-0.424659	2.444670	0.842740
8	0.544014	-2.419136	-0.592728
8	-2.295925	-0.015344	-0.508023
6	4.166777	-1.909469	-2.438186
6	-0.754280	1.844793	-3.053818
1	-0.967977	3.937726	-3.623603
6	-1.383108	-1.706051	2.702998
6	-0.062734	0.036351	3.489109
6	0.632696	3.401945	1.001206
6	-1.668832	3.130542	0.716671
6	1.005045	-3.323021	0.408947
6	0.739674	-3.013476	-1.880779
6	-3.534944	-0.465484	-0.730137
1	3.721194	-2.035164	-3.418513
1	-1.140241	1.121239	-3.771725
1	-1.530723	2.026836	-2.311040
6	-1.910122	-1.300451	4.077500
1	-0.911253	-2.690574	2.722121
1	-2.154524	-1.699377	1.935221
6	-1.347247	0.100545	4.279026
1	0.309542	1.002231	3.154463
1	0.729310	-0.474253	4.045567
1	1.037837	3.323063	2.011642
1	1.425687	3.131700	0.305396
6	0.017045	4.770296	0.725526
1	-2.096801	3.298737	1.711674
1	-2.339700	2.494741	0.141902
6	-1.307376	4.436806	0.052003
6	2.150023	-4.053218	-0.248590
1	0.194154	-4.008633	0.679352
1	1.289281	-2.739219	1.281624
6	1.619796	-4.241120	-1.663201
1	-0.231269	-3.268189	-2.306336
1	1.216530	-2.267363	-2.517526
6	-4.450262	-0.390042	0.506448
6	-3.433328	-1.937855	-1.214056
6	-4.191396	0.395886	-1.844962
1	-1.529779	-1.973700	4.846310
1	-2.997591	-1.326046	4.112475
1	-2.006094	0.851670	3.841911
1	-1.185551	0.349657	5.326756
1	-0.162729	5.302819	1.660172
1	0.665580	5.391534	0.109588
1	-2.065889	5.204909	0.194110
1	-1.175073	4.280409	-1.019634
1	2.386581	-4.994231	0.245908
1	3.039223	-3.422284	-0.247640
1	1.021736	-5.150937	-1.727912
1	2.415976	-4.305999	-2.402410
6	-5.710997	-0.966264	0.578660
6	-3.981446	0.321933	1.594701
9	-3.063131	-2.729565	-0.195122
9	-4.570892	-2.452276	-1.713689
9	-2.506126	-2.079885	-2.168306
9	-5.474354	0.098255	-2.102078
9	-3.537016	0.297410	-3.010314
9	-4.160095	1.687987	-1.485904
6	-6.477940	-0.832561	1.720530
1	-6.110584	-1.523055	-0.256879
6	-4.753415	0.469637	2.731961
1	-2.991501	0.751846	1.527824
6	-6.005226	-0.110218	2.800359
1	-7.457864	-1.293458	1.761936
1	-4.375076	1.040746	3.572113
1	-6.612224	0.000429	3.691299

95
structure_Sr_replacing_Ca_2t.xyz

Sr	0.000001	-0.000019	-0.000001
O	-2.217333	-0.708949	-0.352120
C	-3.546061	-0.582583	-0.271853
C	-4.170048	-1.965905	0.064615
F	-3.628708	-2.447180	1.192010
F	-5.498081	-1.928725	0.255353
F	-3.936786	-2.875427	-0.892901
C	-4.063105	-0.123520	-1.661670
F	-3.688668	1.146929	-1.894930
F	-5.397431	-0.174086	-1.822552
F	-3.541752	-0.860520	-2.648955
C	-3.987828	0.394723	0.833782
C	-3.198473	0.429532	1.972728
H	-2.336245	-0.220427	2.027827
C	-4.592116	2.118416	2.941632
H	-4.826332	2.792229	3.757497
C	-5.386728	2.089161	1.812258
H	-6.249047	2.741290	1.737336
C	-5.094165	1.226653	0.769857
H	-5.743501	1.220975	-0.094116
O	-0.602972	2.351135	0.816687
C	0.290807	4.492176	0.846317
H	0.490223	5.330214	1.512211
C	-1.097966	4.564936	0.195351
H	-1.022456	4.791766	-0.867376
H	-1.721987	5.333587	0.649016
C	-1.696285	3.177718	0.429884
H	-2.156018	2.730635	-0.449307
H	-2.436521	3.187950	1.234137
O	0.602955	-2.351159	-0.816742
O	0.052053	1.045801	-2.356069
O	-0.052060	-1.045830	2.356085
O	2.217331	0.708921	0.352155
C	-3.497355	1.276913	3.020685
C	0.268035	3.169622	1.589044
H	1.076175	4.474592	0.090884
C	1.696267	-3.177728	-0.429904
C	-0.268077	-3.169679	-1.589035
C	-0.511332	0.443653	-3.519727
C	0.404586	2.376786	-2.706470
C	-0.404553	-2.376823	2.706489
C	0.511360	-0.443663	3.519718
C	3.546061	0.582585	0.271861
H	-2.866940	1.284973	3.902506
H	-0.149344	3.282511	2.596417
H	1.233223	2.668205	1.639248
C	1.097954	-4.564942	-0.195325
H	2.155995	-2.730610	0.449273
H	2.436508	-3.187985	-1.234152
C	-0.290846	-4.492190	-0.846233
H	0.149277	-3.282628	-2.596411
H	-1.233261	-2.668257	-1.639243
H	0.287442	-0.042530	-4.087897
H	-1.220997	-0.309722	-3.185346
C	-1.153571	1.580717	-4.319010
H	1.339879	2.370113	-3.277481
H	0.561606	2.935401	-1.786052
C	-0.762121	2.844824	-3.548894
C	0.762185	-2.844827	3.548887
H	-0.561578	-2.935441	1.786073
H	-1.339832	-2.370179	3.277522
C	1.153542	-1.580726	4.319053
H	1.221065	0.309659	3.185303
H	-0.287385	0.042590	4.087869
C	3.987827	-0.394697	-0.833793
C	4.063144	0.123515	1.661662
C	4.170014	1.965924	-0.064605
H	1.022493	-4.791764	0.867407
H	1.721953	-5.333598	-0.649012
H	-0.490309	-5.330264	-1.512068
H	-1.076181	-4.474543	-0.090768
H	-0.763323	1.600337	-5.335910
H	-2.233803	1.465768	-4.377545
H	-0.498975	3.676178	-4.200985
H	-1.578748	3.163430	-2.900693
H	1.578830	-3.163344	2.900665
H	0.499101	-3.676228	4.200943
H	2.233766	-1.465758	4.377707
H	0.763182	-1.600370	5.335910
C	5.094182	-1.226606	-0.769894
C	3.198458	-0.429506	-1.972729
F	3.688665	-1.146919	1.894943
F	5.397476	0.174031	1.822498
F	3.541852	0.860540	2.648962
F	5.498041	1.928770	-0.255384
F	3.936764	2.875428	0.892930
F	3.628634	2.447207	-1.191976
C	5.386752	-2.089090	-1.812312
H	5.743528	-1.220927	0.094072
C	3.497346	-1.276865	-3.020703
H	2.336215	0.220434	-2.027807
C	4.592125	-2.118345	-2.941677
H	6.249086	-2.741202	-1.737410
H	2.866918	-1.284926	-3.902515
H	4.826347	-2.792139	-3.757555

95
structure_Sr_replacing_Ca_2u.xyz

38	-0.008963	0.199074	-0.085006
8	-2.314526	0.483525	-0.549719
6	-3.563117	0.005541	-0.603509
6	-3.594386	-1.129793	-1.666561
9	-2.962385	-2.218279	-1.190857
9	-4.820094	-1.525914	-2.053254
9	-2.948313	-0.774934	-2.785001
6	-4.523937	1.142990	-1.048474
9	-4.416823	2.179701	-0.204542
9	-5.819518	0.791972	-1.068108
9	-4.225857	1.603310	-2.272943
6	-4.072941	-0.517148	0.752706
6	-3.455752	-0.014698	1.884990
1	-2.649828	0.694823	1.757965
6	-4.858446	-1.354212	3.293980
1	-5.163301	-1.683042	4.281409
6	-5.481641	-1.860541	2.168775
1	-6.278267	-2.589964	2.269885
6	-5.101748	-1.436126	0.907322
1	-5.619383	-1.836252	0.046416
8	-0.383612	2.160350	1.581323
6	0.574117	4.285456	1.892375
1	0.921885	4.982790	2.679777
6	-0.921774	4.482513	1.567961
1	-1.055806	4.890529	0.566850
1	-1.414538	5.166313	2.263007
6	-1.516322	3.071169	1.678432
1	-2.227734	2.824344	0.878980
1	-2.015831	2.920978	2.673170
8	0.582904	-2.033968	-1.272393
8	0.332137	1.729098	-2.147479
8	-0.498813	-1.552313	1.748891
8	2.258919	0.683490	0.411916
6	-3.848289	-0.419877	3.146968
6	0.650120	2.796536	2.329731
1	1.213480	4.448272	0.984762
6	1.448984	-3.048430	-0.755173
6	0.292405	-2.306673	-2.650780
6	-0.785823	2.138145	-2.934967
6	1.203330	2.884930	-2.029782
6	-1.267542	-2.755186	1.661529
6	-0.152162	-1.396217	3.125290
6	3.543761	0.323897	0.512881
1	-3.359492	-0.005162	4.021452
1	0.421996	2.679135	3.419494
1	1.595052	2.285072	2.073551
6	1.161863	-4.259166	-1.611372
1	1.222629	-3.173752	0.302824
1	2.488386	-2.726390	-0.857257
6	0.990676	-3.617247	-2.980501
1	0.649506	-1.473057	-3.259483
1	-0.790500	-2.379835	-2.756090
1	-0.493600	2.153893	-4.007426
1	-1.590389	1.412094	-2.762142
6	-1.092416	3.544205	-2.407473
1	1.984977	2.830235	-2.816634
1	1.693657	2.829333	-1.050690
6	0.310064	4.123014	-2.207675
6	-0.682476	-3.702040	2.721177
1	-1.188845	-3.124930	0.636049
1	-2.315038	-2.518150	1.866827
6	0.230973	-2.801019	3.571317
1	0.654834	-0.666129	3.194031
1	-1.022773	-1.021905	3.677493
6	4.021964	-0.523314	-0.679948
6	3.710874	-0.453699	1.848132
6	4.434685	1.596164	0.553735
1	0.236321	-4.741894	-1.290190
1	1.967073	-4.992241	-1.578502
1	1.968911	-3.424497	-3.422272
1	0.411520	-4.224492	-3.675317
1	-1.706024	4.149695	-3.094549
1	-1.636116	3.449264	-1.442246
1	0.622717	4.692574	-3.085051
1	0.363271	4.792277	-1.350716
1	-0.120481	-4.520008	2.262907
1	-1.478445	-4.146895	3.318215
1	1.278973	-2.987459	3.336650
1	0.094038	-2.944217	4.646804
6	5.029781	-1.475965	-0.623656
6	3.378475	-0.304072	-1.888138
9	3.131108	-1.662640	1.749466
9	4.980412	-0.659148	2.241948
9	3.104255	0.177646	2.860875
9	5.751292	1.336407	0.586923
9	4.161330	2.371734	1.613952
9	4.213355	2.339778	-0.538667
6	5.358369	-2.217906	-1.745340
1	5.567020	-1.660925	0.297051
6	3.717313	-1.030083	-3.013085
1	2.596584	0.444075	-1.932518
6	4.703122	-2.000567	-2.943090
1	6.137637	-2.970393	-1.678860
1	3.204229	-0.839537	-3.949683
1	4.964728	-2.580972	-3.820808

95
structure_3a.xyz

Ca	0.000066	1.243882	-0.000028
O	1.846578	0.040626	-0.082241
O	-1.846484	0.040644	0.082154
F	3.276235	0.186591	2.160201
F	1.857485	-1.419340	2.327359
F	3.953882	-1.844649	2.060847
F	4.624688	0.419085	-0.179328
F	3.797515	-0.569448	-1.904263
F	4.936843	-1.687893	-0.461488
F	-1.857671	-1.419393	-2.327447
F	-3.276126	0.186789	-2.160227
F	-3.954132	-1.844332	-2.060844
O	-1.490114	3.250829	0.037037
C	1.075866	-2.343160	-1.182044
C	2.169825	-2.341248	-0.338096
C	2.752096	-3.555537	-0.000588
H	3.616357	-3.593004	0.646617
C	2.236435	-4.739702	-0.490940
H	2.698158	-5.679006	-0.209431
C	1.141565	-4.729330	-1.334496
H	0.733883	-5.659618	-1.712190
C	0.563620	-3.525263	-1.680721
H	-0.305965	-3.501923	-2.324945
C	2.961456	-1.025188	1.677908
C	4.028990	-0.710084	-0.591933
C	-2.961546	-1.025048	-1.677947
F	-4.624611	0.419307	0.179316
C	-4.028962	-0.709886	0.591929
F	-4.936871	-1.687650	0.461534
F	-3.797440	-0.569236	1.904248
C	-1.076064	-2.343196	1.182071
C	-2.169953	-2.341199	0.338031
C	-2.752266	-3.555446	0.000448
H	-3.616485	-3.592848	-0.646820
C	-2.236704	-4.739652	0.490808
H	-2.698459	-5.678923	0.209240
C	-1.141892	-4.729366	1.334439
H	-0.734286	-5.659687	1.712133
C	-0.563913	-3.525339	1.680747
H	0.305623	-3.502058	2.325041
C	-1.329314	4.326993	0.957734
H	-0.374029	4.194637	1.459197
H	-1.305916	5.267337	0.397962
C	-2.538305	4.273609	1.893706
H	-3.011831	5.252367	1.962591
H	-2.251275	3.976713	2.901067
C	-3.462960	3.241358	1.242930
H	-4.508787	3.543705	1.240849
H	-3.388371	2.278480	1.745977
C	-2.895542	3.108036	-0.152736
H	-3.074630	2.138795	-0.606788
H	-3.249965	3.908938	-0.812709
O	0.106514	1.573575	-2.407039
C	-0.871910	2.102163	-3.295892
H	-1.811284	2.178550	-2.754612
C	-0.920146	1.143109	-4.485315
H	-1.824614	0.539344	-4.465379
H	-0.907308	1.695444	-5.424081
C	0.330893	0.272064	-4.314894
H	0.060819	-0.726846	-3.976493
H	0.907524	0.173337	-5.233139
C	1.111178	0.988630	-3.232921
H	1.725581	0.350381	-2.604852
O	-0.106148	1.573220	2.407008
C	-1.111037	0.988611	3.232847
H	-1.725477	0.350374	2.604806
H	-1.732133	1.786459	3.655461
C	-0.331036	0.272086	4.315056
H	-0.907798	0.173672	5.233254
C	0.920183	1.142883	4.485452
H	1.824522	0.538921	4.465608
H	0.907429	1.695314	5.424163
C	0.872205	2.101820	3.295929
H	0.558800	3.104170	3.607313
H	1.811658	2.178071	2.754771
O	1.490301	3.250885	-0.036793
C	2.895743	3.107954	0.152775
H	3.074801	2.138725	0.606866
C	3.462972	3.241115	-1.242988
H	3.388288	2.278187	-1.745923
H	4.508809	3.543429	-1.241075
C	2.538263	4.273327	-1.893760
H	2.250944	3.976222	-2.900977
H	3.011864	5.252024	-1.962994
C	1.329512	4.327035	-0.957503
H	1.306465	5.267414	-0.397769
C	2.688594	-0.977373	0.150231
C	-2.688609	-0.977275	-0.150281
H	0.374078	4.194869	-1.458732
H	1.732366	1.786286	-3.655762
H	0.608709	-1.401951	-1.431575
H	-0.608896	-1.402021	1.431696
H	-0.558463	3.104455	-3.607418
H	3.250347	3.908864	0.812641
H	-0.061149	-0.726952	3.976885

95
structure_3b.xyz

Ca	0.081234	1.218797	0.053823
O	1.845025	-0.105733	-0.046818
O	-1.852118	0.172939	-0.020951
F	3.423504	-0.206427	2.124958
F	1.798941	-1.609989	2.282032
F	3.809139	-2.303706	1.937718
F	4.571603	0.142741	-0.324940
F	3.679309	-0.878052	-1.993988
F	4.823481	-1.983990	-0.543087
F	-1.896188	-1.138885	-2.393421
F	-3.446478	0.348490	-2.254691
F	-3.949682	-1.734433	-2.166716
O	-1.267740	3.306610	0.115372
C	0.891088	-2.351866	-1.286731
C	1.978258	-2.487014	-0.444269
C	2.448247	-3.762736	-0.163581
H	3.310678	-3.908191	0.470113
C	1.818637	-4.873084	-0.693277
H	2.191033	-5.861992	-0.452256
C	0.724715	-4.726562	-1.524991
H	0.227596	-5.598686	-1.933109
C	0.267370	-3.459922	-1.826529
H	-0.594203	-3.329392	-2.468573
C	2.929017	-1.344341	1.611832
C	3.939749	-0.985162	-0.684089
C	-3.039715	-0.836166	-1.765125
F	-4.433689	0.943292	0.187258
C	-4.101241	-0.321680	0.474055
F	-5.169038	-1.077076	0.170568
F	-3.947587	-0.383530	1.804864
C	-1.252157	-2.267257	1.042143
C	-2.415837	-2.155158	0.304848
C	-3.193597	-3.290119	0.115571
H	-4.119180	-3.239601	-0.438731
C	-2.796933	-4.506736	0.637711
H	-3.413089	-5.383029	0.472914
C	-1.626009	-4.607787	1.365166
H	-1.312980	-5.564303	1.767085
C	-0.856238	-3.480872	1.569666
H	0.071096	-3.544376	2.124550
C	-1.513849	4.033165	1.314390
H	-0.914479	3.587815	2.105505
H	-1.210887	5.078488	1.181605
C	-3.006144	3.922300	1.513417
H	-3.400339	4.671066	2.199071
H	-3.255103	2.929285	1.890295
C	-3.504867	4.089643	0.086249
H	-3.567388	5.149067	-0.167940
H	-4.482164	3.641470	-0.079885
C	-2.421502	3.401201	-0.734094
H	-2.701291	2.386153	-1.009139
H	-2.153704	3.965038	-1.630201
O	0.364258	1.716363	-2.305274
C	-0.532589	1.809316	-3.407172
H	-1.526783	1.551413	-3.055121
C	0.016725	0.857135	-4.466301
H	-0.556713	-0.067152	-4.491854
H	-0.033967	1.308877	-5.456071
C	1.461700	0.596303	-4.019069
H	1.575980	-0.422927	-3.653971
H	2.187728	0.751064	-4.815600
C	1.662574	1.571093	-2.875674
H	2.326499	1.208894	-2.095781
O	-0.060310	1.301053	2.470944
C	-1.167988	0.826976	3.232740
H	-1.848319	0.328426	2.549698
H	-1.676095	1.681692	3.695939
C	-0.533634	-0.067703	4.273856
H	-1.161522	-0.182833	5.155826
C	0.794046	0.635713	4.571852
H	1.617480	-0.073481	4.622101
H	0.758792	1.174673	5.518012
C	0.969941	1.610908	3.405922
H	0.849584	2.649329	3.732101
H	1.922635	1.510022	2.894730
O	1.687621	3.100965	0.251718
C	3.039704	2.931353	0.674972
H	3.209920	1.866004	0.801648
C	3.856721	3.562870	-0.425367
H	3.934008	2.873501	-1.266217
H	4.863603	3.823678	-0.103318
C	3.009911	4.776939	-0.782635
H	3.135907	5.092746	-1.817096
H	3.272259	5.620348	-0.142834
C	1.582253	4.316419	-0.496623
H	1.022914	5.049822	0.086553
C	2.614361	-1.194909	0.098681
C	-2.788412	-0.761404	-0.233903
H	1.016282	4.094948	-1.400708
H	2.002373	2.549873	-3.235344
H	0.521204	-1.360138	-1.502979
H	-0.636485	-1.391908	1.186949
H	-0.536753	2.843846	-3.767384
H	3.190381	3.451071	1.628325
H	-0.360755	-1.055188	3.847869

95
structure_3c.xyz

Ca	0.061576	1.215097	0.068541
O	-1.780006	0.025667	-0.062192
O	1.889780	-0.024963	0.140611
F	-3.474248	0.207390	-2.245253
F	-1.921072	-1.275605	-2.424940
F	-3.961056	-1.879228	-2.127325
F	-4.273890	0.878848	0.307416
F	-3.865870	-0.580432	1.830686
F	-5.138184	-1.084955	0.165920
F	1.662082	-1.673804	2.342210
F	3.277503	-0.253732	2.436844
F	3.703581	-2.331661	2.144028
O	1.625331	3.119673	0.237767
C	-1.150536	-2.457963	0.880409
C	-2.380714	-2.294329	0.275141
C	-3.230136	-3.389546	0.182837
H	-4.206851	-3.295212	-0.269343
C	-2.838600	-4.621019	0.671884
H	-3.511950	-5.466170	0.587966
C	-1.598495	-4.776667	1.263741
H	-1.290842	-5.745392	1.640037
C	-0.756200	-3.688591	1.369783
H	0.221523	-3.793516	1.822229
C	-3.044552	-0.973394	-1.762460
C	-4.021640	-0.422121	0.508504
C	2.847404	-1.356421	1.803007
F	4.632981	0.298112	0.170036
C	4.067661	-0.803859	-0.343079
F	4.956602	-1.797321	-0.195610
F	3.938059	-0.590777	-1.659901
C	1.126802	-2.191646	-1.348826
C	2.137257	-2.363108	-0.420446
C	2.625926	-3.643591	-0.199494
H	3.431722	-3.815249	0.498742
C	2.089280	-4.724882	-0.872527
H	2.474484	-5.718700	-0.676255
C	1.071877	-4.543086	-1.789540
H	0.647808	-5.392773	-2.311437
C	0.597304	-3.269646	-2.031033
H	-0.205925	-3.113593	-2.739517
C	1.600677	4.334341	-0.504207
H	0.947169	4.180872	-1.360970
H	1.175887	5.125737	0.119574
C	3.054043	4.644755	-0.875507
H	3.333110	5.635833	-0.519026
H	3.209279	4.628507	-1.953021
C	3.860960	3.553192	-0.170412
H	4.787620	3.923458	0.265186
H	4.110328	2.746300	-0.858076
C	2.895709	3.039957	0.874710
H	3.057672	2.002772	1.155283
H	2.893306	3.676092	1.767935
O	-0.280728	1.628899	2.438345
C	0.649718	1.524686	3.514662
H	1.612202	1.247389	3.095454
C	0.071874	0.481275	4.475287
H	0.687212	-0.414996	4.506984
H	0.015642	0.884313	5.485997
C	-1.321364	0.188235	3.910244
H	-1.318438	-0.727163	3.318823
H	-2.083833	0.092765	4.681450
C	-1.565068	1.367827	2.997858
H	-2.248736	1.170943	2.177979
O	0.430719	1.600002	-2.302537
C	1.771190	1.588040	-2.790767
H	2.418026	1.314008	-1.962359
H	2.023261	2.594353	-3.142652
C	1.774637	0.583546	-3.936096
H	2.400987	0.929469	-4.757060
C	0.296471	0.466345	-4.334224
H	-0.081808	-0.530539	-4.115424
H	0.125210	0.665292	-5.391031
C	-0.397412	1.491675	-3.454102
H	-0.447521	2.470300	-3.945379
H	-1.392916	1.201233	-3.131864
O	-1.358263	3.241325	-0.091395
C	-2.434559	3.268832	-1.036984
H	-2.719225	2.236302	-1.223200
C	-3.556494	4.083296	-0.396327
H	-4.518689	3.583203	-0.485882
H	-3.639041	5.061876	-0.870727
C	-3.104444	4.233835	1.050582
H	-3.418897	3.373965	1.642572
H	-3.480138	5.139887	1.523962
C	-1.601526	4.230237	0.900067
H	-1.241229	5.203961	0.547755
C	-2.741556	-0.890202	-0.241483
C	2.676123	-1.102577	0.280600
H	-1.049071	3.948514	1.794069
H	-1.897345	2.249656	3.559409
H	-0.488513	-1.608731	0.952409
H	0.740368	-1.197410	-1.522548
H	0.739095	2.503869	3.995161
H	-2.078670	3.721357	-1.965805
H	2.163434	-0.375611	-3.600789

95
structure_3d.xyz

Ca	0.055021	1.216121	0.065884
O	1.885223	-0.020177	0.140670
O	-1.785608	0.023835	-0.063530
F	3.284963	-0.235338	2.430309
F	1.677822	-1.665314	2.347750
F	3.722286	-2.311305	2.140428
F	4.629297	0.314075	0.156874
F	3.926980	-0.577906	-1.668754
F	4.957836	-1.780301	-0.209732
F	-1.929194	-1.286301	-2.425199
F	-3.479131	0.199632	-2.240381
F	-3.969714	-1.885592	-2.121602
O	-1.369852	3.239855	-0.088178
C	1.125778	-2.195372	-1.339446
C	2.141637	-2.358584	-0.415502
C	2.638834	-3.635619	-0.193762
H	3.448339	-3.800934	0.501675
C	2.106568	-4.721565	-0.862670
H	2.498895	-5.712527	-0.666053
C	1.084802	-4.547935	-1.776378
H	0.664382	-5.401187	-2.295409
C	0.600657	-3.278018	-2.017748
H	-0.206651	-3.128179	-2.722991
C	2.858339	-1.342154	1.801426
C	4.064620	-0.789944	-0.352593
C	-3.050010	-0.981821	-1.759145
F	-4.289608	0.855922	0.310786
C	-4.021292	-0.441007	0.516920
F	-5.132380	-1.118617	0.186126
F	-3.854283	-0.590732	1.838743
C	-1.142797	-2.450418	0.893427
C	-2.368583	-2.300216	0.275586
C	-3.203422	-3.405414	0.171044
H	-4.176556	-3.321524	-0.290635
C	-2.802208	-4.633512	0.660804
H	-3.463982	-5.486699	0.566821
C	-1.567321	-4.775275	1.266614
H	-1.251991	-5.741099	1.644004
C	-0.739723	-3.677235	1.385259
H	0.234154	-3.771931	1.848062
C	-1.605963	4.227762	0.905901
H	-1.042340	3.948297	1.793647
H	-1.253764	5.203049	0.549573
C	-3.107062	4.224875	1.073398
H	-3.481302	5.128979	1.551623
H	-3.411008	3.363287	1.668412
C	-3.574671	4.073189	-0.368459
H	-3.666909	5.051704	-0.841210
H	-4.535585	3.568865	-0.447431
C	-2.456419	3.263892	-1.022216
H	-2.739047	2.230539	-1.206455
H	-2.112508	3.718934	-1.954298
O	0.413655	1.595926	-2.307862
C	-0.420916	1.485450	-3.454558
H	-1.413676	1.192532	-3.126401
C	0.270287	0.462176	-4.340037
H	-0.110461	-0.534958	-4.126993
H	0.099237	0.667603	-5.395658
C	1.749203	0.573464	-3.942270
H	2.131754	-0.385229	-3.598384
H	2.378536	0.908660	-4.765377
C	1.750647	1.586211	-2.804852
H	2.404640	1.321336	-1.979191
O	-0.276038	1.625694	2.438366
C	-1.553066	1.358148	3.010959
H	-2.241195	1.144607	2.199016
H	-1.890061	2.243368	3.564247
C	-1.290475	0.192544	3.936431
H	-2.043903	0.102502	4.717137
C	0.106882	0.502513	4.482683
H	0.730973	-0.387767	4.511621
H	0.059773	0.910889	5.491693
C	0.663095	1.545195	3.508838
H	0.740952	2.529973	3.980049
H	1.626613	1.278400	3.085240
O	1.615618	3.123461	0.219084
C	2.889455	3.050999	0.849845
H	3.056272	2.015799	1.134864
C	3.848322	3.562521	-0.202437
H	4.103198	2.751505	-0.883092
H	4.772772	3.943862	0.228280
C	3.032115	4.641529	-0.917135
H	3.173914	4.604051	-1.995954
H	3.314992	5.639560	-0.583947
C	1.583641	4.337761	-0.522577
H	1.173246	5.131006	0.108694
C	2.677592	-1.093406	0.279205
C	-2.741439	-0.898708	-0.239379
H	0.915240	4.187182	-1.368138
H	1.995900	2.590872	-3.166399
H	0.732410	-1.203924	-1.513539
H	-0.491239	-1.593487	0.972743
H	-0.476541	2.464158	-3.945059
H	2.888970	3.691723	1.739778
H	-1.287354	-0.731140	3.358140

95
structure_Ca_replacing_Sr_3a.xyz

Sr	0.001037	1.241204	0.009803
O	-1.965915	-0.024274	0.078317
O	1.964154	-0.028730	-0.089970
F	-3.257661	0.142432	-2.236797
F	-1.829465	-1.460624	-2.337044
F	-3.938217	-1.892099	-2.198186
F	-4.725532	0.351637	0.051850
F	-3.985241	-0.672397	1.796237
F	-5.062662	-1.759628	0.281819
F	1.824001	-1.457249	2.328093
F	3.256806	0.141687	2.225330
F	3.931641	-1.894865	2.192936
O	1.570446	3.363206	0.025748
C	-1.193045	-2.414411	1.144553
C	-2.271337	-2.410777	0.280276
C	-2.833907	-3.625970	-0.085620
H	-3.685635	-3.662110	-0.749806
C	-2.312784	-4.811601	0.396469
H	-2.759020	-5.751788	0.093659
C	-1.231284	-4.802323	1.257417
H	-0.818982	-5.733971	1.626570
C	-0.673309	-3.597324	1.632825
H	0.185519	-3.573448	2.291814
C	-2.972424	-1.074712	-1.749300
C	-4.157014	-0.788654	0.472146
C	2.968832	-1.076056	1.740519
F	4.721803	0.347739	-0.067070
C	4.155003	-0.795694	-0.481038
F	5.061278	-1.764727	-0.283441
F	3.984894	-0.687747	-1.805973
C	1.191073	-2.418862	-1.153378
C	2.267962	-2.415520	-0.287252
C	2.828581	-3.630716	0.081388
H	3.679576	-3.666728	0.746518
C	2.306796	-4.816460	-0.399852
H	2.751474	-5.756754	-0.095071
C	1.226647	-4.807071	-1.262471
H	0.813723	-5.738753	-1.630857
C	0.670906	-3.601901	-1.640799
H	-0.186612	-3.577866	-2.301543
C	1.437371	4.397607	-0.945288
H	0.456067	4.297861	-1.404559
H	1.491188	5.366280	-0.438026
C	2.600659	4.217282	-1.922192
H	3.120304	5.161949	-2.078386
H	2.252049	3.867936	-2.892489
C	3.498942	3.179283	-1.242024
H	4.556265	3.434932	-1.284798
H	3.369723	2.195329	-1.690425
C	2.971211	3.141768	0.175786
H	3.110965	2.180832	0.663913
H	3.396872	3.943437	0.790524
O	-0.268136	1.573498	2.559952
C	0.687577	2.046360	3.503247
H	1.631769	2.193207	2.982118
C	0.748476	0.988529	4.605338
H	1.665596	0.407182	4.540464
H	0.717985	1.456145	5.588586
C	-0.482496	0.109325	4.348727
H	-0.187129	-0.853327	3.934453
H	-1.066255	-0.076165	5.248803
C	-1.267504	0.895583	3.318801
H	-1.857318	0.295013	2.630212
O	0.286406	1.584066	-2.540236
C	1.280012	0.909046	-3.308527
H	1.874056	0.306473	-2.625521
H	1.925298	1.652902	-3.790117
C	0.487184	0.125707	-4.334088
H	1.065941	-0.065130	-5.236296
C	-0.739052	1.011742	-4.587006
H	-1.658806	0.433097	-4.537224
H	-0.698967	1.492055	-5.563823
C	-0.682001	2.055880	-3.471087
H	-0.358608	3.030000	-3.853144
H	-1.623642	2.182431	-2.940125
O	-1.571601	3.367378	-0.032699
C	-2.972112	3.143846	-0.182203
H	-3.110221	2.186637	-0.677808
C	-3.497234	3.169692	1.236947
H	-3.364250	2.182851	1.677936
H	-4.555169	3.422028	1.283836
C	-2.600026	4.205026	1.922859
H	-2.245346	3.847425	2.887954
H	-3.122310	5.146193	2.090787
C	-1.442155	4.398785	0.942087
H	-1.506139	5.369050	0.439100
C	-2.785281	-1.044427	-0.208570
C	2.782768	-1.048849	0.199558
H	-0.458044	4.305678	1.396953
H	-1.916337	1.638283	3.797346
H	-0.749140	-1.470199	1.423045
H	0.749454	-1.474187	-1.434102
H	0.347381	3.010817	3.895211
H	-3.400275	3.949460	-0.790049
H	0.187740	-0.834209	-3.916333

95

structure_Ca_replacing_Sr_3b.xyz

38	-0.084777	-1.236940	0.032096
8	1.976864	-0.133958	-0.004381
8	-1.932453	0.198492	-0.100288
9	3.401944	-0.306627	-2.307719
9	1.916951	1.249177	-2.369306
9	4.006508	1.750575	-2.227111
9	4.604557	-0.827501	0.070241
9	4.076313	0.405851	1.750729
9	5.255688	1.220728	0.141547
9	-1.781745	1.631349	2.271515
9	-3.409924	0.231408	2.133667
9	-3.804740	2.335017	2.028123
8	-1.788577	-3.212033	0.303231
6	1.348993	2.300085	1.052665
6	2.492589	2.208932	0.281580
6	3.226937	3.363745	0.045598
1	4.133801	3.330799	-0.540509
6	2.809304	4.576866	0.559688
1	3.391494	5.468800	0.359072
6	1.659625	4.655642	1.323180
1	1.329413	5.609604	1.717193
6	0.931130	3.510189	1.571164
1	0.018427	3.556337	2.151857
6	3.073938	0.890183	-1.793138
6	4.222577	0.409172	0.417665
6	-2.939040	1.386597	1.637777
9	-4.665947	-0.014493	-0.281453
6	-4.032105	1.113851	-0.636770
9	-4.901603	2.118602	-0.450852
9	-3.808738	1.030387	-1.955216
6	-0.947883	2.480772	-1.234317
6	-2.043268	2.593497	-0.399075
6	-2.511996	3.861974	-0.085117
1	-3.378704	3.990266	0.546939
6	-1.875779	4.985243	-0.578826
1	-2.248560	5.967940	-0.314005
6	-0.774173	4.860037	-1.404223
1	-0.271662	5.742414	-1.782600
6	-0.315171	3.601525	-1.735923
1	0.553555	3.486211	-2.371643
6	-1.738918	-4.399375	-0.497166
1	-1.283254	-4.138997	-1.454377
1	-1.103131	-5.134606	-0.002694
6	-3.177694	-4.867386	-0.652521
1	-3.418315	-5.617649	0.102559
1	-3.360994	-5.304808	-1.632705
6	-3.974572	-3.597254	-0.398418
1	-4.996866	-3.786433	-0.075193
1	-4.006433	-2.969899	-1.290486
6	-3.142590	-2.929916	0.670041
1	-3.255825	-1.848698	0.711533
1	-3.345705	-3.361033	1.656343
8	0.139666	-1.325829	2.599519
6	-0.880494	-1.560816	3.566609
1	-1.840718	-1.479444	3.062718
6	-0.675363	-0.515419	4.665590
1	-1.491943	0.203121	4.687195
1	-0.625710	-0.994411	5.642839
6	0.651951	0.156205	4.299648
1	0.477997	1.114830	3.812359
1	1.294792	0.325747	5.161817
6	1.263309	-0.810645	3.309797
1	1.935757	-0.361098	2.583551
8	-0.466656	-1.735339	-2.475429
6	-1.766263	-1.474544	-3.001243
1	-2.399227	-1.147777	-2.178842
1	-2.163550	-2.398842	-3.436989
6	-1.542698	-0.408399	-4.057656
1	-2.273871	-0.478461	-4.861367
6	-0.105017	-0.661007	-4.534076
1	0.497017	0.241273	-4.448094
1	-0.064266	-0.991461	-5.571012
6	0.411074	-1.748375	-3.596109
1	0.364255	-2.735986	-4.067383
1	1.422364	-1.575531	-3.235591
8	1.347995	-3.454363	0.027437
6	2.529387	-3.418477	-0.791517
1	2.733219	-2.373525	-1.026162
6	3.644469	-4.031131	0.041209
1	4.596958	-3.535044	-0.132385
1	3.761893	-5.091482	-0.189572
6	3.128027	-3.858188	1.460662
1	3.295819	-2.835612	1.803413
1	3.578173	-4.548530	2.172606
6	1.650055	-4.098099	1.263031
1	1.429576	-5.168595	1.182342
6	2.884139	0.818616	-0.253975
6	-2.682799	1.288582	0.109751
1	1.016271	-3.663362	2.034848
1	1.774911	-1.636063	3.819606
1	0.768572	1.407533	1.233807
1	-0.582113	1.494579	-1.479137
1	-0.770245	-2.577661	3.956775
1	2.334518	-3.971476	-1.712231
1	-1.634318	0.578627	-3.607763

Optimized geometry for 4a based on crystal geometry of 4.

125

Sr_Oct2_sad_part2__lcwpbe_GD3BJ_internal_Def2TZVP_singlet_optfreq_16core_ultrafine_benzene.xyz

C	8.543147	10.912962	15.329152
C	8.676541	12.080009	16.343217
C	7.148233	10.969056	14.648417
C	8.708919	9.616509	16.129098
C	9.974150	9.059256	16.181798
C	10.221148	7.928036	16.934299
C	7.688033	9.020165	16.854019
C	8.300183	16.663535	13.215184
C	6.777040	16.630344	13.503443
C	9.077261	17.207003	14.442791
C	8.553835	17.592143	12.015555
C	9.119514	17.028848	10.887074
C	9.381500	17.794355	9.766951
C	9.078069	19.142350	9.763167
C	8.510232	19.713641	10.886705
C	8.248236	18.945840	12.006126
C	8.848664	11.489984	10.041282
C	10.020617	12.196871	9.315023
C	7.586001	12.392004	9.956551
C	8.484011	10.151413	9.395917
C	7.759391	9.271579	10.187386
C	7.340526	8.054190	9.690841
C	7.659880	7.687462	8.395655
C	8.388077	8.553881	7.604954
C	8.790076	9.784055	8.096208
C	11.691075	7.338396	8.680463
C	11.039930	7.586507	10.021975
C	10.711804	6.555054	13.729908
C	9.685996	5.586817	14.314446
C	8.364017	6.320917	14.139852
C	8.634851	7.148490	12.909412
C	9.196408	7.328625	17.641343
C	7.929855	7.878676	17.596324
C	12.878330	13.080324	13.172213
C	12.925439	14.317837	12.235460
C	12.623271	13.546961	14.632149
C	13.034807	8.816025	9.909433
C	13.150704	7.624456	8.985662
C	13.829138	7.929158	13.264262
C	13.679717	9.464119	14.909064
C	14.235654	12.382499	13.050094
C	14.327100	7.184239	14.493168
C	14.239349	8.234685	15.609902
C	14.384592	11.477446	12.012150
C	15.318816	12.631425	13.878293
C	15.585128	10.826537	11.809201
C	16.515034	11.962708	13.689745
C	16.653551	11.057762	12.656245
F	8.543329	13.269101	15.720545
F	9.889986	12.063529	16.893809
F	7.077371	12.009512	13.795603
F	6.104350	11.093392	15.471423
F	6.951403	9.863815	13.918048
F	6.476856	15.791886	14.502930
F	6.127061	16.192729	12.417246
F	6.238934	17.819791	13.826860
F	10.380237	17.290097	14.144545
F	8.969553	16.391990	15.498904
F	8.693676	18.426660	14.861035
F	11.088399	11.383423	9.284832
F	9.776881	12.578128	8.056473
F	10.394229	13.297983	9.981959
F	7.173340	12.616941	8.708092
F	7.812731	13.589690	10.530397
F	6.571639	11.840844	10.624077
F	11.755299	14.982690	12.265806
F	11.356848	13.991106	14.771859
F	7.783194	12.052176	17.333741
F	13.118115	13.929713	10.973529
F	13.889518	15.189964	12.536110
F	12.760023	12.519003	15.474234
F	13.408124	14.534163	15.069418
O	9.471817	11.090402	14.363960
O	8.701461	15.404426	13.008466
O	9.205904	11.362402	11.341813
O	11.831094	12.306536	12.800954
O	11.821782	8.617811	10.644139
O	9.977935	7.597360	13.080670
O	12.983572	8.955446	13.770065
Sr	10.860882	10.056094	12.636378
Sr	9.457804	13.262939	12.960197
H	10.776542	9.546956	15.646547
H	11.222248	7.515961	16.976897
H	6.692228	9.439543	16.856514
H	7.802843	19.415222	12.871598
H	8.266827	20.769803	10.895574
H	9.282372	19.747386	8.887332
H	9.825483	17.332221	8.892725
H	9.348512	15.973188	10.905927
H	9.342182	10.446971	7.445320
H	8.642419	8.278152	6.588447
H	7.335881	6.731355	8.002194
H	6.757649	7.389381	10.316978
H	7.514763	9.569616	11.197873
H	11.509169	6.329165	8.315083
H	11.310548	8.046365	7.943404
H	10.009655	7.927360	9.946650
H	11.074597	6.695965	10.655329
H	11.359533	6.063712	12.999168

H	11.333594	7.022826	14.489958
H	9.897573	5.355317	15.356539
H	9.682519	4.651663	13.753766
H	8.171804	6.978838	14.986937
H	7.515401	5.649867	14.017395
H	7.996116	8.021054	12.812792
H	8.566478	6.549590	11.994674
H	9.385297	6.440668	18.232815
H	7.118589	7.422680	18.150871
H	12.960972	9.749395	9.346973
H	13.854511	8.895184	10.620613
H	13.745838	7.846015	8.101520
H	13.606806	6.779922	9.506685
H	13.252907	7.324696	12.567582
H	14.663742	8.395266	12.731469
H	14.478249	10.125068	14.563819
H	12.982428	10.044114	15.507442
H	15.339715	6.812640	14.345502
H	13.689548	6.328457	14.712502
H	13.573871	7.903252	16.405375
H	15.207318	8.448662	16.059413
H	13.548397	11.311532	11.345544
H	15.249174	13.345639	14.685843
H	15.697361	10.143696	10.975245
H	17.346520	12.161379	14.355180
H	17.594998	10.544218	12.501610

Optimized geometry for 4b based on calculated minima.

125
Sr_oct2_sad_pi_A__lcwpbe_GD3BJ_internal_Def2TZVP_singlet_optfreq_16core_ultrafine_benzene_FREQ.xyz

Sr	2.073843	0.337597	0.551393
Sr	-1.197583	-0.315972	-0.452563
O	1.061906	-1.504967	-0.723534
C	1.149924	-2.631329	-1.469245
C	0.227080	-3.703602	-0.832669
9	-1.040754	-3.249985	-0.761611
9	0.622697	-3.959743	0.413738
C	0.637723	-2.299631	-2.899508
9	-0.481537	-1.547536	-2.836718
9	0.329127	-3.355538	-3.656250
9	1.542194	-1.579759	-3.566680
C	2.555376	-3.236146	-1.531501
C	3.400444	-2.989065	-0.466119
C	4.671502	-3.526242	-0.422456
C	3.004824	-4.050107	-2.560449
H	3.043377	-2.380255	0.349315
H	5.311557	-3.326275	0.428791
H	2.368456	-4.283987	-3.402104
O	-3.443589	-0.632004	-0.626716
C	-4.675127	-1.132499	-0.791037
C	-4.770649	-1.681586	-2.238086
9	-3.796527	-2.563025	-2.500670
9	-4.629354	-0.672921	-3.107933
9	-5.930841	-2.291564	-2.534013
C	-4.862056	-2.278137	0.238537
9	-3.858130	-3.161098	0.185835
9	-5.998298	-2.984831	0.095181
9	-4.873015	-1.771202	1.476755
C	-5.790244	-0.093968	-0.588577
C	-5.400896	1.206478	-0.332456
C	-6.339619	2.201473	-0.136847
C	-7.687407	1.903929	-0.194004
C	-8.085512	0.604990	-0.451964
C	-7.145238	-0.388573	-0.650293
H	-7.481060	-1.395378	-0.853787
H	-9.139967	0.359207	-0.501564
H	-8.427500	2.680970	-0.040816
H	-6.013862	3.216269	0.062378
H	-4.343185	1.417440	-0.283454
O	0.527863	1.579557	-0.917736
C	0.177112	2.650105	-1.664582
C	-1.086675	3.284108	-1.021579
9	-0.764648	3.925742	0.105853
9	-1.760526	4.139727	-1.794214
9	-1.973061	2.325661	-0.675470
C	-0.174038	2.150701	-3.091144
9	-0.518749	3.117533	-3.943684
9	-1.196713	1.272375	-3.052669
9	0.871971	1.509122	-3.614252
C	1.275336	3.707891	-1.820275
C	2.586603	3.285287	-1.717656
C	3.636945	4.166171	-1.882550
C	3.386085	5.500778	-2.135060
C	2.078320	5.934933	-2.239714
C	1.029975	5.044784	-2.097828
H	0.020615	5.414782	-2.205643
H	1.866348	6.977799	-2.442369
H	4.205025	6.199641	-2.256995
H	4.657291	3.806492	-1.814243
H	2.784306	2.244918	-1.519447
O	-0.049540	0.246914	1.734259
9	-0.155855	0.061717	5.284043
9	-0.960192	-2.217306	4.120746
O	2.982265	2.550664	1.572967
C	3.899804	4.740639	1.619739
H	4.231821	5.100905	2.593826
H	4.387522	5.339861	0.853328
C	4.221511	3.256028	1.468769
H	4.654045	3.013934	0.497775
H	4.898850	2.911052	2.253018
O	4.212612	0.329544	-1.012963
C	5.548479	-0.064418	-0.676080
H	6.074488	0.790087	-0.244116
H	5.478160	-0.852662	0.072964
C	6.198751	-0.552666	-1.963068
H	6.811201	-1.436809	-1.794385
H	6.834255	0.225116	-2.388636
C	5.010117	-0.824102	-2.872557
H	4.591142	-1.810663	-2.681469
H	5.256892	-0.748853	-3.930170
C	4.042592	0.244834	-2.434816
H	2.997945	0.017461	-2.633434
H	4.289115	1.211099	-2.885971
O	3.824695	-0.884596	2.194387
9	0.196549	-4.864862	-1.488470
C	5.121628	-4.319310	-1.460440
C	4.282593	-4.578097	-2.527744
C	-0.612161	-0.106980	2.918548
C	0.230671	0.480660	4.075516
C	-0.576779	-1.653488	2.971858
C	1.983669	3.448004	2.071265
C	2.381289	4.793895	1.518725
C	4.849857	-0.097575	2.790298
C	3.714677	-2.045194	3.023836
H	6.119630	-4.740316	-1.436066
H	4.620739	-5.202459	-3.345879
C	-2.039040	0.433062	3.070818
9	0.157250	1.814608	4.074700
9	1.535723	0.173356	3.947524

9	-1.375662	-2.133105	2.003255
9	0.657543	-2.109382	2.721325
H	1.013702	3.088947	1.731798
H	2.003862	3.437920	3.164248
H	2.064689	4.880643	0.480609
H	1.947883	5.616861	2.084566
H	5.172289	0.646264	2.065645
H	4.446119	0.414862	3.670075
C	5.928193	-1.094950	3.166381
C	5.138649	-2.375906	3.462525
H	3.080513	-1.806468	3.879310
H	3.234864	-2.832154	2.447125
C	-2.310281	1.630234	2.426204
C	-3.043999	-0.176155	3.803277
H	6.517842	-0.753565	4.015400
H	6.607324	-1.244612	2.326861
H	5.538844	-3.223396	2.908876
H	5.162251	-2.635878	4.519375
H	-1.516809	2.110543	1.871051
C	-3.565741	2.197558	2.487486
C	-4.304835	0.389915	3.857692
H	-2.869614	-1.104134	4.327739
H	-3.760690	3.126874	1.965609
C	-4.572717	1.571922	3.197994
H	-5.088838	-0.112536	4.411035
H	-5.567665	1.999066	3.226184

Optimized geometry for 4c based on calculated minima.

125
Sr_oct2_sad_pi_B__lcwpbe_GD3BJ_internal_Def2TZVP_singlet_optfreq_16core_ultrafine_benzene_FREQ.xyz

Sr	-2.052658	-0.016326	0.013400
Sr	1.441778	0.192973	0.210325
O	-0.478316	1.873063	-0.107727
C	-0.177499	3.154792	-0.420814
C	0.691422	3.745860	0.724251
9	1.780605	2.977331	0.937476
9	-0.000628	3.761980	1.862257
C	0.652277	3.142136	-1.736585
9	1.595482	2.178926	-1.687550
9	1.296343	4.275286	-2.023212
9	-0.130673	2.853618	-2.780016
C	-1.399902	4.068045	-0.548590
C	-1.450412	5.190145	-1.360700
C	-2.577338	5.992409	-1.379480
C	-2.487893	3.783216	0.259546
H	-0.613619	5.458899	-1.989447
H	-2.601773	6.860080	-2.027636
H	-2.429288	2.935878	0.929751
O	3.716630	0.117340	0.176584
C	5.029587	0.376097	0.209890
C	5.287965	1.625805	-0.672084
9	4.537970	2.667371	-0.289890
9	4.959132	1.351064	-1.940859
9	6.561682	2.055408	-0.684461
C	5.414464	0.662177	1.684873
9	5.287776	-0.458088	2.406881
9	4.609222	1.577608	2.238400
9	6.672807	1.102677	1.864632
C	5.895937	-0.780125	-0.315984
C	5.236955	-1.899117	-0.789443
C	5.941624	-2.980606	-1.282991
C	7.322947	-2.954910	-1.306749
C	7.989715	-1.839929	-0.833858
C	7.283295	-0.758145	-0.342082
H	7.827006	0.102497	0.020606
H	9.073049	-1.808142	-0.846882
H	7.880144	-3.800859	-1.692511
H	5.405411	-3.847855	-1.651036
H	4.157261	-1.901388	-0.761564
O	-0.284947	-1.004638	-1.333964
C	0.154489	-1.936664	-2.208832
C	0.843008	-3.062045	-1.389022
9	-0.075150	-3.811378	-0.766618
9	1.625124	-3.888061	-2.089609
9	1.623960	-2.538018	-0.424023
C	1.197840	-1.261852	-3.139946
9	1.721732	-2.072875	-4.059994
9	2.221240	-0.761002	-2.419382
9	0.635123	-0.236023	-3.779616
C	-0.929912	-2.514226	-3.124642
C	-2.008438	-1.700664	-3.418328
C	-2.983159	-2.103677	-4.309445
C	-2.898752	-3.344129	-4.912190
C	-1.830543	-4.169665	-4.617011
C	-0.847029	-3.755791	-3.737132
H	-0.011404	-4.414396	-3.546979
H	-1.751865	-5.144608	-5.082712
H	-3.660643	-3.666329	-5.611907
H	-3.811201	-1.443590	-4.540853
H	-2.058151	-0.727051	-2.955750
O	-0.354708	-0.699294	1.706924
9	2.057353	-0.550880	3.015135
9	0.197987	1.353261	3.302894
O	-3.691547	-1.968396	-0.596896
C	-5.281076	-3.269558	-1.787707
H	-6.130075	-3.637141	-1.210417
H	-5.551293	-3.302313	-2.841816
C	-4.905501	-1.858220	-1.344610
H	-4.710673	-1.191084	-2.182297
H	-5.674277	-1.409111	-0.710681
O	-3.658799	1.127465	-1.744727
C	-4.932007	1.723521	-1.463044
H	-5.670095	0.934786	-1.315214
H	-4.835637	2.295860	-0.542137
C	-5.250644	2.630756	-2.637442
H	-5.796611	3.519049	-2.323865
H	-5.851827	2.103267	-3.380098
C	-3.871283	2.941584	-3.193472
H	-3.381862	3.713998	-2.601071
H	-3.885425	3.254660	-4.235967
C	-3.171768	1.621088	-2.999228
H	-2.089298	1.693110	-2.935354
H	-3.439683	0.915008	-3.791589
O	-4.134520	0.241956	1.631630
9	1.121200	4.988314	0.501917
C	-3.660130	5.696773	-0.574786
C	-3.607648	4.590813	0.253067
C	-0.296723	-0.959706	3.035912
C	1.131060	-1.456577	3.384860
C	-0.565740	0.371510	3.780390
C	-3.460924	-3.344010	-0.301086
C	-4.033228	-4.085952	-1.481423
C	-4.525666	-0.957036	2.320297
C	-4.698551	1.383594	2.289342
H	-4.537503	6.332336	-0.583963
H	-4.439885	4.365605	0.909818
C	-1.270167	-2.061175	3.461128
9	1.407401	-2.575689	2.711032

9	1.311715	-1.710025	4.680972
9	-1.844914	0.740895	3.579949
9	-0.369541	0.339844	5.105149
H	-2.391428	-3.483931	-0.168974
H	-3.975008	-3.605420	0.629485
H	-3.330653	-4.050415	-2.312696
H	-4.251846	-5.127951	-1.253076
H	-5.292300	-1.464710	1.728715
H	-3.657965	-1.609673	2.411925
C	-5.065783	-0.497706	3.653983
C	-5.693115	0.836444	3.291297
H	-3.897204	1.936995	2.783535
H	-5.161370	2.023867	1.539393
C	-1.450420	-3.096291	2.556567
C	-1.947827	-2.101694	4.668717
H	-4.246974	-0.357161	4.359550
H	-5.769708	-1.210722	4.079504
H	-6.666158	0.686227	2.819727
H	-5.823146	1.501593	4.143108
H	-0.907429	-3.064101	1.621664
C	-2.293200	-4.149555	2.847882
C	-2.813125	-3.144235	4.949233
H	-1.817198	-1.323617	5.406894
H	-2.406109	-4.960190	2.137917
C	-2.988563	-4.171359	4.043095
H	-3.347381	-3.153295	5.891827
H	-3.657034	-4.992848	4.272006

Optimized geometry for 4d based on calculated minima.

125
Sr_oct2_sad_pi_AB__lcwpbe_GD3BJ_internal_Def2TZVP_singlet_optfreq_16core_ultrafine_benzene_FREQ.xyz

Sr	-2.006679	-0.241789	-0.663028
Sr	1.249135	0.154350	0.522359
O	-0.813492	1.715199	0.244458
C	-0.648050	3.030215	0.508015
C	-0.219205	3.169085	1.992472
9	0.918470	2.476020	2.224639
9	-1.157472	2.649366	2.784350
C	0.492440	3.562746	-0.405997
9	1.533219	2.700768	-0.416214
9	1.006059	4.745035	-0.058698
9	0.070721	3.660619	-1.668556
C	-1.908036	3.884462	0.327203
C	-1.882074	5.256453	0.121404
C	-3.058914	5.967594	-0.019737
C	-3.133791	3.252187	0.416604
H	-0.945645	5.792228	0.066123
H	-3.017011	7.036062	-0.193421
H	-3.165807	2.187446	0.586347
O	3.510347	0.213413	0.712182
C	4.758969	0.433279	1.141303
C	5.049777	1.948385	0.983355
9	4.110665	2.697580	1.575086
9	5.045218	2.272853	-0.316530
9	6.231224	2.352987	1.480874
C	4.818208	0.024570	2.636811
9	4.615381	-1.293827	2.748130
9	3.858744	0.628301	3.352593
9	5.981774	0.295422	3.251971
C	5.819322	-0.367417	0.369977
C	5.373437	-1.210885	-0.630156
C	6.265736	-1.978846	-1.354187
C	7.619433	-1.906233	-1.090038
C	8.073552	-1.059004	-0.095797
C	7.180795	-0.295527	0.632036
H	7.557820	0.355088	1.408234
H	9.133553	-0.992089	0.120319
H	8.321249	-2.507674	-1.656444
H	5.895722	-2.634776	-2.133000
H	4.312128	-1.249407	-0.833481
O	0.226409	-0.303723	-1.726690
C	0.755191	-1.124705	-2.667646
C	0.757229	-2.541485	-2.045322
9	-0.465249	-2.845239	-1.569354
9	1.107380	-3.551008	-2.844459
9	1.596777	-2.548068	-0.999837
C	-0.150128	-1.082613	-3.920405
9	0.149501	-2.002259	-4.842590
9	-0.074571	0.113187	-4.505405
9	-1.447999	-1.268165	-3.593613
C	2.166226	-0.724075	-3.111045
C	2.580423	0.562094	-2.818151
C	3.836287	1.002966	-3.188036
C	4.688643	0.161060	-3.872684
C	4.271134	-1.116716	-4.194788
C	3.018007	-1.560536	-3.819553
H	2.716155	-2.562478	-4.088937
H	4.931489	-1.782598	-4.737578
H	5.680432	0.496479	-4.150417
H	4.149109	2.006043	-2.927987
H	1.897871	1.225995	-2.307483
O	-0.722151	-1.268811	1.194655
9	1.520028	-2.658127	1.988562
9	0.737632	-0.343706	3.180594
O	-3.600545	-2.138335	-1.831899
C	-5.073860	-2.880811	-3.540151
H	-6.143660	-2.828065	-3.734484
H	-4.559263	-2.967788	-4.497746
C	-4.571138	-1.663106	-2.778194
H	-4.101387	-0.927087	-3.430640
H	-5.368481	-1.175657	-2.215412
O	-3.215304	1.306475	-2.377080
C	-4.534083	1.851562	-2.474604
H	-5.181065	1.140801	-2.992506
H	-4.905624	1.991535	-1.460716
C	-4.411796	3.168565	-3.233049
H	-4.996525	3.958669	-2.765642
H	-4.760631	3.048696	-4.259352
C	-2.917224	3.454342	-3.207536
H	-2.637373	3.965432	-2.287395
H	-2.583121	4.051430	-4.054323
C	-2.338501	2.061856	-3.222236
H	-1.332145	1.984057	-2.815160
H	-2.349409	1.631494	-4.227921
O	-4.128182	-0.000640	0.852963
9	-0.002441	4.420961	2.395044
C	-4.279461	5.326252	0.070239
C	-4.311525	3.964758	0.299788
C	-0.751667	-2.006623	2.330236
C	0.287755	-3.151752	2.202023
C	-0.344937	-1.082012	3.511210
C	-3.370188	-3.529319	-2.080067
C	-4.679690	-4.033576	-2.632013
C	-5.469479	-0.400605	0.546638
C	-4.029711	0.269357	2.259129
H	-5.200674	5.887687	-0.030287
H	-5.262166	3.451529	0.388554
C	-2.114539	-2.652386	2.596132
9	-0.013444	-3.925326	1.157767
9	0.353717	-3.944148	3.275617

9	-1.320896	-0.216825	3.787804
9	-0.028170	-1.710801	4.645751
H	-2.566136	-3.642098	-2.811428
H	-3.063656	-4.000595	-1.148913
H	-4.574073	-4.980330	-3.158754
H	-5.405261	-4.162906	-1.826388
H	-5.849845	0.267727	-0.228579
H	-5.453515	-1.419845	0.159231
C	-6.260921	-0.276385	1.838894
C	-5.420739	0.692421	2.655520
H	-3.734665	-0.643812	2.777835
H	-3.259868	1.024162	2.406406
C	-2.858970	-3.011235	1.486707
C	-2.640095	-2.895383	3.855518
H	-6.307440	-1.241632	2.344412
H	-7.278367	0.069076	1.663731
H	-5.604005	1.725858	2.355939
H	-5.589197	0.607396	3.727521
H	-2.425752	-2.863021	0.508237
C	-4.114799	-3.566840	1.617357
C	-3.903218	-3.442938	3.991393
H	-2.081535	-2.652259	4.748222
H	-4.678150	-3.842146	0.733391
C	-4.648442	-3.773748	2.875914
H	-4.305178	-3.612477	4.983021
H	-5.636852	-4.203402	2.987749

Optimized geometry for 4e based on calculated minima.

125

Sr_oct2_sad_pi_ABC__lcwpbe_GD3BJ_internal_Def2TZVP_singlet_optfreq_16core_ultrafine_benzene_FREQ.xyz

Sr	2.047295	-0.708616	0.164047
Sr	-1.080028	0.793501	-0.322841
O	1.391798	1.617341	-0.340911
C	1.616433	2.946527	-0.450945
C	1.046172	3.413128	-1.817687
9	-0.272512	3.122596	-1.905029
9	1.650328	2.760882	-2.809374
C	0.845817	3.661834	0.694402
9	-0.410188	3.165942	0.803959
9	0.696685	4.980658	0.554619
9	1.438701	3.442200	1.868177
C	3.094260	3.355522	-0.430248
C	4.011728	2.442449	-0.914872
C	5.354099	2.757105	-0.997135
C	3.549956	4.600379	-0.020384
H	3.658744	1.478084	-1.243817
H	6.055612	2.033904	-1.395915
H	2.866852	5.350504	0.350378
O	-3.298890	1.307696	-0.257231
C	-4.599878	1.283796	-0.577679
C	-5.294580	2.533428	0.027103
9	-4.804180	3.676445	-0.470475
9	-5.099370	2.567034	1.350142
9	-6.623336	2.557844	-0.170304
C	-4.701807	1.344479	-2.126986
9	-4.321410	0.177183	-2.660571
9	-3.889063	2.280048	-2.631611
9	-5.931278	1.615620	-2.604002
C	-5.320366	0.047224	-0.019151
C	-4.800752	-0.502134	1.141397
C	-5.381214	-1.614749	1.717953
C	-6.488956	-2.201513	1.134122
C	-7.016168	-1.655541	-0.020859
C	-6.442305	-0.532598	-0.589054
H	-6.879821	-0.121479	-1.488171
H	-7.885352	-2.104546	-0.487420
H	-6.940947	-3.080970	1.578104
H	-4.957339	-2.027212	2.625408
H	-3.930215	-0.042930	1.590368
O	0.037309	-0.275370	1.626319
C	-0.393094	-0.862815	2.768647
C	-0.661588	-2.361777	2.460933
9	-1.815993	-2.502154	1.803742
9	-0.713594	-3.165905	3.532713
9	0.299919	-2.862296	1.666987
C	0.732256	-0.748051	3.824283
9	1.869600	-1.325420	3.376478
9	0.449061	-1.308407	5.002233
9	1.016945	0.534242	4.054270
C	-1.635963	-0.201710	3.371255
C	-1.796382	1.155468	3.151264
C	-2.892989	1.830340	3.649320
C	-3.840953	1.152701	4.390414
C	-3.672231	-0.195561	4.642152
C	-2.575557	-0.870857	4.140707
H	-2.471715	-1.926123	4.352929
H	-4.408486	-0.734386	5.226590
H	-4.712296	1.673128	4.768617
H	-3.010593	2.886866	3.444450
H	-1.038608	1.688046	2.593816
O	0.095338	-1.203504	-1.284747
9	0.622469	-3.382383	-4.030314
9	-0.255352	-0.921165	-4.806760
O	3.171107	-2.958261	0.970431
C	3.315538	-4.309466	2.850246
H	4.007732	-4.877348	3.469758
H	2.543374	-3.892877	3.494890
C	4.007616	-3.192941	2.098460
H	4.096673	-2.262190	2.652846
H	5.001216	-3.503064	1.750974
O	4.058104	0.163374	1.591060
C	5.463936	0.106672	1.325243
H	5.823059	-0.905512	1.521436
H	5.608734	0.329806	0.269703
C	6.117267	1.144657	2.223998
H	6.941419	1.649922	1.722849
H	6.504949	0.676866	3.130271
C	4.966027	2.078931	2.561289
H	4.807200	2.803549	1.763661
H	5.111857	2.616119	3.497000
C	3.806056	1.118379	2.629033
H	2.838546	1.575365	2.437419
H	3.770732	0.600160	3.591089
O	4.192823	-0.952896	-1.548419
9	1.175195	4.718198	-2.055936
C	5.803847	3.992873	-0.574851
C	4.895966	4.910293	-0.082091
C	-0.245345	-1.730233	-2.486893
C	0.668486	-2.944679	-2.767507
C	-0.004559	-0.615514	-3.534373
C	2.770879	-4.246935	0.498716
C	2.690417	-5.147804	1.731110
C	5.187974	-1.965738	-1.441113
C	4.385330	-0.403528	-2.851692
H	6.856591	4.242276	-0.632482
H	5.232945	5.884060	0.252193
C	-1.696881	-2.223910	-2.563104
9	0.332035	-3.967566	-1.975191
9	1.956868	-2.664221	-2.498236

9	-0.794367	0.433465	-3.219785
9	1.258574	-0.175787	-3.488644
H	1.822992	-4.134036	-0.019981
H	3.518799	-4.609422	-0.213569
H	1.660196	-5.409882	1.962390
H	3.240062	-6.073070	1.564222
H	6.158895	-1.491231	-1.256913
H	4.936017	-2.598066	-0.594422
C	5.175610	-2.674204	-2.785681
C	4.683880	-1.591434	-3.756575
H	3.495498	0.153921	-3.126896
H	5.236496	0.284736	-2.818746
C	-2.316611	-2.610023	-3.744197
C	-2.396684	-2.333235	-1.376273
H	4.483518	-3.514229	-2.766103
H	6.162082	-3.056560	-3.043467
H	5.430739	-1.331914	-4.505125
H	3.785687	-1.914895	-4.277942
H	-1.792821	-2.567273	-4.687697
C	-3.622092	-3.061873	-3.727298
C	-3.702049	-2.785022	-1.358501
H	-1.896053	-2.086322	-0.451704
H	-4.096913	-3.347861	-4.658399
C	-4.321090	-3.144993	-2.537668
H	-4.238289	-2.841189	-0.419965
H	-5.349163	-3.486634	-2.529378

References

(1) Smith, M.; Li, Z.; Landry, L.; Merz, K. M.; Li, P. Consequences of Overfitting the van Der Waals Radii of Ions. *J. Chem. Theory Comput.* **2023**, *19* (7), 2064–2074. https://doi.org/10.1021/acs.jctc.2c01255.

(2) Mantina, M.; Chamberlin, A. C.; Valero, R.; Cramer, C. J.; Truhlar, D. G. Consistent van Der Waals Radii for the Whole Main Group. *J. Phys. Chem. A* **2009**, *113* (19), 5806–5812. https://doi.org/10.1021/jp8111556.

(3) Long, J.; Liu, Y.; He, Z.; Tan, S.; Xiong, F.; Xu, H.; Wang, W.; Zhang, G.; Yang, Z.; An, Q. Redesigning Solvation Structure toward Passivation-Free Magnesium Metal Batteries. *ACS Nano* **2024**, *18* (23), 15239–15248. https://doi.org/10.1021/acsnano.4c03968.

(4) Kögel, J. F.; Feige, F.; Duvinage, D.; Lork, E.; Timoshkin, A. Y.; Beckmann, J. Metal Complexes of the Perfluorinated Trityl Alkoxide $[(C_6F_5)_3CO]^-$. *Eur J Inorg Chem* **2022**, *2022* (4), e202100872. https://doi.org/10.1002/ejic.202100872.

(5) Roesky, H. W.; Scholz, M.; Noltemeyer, M. Über Reaktionen Des 2,4,6-Tris(Trifluormethyl)Phenols Mit Verbindungen von Hauptgruppen- Und Nebengruppen-Elementen (Li, Na, Mg, Ca, Ba, Ge, Sn Und Ti, W, Mn, Cd). *Chem. Ber.* **1990**, *123* (12), 2303–2309.

(6) Tuskaev, V. A.; Kurmaev, D. A.; Gagieva, S. Ch.; Golubev, E. K.; Buzin, M. I.; Khrustalev, V. N.; Aksenova-Soloveva, S. A.; Gracheva, A. V.; Bulychev, B. M. Impact of the Method of Mg^{2+} Cation Introducing into the Titanium Post-Metallocene Catalytic System on Their Activity. *Polym. Bull.* **2025**, *82* (4), 1131–1147. https://doi.org/10.1007/s00289-024-05558-6.

(7) Buchanan, W. D.; Guino-o, M. A.; Ruhlandt-Senge, K. Highly Volatile Alkaline Earth Metal Fluoroalkoxides. *Inorg. Chem.* **2010**, *49* (15), 7144–7155. https://doi.org/10.1021/ic100960z.

(8) Roşca, S.-C.; Roisnel, T.; Dorcet, V.; Carpentier, J.-F.; Sarazin, Y. Potassium and Well-Defined Neutral and Cationic Calcium Fluoroalkoxide Complexes: Structural Features and Reactivity. *Organometallics* **2014**, *33* (20), 5630–5642. https://doi.org/10.1021/om500343w.

(9) Roşca, S.; Dinoi, C.; Caytan, E.; Dorcet, V.; Etienne, M.; Carpentier, J.; Sarazin, Y. Alkaline Earth–Olefin Complexes with Secondary Interactions. *Chemistry A European J* **2016**, *22* (19), 6505–6509. https://doi.org/10.1002/chem.201601096.

(10) Roşca, S.-C.; Dorcet, V.; Roisnel, T.; Carpentier, J.-F.; Sarazin, Y. Tethered Cationic Alkaline Earth – Olefin Complexes. *Dalton Trans.* **2017**, *46* (43), 14785–14794. https://doi.org/10.1039/C7DT03300A.

(11) Roşca, S.-C.; Caytan, E.; Dorcet, V.; Roisnel, T.; Carpentier, J.-F.; Sarazin, Y. π Ligands in Alkaline Earth Complexes. *Organometallics* **2017**, *36* (7), 1269–1277. https://doi.org/10.1021/acs.organomet.7b00006.

(12) Roşca, S.-C.; Dorcet, V.; Carpentier, J.-F.; Sarazin, Y. Secondary Interactions – Cement in Trinuclear Calcium Complexes. *Inorganica Chimica Acta* **2018**, *475*, 59–64. https://doi.org/10.1016/j.ica.2017.08.038.
(13) Hammoud, J.; Abou-Khalil, F.; Roisnel, T.; Dorcet, V.; Bour, C.; Gandon, V.; Lebœuf, D.; Carpentier, J.-F.; Sarazin, Y. Alkaline-Earth Complexes with Macrocyclic-Functionalised Bis(Phenolate)s and Bis(Fluoroalkoxide)s. *Dalton Trans.* **2020**, *49* (37), 13017–13028. https://doi.org/10.1039/D0DT02573A.
(14) Sarazin, Y.; Liu, B.; Roisnel, T.; Maron, L.; Carpentier, J.-F. Discrete, Solvent-Free Alkaline-Earth Metal Cations: Metal···Fluorine Interactions and ROP Catalytic Activity. *J. Am. Chem. Soc.* **2011**, *133* (23), 9069–9087. https://doi.org/10.1021/ja2024977.
(15) Chi, Y.; Ranjan, S.; Chung, P.-W.; Hsieh, H.-Y.; Peng, S.-M.; Lee, G.-H. Alkaline-Earth Metal Fluoroalkoxide Complexes with Multi-Coordinated Polyether Appendage: Synthesis and Characterization. *Inorganica Chimica Acta* **2002**, *334*, 172–182. https://doi.org/10.1016/S0020-1693(02)00860-5.
(16) Vincent, H.; Labrize, F.; Hubert-Pfalzgraf, L. G. Synthesis and Molecular Structure of $Ba_5(\mu_5\text{-}OH)[\mu_3\text{-}OCH(CF_3)_2]_4[\mu_2\text{-}OCH(CF_3)_2]_4\ [OCH(CF_3)_2](THF)_4(H_2O)\cdot THF$: A Source of Barium Fluoride. *Polyhedron* **1994**, *13* (24), 3323–3327. https://doi.org/10.1016/S0277-5387(00)83114-9.
(17) Chi, Y.; Ranjan, S.; Chou, T.-Y.; Liu, C.-S.; Peng, S.-M.; Lee, G.-H. Preparation and Characterization of Volatile Alkaline-Earth Metal Complexes with Multiply Coordinated Aminoalkoxide Ligands. *J. Chem. Soc., Dalton Trans.* **2001**, No. 17, 2462–2466. https://doi.org/10.1039/b103310g.
(18) Liu, B.; Roisnel, T.; Sarazin, Y. Well-Defined, Solvent-Free Cationic Barium Complexes: Synthetic Strategies and Catalytic Activity in the Ring-Opening Polymerization of Lactide. *Inorganica Chimica Acta* **2012**, *380*, 2–13. https://doi.org/10.1016/j.ica.2011.09.020.
(19) Hunter, C. A.; Sanders, J. K. M. The Nature of Pi-Pi Interactions. *J. Am. Chem. Soc.* **1990**, *112* (14), 5525–5534. https://doi.org/10.1021/ja00170a016.
(20) Janiak, C. A Critical Account on π–π Stacking in Metal Complexes with Aromatic Nitrogen-Containing Ligands †. *J. Chem. Soc., Dalton Trans.* **2000**, No. 21, 3885–3896. https://doi.org/10.1039/b003010o.
(21) Meyer, E.; Castellano, R.; Diederich, F. Interactions with Aromatic Rings in Chemical and Biological Recognition. *Angew Chem Int Ed* **2003**, *42* (11), 1211–1250.
(22) Nayak, S. K.; Sathishkumar, R.; Row, T. N. G. Directing Role of Functional Groups in Selective Generation of C–H···π Interactions: In Situ Cryo-Crystallographic Studies on Benzyl Derivatives. *CrystEngComm* **2010**, *12* (10), 3112. https://doi.org/10.1039/c001190h.
(23) Xiao, Y.; Woods, R. J. Protein–Ligand CH−π Interactions: Structural Informatics, Energy Function Development, and Docking Implementation. *J. Chem. Theory Comput.* **2023**, *19* (16), 5503–5515. https://doi.org/10.1021/acs.jctc.3c00300.
(24) Platzer, G.; Mayer, M.; Beier, A.; Brüschweiler, S.; Fuchs, J. E.; Engelhardt, H.; Geist, L.; Bader, G.; Schörghuber, J.; Lichtenecker, R.; Wolkerstorfer, B.; Kessler, D.; McConnell, D. B.; Konrat, R. PI by NMR: Probing CH–π Interactions in Protein–Ligand Complexes by NMR Spectroscopy. *Angew Chem Int Ed* **2020**, *59* (35), 14861–14868. https://doi.org/10.1002/anie.202003732.
(25) Sheldrick, G. M. A Short History of *SHELX*. *Acta Crystallogr A Found Crystallogr* **2008**, *64* (1), 112–122. https://doi.org/10.1107/S0108767307043930.
(26) Macrae, C. F.; Sovago, I.; Cottrell, S. J.; Galek, P. T. A.; McCabe, P.; Pidcock, E.; Platings, M.; Shields, G. P.; Stevens, J. S.; Towler, M.; Wood, P. A. *Mercury 4.0* : From Visualization to Analysis, Design and Prediction. *J Appl Crystallogr* **2020**, *53* (1), 226–235. https://doi.org/10.1107/S1600576719014092.

(27) Alvarez, S. A Cartography of the van Der Waals Territories. *Dalton Trans.* **2013**, *42* (24), 8617. https://doi.org/10.1039/c3dt50599e.
(28) Lavin, C. M.; Allis, D. G.; Gillett-Kunnath, M. M.; Clements, A.; Goos, A. G.; Woods, J. J.; Hager, P.; Logan, D. S.; Ruhlandt-Senge, K. Examination of Metal-π Interactions in the Coordination Chemistry of Heavy Alkaline Earth Metal Tetraarylborates. *Journal of Coordination Chemistry* **2025**, *78* (1–3), 233–250. https://doi.org/10.1080/00958972.2025.2453065.
(29) Long, J.; Tan, S.; Wang, J.; Xiong, F.; Cui, L.; An, Q.; Mai, L. Revealing the Interfacial Chemistry of Fluoride Alkyl Magnesium Salts in Magnesium Metal Batteries. *Angew Chem Int Ed* **2023**, *62* (21), e202301934. https://doi.org/10.1002/anie.202301934.
(30) Terzopoulos, A.; Marinho Fonseca, R.; Bond, A. D.; Grey, C. P.; Wright, D. S. The Role of Li···F Interactions in Lithium and Heterobimetallic Lithium–Magnesium Complexes of the Hexafluoroisopropoxide Anion. *Polyhedron* **2025**, *267*, 117322. https://doi.org/10.1016/j.poly.2024.117322.
(31) Armstrong, D. R.; Clegg, W.; García-Alvarez, P.; McCall, M. D.; Nuttall, L.; Kennedy, A. R.; Russo, L.; Hevia, E. Shedding New Light on $ZnCl_2$ -Mediated Addition Reactions of Grignard Reagents to Ketones: Structural Authentication of Key Intermediates and Diffusion-Ordered NMR Studies. *Chemistry A European J* **2011**, *17* (16), 4470–4479. https://doi.org/10.1002/chem.201002544.
(32) Leon, N. J.; Mankad, N. P. CCDC 1998385: Experimental Crystal Structure Determination, 2021. https://doi.org/10.5517/ccdc.csd.cc252h1t.
(33) Bruker APEX2 and SAINT, Bruker AXS Inc.: Madison, Wisconsin, USA, 2012.
(34) Sheldrick, G. M. CELL_NOW, Georg-August-Universität, Göttingen, Germany, 2008.
(35) Sheldrick, G. M. SADABS, Version 2.10, Siemens Area Detector Correction, 2003.
(36) Blessing, R. H. An Empirical Correction for Absorption Anisotropy. *Acta Crystallogr A Found Crystallogr* **1995**, *51* (1), 33–38. https://doi.org/10.1107/S0108767394005726.
(37) Sheldrick, G. M. *SHELXT* – Integrated Space-Group and Crystal-Structure Determination. *Acta Crystallogr A Found Adv* **2015**, *71* (1), 3–8. https://doi.org/10.1107/S2053273314026370.
(38) Sheldrick, G. M. Phase Annealing in SHELX-90: Direct Methods for Larger Structures. *Acta Crystallogr A Found Crystallogr* **1990**, *46* (6), 467–473. https://doi.org/10.1107/S0108767390000277.
(39) Sheldrick, G. M. Crystal Structure Refinement with *SHELXL*. *Acta Crystallogr C Struct Chem* **2015**, *71* (1), 3–8. https://doi.org/10.1107/S2053229614024218.
(40) Hübschle, C. B.; Sheldrick, G. M.; Dittrich, B. *ShelXle* : A Qt Graphical User Interface for *SHELXL*. *J Appl Crystallogr* **2011**, *44* (6), 1281–1284. https://doi.org/10.1107/S0021889811043202.
(41) Spek, A. L. *PLATON* SQUEEZE: A Tool for the Calculation of the Disordered Solvent Contribution to the Calculated Structure Factors. *Acta Crystallogr C Struct Chem* **2015**, *71* (1), 9–18. https://doi.org/10.1107/S2053229614024929.
(42) Spek, A. L. Structure Validation in Chemical Crystallography. *Acta Crystallogr D Biol Crystallogr* **2009**, *65* (2), 148–155. https://doi.org/10.1107/S090744490804362X.
(43) Müller, P.; Herbst-Irmer, R.; Spek, A. L.; Schneider, T. R.; Sawaya, M. R. Crystal Structure Refinement: A Crystallographer's Guide to SHELXL; Oxford University Press, 2006; p 232.
(44) Van Der Sluis, P.; Spek, A. L. BYPASS: An Effective Method for the Refinement of Crystal Structures Containing Disordered Solvent Regions. *Acta Crystallogr A Found Crystallogr* **1990**, *46* (3), 194–201. https://doi.org/10.1107/S0108767389011189.
(45) Macrae, C. F.; Bruno, I. J.; Chisholm, J. A.; Edgington, P. R.; McCabe, P.; Pidcock, E.; Rodriguez-Monge, L.; Taylor, R.; Van De Streek, J.; Wood, P. A. *Mercury CSD 2.0* – New Features for the Visualization and Investigation of Crystal Structures. *J Appl Crystallogr* **2008**, *41* (2), 466–470. https://doi.org/10.1107/S0021889807067908.

(46) Putz, H.; Brandenburg, K. Diamond - Crystal and Molecular Structure Visualization.
(47) Groom, C. R.; Bruno, I. J.; Lightfoot, M. P.; Ward, S. C. The Cambridge Structural Database. *Acta Crystallogr B Struct Sci Cryst Eng Mater* **2016**, *72* (2), 171–179. https://doi.org/10.1107/S2052520616003954.
(48) Allen, F. H. The Cambridge Structural Database: A Quarter of a Million Crystal Structures and Rising. *Acta Crystallogr B Struct Sci* **2002**, *58* (3), 380–388. https://doi.org/10.1107/S0108768102003890.
(49) Allen, F. H.; Johnson, O.; Shields, G. P.; Smith, B. R.; Towler, M. CIF Applications. XV. *enCIFer*: A Program for Viewing, Editing and Visualizing CIFs. *J Appl Crystallogr* **2004**, *37* (2), 335–338. https://doi.org/10.1107/S0021889804003528.
(50) Westrip, S. P. *publCIF*: Software for Editing, Validating and Formatting Crystallographic Information Files. *J Appl Crystallogr* **2010**, *43* (4), 920–925. https://doi.org/10.1107/S0021889810022120.
(51) Dolomanov, O. V.; Bourhis, L. J.; Gildea, R. J.; Howard, J. A. K.; Puschmann, H. *OLEX2*: A Complete Structure Solution, Refinement and Analysis Program. *J Appl Crystallogr* **2009**, *42* (2), 339–341. https://doi.org/10.1107/S0021889808042726.
(52) Pracht, P.; Grimme, S.; Bannwarth, C.; Bohle, F.; Ehlert, S.; Feldmann, G.; Gorges, J.; Müller, M.; Neudecker, T.; Plett, C.; Spicher, S.; Steinbach, P.; Wesołowski, P. A.; Zeller, F. CREST—A Program for the Exploration of Low-Energy Molecular Chemical Space. *The Journal of Chemical Physics* **2024**, *160* (11), 114110. https://doi.org/10.1063/5.0197592.
(53) Bannwarth, C.; Caldeweyher, E.; Ehlert, S.; Hansen, A.; Pracht, P.; Seibert, J.; Spicher, S.; Grimme, S. Extended TIGHT-BINDING Quantum Chemistry Methods. *WIREs Comput Mol Sci* **2021**, *11* (2), e1493. https://doi.org/10.1002/wcms.1493.
(54) Bannwarth, C.; Ehlert, S.; Grimme, S. GFN2-xTB—An Accurate and Broadly Parametrized Self-Consistent Tight-Binding Quantum Chemical Method with Multipole Electrostatics and Density-Dependent Dispersion Contributions. *J. Chem. Theory Comput.* **2019**, *15* (3), 1652–1671. https://doi.org/10.1021/acs.jctc.8b01176.
(55) Becke, A. D. Density-Functional Thermochemistry. III. The Role of Exact Exchange. *The Journal of Chemical Physics* **1993**, *98* (7), 5648–5652. https://doi.org/10.1063/1.464913.
(56) Hehre, W. J.; Ditchfield, R.; Pople, J. A. Self—Consistent Molecular Orbital Methods. XII. Further Extensions of Gaussian—Type Basis Sets for Use in Molecular Orbital Studies of Organic Molecules. *The Journal of Chemical Physics* **1972**, *56* (5), 2257–2261. https://doi.org/10.1063/1.1677527.
(57) Grimme, S.; Ehrlich, S.; Goerigk, L. Effect of the Damping Function in Dispersion Corrected Density Functional Theory. *J Comput Chem* **2011**, *32* (7), 1456–1465. https://doi.org/10.1002/jcc.21759.
(58) Henderson, T. M.; Izmaylov, A. F.; Scalmani, G.; Scuseria, G. E. Can Short-Range Hybrids Describe Long-Range-Dependent Properties? *The Journal of Chemical Physics* **2009**, *131* (4), 044108. https://doi.org/10.1063/1.3185673.
(59) Weigend, F. Accurate Coulomb-Fitting Basis Sets for H to Rn. *Phys. Chem. Chem. Phys.* **2006**, *8* (9), 1057. https://doi.org/10.1039/b515623h.
(60) Tomasi, J.; Mennucci, B.; Cammi, R. Quantum Mechanical Continuum Solvation Models. *Chem. Rev.* **2005**, *105* (8), 2999–3094. https://doi.org/10.1021/cr9904009.
(61) M. J. Frisch; G. W. Trucks; J. R. Cheeseman; M. Caricato; H.P. Hratchian; X. Li; V. Barone; J. Bloino; G. Zheng; T. Vreven; J. A. Montgomery; G. A. Petersson; G. E. Scuseria; H. B. Schlegel; H. Nakatsuji; A. F. Izmaylov; R. L. Martin; J. L. Sonnenberg; J. E. Peralta; J. J. Heyd; E. Brothers; F. Ogliaro; M. Bearpark; M. A. Robb; B. Mennucci; K. N. Kudin; V. N. Staroverov; R. Kobayashi; J. Normand; A. Rendell; R. Gomperts; V. G. Zakrzewski; M. Hada; M. Ehara; K. Toyota; R. Fukuda; J. Hasegawa; M. Ishida; T. Nakajima; Y. Honda; O. Kitao; H. Nakai. Gaussian 09, 2016.

(62) Humphrey, W.; Dalke, A.; Schulten, K. VMD: Visual Molecular Dynamics. *Journal of Molecular Graphics* **1996**, *14* (1), 33–38. https://doi.org/10.1016/0263-7855(96)00018-5.
(63) POV-Ray - Persistence of Vision Raytracer, 2006.
